# Facile N–C bond cleavage and arene reduction by a transient uranium(ii) complex†

**DOI:** 10.1039/d5sc03694a

**Published:** 2025-07-02

**Authors:** R. A. Keerthi Shivaraam, Leonor Maria, Thayalan Rajeshkumar, Rosario Scopelliti, Ivica Živković, Andrzej Sienkiewicz, Laurent Maron, Marinella Mazzanti

**Affiliations:** a Insititut des Sciences et Ingénierie Chimiques, Ecole Polytechnique Fédérale de Lausanne (EPFL) 1015 Lausanne Switzerland marinella.mazzanti@epfl.ch; b Centro de Química Estrutural, Institute of Molecular Sciences, Instituto Superior Técnico, Universidade de Lisboa 2695-066 Bobadela Portugal; c Laboratoire de Physique et Chimie des Nano-objets, Institut National des Sciences Appliquées 31077 Toulouse Cedex 4 France; d X-Ray Diffraction and Surface Analytics Platform, Institute of Chemical Sciences and Engineering (ISIC), École Polytechnique Fédérale de Lausanne (EPFL) 1015 Lausanne Switzerland; e Laboratory for Quantum Magnetism, Institute of Physics, Ecole Polytechnique Fédérale de Lausanne (EPFL) 1015 Lausanne Switzerland; f ADSresonances SàrL 1920 Martigny Switzerland

## Abstract

Complexes of uranium(ii) remain extremely rare and their reactivity is practically unexplored. Here we report that the reduction of the heteroleptic bis-aryloxide U(iii) complex [U(*κ*^6^-{(^*t*Bu2^ArO)_2_Me_2_-cyclam})I], A, yields a rare and highly reactive U(ii) intermediate that enables a rare example of intramolecular uranium mediated N–C cleavage and effects arene reduction resulting in the isolation of the U(iv) complex [U{*κ*^5^-((^*t*^Bu_2_ArO)Me_2_-cyclam)}{*κ*^2^-(^*t*^Bu_2_ArOCH_2_)}] (2) and of the inverse–sandwich complex [{U(*κ*^5^-{(^*t*Bu2^ArO)_2_Me_2_-cyclam})}_2_(*μ-η*^6^:*η*^6^-benzene)] (3) respectively. Moreover, the U(ii) solvent-dependent reactivity results in the formation of a putative U–N_2_ complex in diethyl ether. Computational, EPR and magnetic studies indicate the electronic structure of 3 to be an equilibrium between two possible electronic structures very close in energy: (U(iv)–arene^4−^–U(iv) and U(iii)–arene^2−^–U(iii)). These results indicate that polydentate amine-phenolate ligands can be used to access highly reactive U(ii) intermediates and that provides evidence that U(ii) species are involved in the formation of inverse sandwich complexes.

## Abstract 2

A highly reactive U(ii) intermediate supported by a polydentate amine-phenolate ligand undergoes solvent dependent divergent reactivity affording an inverse–sandwich complex in benzene, N–C cleavage in THF and N_2_ activation in diethyl ether.

## Graphical abstract

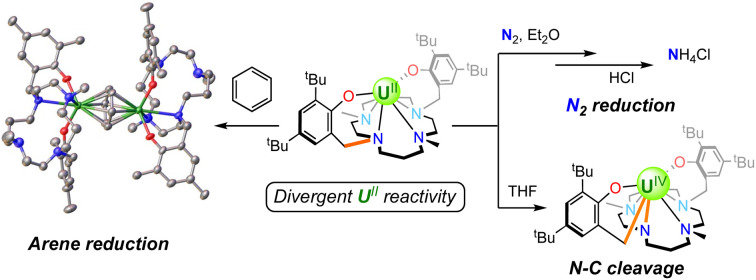

## Introduction

**Fig. 1:**
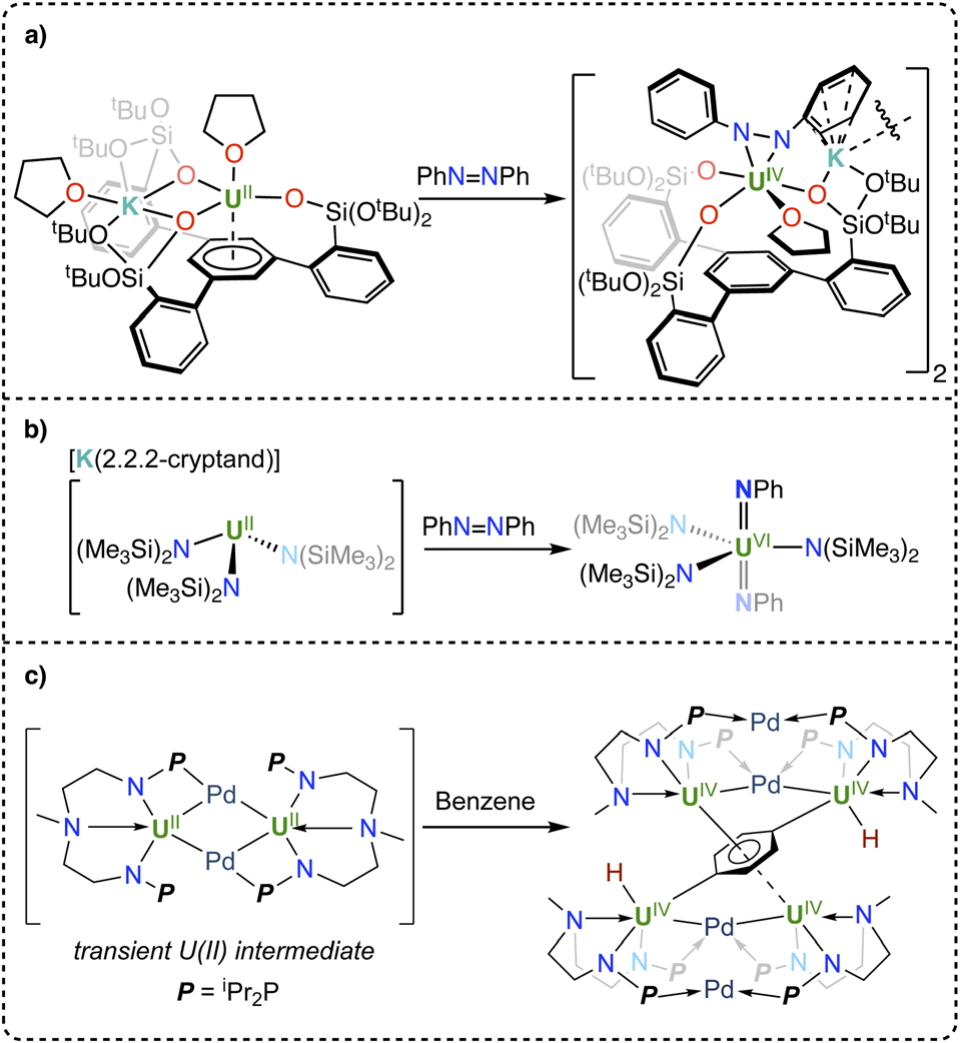
Previously reported oxidative addition reactions featuring U(ii) complexes.

The recent discoveries of molecular compounds of uranium in the +2 oxidation state^1–11^ have opened a route to explore new reactivity in uranium chemistry. Notably, it has resulted in a few unambiguous examples of single-metal oxidative addition examples involving both two and four electron transfer to N=N (*e.g.* in azobenzene, Fig. 1a and b) and C=C (*e.g.* in phenylacetylene) π-bonds.^8,12^ Examples of both intramolecular^13–16^ C–H activation and intermolecular^17^ C–H oxidative addition (Fig. 1c) by putative U(ii) intermediates have also been reported. More recently examples of two-electron oxidative atom and group transfer reactions at a well-defined uranium(ii) centre have also been reported.^9,18^ These examples suggest that oxidative addition reactivity may be easily accessible for uranium compounds in the +2 oxidation state, but the study of these complexes is limited by their scarce number and their high reactivity.

Single-metal oxidative–additions reactions play a key role in d-block metal chemistry and in transition metal mediated catalysis.^19^ In contrast, these reactions are hard to reach in f element chemistry because of their limited access to two electron redox couples and multielectron transfer.^20,21^ Notably, uranium mediated oxidative addition reactions have been for a long time limited to single electron transfer. Fewer cases of N≡N, N=N and N–N oxidative additions involving multiple electron transfer are also known, although the reaction intermediates remain in many case unidentified.^22–27^ Redox active ligands^8,9,28–31^ and sterically induced reductions (SIR)^32,33^ have provided a successful alternative metal–ligand cooperative approach to implement oxidative addition at a single metal centre.

In contrast, examples of metal-based oxidative addition reactions at a single uranium centre, not involving oxidative atom or group transfer, remain scarce^12,20,21^ but recent reports^8,12,17^ indicate that U(ii) compounds are well poised to develop this chemistry.

Moreover, U(ii) species are also likely intermediates in the formation of uranium inverse sandwich complexes;^34–36^ however evidence of their involvement is still lacking due to the high reactivity of putative intermediate species. Notably, prior to the isolation of the first example of a U(ii) complex by Evans in 2013,^2^ attempts to isolate molecular complexes of uranium in the +2 oxidation state by reduction of the U(iii) analogues in aromatic solvents had resulted in the isolation of inverse sandwich complexes with uranium in a formal +2 oxidation state. However, further spectroscopic and computational studies indicated that the electronic structure of these complexes is better described as U(iii)–arene^2−^–U(iii) or U(iv)–arene^4−^–U(iv).^36,37^ The possible involvement of intermediate “U(ii)” species in the formation of inverse sandwich complexes still remains to be proven.

So far, stabilisation of U(ii) species has been attained with bulky cyclopentadienyl derivatives preventing access to the metal centre^2–5,14,15,38^ or with arene anchored polydentate ligands that can establish strong delta bonding interactions with the uranium centre.^1–10^ The only example of a U(ii) complex with a strong N-donor ligand set –N((SiMe_3_)_2_)_3_ that did not present high steric crowding^4^ was isolated at low temperature and was found to be extremely reactive towards C–H activation of the –N(SiMe_3_)_2_ ligand resulting in the cyclometalated U(iii) complex [U{N(SiMe_3_)_2_}_2_(*κ*_2_-C,N–CH_2_SiMe_2_NSiMe_3_)].^39^ Amine-phenolate ligands of different charge, bulk and denticity were shown to be well suited for isolating well defined and highly reactive U(iii) complexes^40,41^ but have so far failed to produce complexes of uranium in lower oxidation state. We became interested in investigating U(iii) to U(ii) reduction using the polydentate aminophenolate ligand (^*t*Bu2^ArO)_2_Me_2_-cyclam. This was prompted by the reported ability of the heteroleptic bis-aryloxide U(iii) precursor [U(*κ*^6^-{(^*t*Bu2^ArO)_2_Me_2_-cyclam})I], A^41^ to cleave azobenzene yielding the U(vi) bis–imido complex [U{(^*t*Bu2^OAr)_2_Me_2_-cyclam}(NPh)_2_] and the U(iv) byproduct [U{(^*t*Bu2^OAr)_2_Me_2_-cyclam}I]I. While a different mechanism was proposed based on the seminal work of Burns,^26^ the possibility of a U(ii) intermediate in this reaction cannot be ruled out. Here we provide evidence that reduction of the complex A produces a highly reactive U(ii) intermediate enabling arene reduction and the first example of uranium(ii) mediated N–C cleavage. We also report the synthesis and reduction of the analogous complex [U(*κ*^6^-{(^*t*Bu2^ArO)_2_Me_2_-cyclam})(OSi(O^*t*^Bu)_3_)] (1) to elucidate the importance of the presence of a reactive halide position for generating neutral U(ii) species supported by non-carbon based ligands. It should be noted that, to date, most reported U(ii) complexes are “ate” complexes probably due to the rarity of stable heteroleptic U(iii) precursors.^3,7^

## Results and discussion

### Synthesis of a heteroleptic U(iii) complex

**Scheme 1:**
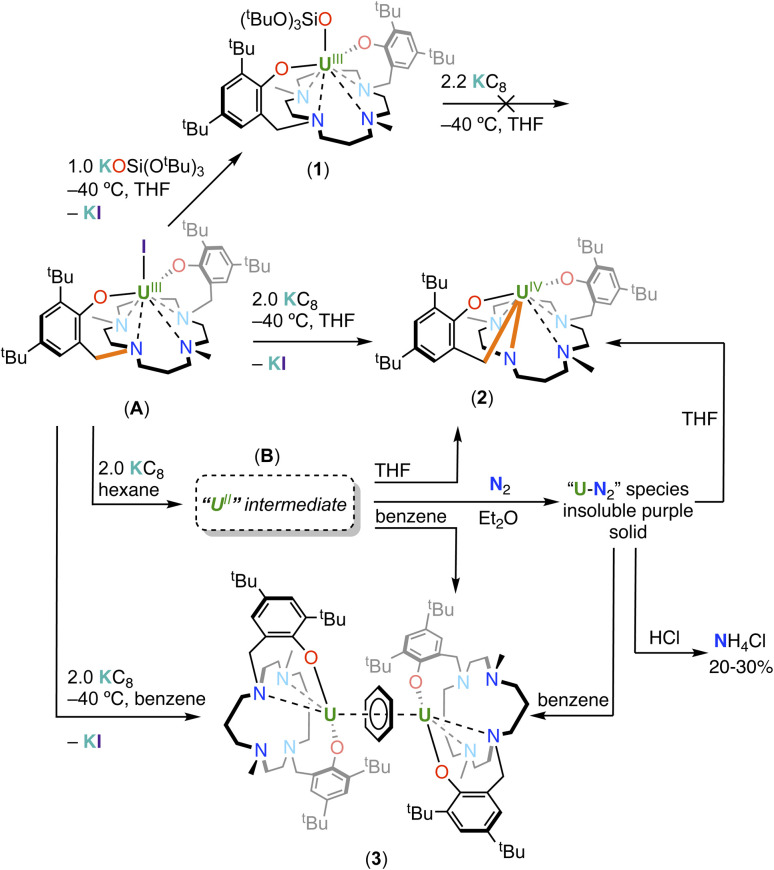
Synthesis of complexes 1, 2 and 3 (bonds involved in oxidative addition are drawn in orange).

With the bis-aryloxide U(iii) precursor [U(*κ*^6^-{(^*t*Bu2^ArO)_2_Me_2_-cyclam})I] (A) in hand, we first prepared the heteroleptic dark red brown U(iii) complex [U(*κ*^6^-{(^*t*Bu2^ArO)_2_Me_2_-cyclam})(OSi(O^*t*^Bu)_3_)] (1) in 56% yield from the salt metathesis reaction between A and KOSi(O^*t*^Bu)_3_ (see Scheme 1 below).

**Fig. 2:**
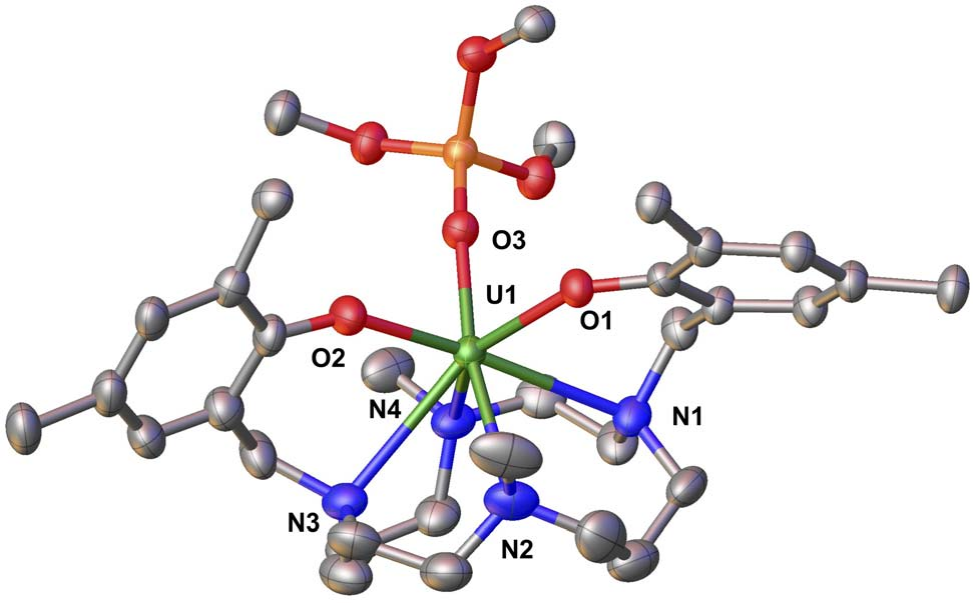
Molecular structure of complex 1 with thermal ellipsoids drawn at the 50% probability level. Methyl groups of ^*t*^Bu group and hydrogen atoms have been omitted for clarity.

Single crystals suitable for X-ray diffraction (Fig. 2) were grown from slow evaporation of a solution of 1 in pentane at −40 °C, while bulk isolation was performed from a concentrated reaction mixture in hexane at −40 °C. The ^1^H NMR spectrum of 1 in *d*_8_-THF at −40 °C (Fig. S4†) displays 35 peaks between 43 ppm and −50 ppm, which corresponds well with the ^1^H NMR spectrum of the crude reaction mixture (Fig. S3†) in *d*_8_-THF at −40 °C, indicating a straightforward ligand displacement reaction. The solid-state molecular structure of 1 (Fig. 2) features a seven-coordinate U(iii) ion bound by two oxygens from the two aryloxide arms, four nitrogen atoms of the cyclam ring and a tris(*tert*-butoxy)siloxide ligand in a distorted pentagonal bipyramidal geometry. The U–O_aryloxide_ bond distances in 1 (2.251(4) Å and 2.309(4) Å), although slightly longer presumably to the presence of the siloxide ligand, are still comparable to the U(iii) precursor A^41^ (2.223(4) Å and 2.263(3) Å). The U–N_cylam_ bond distances in 1 (2.797(6) Å, 2.824(5) Å, 2.909 (6) Å, 2.953(6) Å; average U–N_cylam_ = 2.87(6) Å) are slightly longer than those in A (2.721(5) Å − 2.809(5) Å; average U–N_cylam_ = 2.76(5) Å).

### Reduction of U(iii) complexes

The reduction of the U(iii) complexes A^41^ and 1 was pursued in order to explore the possibility of accessing a uranium(ii) complex. The ^1^H NMR spectrum in *d*_8_-THF of the reaction mixture obtained after reacting 1 with 1–2.2 KC_8_ at −40 °C (Fig. S7a and S8b†) after 14 hours showed the presence of the resonances of 1 and of minor decomposition products suggesting that its reduction was not possible in the investigated conditions.

**Fig. 3:**
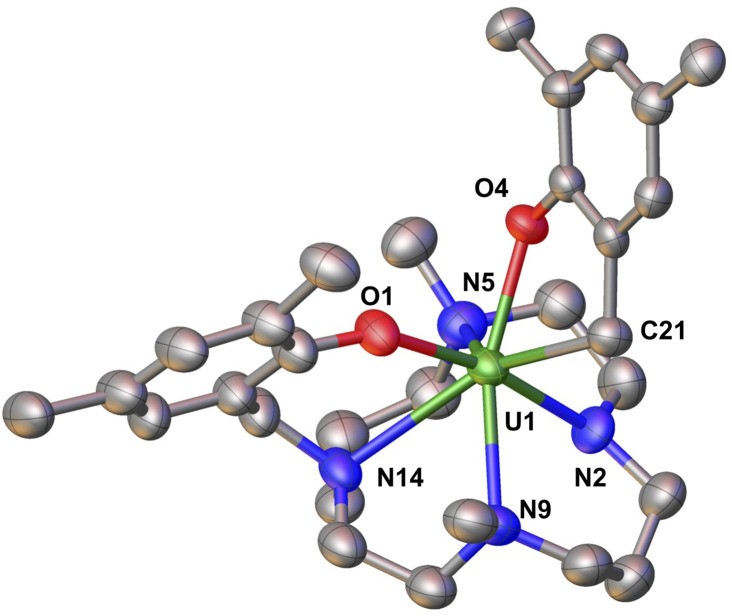
Molecular structure of complex 2 with thermal ellipsoids drawn at the 50% probability level. Methyl groups of ^*t*^Bu group and hydrogen atoms have been omitted for clarity.

In contrast, the reaction of a violet solution of complex A with excess KC_8_ (2.2 equiv.) in *d*_8_-THF at −40 °C led to the complete disappearance of the signals assigned to A in the ^1^H NMR spectrum (Fig. S12†) and the appearance of a new set of resonances. Single crystals of the U(iv) complex [U{*κ*^5^-((^*t*^Bu_2_ArO)Me_2_-cyclam)}{*κ*_2_-(^*t*^Bu_2_ArOCH_2_)}] (2) suitable for X-ray diffraction were isolated from a concentrated reaction mixture in THF at −40 °C (Fig. 3). The easier reduction of A compared to 1 is most likely due to the presence of the reactive iodide ligand that is easily eliminated upon reduction with KC_8_. In contrast, reduction of the complex 1 would require the formation of a “ate” complex which is not accessible in the reducing conditions used.

Analytically pure complex 2 was isolated in 45% yield from a concentrated hexane solution of the reaction mixture. The ^1^H NMR spectrum of 2 in *d*_8_-THF at −40 °C features 39 peaks from 230 ppm to −273 ppm (Fig. S13†). Complex 2 was observed as the major species formed even when the reaction was conducted in the presence of one equiv. of 2.2.2-cryptand. The solid-state structure of 2 shows that the reduction of A with KC_8_ did not lead the formation of a stable divalent uranium species as anticipated, but resulted in a U(iv) complex derived from the reductive C–N cleavage of one ligand arm.

Notably, the benzylic C–N bond connecting the cyclam ring to the aryloxide arm is cleaved forming a new C–U bond. The formation of complex 2 is likely to involve a highly reactive putative U(ii) species that undergoes intramolecular oxidative addition of the C–N bond to yield the respective U(iv) complex. The removal of the bound iodide in A as KI upon reduction opens a coordination site at the uranium centre that likely facilitates the intramolecular oxidative addition reaction, resulting in the seven-coordinate complex in 2. Monitoring the reduction of K_2_(^*t*Bu2^ArO)_2_Me_2_-cyclam with 2.2 equiv. KC_8_ by ^1^H NMR spectroscopy (see ESI and Fig. S29†) did not show any evolution, and only unreacted starting material was observed; thereby ruling out the possibility of a ligand reduction occurring without metal assistance. The proposed oxidative addition mechanism invoking a putative U(ii) species is further corroborated by the stability of the U(iii) complex 1 under similar reducing conditions as those used for the formation of 2.

Stoichiometric oxidative addition of the C–N bond to transition metals is well established and has been used to develop catalytic methodologies that have emerged recently as a powerful strategy for the preparation or utilization of nitrogen-containing compounds.^42–47^ However, complex 2 represents only the second example of an oxidative addition of a C–N bond mediated by a uranium complex^48^ and the first promoted by a monometallic putative uranium(ii) species. In the only reported example of oxidative C–N addition to an f-element, Liddle and coworkers showed that the reduction of the mononuclear U(iv) complex [U(TsXy)(Cl)(THF)] [TsXy = HC-(SiMe_2_NAr)_3_; Ar = 3,5-Me_2_C_6_H_3_] in hexane yielded the bimetallic U(iv)/U(iv) [U{HC(SiMe_2_Ar)_2_(SiMe_2_-*μ*-N)}(*μ-η*^1^:*η*^1^-Ar)U-(TsXy)] as a result of a bimolecular reductive C–N bond cleavage by a putative U(iii) intermediate.^48^ In the latter example only a one-electron transfer from each metal is involved resulting in an overall unchanged oxidation state. In contrast, the formation of 2 requires a two-electron transfer process at a single metal centre, a putative U(ii), which results in the overall oxidation state of the metal increases by +1 on going from U(iii) to U(iv) reminiscent of transition metal promoted C–N oxidative addition.

The solid-state molecular structure of 2 (Fig. 3) reveals a uranium ion in the +4 oxidation state with an overall coordination number of seven, wherein the metal center is bound by three neutral amino nitrogen atoms from the cyclam ring, two anionic oxygens from the aryloxide arms, one anionic amido nitrogen and one monoanionic carbon atom resulting from the benzylic C–N bond cleavage. The U–O_aryloxide_ bond distances in 2 (U–O_aryloxide_ = 2.177(6), 2.219(6) Å) are shorter than those in A (2.223(4) and 2.263(3) Å) in agreement with the ionic radii contraction expected for a U(iv) ion. The U–N_cyclam-amine_ bond distances in 2 (2.599(9) − 2.779(9) Å; av. U–N_cyclam-amine_ = 2.68(2) Å) are also shorter than those in A (2.721(5) Å − 2.809(5) Å; av. U–N_cyclam_ = 2.76(5) Å), but considerably longer than the U–N_cyclam-amido_ bond distance (2.265(6) Å) in 2.^41^ The U–N_cyclam-amido_ bond distance (2.265(6) Å) in 2 compares well with previously reported U(iv) amido bond distances.^49–51^ The newly formed U(iv)–C_cylam_ bond distance (2.505(11) Å) is in the range of previously reported U(iv)-alkyl bond distances (2.4–2.5 Å)^52–57^ and is similar to that found in a eight coordinate U-alkyl compound (2.5134(7) Å).^58^

Given the highly reactive nature of the putative U(ii) species generated upon reduction we sought to investigate if dinitrogen reduction reactivity could compete with N–C cleavage by conducting the reduction of A with KC_8_ (2.2 equiv.) in a less coordinating solvent (diethyl ether) under N_2_. The reaction was carried out at −40 °C for 3 days and resulted in the formation of an insoluble purple species containing N_2_-bound uranium species that was stable under vacuum. Dissolution of the purple species in THF or in benzene resulted in N_2_ release and formation of complex 2 (see ESI, Fig. S27†) and the inverse sandwich complex [{U(*κ*^5^-{(^*t*^Bu_2_ArO)_2_Me_2_-cyclam})}_2_(*μ-η*^6^:*η*^6^-benzene)], 3 respectively (see infra and ESI, Fig. S26†).

We were not able to isolate any N_2_-bound uranium complex, but we found that quenching the reaction mixture, resulting from the reduction reaction of A with 2.2 equiv. of KC_8_ in diethyl ether −40 °C under N_2_, with a strong acid (HCl in diethyl ether) revealed (upon quantitative integration performed with dimethyl sulfone as the internal standard) the formation of ammonium chloride in 20% yield (0.2 equiv. per complex A used) (see ESI†). When larger excess of KC8 was used (6–10 equiv.) ammonium chloride was obtained after quenching in similar yield (20–30%). Performing the reaction under ^15^N_2_ also yielded ammonium chloride (^15^NH_4_Cl) in 30% yield. In contrast, no ammonium chloride formation was observed when the same reaction was performed under argon or when the reduction was carried out in hexane under dinitrogen. Moreover, the ^1^H NMR spectrum measured in *d*_8_-toluene at −40 °C of the reaction mixture obtained after reaction of A with 2.2 equiv. of KC_8_ in diethyl ether −40 °C under argon showed the resonances assigned to 2 (Fig. S24b†).

**Fig. 4:**
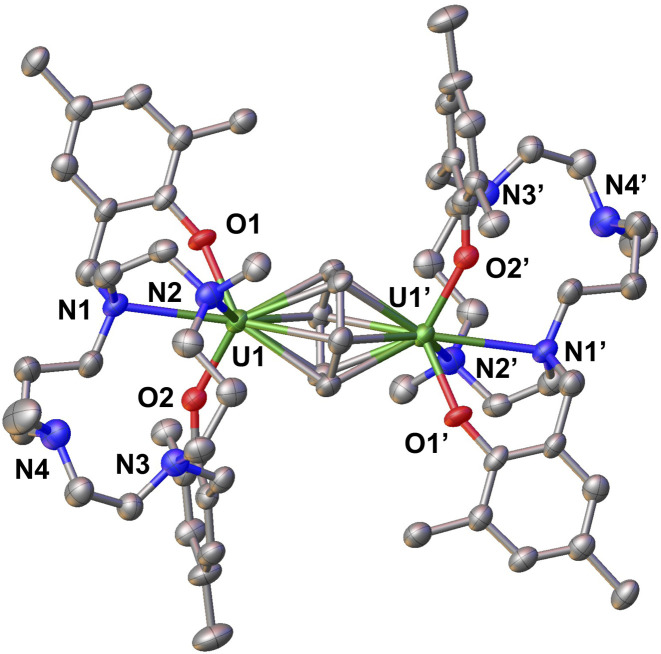
Molecular structure of complex 3 with thermal ellipsoids drawn at the 50% probability level. Methyl groups of ^*t*^Bu group and hydrogen atoms have been omitted for clarity.

Finally, the reduction of the complex A in benzene prevented the C–N oxidative addition from occurring leading instead to the isolation of the inverse sandwich complex [{U(*κ*^5^-{(^*t*Bu2^ArO)_2_Me_2_-cyclam})}_2_(*μ-η*^6^:*η*^6^-benzene)] (3) in 32% yield, formed from the reduction of the benzene molecule by the putative U(ii) intermediate. Analogous uranium inverse sandwich complexes have been reported by several groups^34,36,37,59–63^ and are considered to be U(ii) synthons because they can function as four electron reductants with diverse substrates; spectroscopic and structural studies led to the assignment of their electronic structure as consistent of U(iii) centres and a dianionic bridging arene. Single crystals of 3 suitable for X-ray diffraction were isolated from a concentrated reaction mixture in toluene at −40 °C (see Fig. 4). The reduction of A was also performed in hexane (both at −40 °C and r.t.) to isolate the putative U(ii). Reduction of A in hexane with 2.2 equiv. KC_8_ at r.t. over three days resulted in a dark red brown suspension. The ^1^H NMR spectrum (Fig. S23†) of the crude reaction mixture in *d*_6_-benzene at r.t showed the presence of complex 3 as the only identifiable species suggesting that the low solubility of the putative U(ii) complex in hexane prevents the reductive cleavage of the ligand. Dissolution of the residue in a more polar solvent such as *d*_8_-THF resulted in the observation of the signals assigned to complex 2 by ^1^H NMR spectroscopy (see ESI, Fig. S16c†), signifying that the intramolecular C–N cleavage step is instantaneous in *d*_8-_THF.

The solid-state molecular structure of 3 (see Fig. 4) reveals that each uranium metal centre is coordinated by two aryloxide oxygen atoms and two nitrogen atoms of the cyclam ring, with a benzene molecule bridging the two uranium centres. In each half of the complex, only two out of the four nitrogen atoms in the cyclam ring interact with the uranium ion in order to accommodate the benzene moiety: thereby exhibiting a conformationally flexible cyclam backbone. The U–O_aryloxide_ bond lengths (2.207(3) Å and 2.229(4) Å) are comparable to those found in complex A (2.223(4) Å and 2.263(3) Å). The average U–C_arene_ (2.61(2) Å) and C_arene_–C_arene_ (1.430(14) Å) bond lengths are consistent with the previously reported diuranium(iii) inverse sandwich complexes.^34,36,37,63^

### EPR and magnetism studies

**Fig. 5:**
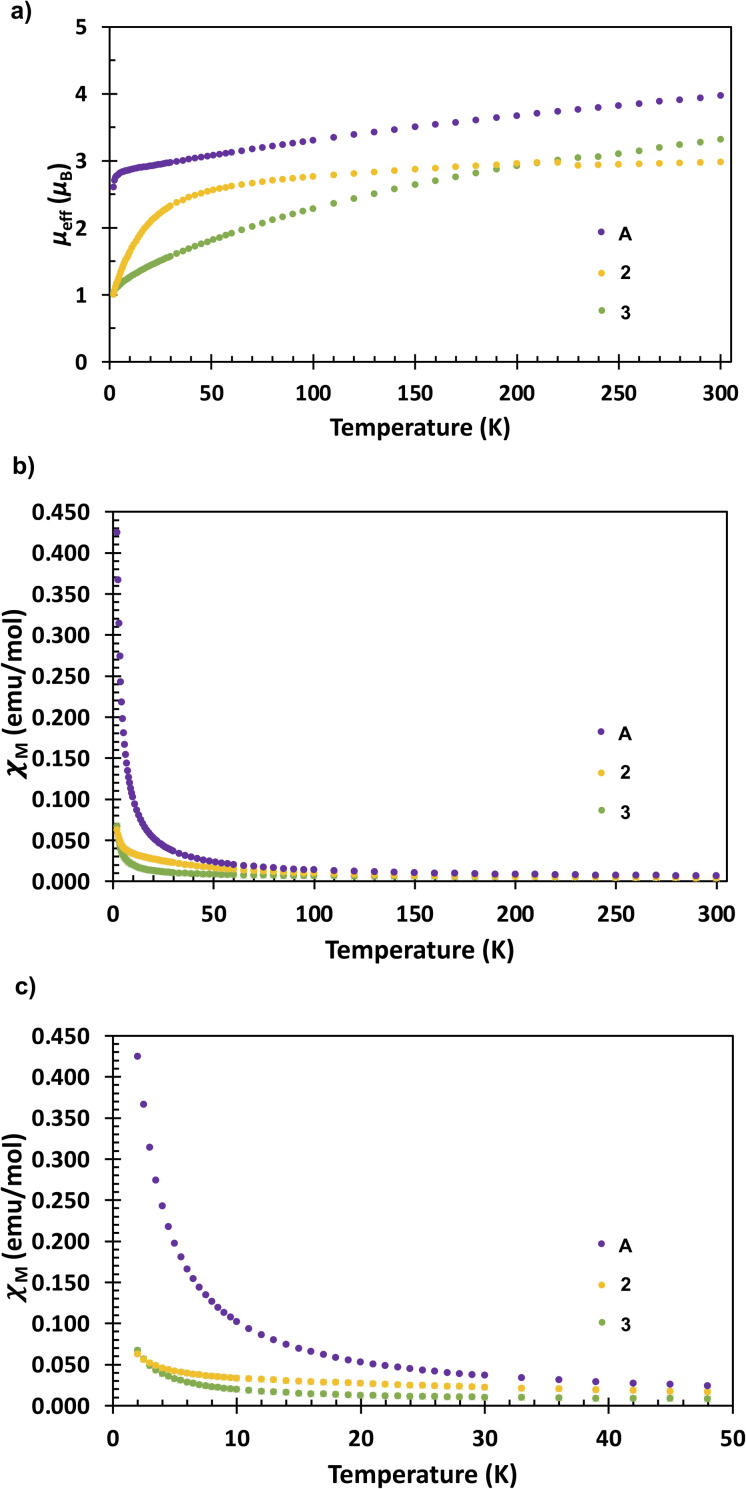
Temperature dependent SQUID magnetisation data (per U ion) for complexes A, 2 and 3 plotted as functions of (a) *μ*_eff_*vs.* temperature, (b) *χ*_M_*vs.* temperature (where *χ*_M_ is the molar magnetic susceptibility) and (c) *χ*_M_*vs.* temperature (shown up to 50 K only for clarity), measured at 1 T.

**Fig. 6:**
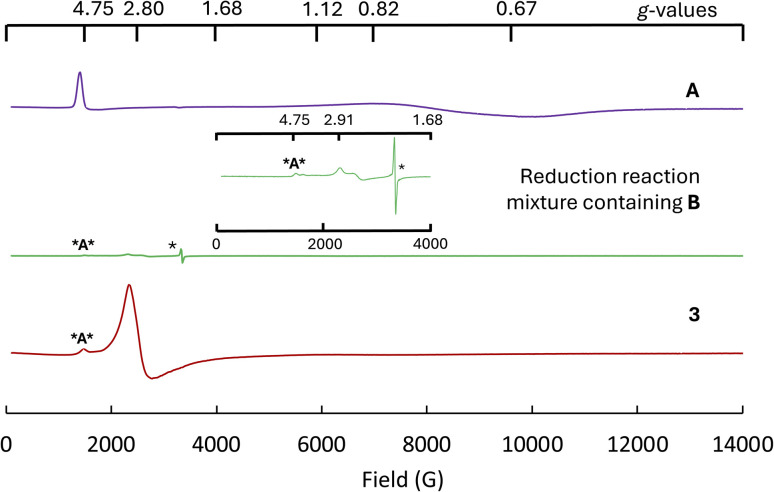
Solid state X-band (9.40 GHz) EPR spectrum of: (top) A (8.0 mg), (middle) (7.2 mg) the precipitate obtained from A + 2.2 KC_8_ reaction in hexane (which contains the intermediate species B) and (bottom) 3 (8.0 mg) at 6 K (* corresponds to a signal arising from the itinerant electrons in the KC_8_). For clarity, the inset depicts the low-field portion (0–4000 G) of the middle spectrum, which encompasses resonance features with high *g*-factor values. The signal *A* at *g* = ∼4.75 in the low-field sections of the EPR spectra corresponds todenote the residual content of the complex A.

To further elucidate the electronic structure of complex A, 2 and 3, SQUID magnetometry (Fig. 5) and EPR (Fig. 6) studies were undertaken. Complex A has a magnetic moment of 3.97*μ*_B_ (*χ*_M_ = 6.58 × 10^−3^ emu mol^−1^; *χ*T = 1.97 emu K mol^−1^) at 300 K and 2.61*μ*_B_ (*χ*_M_ = 4.25 × 10^−1^ emu mol^−1^; *χ*T = 8.50 × 10^−1^ emu K mol^−1^) at 2 K. The observed behaviour is similar to that reported for other U(iii) compounds with an f^3^ electronic configuration corresponding to ^4^I_9/2_ ground state^40,64^ In such U(iii) complexes the magnetic moment decreases steadily with decrease in temperature to reach a non-zero value corresponding to a doublet state for the Kramers ion. On the other hand, complex 2 has a magnetic moment of 2.98*μ*_B_ (*χ*_M_ = 3.70 × 10^−3^ emu mol^−1^; *χ*T = 1.11 emu K mol^−1^) at 300 K and 1.00*μ*_B_ (*χ*_M_ = 6.27 × 10^−2^ emu mol^−1^; *χ*T = 1.25 × 10^−1^ emu K mol^−1^) at 2 K. The decrease in magnetic moment is more abrupt for complex 2 below 50 K, when compared to complex A. Such a decrease in magnetic moment below 50 K is well documented for various U(iv) complexes reported in the literature.^64^ The magnetic moment for 2 does not approach zero at 2 K, which could indicate the presence of a non-singlet ground state.^65^ Nonetheless, the magnetic data for 2 compare well with those of several U(iv) complexes reported previously.^66–69^ In comparison, the measured magnetic moment of 3 is of 3.32*μ*_B_ (*χ*_M_ = 4.58 × 10^−3^ emu mol^−1^; *χ*T = 1.37 emu K mol^−1^) at 300 K and 1.04*μ*_B_ (*χ*_M_ = 6.70 × 10^−2^ emu mol^−1^; *χ*T = 1.34 × 10^−1^ emu K mol^−1^) at 2 K per uranium ion, while per complex it is 4.69*μ*_B_ (*χ*_M_ = 9.17 × 10^−3^ emu mol^−1^; *χ*T = 2.75 emu K mol^−1^) at 300 K and 1.46*μ*_B_ (*χ*_M_ = 1.34 × 10^−1^ emu mol^−1^; *χ*T = 2.68 × 10^−1^ emu K mol^−1^) at 2 K. Although the decrease in magnetic moment with the decrease in temperature in 3 is quite different from both complexes A and 2, the low value of its magnetic moment at 2 K is close to that of the U(iv) complex 2. This correlates well with DFT studies (*vide infra*) with complex 3 calculated to have a U(iv)–arene^4−^–U(iv) ground state but with a very close in energy U(iii)–arene^2−^–U(iii) state. Considering that an EPR signal is also observed (see next section) that can be assigned to the U(iii) species is likely that both configurations contribute to the magnetic response. The observed behaviour is rather different from that reported for the analogous arene-bridged complex [(*μ*-toluene)U_2_(N[^*t*^Bu]Ar)_4_] (Ar = 3,5-C_6_H_3_Me_2_) that presented a strong antiferromagnetic behaviour below 125 K and lower magnetic moment in all 5–300 K temperature range (1.57*μ*_B_ at 300 K and 0.25*μ*_B_ at 5 K for one uranium center).^36,61^

The EPR spectrum of complex A at 6 K features *g*-values of 4.75, 0.82 and 0.65 at 6 K (Fig. 6). EPR spectra with rhombic signals and similar low field *g*-factors have been reported for heteroleptic uranium(iii) complexes supported by tris(3,5- dimethyl-1-pyrazolyl)borate ligands or amide ligands.^70,71^

While assignment of oxidation state is not trivial in inverse-sandwich complexes, EPR data measured in the solid state (Fig. 6) and in frozen solution (see ESI†) at 6 K show for complex 3 the presence of a signal in agreement with the presence of a f^3^ U(iii) rather that EPR silent f^2^ U(iv) or f^4^ U(ii) ions. The EPR spectrum of 3 at 6 K features *g*-values of 2.80, 2.60 and 0.8 (Fig. 6), which following spin quantification accounts for 40% of the total expected spins. Similar EPR spectra have been reported for monometallic uranium(iii) complexes featuring strong donor ligands.^8,72,73^ The observation of this EPR profile is suggestive of a U(iii)–arene^2−^U(iii) structure for complex 3 similar to the structure proposed for the inverse sandwich complex [(*μ*-toluene)U_2_(N[^*t*^Bu]Ar)_4_] complex.^61^ The presence of such species is corroborated by the computational studies indicating the possible existence of U(iii)–arene^2−^U(iii) and U(iv)–arene^4−^U(iv) structures in equilibrium. Moreover, the EPR spectrum of the putative U(ii) species obtained as highly insoluble solid upon reacting A with 2.2 KC_8_ in hexane was also recorded to further characterize the nature of this intermediate. The EPR spectrum shows a radical-like signal due to the presence of unreacted KC_8_ at *g* = 2.0021 and two additional signals of very low intensity at *g* = 2.82 and 2.52. The signals are significantly shifted with respect to the spectrum of complex A (a small residual signal corresponding to A is also observed (Fig. S40†)). The observed low intensity signals (4% integrated spins) could be tentatively assigned to the U(ii) species generated upon reduction. The possibility that the signal could be arising from metallic impurity contained in KC_8_ (ref. 74) was ruled out by measuring the solid state EPR of the same batch of KC_8_ which revealed only an asymmetric signal at *g* = 2.0020, thus pointing to the itinerant electrons in the highly conducting particles of KC_8_. Several U(ii) complexes were reported to possess a 5f^4^ configuration^1,7,8,11^ that did not feature any metal based EPR signal. However, recently Evans and co-workers reported EPR studies on 10 U(ii) complexes having a 5f^3^6d^1^ electronic configuration that showed display two-line axial signals with *g*_‖_ = 2.04 and *g*_⊥_ = 2.00.^75^ The reported studies suggested that the presence of EPR signals can be used to differentiate 5f^3^6d^1^ and 5f^4^ configurations in U(ii) complexes.

The almost silent EPR spectrum of the product of the reduction of A in hexane indicate that a U(ii) species is formed which is mostly in a 5f^4^ configuration. The observed low intensity signals could suggest some contribution of the 5f^3^6d^1^ configuration, because of the f–d mixing also found in the computed structure (see below). To further corroborate our hypothesis the precipitate obtained from the reduction reaction mixture was suspended in benzene and the EPR spectrum of the resulting dark red brown solution (following filtration to remove excess KC_8_ and graphite) was measured at 6 K. The signal corresponding to the U(ii) intermediate disappeared, and the signal corresponding to complex 3 was observed in the EPR spectrum recorded (Fig. S27†). These results provide further evidence of the involvement of a U(ii) species in both reduction of benzene observed in 3 and the N–C cleavage observed in the case of 2.

### Computational studies

**Fig. 7:**
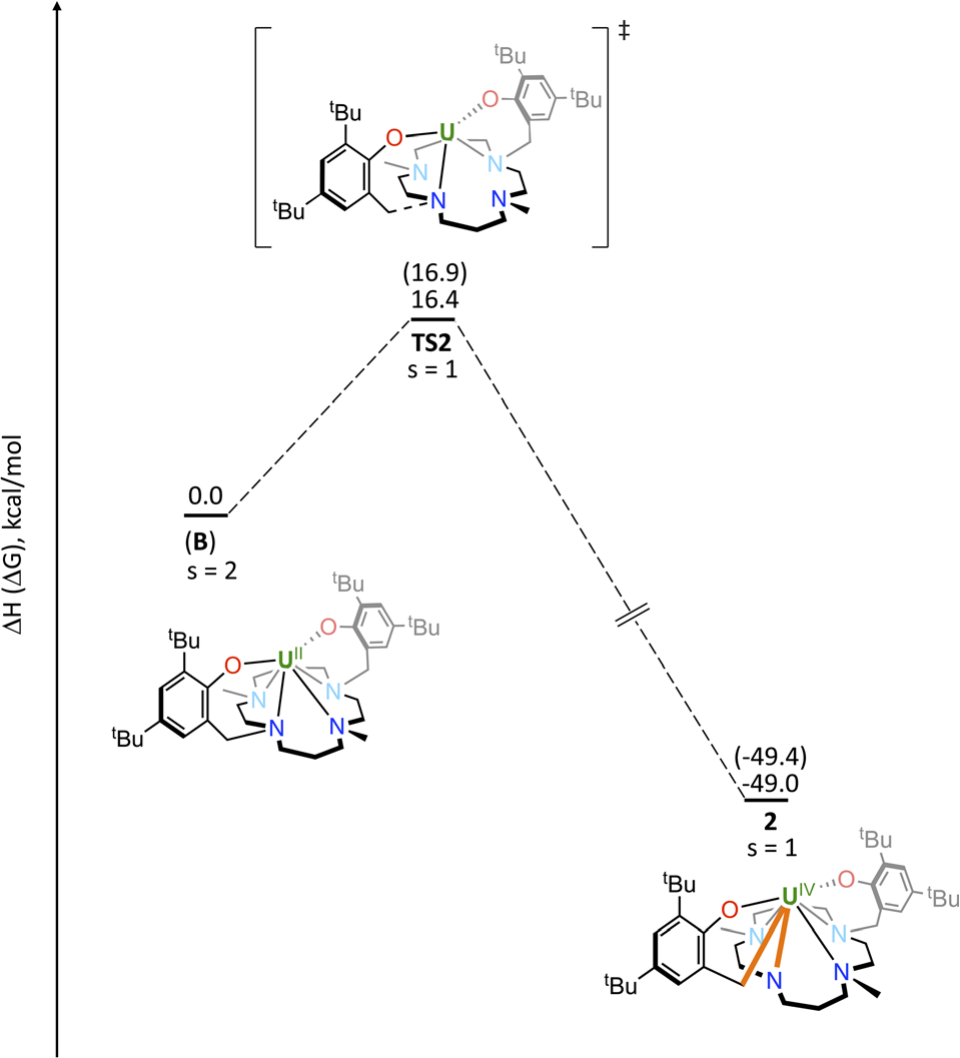
Computed enthalpy profile (Gibbs free energy between bracket) at room temperature for the formation of complex 2 from intermediate B. The energies are given in kcal mol^−1^. *S* stands for the total spin state of each intermediate.

DFT studies were carried out (B3PW91 functional) to give some insights on the formation of complex 2 and the electronic structure of complex 3. First of all the formation of the U(ii) intermediate B was investigated computationally. Although disproportionation of the starting complex A is prohibitively high (68.0 kcal mol^−1^ in enthalpy and 68.9 kcal mol^−1^ in Gibbs Free Energy), the reduction of complex A to form the transient complex B is endothermic by 23.4 kcal mol^−1^ in enthalpy (19.5 kcal mol^−1^ in Gibbs Free Energy). In THF solution, this reaction is becoming endothermic by 15.2 kcal mol^−1^ in enthalpy (11.1 kcal mol^−1^ in Gibbs Free energy). This makes this intermediate plausible and it is in line with the fact that this transient species was not experimentally stabilised. The electronic structure of B clearly indicates a U(ii) system in a *S* = 2 ground state. The associated electronic configuration is a mixture of the 5f^4^ and 5f^3^d^1^ configurations since three pure 5f orbitals are occupied and the fourth one is a mixture (60–40) between the 5f_0_ and 6d_0_ atomic orbitals. This electronic structure fits the EPR data and the hypothesis given experimentally. The formation of complex 2 from complex B was thus investigated (Fig. 7). In complex B, the coordination of both nitrogen and oxygen induces a slight elongation of the C–N bond (1.50 Å) with respect to simple amines (1.47 Å). Complex B can easily reach a C–N bond breaking transition state (TS2) with an accessible barrier of 16.4 kcal mol^−1^ in enthalpy (16.9 kcal mol^−1^ in Gibbs Free Energy). At the transition state, the C–N bond is already broken (1.88 Å) while the U–N distance is decreased to 2.55 Å. Following the intrinsic reaction coordinate, it yields the very stable complex 2 (−49.0 kcal mol^−1^ in enthalpy, −49.4 kcal mol^−1^ in Gibbs Free Energy).

The electronic structure of complex 3 was finally investigated computationally using the same methodology. Two spin states were considered, namely a *S* = 3 (two U(iii) centres) and *S* = 2 (two U(iv) centres) spin states. The *S* = 2 (quintet) is slightly more stable than the *S* = 3 (septet) by 4.8 kcal mol^−1^ in enthalpy (2.5 kcal mol^−1^ in Gibbs Free Energy). This TS is located on the triplet Potential Energy Surface (PES) and corresponds to a U(iv). This small energy difference indicates that these two structures could be in equilibrium and that clearly both can be populated at room temperature, in agreement with the observed experimental magnetism. The comparison between the optimized geometry in both spin states and the experimental one clearly shows that the quintet geometry is the one that is in best agreement with the X-ray (see Table S4†). In both cases, the unpaired spin density plots (Fig. S47k and S48k†) are located at the two uranium centres only, indicating either two U(iv) for the quintet or two U(iii) for the septet. The main difference between the two spin states is the presence of two doubly occupied δ-bonds (HOMO and HOMO − 1) for the quintet while only one is occupied (HOMO − 1) for the septet (Fig. S47 and S48†). The situation is different for [(*μ*-toluene)U_2_(N[Ad]Ar)_4_] (Ar = 3,5-C_6_H_3_Me_2_), since the quintet is found to be the ground state while the septet is 15.3 kcal mol^−1^ higher in enthalpy (16.9 kcal mol^−1^ in Gibbs Free Energy). Therefore, there is no equilibrium between the two spin states, explaining the difference in magnetism observed with complex 3.

## Conclusion

In conclusion reduction of the heteroleptic aminephenolate complex A in apolar solvents produces a highly reactive transient neutral “U(ii)” species (B). In contrast, upon replacement of the iodide ligand in A with a siloxide to yield complex 1, the reduction of the uranium centre is not observed even in presence of excess reducing agent with only minor decomposition products formed.

In polar solvents B undergoes a unique example of C–N oxidative addition to a U(ii) center to yield complex 2. The transient complex B was also found to reduce benzene to yield the inverse sandwich complex 3. Computational, EPR and magnetic studies indicate the presence for 3 of an equilibrium between two possible electronic structures very close in energy (U(iv)–arene^4−^–U(iv) and U(iii)–arene^2−^–U(iii)). These results indicate that polydentate amine-phenolate ligands can be used to access highly reactive U(ii) intermediates and that U(ii) species are involved in the formation of inverse sandwich complexes. Moreover the “U(ii)” intermediate was found to react with N_2_ yielding ammonia (even if in low yield) upon acidification. Future studies will be directed to tune the stability and the reactivity of the U(ii) intermediate by ligand design.

## Author contributions

R. A. K. S designed and carried out all the experiments and analysed the data; L. M. carried out the ligand synthesis and some of the experiments and analysed the data. M. M. designed and supervised the project and analysed the data; T. R. and L. M. carried out the computational study; R. S. measured and analyzed the X-ray data, I. Z. measured and analysed the magnetic data; A. S. measured and analysed the EPR data; R. A. K. S and M. M. wrote the manuscript with contributions of all authors, and all authors have given approval for the final version of the manuscript.

## Conflicts of interest

There are no conflicts to declare.

## Supplementary Material

SC-OLF-D5SC03694A-s001

SC-OLF-D5SC03694A-s002

## Data Availability

Synthetic details, analytical data including depictions of all spectra and coordinate data of all computationally optimised species, are documented in the ESI.† Crystallographic data is made available *via* the CCDC. The data that support the findings of this study are openly available in the zenodo repository at https://doi.org/10.5281/zenodo.15801184.†
