# Supplementary material for: Facile N–C bond cleavage and arene reduction by a transient uranium(ii) complex
Source: Chem Sci. 2025 Jul 2;16(31):14242–51. doi: 10.1039/d5sc03694a (PMC12236195; doi:10.1039/d5sc03694a)
Supplement: SC-016-D5SC03694A-s001 [file SC-016-D5SC03694A-s001.pdf]

Supplementary Information (SI) for Chemical Science.
This journal is © The Royal Society of Chemistry 2025

Electronic Supporting Information

Facile N-C Bond Cleavage and Arene Reduction by a Transient Uranium (II) Complex

R. A. Keerthi Shivaraam,[a] Leonor Maria,[b] Thayalan Rajeshkumar,[c] Rosario Scopelliti,[d] Ivica Živković,[e] Dr. Andrzej Sienkiewicz,[e,f] Laurent Maron[c] and Marinella Mazzanti.*[a]

[a] Institut des Sciences et Ingénierie Chimiques, École Polytechnique Fédérale de Lausanne (EPFL), CH-1015 Lausanne, Switzerland.
[b] Centro de Química Estrutural, Institute of Molecular Sciences, Instituto Superior Técnico, Universidade de Lisboa, 2695-066 Bobadela, Portugal
[c] Laboratoire de Physique et Chimie des Nano-objets, Institut National des Sciences Appliquées, 31077 Toulouse, Cedex 4 (France).
[d] X-Ray Diffraction and Surface Analytics Platform, Institut des Sciences et Ingénierie Chimiques, École Polytechnique Fédérale de Lausanne (EPFL), CH-1015 Lausanne, Switzerland.
[e] Laboratory for Quantum Magnetism, Institute of Physics, Ecole Polytechnique Fédérale de Lausanne (EPFL), 1015 Lausanne (Switzerland).
[f] ADSresonances SàrL, 1920 Martigny (Switzerland)

*Email to whom correspondence should be addressed: marinella.mazzanti@epfl.ch

Table of Contents

A. Experimental Procedures

B. NMR Spectroscopic Data

C. X-Ray Crystal Structure Determination Details

D. EPR Spectroscopic Data

E. SQUID Magnetometry Data

F. Cyclic Voltammetry Data

G. UV/Vis/NIR Spectra

H. Computational Details

I. References

A. Experimental Procedures

General Considerations. Unless otherwise noted, all manipulations were carried out at ambient temperature under an argon atmosphere using Schlenk techniques and an MBraun glovebox equipped with a purifier unit. The water and oxygen levels were always kept at less than 0.1 ppm. Glassware was dried overnight at 140 °C before use. Syntheses were performed using glass coated stirrer bars. Volatiles transfer experiments to release and trap ammonia were performed using a two-bulb apparatus J. Young sealable distillation apparatus.

NMR experiments were carried out using NMR tubes adapted with J-Young valves. NMR spectra were recorded on a Bruker 400 MHz or 600 MHz spectrometers. NMR chemical shifts are reported in ppm with solvent as internal reference Elemental analyses were performed under nitrogen using a Thermo Scientific Flash 2000 Organic Elemental Analyzer at the Institute of Chemistry and Chemical Engineering at EPFL.

EPR analyses were performed on a Bruker Elexsys E500 spectrometer working at 9.4 GHz frequency with an Oxford ESR900 cryostat for 4-300 K operation. Spin quantification was performed using $CuSO_4 \times 5H_2O$ (Cu^{2+}, ***S*** =1/2, 5.36 mg, number of Cu^{2+} paramagnetic centers in this standard sample is 1.29 x 10^{19}) as a standard.

Magnetic measurements were performed using a Quantum Design MPMS3 superconducting quantum interference device (SQUID) magnetometer in a temperature range 2-300 K. The sample was restrained with eicosane (for complex **A**) or glass wool (for complexes **2** and **3**) and enclosed in a quartz capsule packed under argon and placed inside a plastic straw. The measurements were performed with applied magnetic field of 1 T in the zero-field cooled (ZFC) regime. Diamagnetic corrections were applied using Pascal's constants.[1] The magnetic moment per complex was calculated using the following formula:

$$\mu_{eff} = \sqrt{8\chi_{corr}T}$$

where $\chi_{corr} = \chi_{measured} - \chi_{dia}$, χ_{dia} was calculated using Pascal's constants.[1]

UV/Vis/NIR spectra were recorded as solutions using 1 mm cuvettes equipped with a J-Young valve and a Perkin Elmer 950 spectrometer.

Electrochemical Methods. Cyclic voltammetry data were carried out at room temperature in an argon-filled glovebox described above. Data were collected using a Biologic SP-300 potentiostat connected to a personal computer. All samples were saturated in complex with 0.1 M $[Bu_4N][BPh_4]$ supporting electrolyte in THF solution. The experiments were carried out with a platinum disk (d = 5 mm) working electrode, a platinum wire counter electrode, and an Ag/AgCl reference electrode. Potential calibration was performed at the end of each data collection cycle using the ferrocene/ferrocenium $[(C_5H_5)_2Fe]^{+/0}$ couple as an internal standard.

Starting materials. Unless otherwise noted, reagents were purchased from commercial suppliers and used without further purification. Anhydrous solvents were purchased from Aldrich and further distilled from K/benzophenone (THF, diethyl ether), sodium sand/benzophenone (*n*-hexane). Deuterated solvents for NMR spectroscopy (d_8-THF) were purchased from Cortecnet, freeze-degassed and distilled over K/benzophenone. d_6-DMSO were freeze-degassed and dried over 3 Å molecular sieves for several days. KC_8, $[U(\kappa^6\text{-}\{(^{tBu2}ArO)_2Me_2\text{-cyclam}\})I]$ (**A**), $K_2\{(^{tBu2}PhO)_2Me_2$-cyclam$\}(THF)_2$ and $KOSi(O^tBu)_3$ were synthesized according to literature procedures.[2–5]

Synthesis of [U(κ^6-{(^{tBu2}ArO)$_2$Me$_2$-cyclam})(OSi(O^{t}Bu)$_3$)] (1)

A colorless solution of KOSi(O^{t}Bu)$_3$ (15.5 mg, 0.05 mmol, 1.0 equiv.) in THF (1 mL) at -40 °C was added to a violet suspension of [U(κ^6-{(^{tBu2}ArO)$_2$Me$_2$-cyclam})I] (**A**) (52.5 mg, 0.05 mmol, 1.0 equiv.) in THF (1.5 mL) at -40 °C. The reaction mixture darkened during the addition of the reagents, following which it was stirred at -40 °C overnight (20 h). The reaction mixture supernatant was dark red brown in colour and a white precipitate was observed. The volatiles were removed under vacuum and the resulting residue was triturated with diethyl ether (0.5 mL) and dried under vacuum for 15 mins. This procedure was repeated two times more. The resulting residue was suspended in hexane (1.5 mL) and stirred at r.t for two minutes, following which the reaction mixture was filtered over a porosity 4 filter frit to obtain a dark red brown solution, which was placed at -40 °C for one day. The maroon solid formed was collected over a cold (-40 °C) porosity 4 filter frit and dried under vacuum, affording **1** as a maroon solid (33.4 mg, 0.03 mmol, 56%). Dark red brown crystals of **1.(hexane)$_{0.5}$** suitable for X-Ray diffraction were obtained from slow evaporation of a concentrated reaction mixture in pentane at -40 °C. ^{1}H NMR (400 MHz, d_8-THF, 233 K) δ 42.6 (s, 1H), 38.95 (s, 1H), 35.79 (s, 1H), 35.48 (s, 1H), 30.55 (s, 1H), 30.02 (s, 1H), 28.16 (s, 1H), 24.74 (s, 1H), 16.45 (s, 1H), 13.55 (s, 1H), 11.36 (s, 1H), 9.72 (s, 1H), 9.20 (s, 1H), 7.90 (s, 1H), 6.77 (s, 27H, O^{t}Bu), 3.08 (s, 9H, Ar-^{t}Bu), 0.76 (s,1H), 0.58 (s, 9H, Ar-^{t}Bu), -2.0 (s, 3H, NCH$_3$), -4.65 (s, 1H), -5.74 (s, 1H), -6.41 (s, 1H), -8.71 (s, 9H, Ar-^{t}Bu), -11.07 (s, 1H), -11.62 (s, 1H), -15.46 (s, 1H), -22.12 (s9H, Ar-^{t}Bu), -25.18 (s, 1H), -27.17 (s, 3H, NCH$_3$), -27.8 (s, 1H), -30.45 (s, 1H), -32.78 (s, 1H), -40.06 (s, 1H), -48.84 (s, 1H), -49.30 (s, 1H) ppm (**Figure S4**). Elemental analysis calcd (%) for [U(κ^6-{(^{tBu2}ArO)$_2$Me$_2$-cyclam})(OSi(O^{t}Bu)$_3$)] (**1**) (1164.51 g mol^{-1}): $C_{54}H_{97}N_4O_6Si_1U_1$: C 55.70, H 8.33, N 4.81; found: C 55.26, H 8.36, N 4.63. Complex **1** is stable in solution (d_8-THF) for at least 7 days (**Figure S5**) at -40 °C and continues to persist after 15 days (**Figure S6**) at r.t.

Synthesis of [U(κ^7-{(^{tBu2}ArO)(^{tBu2}ArO- κ^2-N,C)Me$_2$-cyclam})] (2)

A violet suspension of **A** (34.3 mg, 0.03 mmol, 1.0 equiv.) in THF (1.5 mL) at -40 °C was added to solid KC$_8$ (9.9 mg, 0.07 mmol, 2.2 equiv.) at -40 °C. No observable colour change was perceived immediately after addition. The reaction mixture was stirred at -40 °C for one day resulting in a dark brown yellow supernatant and a black precipitate (i.e. graphite) along with excess KC$_8$. The reaction mixture was filtered over a cold (-40 °C) porosity 4 glass filter frit to obtain a dark brown yellow solution. The volatiles were evaporated under reduced pressure and the reaction mixture was dissolved in cold (-40 °C) hexane (0.2 mL) to obtain a dark brown yellow solution, which was placed at -40 °C for four days. The dark green yellow solid formed was collected over a cold (-40 °C) porosity 4 filter frit and dried under vacuum, affording **2** as a dark green yellow solid (13.4 mg, 0.02 mmol, 45 %). Yellow crystals of **2.(THF)$_2$** suitable for X-Ray diffraction were obtained from a concentrated reaction mixture in THF at -40 °C. ^{1}H NMR (400 MHz, d_8-THF, 233 K) δ 229.50 (s, 1H, $^-$NCH$_2$-amide), 223.68 (s, 1H, $^-$NCH$_2$-amide), 221.78 (s, 1H, $^-$NCH$_2$-amide), 216.67 (s, 1H, $^-$NCH$_2$-amide), 52.99 (s, 1H), 51.35 (s, 9H, ^{t}Bu), 39.31 (s, 1H), 36.83 (s, 1H), 31.24 (s, 1H), 17.21 (s, 1H), 15.26 (s, 9H, ^{t}Bu), 8.81 (s, 1H), 7.09 (s, 1H), -2.91 (s, 1H), -7.06 (s, 9H, ^{t}Bu), -8.52 (s, 1H), -10.31 (s, 1H), -16.50 (s, 1H), -22.08 (s, 9H, ^{t}Bu), -22.61 (s, 1H), -33.10 (s, 1H), -33.34 (s, 1H), -41.31 (s, 1H), -44.93 (s, 1H), -45.20 (s, 1H), -60.94 (s, 1H), -64.80 (s, 2H), -73.58 (s, 1H), -81.18 (s, 1H), -84.72 (s, 3H, NCH$_3$), -94.25 (s, 1H), -97.56 (s, 1H), -112.47 (s, 3H, NCH$_3$), -137.21 (s, 1H), -272.05 (s, 1H) ppm (**Figure S13**). Elemental analysis calcd (%) for [U(κ^7-{(^{tBu2}ArO)(^{tBu2}ArO- κ^2-N,C)Me$_2$-cyclam})].(THF)$_{0.5}$ (**2.(THF)$_{0.5}$**) (937.13 g mol^{-1}): $C_{44}H_{74}N_4O_{2.5}U_1$: C 56.39, H 7.90, N 5.98; found: C 56.19, H 8.12, N 5.56. Complex **2** is stable in solution (d_8-THF) for at least 13 days (**Figure S14**) at -40 °C whilst significant decomposition could be observed after seven days at r.t. (**Figure S15**). *NOTE*: When the reduction of **A** was carried out in THF in the presence of 2.2.2-cryptand, complex **2** was still observed as the major species, when all the other parameters where remained constant. Complex **2** was still obtained as the major species when the analogous reaction was performed under dinitrogen.

Synthesis of [{U(κ^5-{(^{tBu2}ArO)$_2$Me$_2$-cyclam})}$_2$(μ-η^6:η^6-benzene)] (3)

A violet suspension of [U(κ^6-{(^{tBu2}ArO)$_2$Me$_2$-cyclam})I] (**A**) (129.8 mg, 0.1 mmol, 1.0 equiv.) in benzene (10.0 mL) at r.t. was added to solid KC$_8$ (34.1 mg, 0.3 mmol, 2.0 equiv.) at r.t. No observable colour change was perceived immediately after addition. The reaction mixture was stirred at r.t. for one day resulting in a dark brown supernatant and a black precipitate (i.e. graphite) along with excess KC$_8$. The reaction mixture was filtered over a porosity 4 glass filter frit to obtain a dark brown solution. The volatiles were evaporated under reduced pressure and the reaction mixture was dissolved in toluene (1.5 mL) and hexane (3.0 mL) to obtain a dark brown solution, which was placed at -40 °C for two days. The dark brown solid formed was filtered over a cold (-40 °C) porosity 4 filter frit, affording **3** as a dark brown solid (19.7 mg, 0.01 mmol). A second crop (18.7 mg, 0.01 mmol) could be obtained from a toluene/hexane (0.1 mL/0.6 mL) solution of the mother liquor at -40 °C. The combined yield of the crops is 32%. Brown crystals of **3.(toluene)$_{3.6}$** suitable for X-Ray diffraction were obtained from a concentrated reaction mixture in toluene at -40 °C. ^{1}H NMR (400 MHz, d_6-benzene, 298 K) δ 28.44 (s), 24.49 (s), 23.74 (s), 19.0 (s), 17.34 (s), 15.62 (s), 15.18 (s), 13.41 (s), 13.13 (s), 12.77 (s), 12.10 (s), 11.90 (s), 11.38 (s), 11.05 (s), 10.47 (s), 10.19 (s), 9.35 (s), 8.50 (s), 6.63 (s), 5.65 (s), 4.07 (s), 3.70 (s), 2.96 (s), 2.48 (s), 2.00 (s), 0.12 (s), -1.39 (s), -3.34 (s), -4.98 (s), -5.31 (s), -5.79 (s), -7.57 (s), -9.15 (s), -10.90 (m), -12.2 (s), -14.4 (m), -17.0 (s), -18.66 (s), -20.43 (s), -24.70 (s), -31.18 (s), -84.18 (s) ppm (**Figure S20**). Elemental analysis calcd (%) for [{U(κ^5-{(^{tBu2}ArO)$_2$Me$_2$-cyclam})}$_2$(μ-η^6:η^6-benzene)] (**3.(toluene)$_{0.5}$**) (1926.34 g mol^{-1}): $C_{93.5}H_{150}N_8O_4U_2$: C 58.30, H 7.79, N 5.82;

found: C 58.46, H 8.06, N 6.02. Complex **3** has limited stability in solution (d_6-benzene) as full decomposition could be observed after two days (**Figure S21**) at r.t. *NOTE*: An insoluble dark brown solid was obtained when the analogous reduction of **A** was carried out in benzene in the presence of 2.2.2-cryptand (1.0 equiv.), thus precluding its further characterisation.

Titration of A with KC_8 (1.0 to 2.0 equiv.) in d_8-THF at -40 °C

A violet suspension of [U(κ^6-{(^{tBu2}ArO)$_2$Me$_2$-cyclam})I] (**A**) (20.0 mg, 0.02 mmol, 1.0 equiv.) in d_8-THF (0.5 mL) at -40 °C was added to solid KC_8 (2.6 mg, 0.02 mmol, 1.0 equiv.) at -40 °C. No observable colour change was perceived immediately after addition. The reaction mixture was stirred at -40 °C for overnight (14 h) resulting in a dark brown-purple supernatant and a black precipitate (i.e. graphite). Analysis of the ^{1}H NMR spectrum (**Figure S9**b)) of the crude reaction mixture at -40 °C revealed the presence of resonances corresponding to the starting material complex **A** and new resonances assigned to complex **2**. The resulting reaction mixture was added to solid KC_8 (2.6 mg, 0.02 mmol, 1.0 equiv.) and stirred at -40 °C overnight (14 h) resulting in a dark brown yellow supernatant and a black precipitate (i.e. graphite), along with excess KC_8. Analysis of the ^{1}H NMR spectrum (**Figure S9**c)) of the crude reaction mixture at -40 °C revealed only the presence of complex **2** and indicating complete consumption of complex **A**.

Reaction of 1 with KC_8 (1.0-5.0 equiv.) in d_8-THF at -40 °C

A dark red brown solution of **1** (7.5 mg, 0.006 mmol, 1.0 equiv.) in d_8-THF (0.5 mL) at -40 °C was added to solid bronze KC_8 (0.9 mg, 0.006 mmol, 1.0 equiv.) at -40 °C. No observable colour change was perceived immediately after addition. The reaction mixture was stirred at -40 °C overnight (14 h) resulting in a dark red brown supernatant and a black precipitate (i.e. graphite). Analysis of the ^{1}H NMR spectrum of the crude reaction mixture at -40 °C revealed the presence of resonances corresponding to the starting material complex **1** (**Figure S7**a)). The existing reaction mixture was added to solid bronze KC_8 (3.5 mg, 0.026 mmol, 4.0 equiv.) at -40 °C and the reaction mixture was stirred at -40 °C for 1 h, resulting in a dark red brown supernatant (in addition to excess bronze KC_8 and graphite). Analysis of the ^{1}H NMR spectrum of the crude reaction mixture at -40 °C revealed the presence of complex **1** as the major species (**Figure S7**b)).

Reaction of 1 with KC_8 in d_8-THF at -40 °C

A dark red brown solution of **1** (5.1 mg, 0.004 mmol, 1.0 equiv.) in d_8-THF (0.4 mL) at -40 °C was added to solid bronze KC_8 (1.3 mg, 0.01 mmol, 2.2 equiv.) at -40 °C. No observable colour change was perceived immediately after addition. The reaction mixture was stirred at -40 °C overnight (14 h) resulting in a dark red brown supernatant along with a black precipitate (i.e. graphite) and excess bronze KC_8. Analysis of the ^{1}H NMR spectrum of the crude reaction mixture at -40 °C revealed the presence of resonances corresponding to the starting material complex **1** (**Figure S8**).

Reaction of K_2(^{tBu2}ArO)$_2$Me$_2$-cyclam with KC_8 in d_8-THF at -40 °C

A colorless solution of K_2(^{tBu2}ArO)$_2$Me$_2$-cyclam (5.3 mg, 0.007 mmol, 1.0 equiv.) in d_8-THF (0.5 mL) at -40 °C was added to solid KC_8 (2.3 mg, 0.02 mmol, 2.2 equiv.) at -40 °C. No colour change was perceived immediately after addition. The reaction mixture was stirred at -40 °C for one day resulting in a colourless supernatant and unreacted bronze KC_8. The reaction mixture was filtered over a cold (-40 °C) porosity 4 glass filter frit to obtain a colourless solution. Analysis of the ^{1}H NMR spectrum of the colourless solution at -40 °C revealed the presence of unreacted K_2(^{tBu2}ArO)$_2$Me$_2$-cyclam starting material and no other species were observed (**Figure S29**).

Reaction of A with KC_8 in hexane at r.t. followed by analysis of the reaction mixture in d_8-THF

A violet suspension of **A** (10.3 mg, 0.01 mmol, 1.0 equiv.) in hexane (0.8 mL) at r.t. was added to solid bronze KC_8 (3.0 mg, 0.02 mmol, 2.2 equiv.) at r.t. No observable colour change was perceived immediately after addition. The reaction mixture was stirred at r.t. for three days resulting in a dark red brown precipitate (in addition to excess bronze KC_8 and graphite) and a pale brown yellow supernatant. Analysis of the ^{1}H NMR spectrum (at -40 °C) of the crude reaction mixture (after removal of volatiles under reduced pressure) in cold (-40 °C) d_8-THF (0.5 mL) revealed many resonances from which complex **2** was identified as the major species (**Figure S16**c) and **Figure S17**c)).

Reaction of A with KC_8 in hexane at r.t. followed by analysis of the reaction mixture in d_6-benzene

A violet suspension of **A** (10.0 mg, 0.01 mmol, 1.0 equiv.) in hexane (0.8 mL) at r.t. was added to solid bronze KC_8 (2.9 mg, 0.02 mmol, 2.2 equiv.) at r.t. No observable colour change was perceived immediately after addition. The reaction mixture was stirred at r.t. for three days resulting in dark red brown precipitate (in addition to excess bronze KC_8 and

graphite) and a pale brown yellow supernatant. Analysis of the ^{1}H NMR spectrum (at r.t.) of the crude reaction mixture (after removal of volatiles under reduced pressure) in d_6-benzene (0.5 mL) revealed the presence of many resonances from which complex **3** was the only identifiable species (**Figure S23**).

Reaction of A with KC_8 in hexane at r.t. followed by analysis by EPR spectroscopy

A violet suspension of **A** (11.5 mg, 0.01 mmol, 1.0 equiv.) in hexane (0.8 mL) at r.t. was added to solid bronze KC_8 (3.3 mg, 0.03 mmol, 2.2 equiv.) at r.t. No observable colour change was perceived immediately after addition. The reaction mixture was stirred at r.t. for 3.5 days resulting in a dark red brown precipitate (in addition to excess bronze KC_8 and graphite) and a pale brown yellow supernatant. The precipitate formed was filtered over a porosity 4 filter frit and washed with hexane (0.5 mL) to obtain 7.2 mg of solid (*NOTE*: the solid collected is a mixture of uranium containing species, excess KC_8 and graphite). Analysis of the EPR spectrum (**Figure S40**) of the precipitate (7.2 mg) at 6 K revealed a signal *g*-values 2.91and 2.61 probably arising from a uranium containing species, in addition to a signal at $g = 2$ corresponding to the excess KC_8 present. The precipitate was then suspended in benzene (0.2 mL) and filtered over a porosity 4 filter frit to obtain a red brown solution. Analysis of the EPR spectrum (**Figure S42**) of the frozen red brown solution in benzene at 6 K revealed an EPR spectrum corresponding to that of complex **3**.

Titration of A with KC_8 (1.0 to 2.0 equiv.) in d_6-benzene at r.t.

A violet suspension of **A** (9.5 mg, 0.009 mmol, 1.0 equiv.) in d_6-benzene (0.5 mL) at r.t. was added to solid bronze KC_8 (1.3 mg, 0.009 mmol, 1.0 equiv.) at r.t. No observable colour change was perceived immediately after addition. The reaction mixture was stirred at r.t. for one day resulting in dark brown suspension. Analysis of the ^{1}H NMR spectrum of the crude reaction mixture at r.t. revealed the presence of new resonances assigned to complex **3**, and the presence of unreacted complex **A** (**Figure S22**a)). The resulting reaction mixture was added to solid KC_8 (1.3 mg, 0.009 mmol, 1.0 equiv.) and stirred at r.t. for one day resulting in dark brown suspension. Analysis of the ^{1}H NMR spectrum of the crude reaction mixture at r.t. revealed complex **3** as the predominant species and the complete consumption of the starting material **A** (**Figure S22**b)).

Reaction of A with KC_8 in diethyl ether under dinitrogen at -40 °C followed by acid quenching

A violet suspension of **A** (10.3 mg, 0.01 mmol, 1.0 equiv.) in diethyl ether (0.8 mL) at -40 °C was added to solid bronze KC_8 (3.0 mg, 0.02 mmol, 2.2 equiv.) under dinitrogen. No observable colour change was perceived immediately after addition. The reaction mixture was stirred at -40 °C for three days resulting in dark purple precipitate (in addition to excess bronze KC_8 and graphite) and a pale brown yellow supernatant.

The volatiles were evaporated under reduced pressure and the resulting reaction mixture was cooled to -80 °C. A cold (-80 °C) colourless solution of 1 M HCl in diethyl ether (2.0 mL) was added to the reaction mixture at -80 °C resulting in the concomitant formation of a black precipitate and a colourless supernatant. The reaction mixture was brought to room temperature (r.t.) and stirred at r.t. overnight (16 h). The volatiles were evaporated under reduced pressure and the reaction mixture was dried under vacuum for 4 h. ^{1}H NMR spectrum (**Figure S30**) of the resulting sample in d_6-DMSO (0.5 mL) revealed the presence of two peaks at 7.55 ppm and 7.43 ppm which correspond to the ammonium chloride triplet arising from the acid quenching of the product of dinitrogen reduction upon the reaction of **A** with KC_8 (2.2 equiv.). The third signal corresponding to the ammonium chloride triplet is obscured by resonances corresponding to the cyclam ligand. The ammonium chloride triplet could however be observed and quantified upon performing a volatiles-transfer experiment (*vide infra*). Control experiments were performed (*vide infra*) to confirm the presence of ammonium chloride arising from dinitrogen reduction. Two control experiments were conducted wherein (1) the reduction reaction mixture conducted under argon was quenched with HCl in diethyl ether to detect the presence of the resonances at 7.55 ppm and 7.43 ppm and (2) the protonated ligand $H_2(^{tBu2}ArO)_2Me_2$-cyclam was reacted with HCl in diethyl ether to rule out the possibility of ammonium chloride formation from the nitrogenous cyclam ligand (*vide infra*). No ammonium chloride was observed in either case (**Figure S34** and **Figure S36**) thereby confirming dinitrogen reduction occurring when the reduction of **1** is carried out under dinitrogen.

Reaction of A with KC_8 in diethyl ether under argon at -40 °C

The ^{1}H NMR spectrum in toluene at -40°C (Figure **S24b**) of a crude reaction mixture resulting from **A** + 2.2 equiv. of KC_8 stirred for three days in diethyl ether at -40 °C showed resonances assigned to complex **2**.

Quantification of ammonium chloride generated from the reaction of A with KC_8 in diethyl ether under dinitrogen at -40 °C

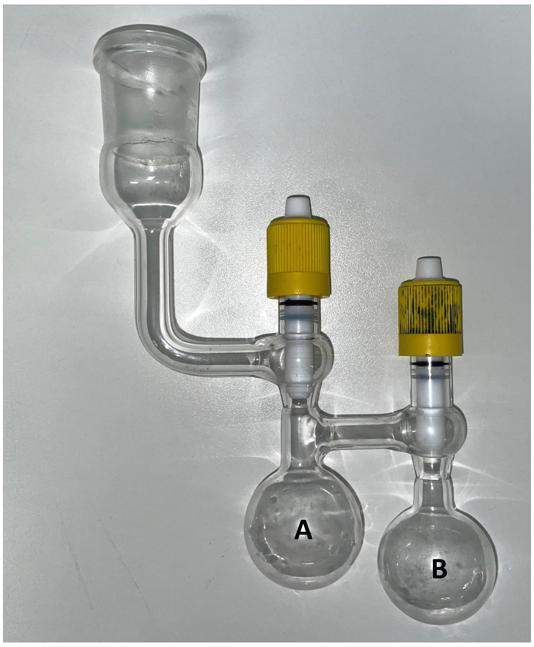

Figure S1. Picture of the double bulb distillation apparatus used for the volatiles transfer experiment.

NOTE: The distillation apparatus used contains two bulbs (namely *A* and *B*), both of which are adapted with a J. Young valve and can be sealed on their own; both of which are connected to each other by a glass tube fitting at 90 **°** with respect to each of them. The apparatus has one attachment/outlet closer to one of the bulbs (namely bulb *A*) which can be connected to the Schlenk line for Schlenk-line manipulations.

A violet suspension of **A** (12.3 mg, 0.01 mmol, 1.0 equiv.) in diethyl ether (0.8 mL) at -40 °C was added to solid bronze KC_8 (3.6 mg, 0.03 mmol, 2.2 equiv.) under dinitrogen. No observable colour change was perceived immediately after addition. The reaction mixture was stirred at -40 °C for three days resulting in dark purple precipitate (in addition to excess bronze KC_8 and graphite) and a pale brown yellow supernatant. The volatiles were evaporated under reduced pressure and the resulting reaction mixture was cooled to -80 °C. A cold (-80 °C) colourless solution of 1 M HCl in diethyl ether (2.0 mL) was added to the reaction mixture at -80 °C resulting in the concomitant formation of a black precipitate and a colourless supernatant. The reaction mixture was brought to room temperature (r.t.) and stirred at r.t. overnight (16 h). The volatiles were evaporated under reduced pressure to eliminate any unreacted HCl, and resuspended in diethyl ether (2.0 mL). Inside the dinitrogen glovebox, the J. Young valve-capped distillation apparatus was placed in a pre-chilled (-160 ºC) glovebox-fitted cold well following which 1 M HCl in diethyl ether (2.0 mL) was pipetted in bulb *B* and frozen. The apparatus was connected to the glovebox vacuum line, and placed under vacuum. After 5 minutes, the J. Young valve leading to the bulb *B* was closed. The resulting reaction mixture in diethyl ether was transferred to bulb *A* of the distillation apparatus fitted with a stirrer bar (*NOTE*: bulb *B* is still closed and under static vacuum). The J. Young valves for both the bulbs were closed, the apparatus was taken out of the glovebox, attached to a Schlenk line and bulb *A* was frozen. Under a strong flow of dinitrogen, a colourless solution of NaO^{t}Bu (11.5 mg, 0.1 mmol, 10.0 equiv.) in diethyl ether (2 mL) was added *via* a syringe, after which the bulb *A* was closed and thawed (*NOTE*: bulb *B* remains closed throughout this manipulation). The resulting black suspension was stirred at r.t. overnight (18 h). The bulb containing 1 M HCl in diethyl ether (2.0 mL) (i.e. bulb *B*) was frozen and the volatiles of bulb *A* (i.e. the reaction mixture) were vacuum-transferred to bulb *B*. Bulb *B* was thawed and the entire apparatus was entered into the dinitrogen glovebox. The volatiles in bulb *B* were evaporated under reduced pressure and the resulting white residue obtained was dried under vacuum for 30 mins. ^{1}H NMR spectrum of the white residue in d_6-DMSO (0.4 mL) revealed the presence of ammonium chloride at 7.16 ppm (**Figure S31**). A quantitative ^{1}H NMR spectrum (**Figure S32**) in d_6-DMSO (0.6 mL) with dimethylsulfone as the internal standard revealed the formation of ammonium chloride in 20% yield (0.2 equiv. of NH_4Cl per complex **A** used).

Quantification of ammonium chloride ($^{15}NH_4Cl$) generated from the reaction of A with KC_8 in diethyl ether under $^{15}N_2$ at -40 °C

A violet suspension of **A** (25.3 mg, 0.02 mmol, 1.0 equiv.) in diethyl ether (3.0 mL) was added to solid bronze KC_8 (33.3 mg, 0.25 mmol, 10.0 equiv.) under argon. No observable colour change was perceived immediately after addition. The reaction mixture was immediately brought outside the glovebox, attached to a Schlenk line, freeze/degassed (x3) and $^{15}N_2$ (820 mbar) was added whilst the reaction mixture was frozen The resulting reaction mixture was stirred at -40 °C for three days resulting in dark purple precipitate (in addition to excess bronze KC_8 and graphite) and a pale brown yellow supernatant. The volatiles were evaporated under reduced pressure and the resulting reaction mixture was cooled to -80 °C. A cold (-80 °C) colourless solution of 1 M HCl in diethyl ether (6.0 mL) was added to the reaction mixture at -80 °C resulting in the concomitant formation of a black precipitate and a colourless supernatant. The reaction mixture was brought to room temperature (r.t.) and stirred at r.t. overnight (16 h). The volatiles were evaporated under reduced pressure to eliminate any unreacted HCl, and resuspended in diethyl ether (2.0 mL). Inside the dinitrogen glovebox, the J. Young valve-capped distillation apparatus was placed in a pre-chilled (-160 ºC) glovebox-fitted cold well following which 1 M HCl in diethyl ether (2.0 mL) was pipetted in bulb *B* and frozen. The apparatus was connected to the glovebox vacuum line, and placed under vacuum. After 5 minutes, the J. Young valve leading to the bulb *B* was closed. The resulting reaction mixture in diethyl ether was transferred to bulb *A* of the distillation apparatus fitted with a stirrer bar (*NOTE*: bulb *B* is still closed and under static vacuum). The J. Young valves for both the bulbs were closed, the apparatus was taken out of the glovebox, attached to a Schlenk line and bulb *A* was frozen. Under a strong flow of dinitrogen, a colourless solution of aqueous KOH (30%, 2.5 mL) was added *via* a syringe, after which the bulb *A* was closed and frozen (*NOTE*: bulb *B* remains closed throughout this manipulation). The reaction mixture was thawed and the resulting black suspension was stirred at r.t. for 1 h). The bulb containing 1 M HCl in diethyl ether (2.0 mL) (i.e. bulb *B*) was frozen and the volatiles of bulb *A* (i.e. the reaction mixture) were vacuum-transferred to bulb *B*. Bulb *B* was thawed and all the volatiles present in bulb *B* were evaporated under reduced pressure and the resulting white residue obtained was dried under vacuum for 30 mins. A quantitative ^{1}H NMR spectrum (**Figure S33**) of the white residue in d_6-DMSO (0.4 mL) with dimethylsulfone as the internal standard revealed the presence of $^{15}NH_4Cl$ in 30% yield (0.3 equiv. of $^{15}NH_4Cl$ per complex **A** used).

Reaction of A with KC_8 in diethyl ether under argon at -40 °C followed by acid quenching

A violet suspension of **A** (10.5 mg, 0.01 mmol, 1.0 equiv.) in diethyl ether (0.8 mL) at -40 °C was added to solid bronze KC_8 (3.0 mg, 0.02 mmol, 2.2 equiv.) under argon. No observable colour change was perceived immediately after addition. The reaction mixture was stirred at -40 °C for three days resulting in dark brown supernatant along with excess bronze KC_8 and graphite precipitate. The volatiles were evaporated under reduced pressure and the resulting reaction mixture was cooled to -80 °C. A cold (-80 °C) colourless solution of 2 M HCl in diethyl ether (1.0 mL) was added to the reaction mixture at -80 °C resulting in the concomitant formation of a black precipitate and a colourless supernatant. The reaction mixture was brought to room temperature (r.t.) and stirred at r.t. overnight (16 h). The volatiles were evaporated under reduced pressure and the reaction mixture was dried under vacuum for 4 h. ^{1}H NMR spectrum (**Figure S34**) of the resulting sample in d_6-DMSO (0.5 mL) revealed only the presence of resonances corresponding to the ligand and no peaks resembling the ammonium chloride triplet were observed, thereby confirming the activation and reduction of dinitrogen when the reaction is conducted under a dinitrogen atmosphere.

Reaction of A with KC_8 in hexane under dinitrogen at r.t. followed by acid quenching

A violet suspension of **A** (11.7 mg, 0.01 mmol, 1.0 equiv.) in hexane (0.8 mL) at r.t. was added to solid bronze KC_8 (3.4 mg, 0.03 mmol, 2.2 equiv.) under dinitrogen. No observable colour change was perceived immediately after addition. The reaction mixture was stirred at r.t. for three days resulting in dark red brown precipitate (in addition to excess bronze KC_8 and graphite) and a pale brown yellow supernatant. The volatiles were evaporated under reduced pressure and the resulting reaction mixture was cooled to -80 °C. A cold (-80 °C) colourless solution of 1 M HCl in diethyl ether (2.0 mL) was added to the reaction mixture at -80 °C resulting in the concomitant formation of a black precipitate and a colourless supernatant. The reaction mixture was brought to room temperature (r.t.) and stirred at r.t. overnight (16 h). The volatiles were evaporated under reduced pressure to eliminate any unreacted HCl, and resuspended in diethyl ether (2.0 mL). Inside the dinitrogen glovebox, the J. Young valve-capped distillation apparatus (*vide supra*, **Figure S1**) was placed in a pre-chilled (-160 ºC) glovebox-fitted cold well following which 1 M HCl in diethyl ether (2.0 mL) was pipetted in bulb *B* and frozen. The apparatus was connected to the glovebox vacuum line, and placed under vacuum. After 5 minutes, the J. Young valve leading to the bulb *B* was closed. The resulting reaction mixture in diethyl ether was transferred to bulb *A* of the distillation apparatus fitted with a stirrer bar (*NOTE*: bulb *B* is still closed and under static vacuum). The J. Young valves for both the bulbs were closed, the apparatus was taken out of the glovebox, attached to a Schlenk line and bulb *A* was frozen. Under a strong flow of dinitrogen, a colourless solution of NaO^tBu (10.9 mg, 0.1 mmol, 10.0 equiv.) in diethyl ether (1.5 mL) was added *via* a syringe, after which the bulb *A* was closed and thawed (*NOTE*: bulb *B* remains closed throughout this manipulation). The resulting black suspension was stirred at r.t. overnight (18 h). The bulb containing 1 M HCl in diethyl ether (2.0 mL) (i.e. bulb *B*) was frozen and the volatiles of bulb *A* (i.e. the reaction mixture) were vacuum-

transferred to bulb *B*. Bulb *B* was thawed and the entire apparatus was entered into the dinitrogen glovebox. The volatiles in bulb *B* were evaporated under reduced pressure and the resulting white residue obtained was dried under vacuum for 30 mins. Analysis of the ^{1}H NMR spectrum (**Figure S35**) of the remaining residue in d_6-DMSO (0.4 mL) did not reveal the presence of ammonium chloride.

Reaction of $H_2(^{tBu2}ArO)_2Me_2$-cyclam with HCl in diethyl ether

A cold (-80 °C) colourless solution of 2 M HCl in diethyl ether (1.5 mL) was added to solid $H_2(^{tBu2}ArO)_2Me_2$-cyclam (9.1 mg, 0.01 mmol, 1.0 equiv.) at -80 °C. No observable colour change was perceived immediately after addition. The reaction mixture was brought to room temperature (r.t.) and stirred at r.t. overnight (16 h) resulting in a pink precipitate and a colourless supernatant. The volatiles were evaporated under reduced pressure and the reaction mixture was dried under vacuum for 4 h. ^{1}H NMR spectrum (**Figure S36**) of the resulting sample in d_6-DMSO (0.5 mL) revealed the presence of resonances corresponding to the ligand.

B. NMR Spectroscopic Data

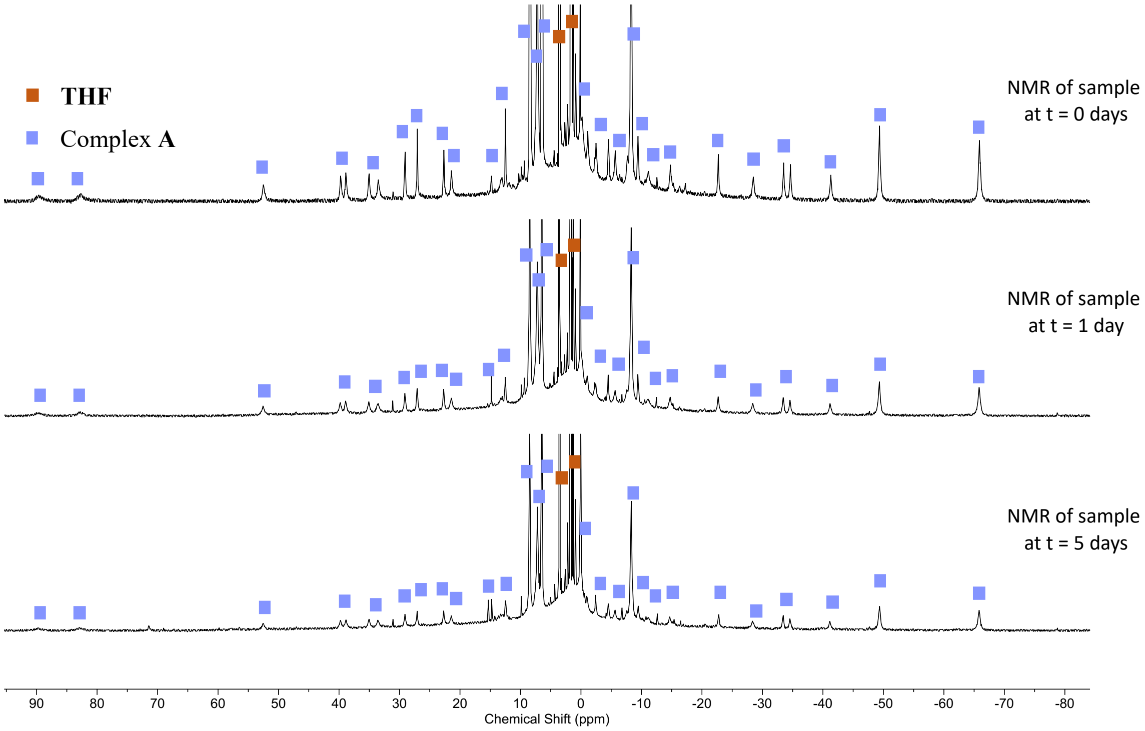

Figure S2. Evolution of the ^1H NMR spectrum (400 MHz, d_8-THF, 298 K) of a THF solution of isolated **A** after dissolution (sample was stored at r.t. at all times).

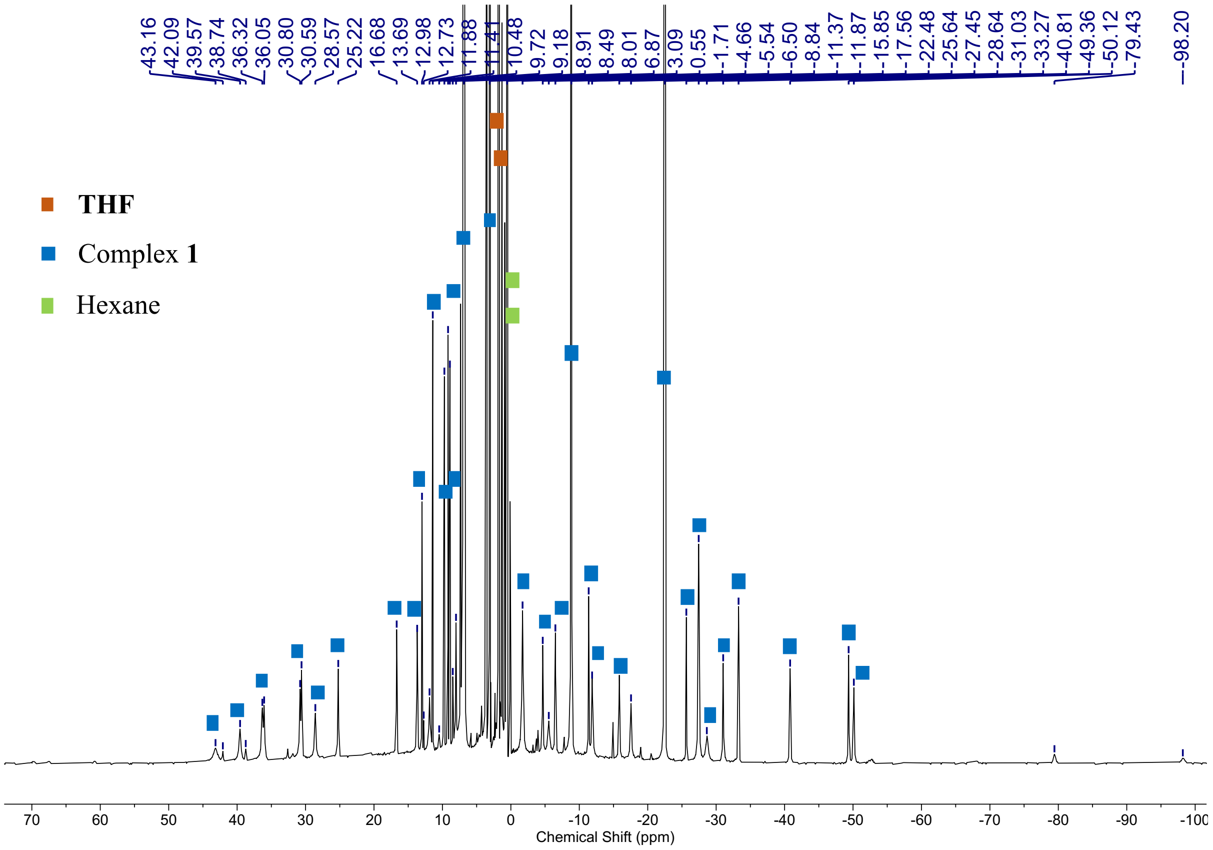

Figure S3. ^{1}H NMR spectrum (400 MHz, d_8-THF, 233 K) of the crude reaction mixture obtained by reacting **A** with KOSi(O^{t}Bu)$_3$ in THF at -40 °C overnight (20 h) to yield **1**.

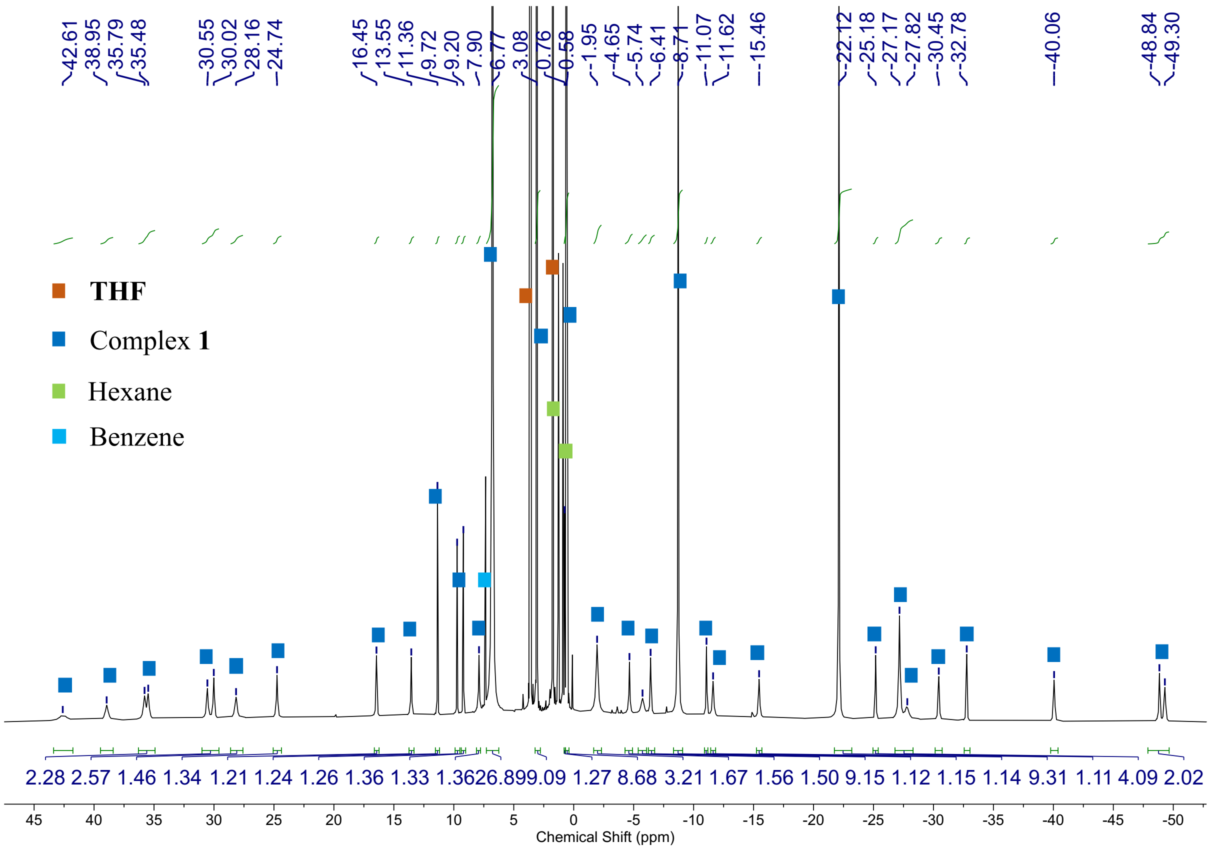

Figure S4. ^{1}H NMR spectrum (400 MHz, d_8-THF, 233 K) of isolated **1**.

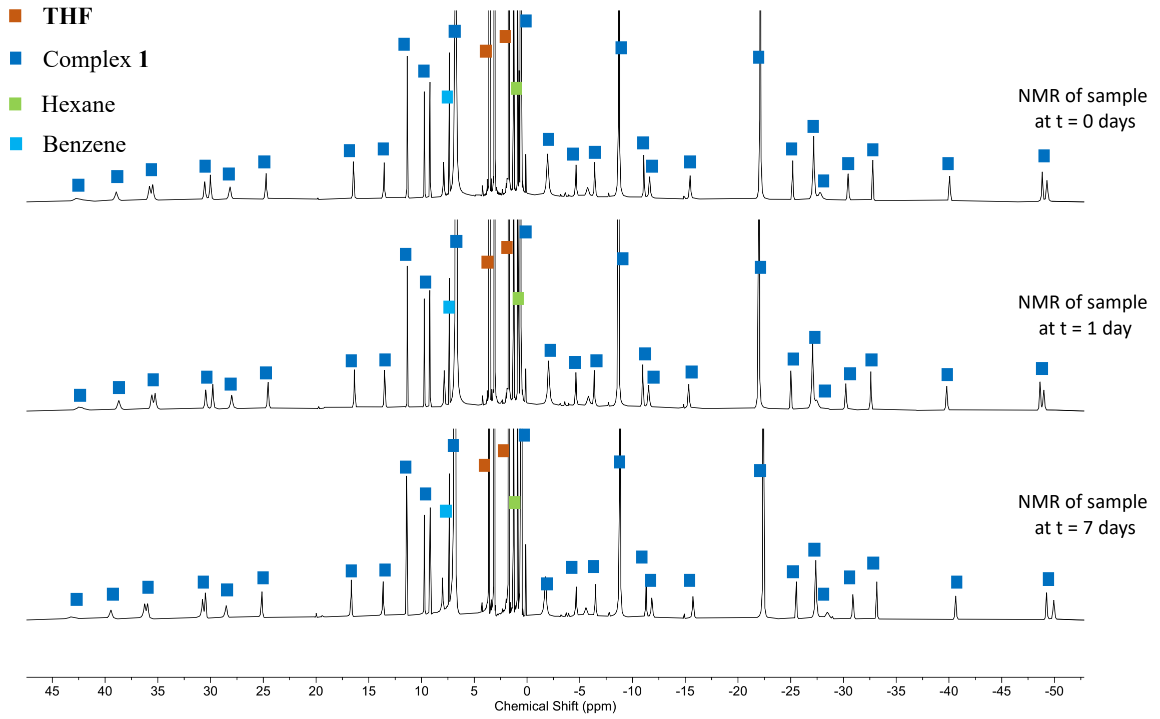

Figure S5. Evolution of the ^1H NMR spectrum (400 MHz, d_8-THF, 233 K) of a THF solution of isolated **1** after dissolution (sample was stored at -40 °C at all times).

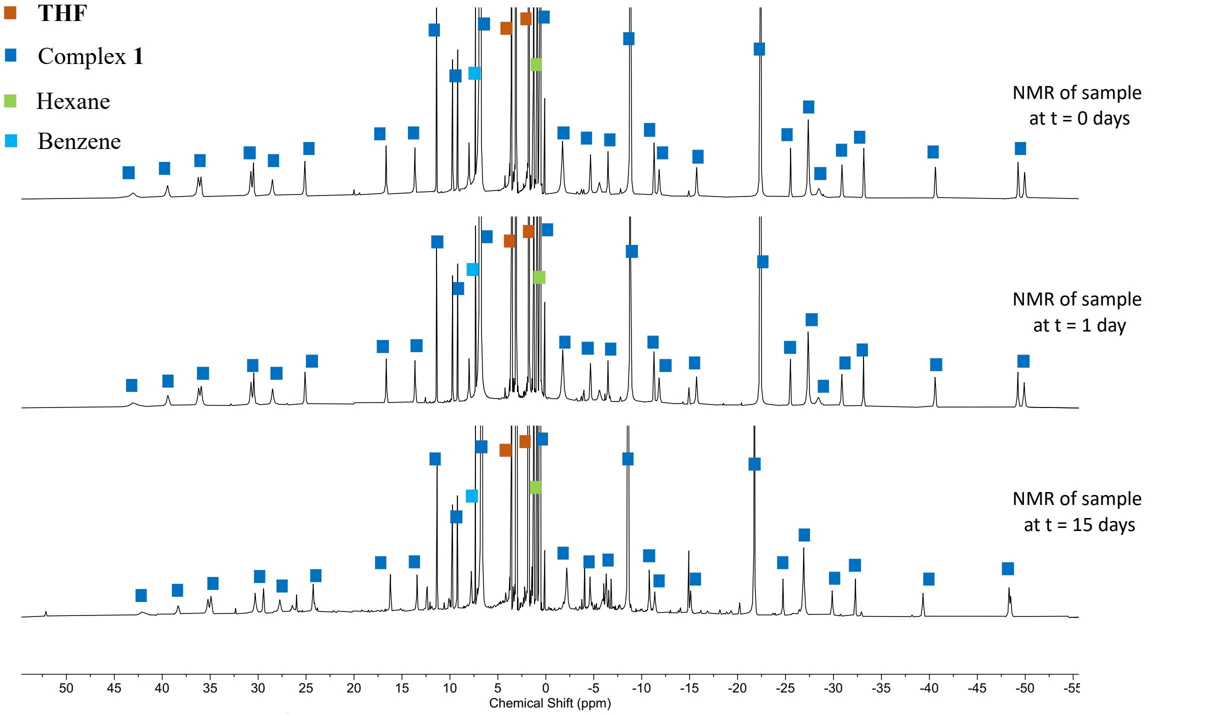

Figure S6. Evolution of the ^{1}H NMR spectrum (400 MHz, d_8-THF, 233 K) of a THF solution of isolated **1** after dissolution (sample was stored at r.t. at all times).

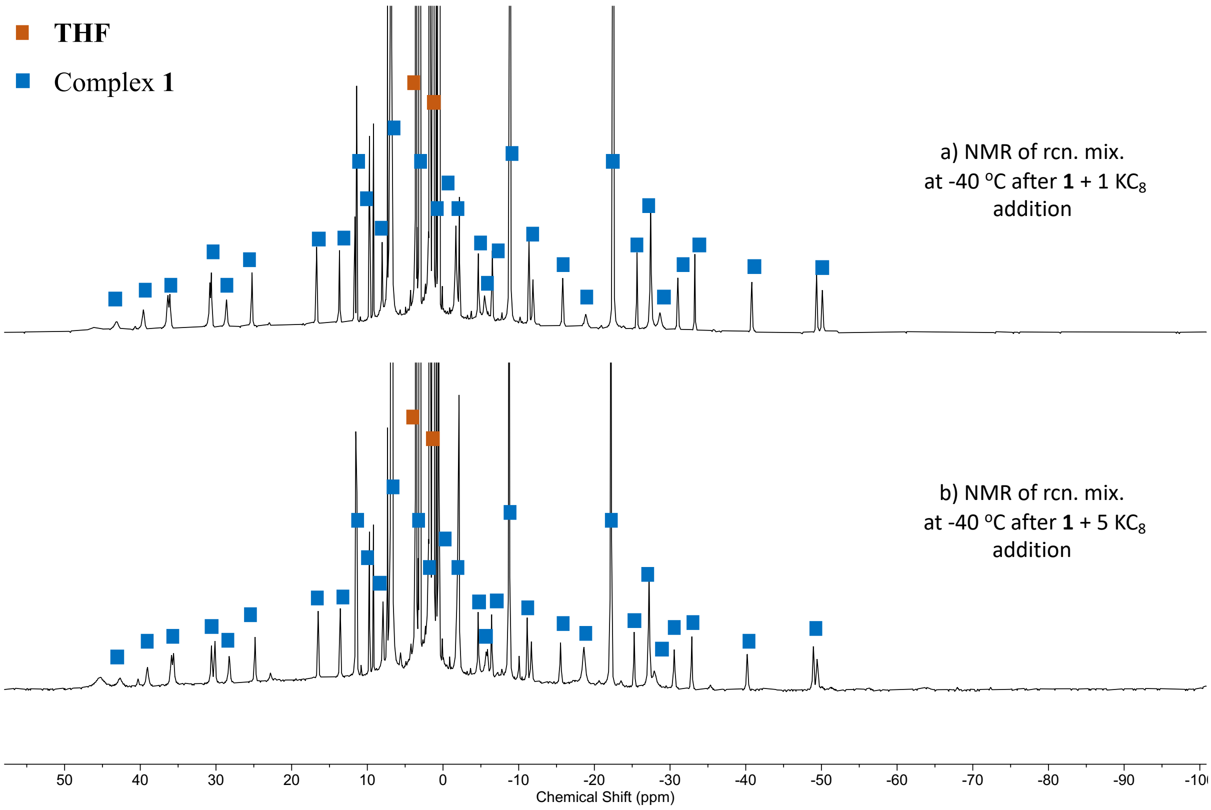

Figure S7. ^{1}H NMR spectrum (400 MHz, d_8-THF, 233 K) of a) the crude reaction mixture resulting from **1** + 1.0 equiv. of KC_8 overnight (14 h) in d_8-THF at -40 °C, b) the crude reaction mixture resulting from the addition of 4.0 equiv. of KC_8 to a) at -40 °C for 1 h.

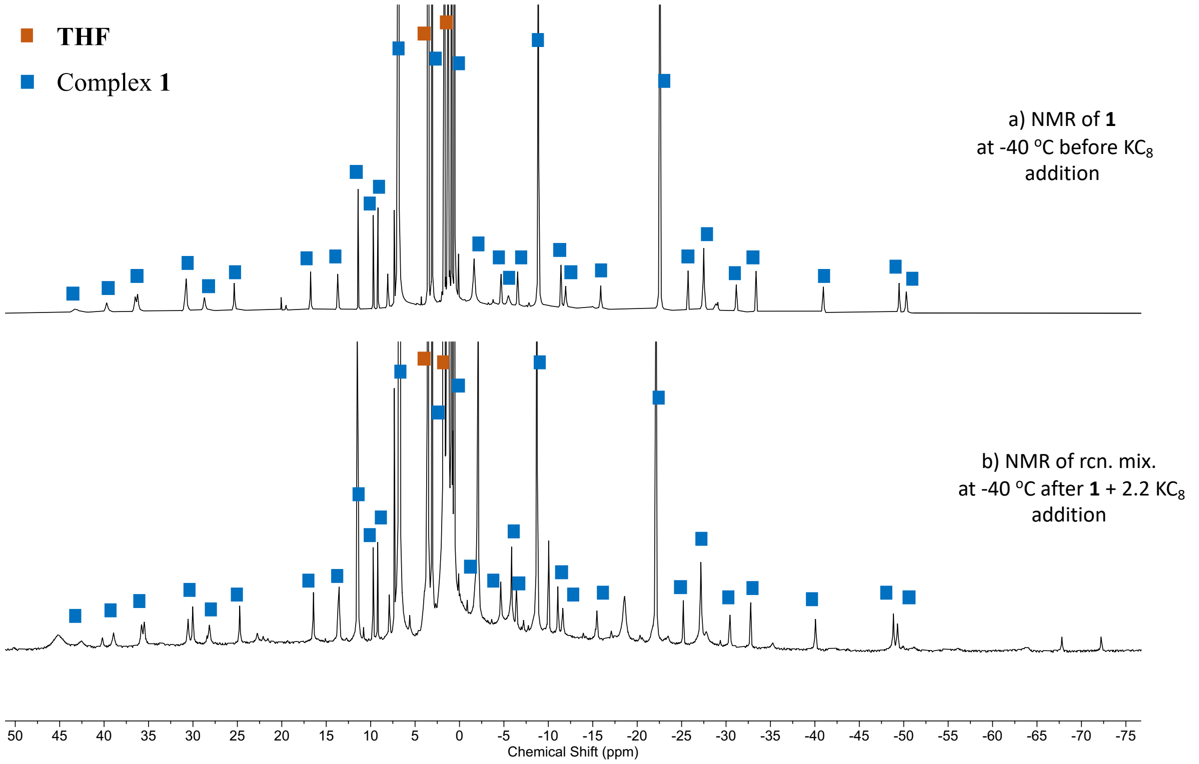

Figure S8. ^{1}H NMR spectrum (400 MHz, d_8-THF, 233 K) of a) complex **1** before addition of KC_8 and b) the crude reaction mixture resulting from **1** + 2.2 equiv. of KC_8 in d_8-THF overnight (14 h) at -40 °C.

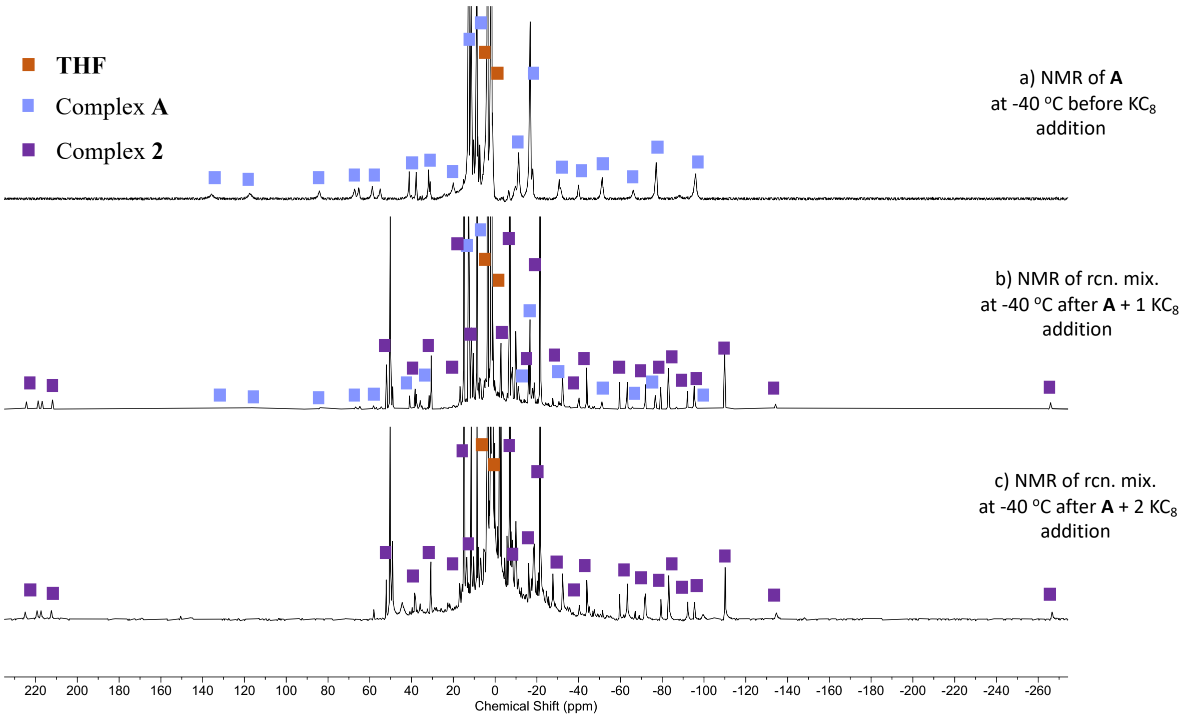

Figure S9. ^{1}H NMR spectrum (400 MHz, d_8-THF, 233 K) of a) complex **A** before addition of KC_8, b) the crude reaction mixture resulting from **A** + 1.0 equiv. of KC_8 in d_8-THF overnight (14 h) at -40 °C and c) the crude reaction mixture resulting from **A** + 2.0 equiv. of KC_8 in d_8-THF overnight (14 h) at -40 °C.

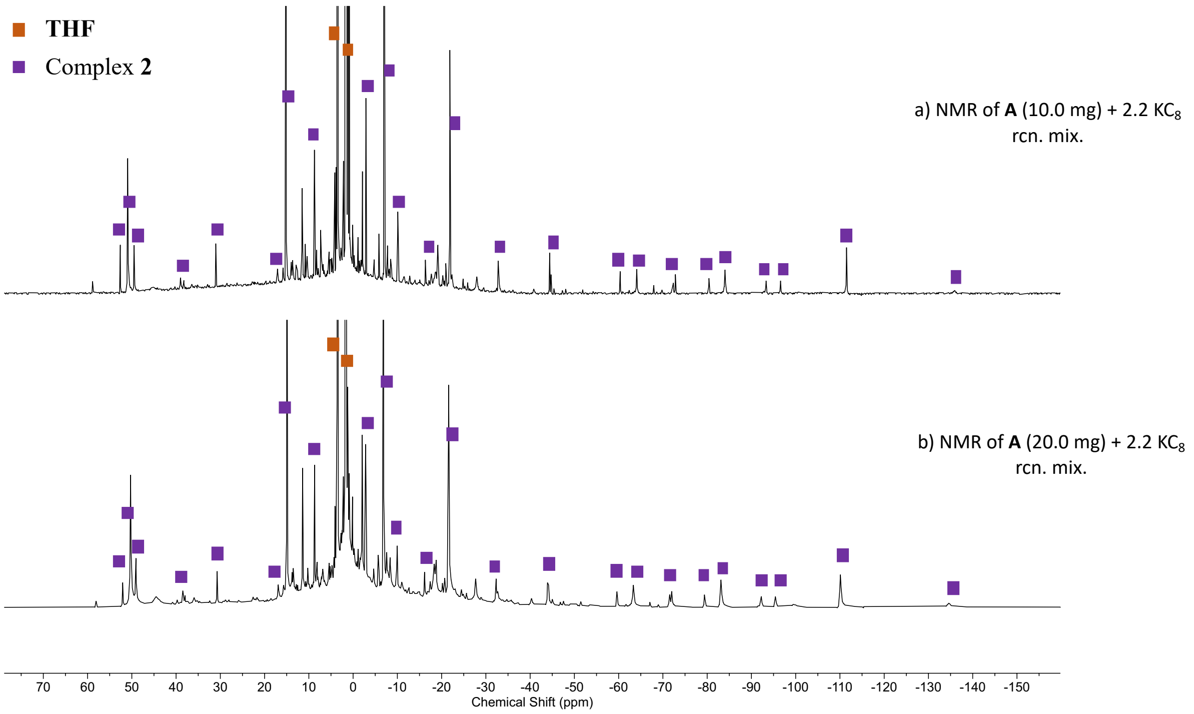

Figure S10. ^{1}H NMR spectrum (400 MHz, d_8-THF, 233 K) of the crude reaction mixture obtained by reacting **A** and 2.2 equiv. KC_8 at -40 °C in d_8-THF (0.5 mL) under argon overnight (14 h) to yield **2**, in differing concentrations of **A** (partial window shown for clarity).

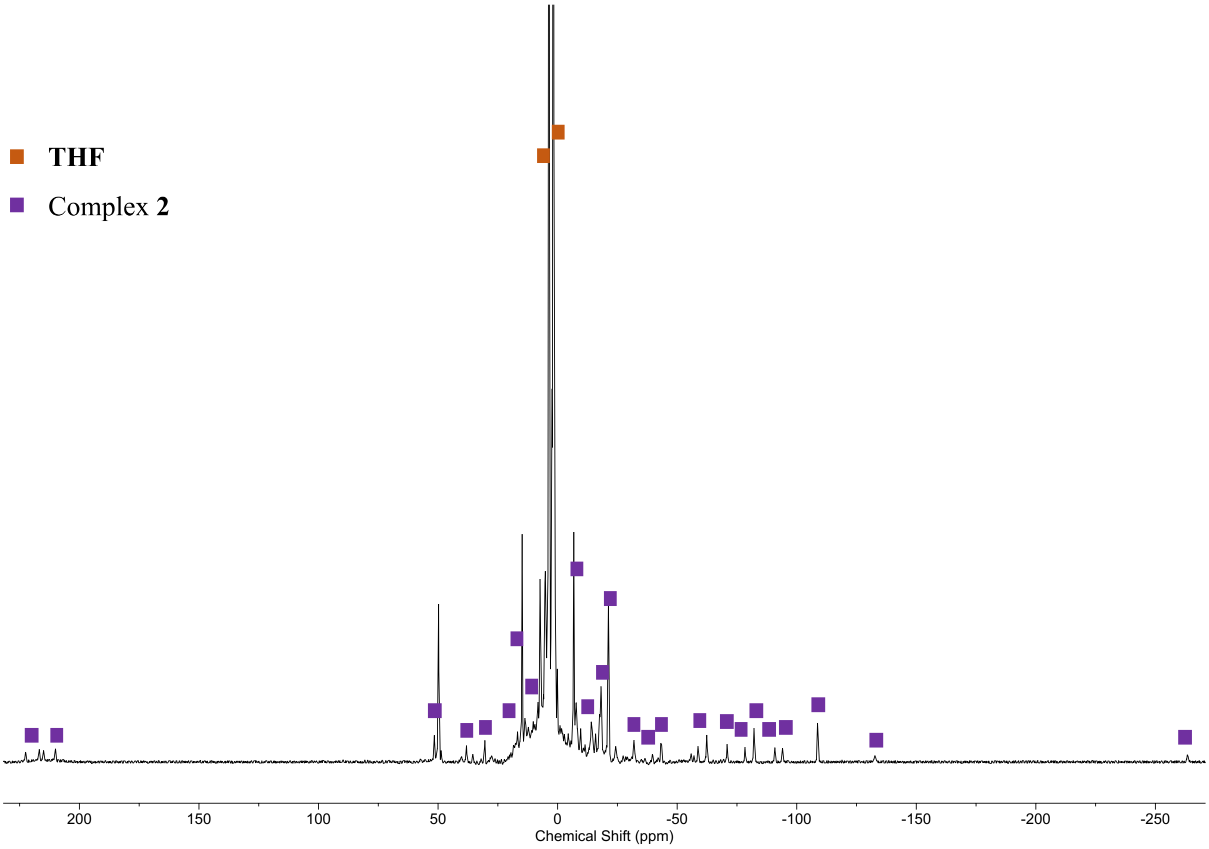

Figure S11. ^{1}H NMR spectrum (400 MHz, d_8-THF, 233 K) of the crude reaction mixture obtained by reacting **A** and 2.2 equiv. KC_8 at -40 °C in d_8-THF for one day under dinitrogen.

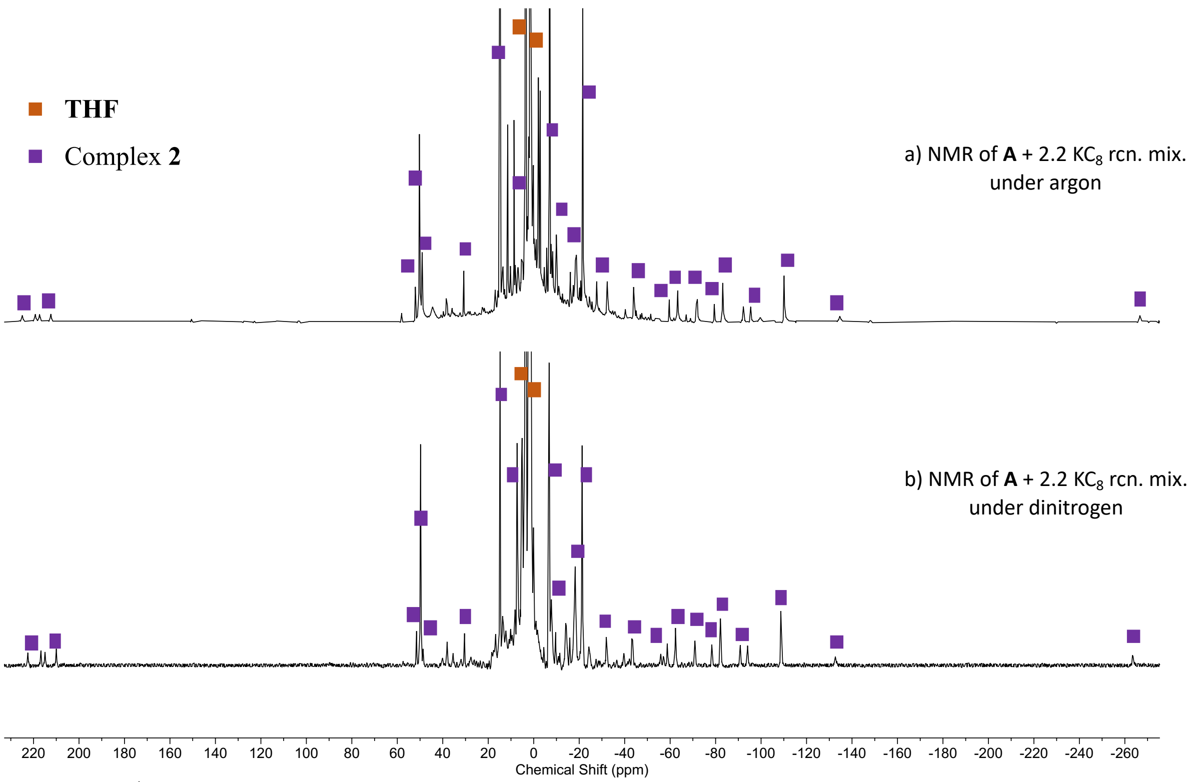

Figure S12. ^{1}H NMR spectrum (400 MHz, d_8-THF, 233 K) of the crude reaction mixture obtained by reacting **A** and 2.2 equiv. KC_8 at -40 °C in d_8-THF for one day under a) argon and b) dinitrogen.

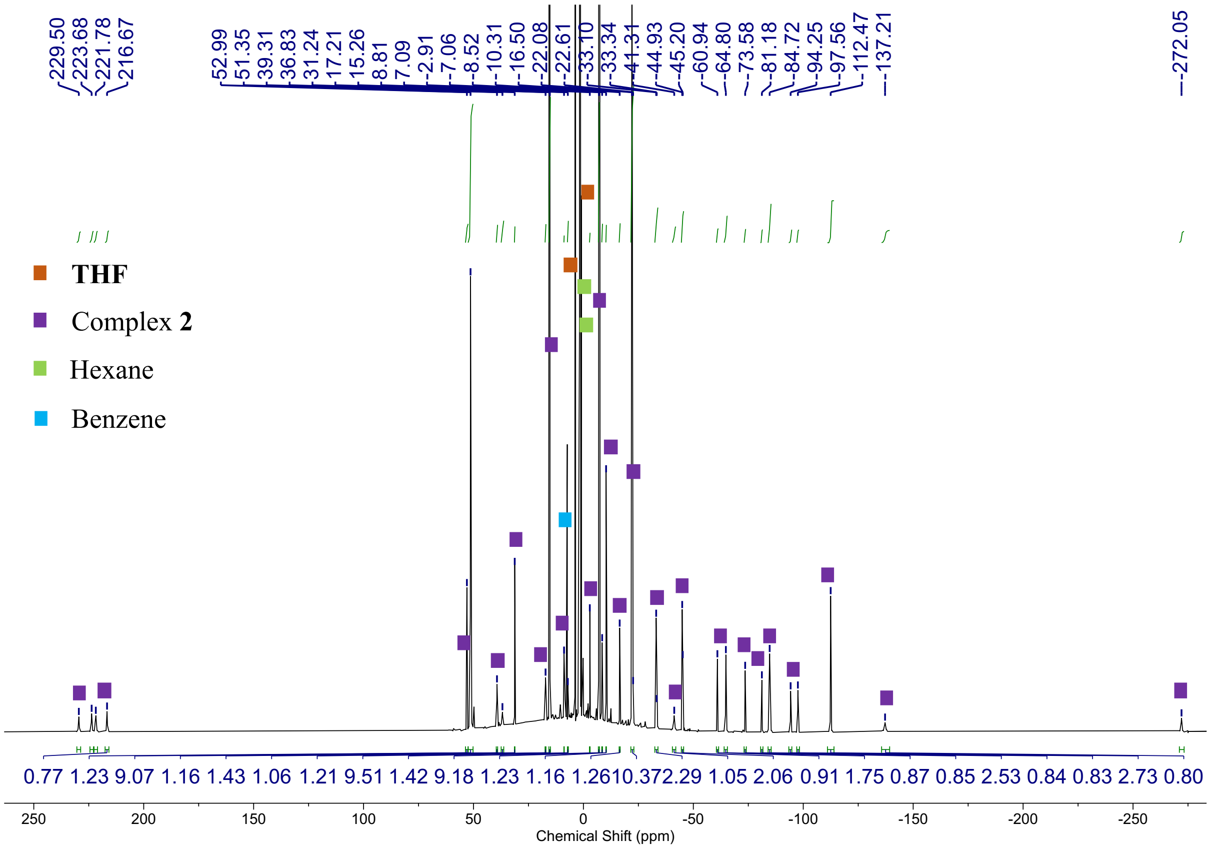

Figure S13. ^{1}H NMR spectrum (400 MHz, d_8-THF, 233 K) of isolated **2**.

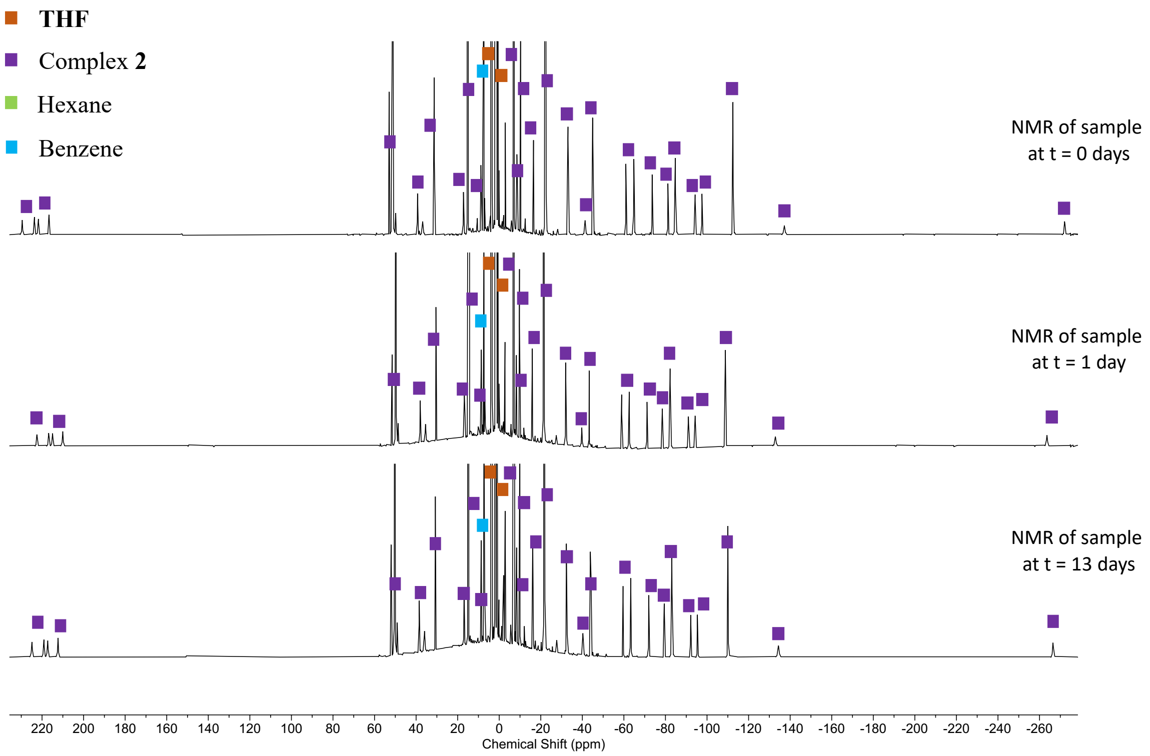

Figure S14. Evolution of the ^{1}H NMR spectrum (400 MHz, d_8-THF, 233 K) of a THF solution of isolated **2** after dissolution (sample was stored at -40 °C at all times).

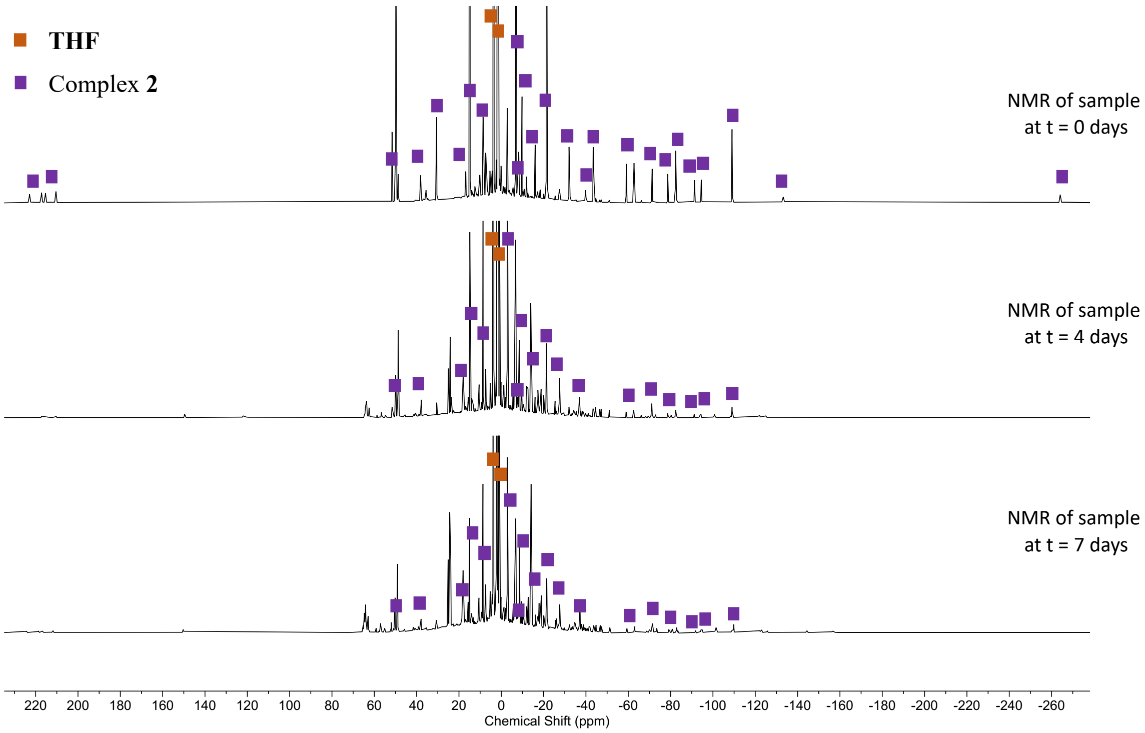

Figure S15. Evolution of the ^{1}H NMR spectrum (400 MHz, d_8-THF, 233 K) of a THF solution of isolated **2** after dissolution (sample was stored at r.t. at all times).

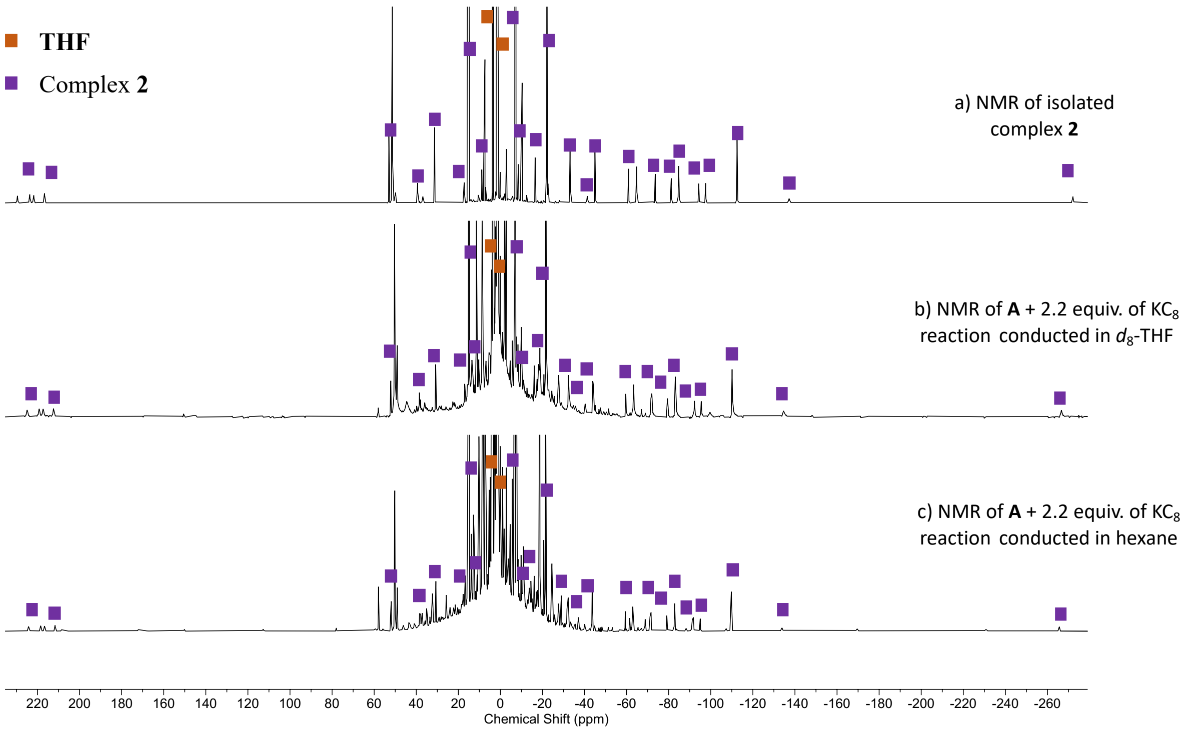

Figure S16. ^{1}H NMR spectrum (400 MHz, d_8-THF, 233 K) of a) isolated complex **2**, b) the crude reaction mixture obtained by reacting **A** and 2.2 equiv. KC_8 at -40 °C in d_8-THF overnight (14 h) and c) the crude reaction mixture obtained by reacting **A** and 2.2 equiv. KC_8 in hexane at r.t. for three days.

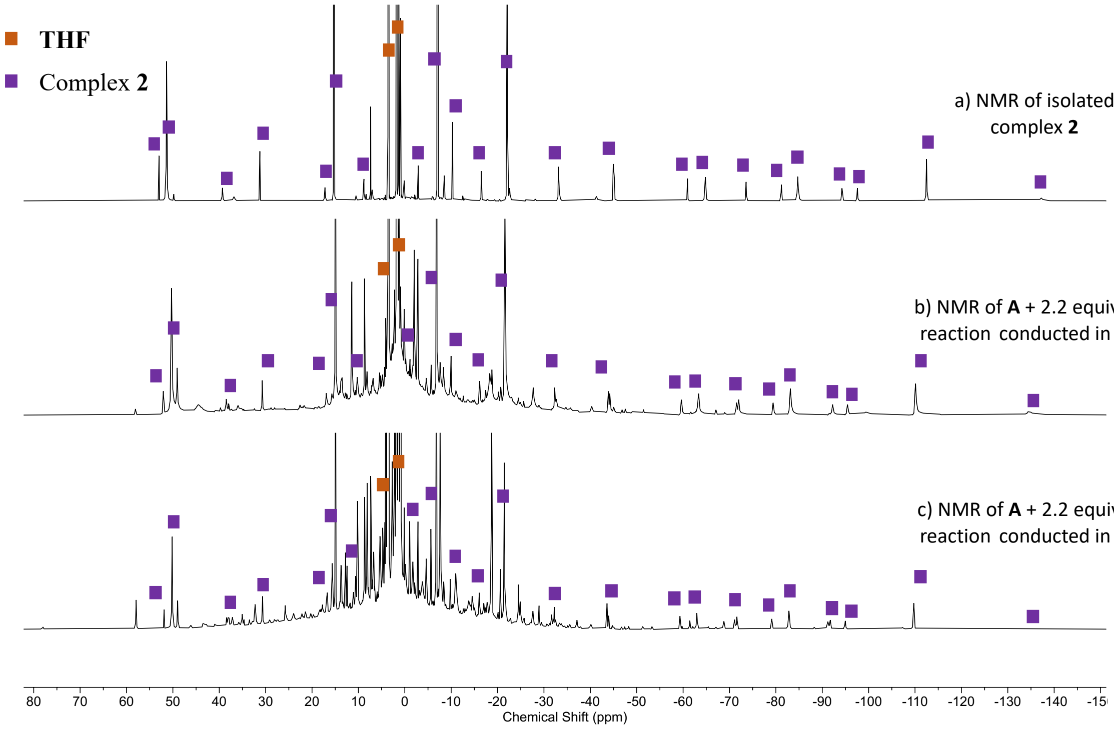

Figure S17. ^{1}H NMR spectrum (400 MHz, d_8-THF, 233 K) of a) isolated complex **2**, b) the crude reaction mixture obtained by reacting **A** and 2.2 equiv. KC_8 at -40 °C in d_8-THF overnight (14 h) and c) the crude reaction mixture obtained by reacting **A** and 2.2 equiv. KC_8 in hexane at r.t. for three days (*NOTE*: the spectra depicted are the same as **Figure S16** and a smaller spectral width has been displayed for clarity).

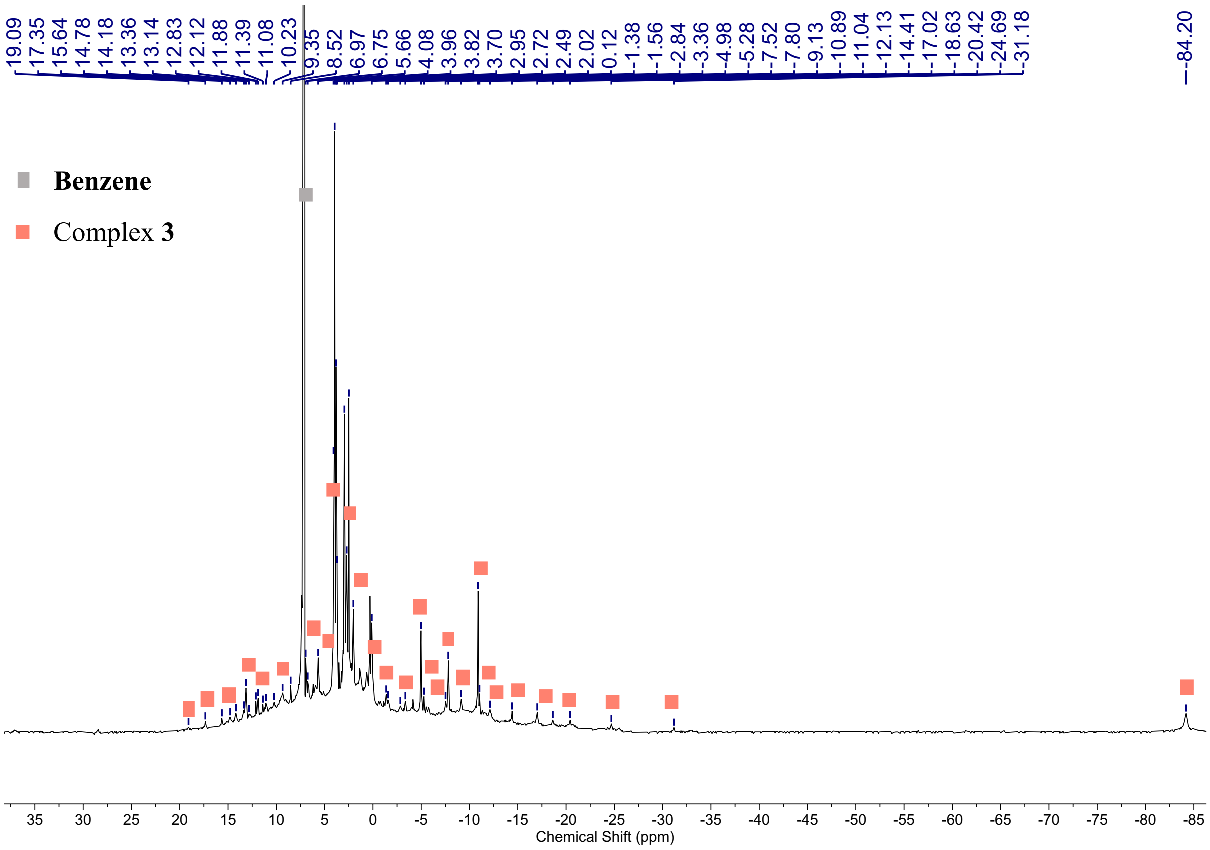

Figure S18. ^{1}H NMR spectrum (400 MHz, d_6-benzene, 298 K) of the crude reaction mixture obtained by reacting **A** and 2.2 equiv. of KC_8 at r.t. in benzene for one day to yield **3**.

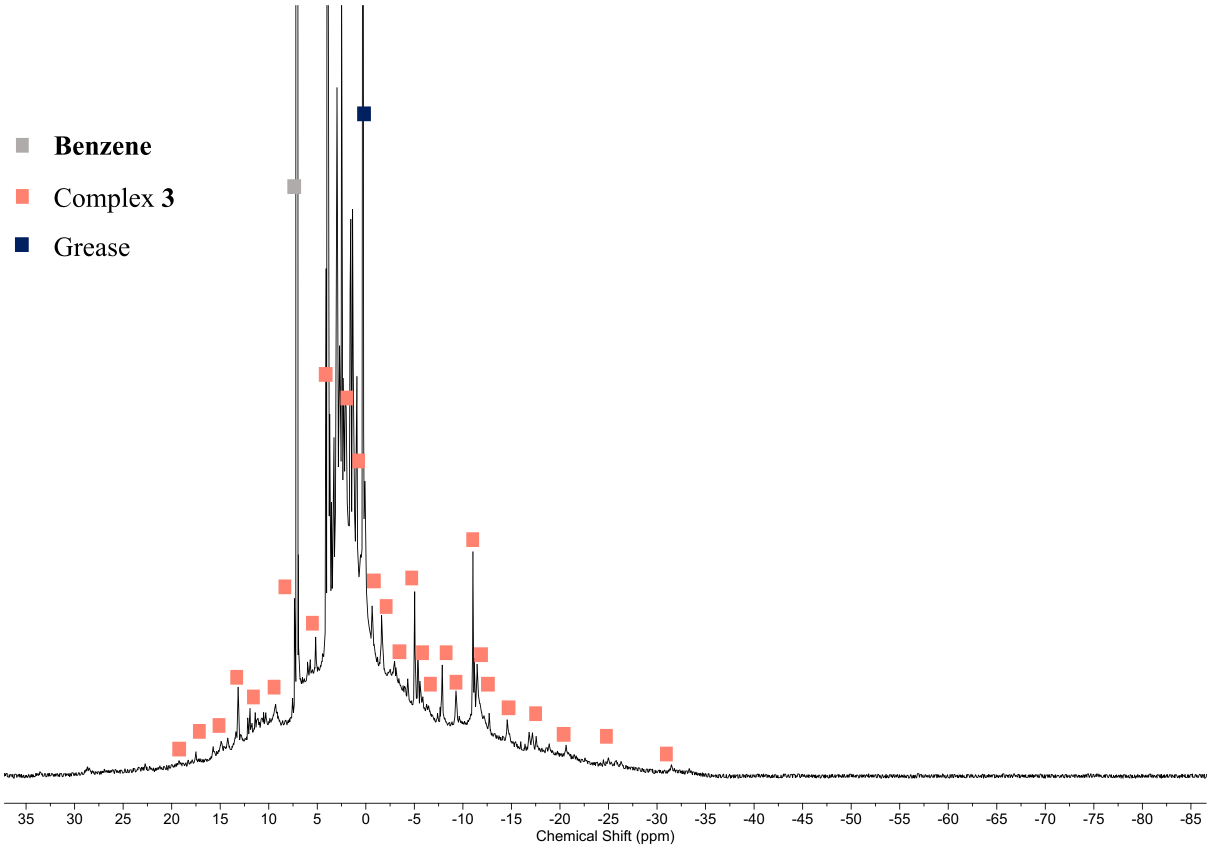

Figure S19. ^{1}H NMR spectrum (400 MHz, d_6-benzene, 298 K) of the crude reaction mixture obtained by reacting **A** and 2.2 equiv. of KC_8 at r.t. in d_6-benzene for one day under dinitrogen.

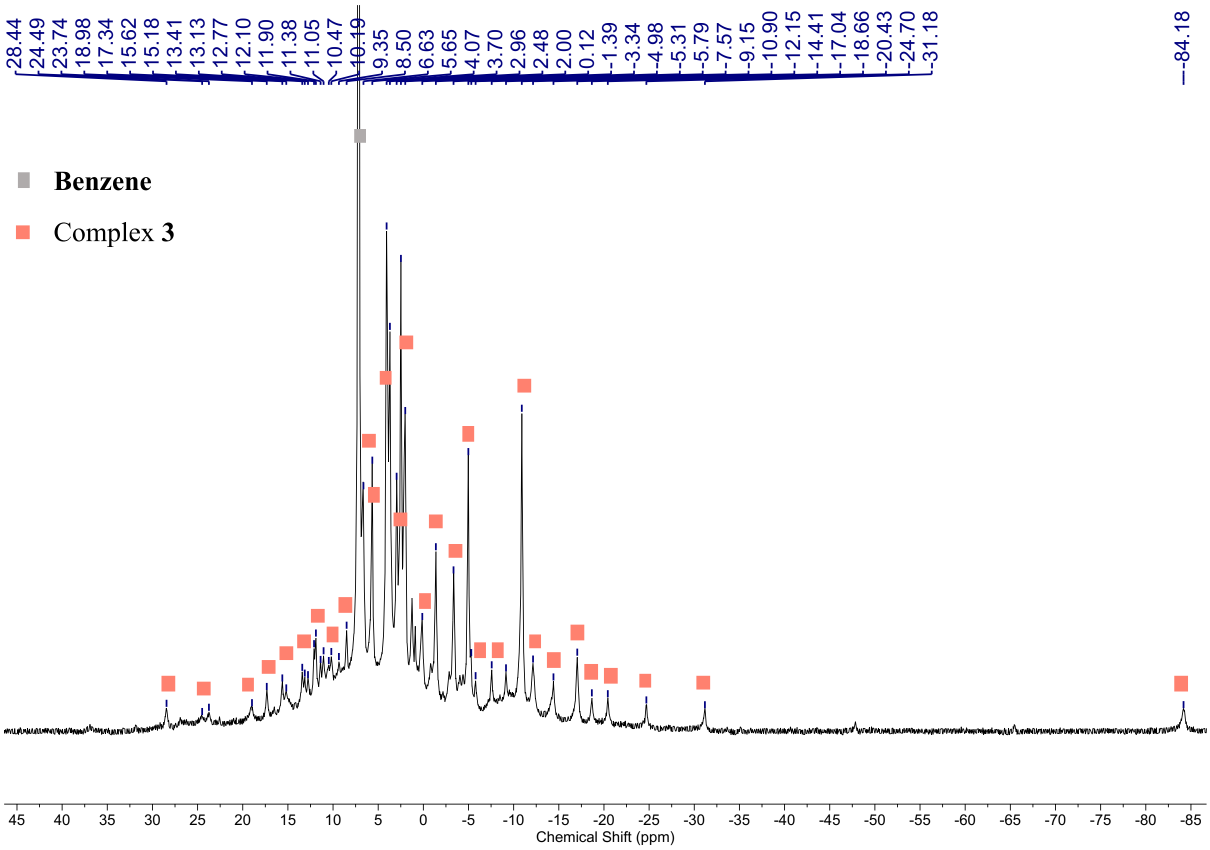

Figure S20. ^{1}H NMR spectrum (400 MHz, d_6-benzene, 298 K) of isolated **3**.

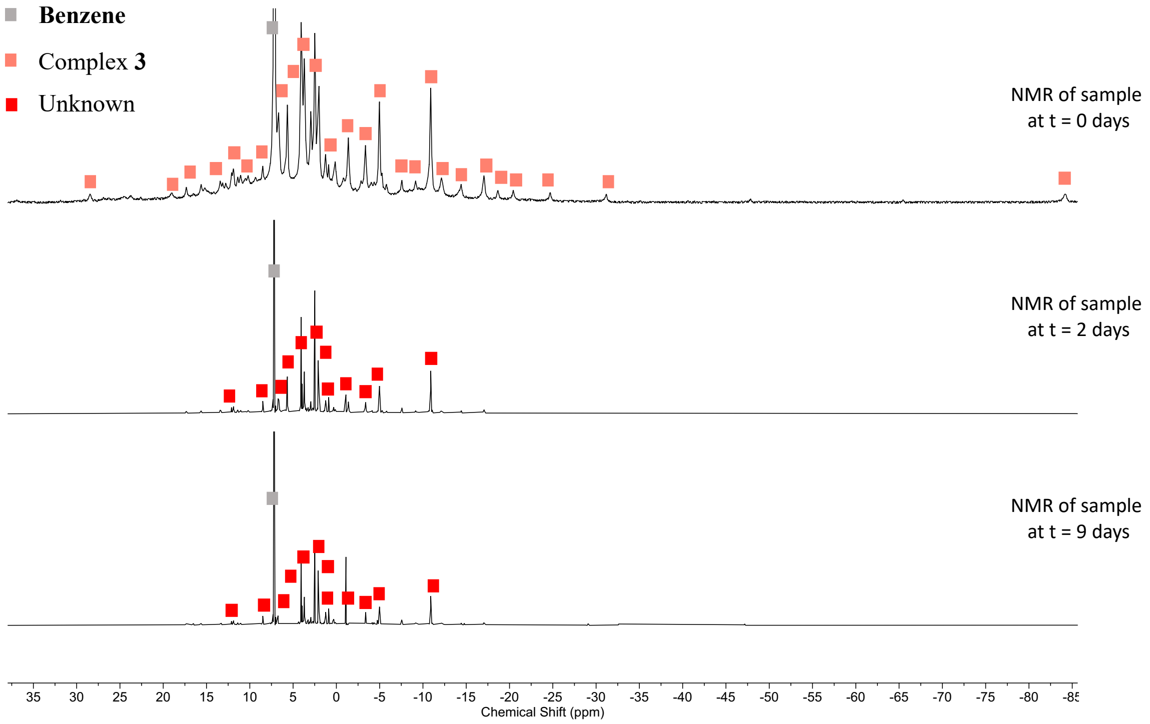

Figure S21. Evolution of the ^{1}H NMR spectrum (400 MHz, d_6-benzene, 298 K) of a benzene solution of isolated **3** after dissolution (sample was stored at r.t. at all times).

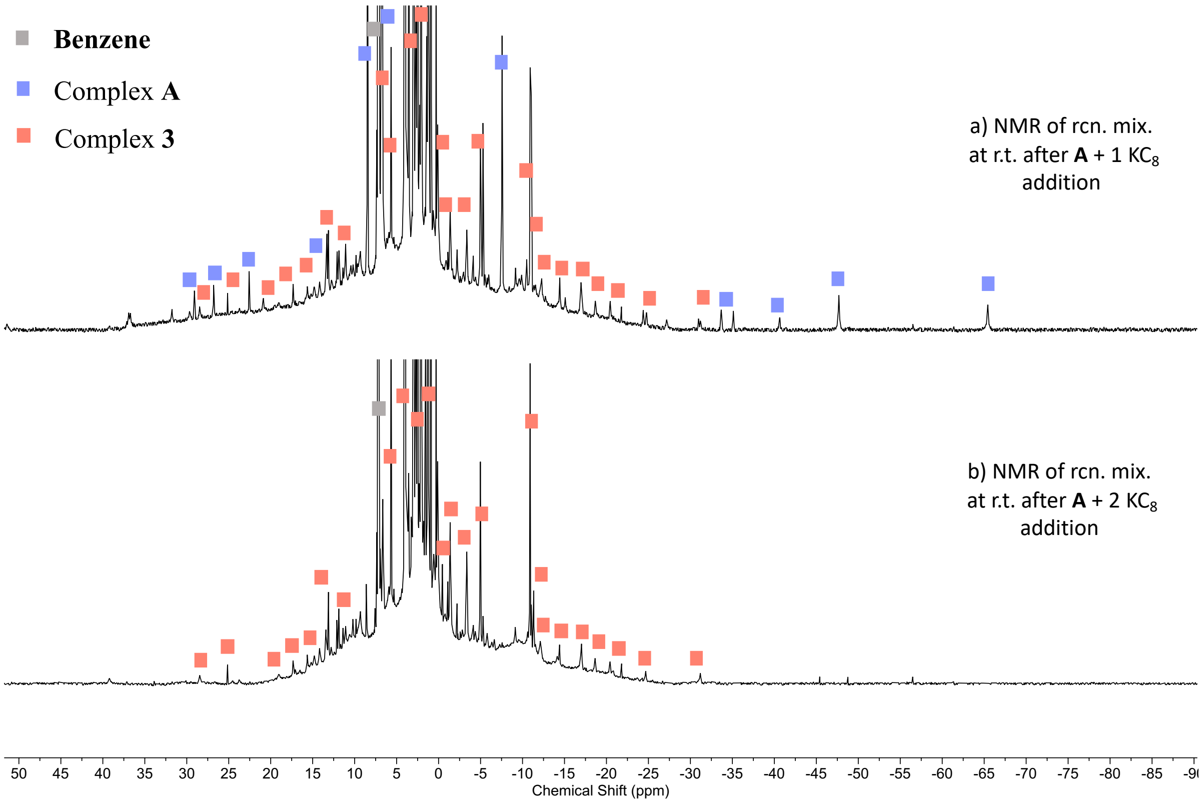

Figure S22. ^{1}H NMR spectrum (400 MHz, d_6-benzene, 298 K) of a) the crude reaction mixture resulting from **A** + 1.0 equiv. of KC_8 in d_6-benzene for one day at r.t. and b) the crude reaction mixture resulting from **A** + 2.0 equiv. of KC_8 in d_6-benzene for one day at r.t.

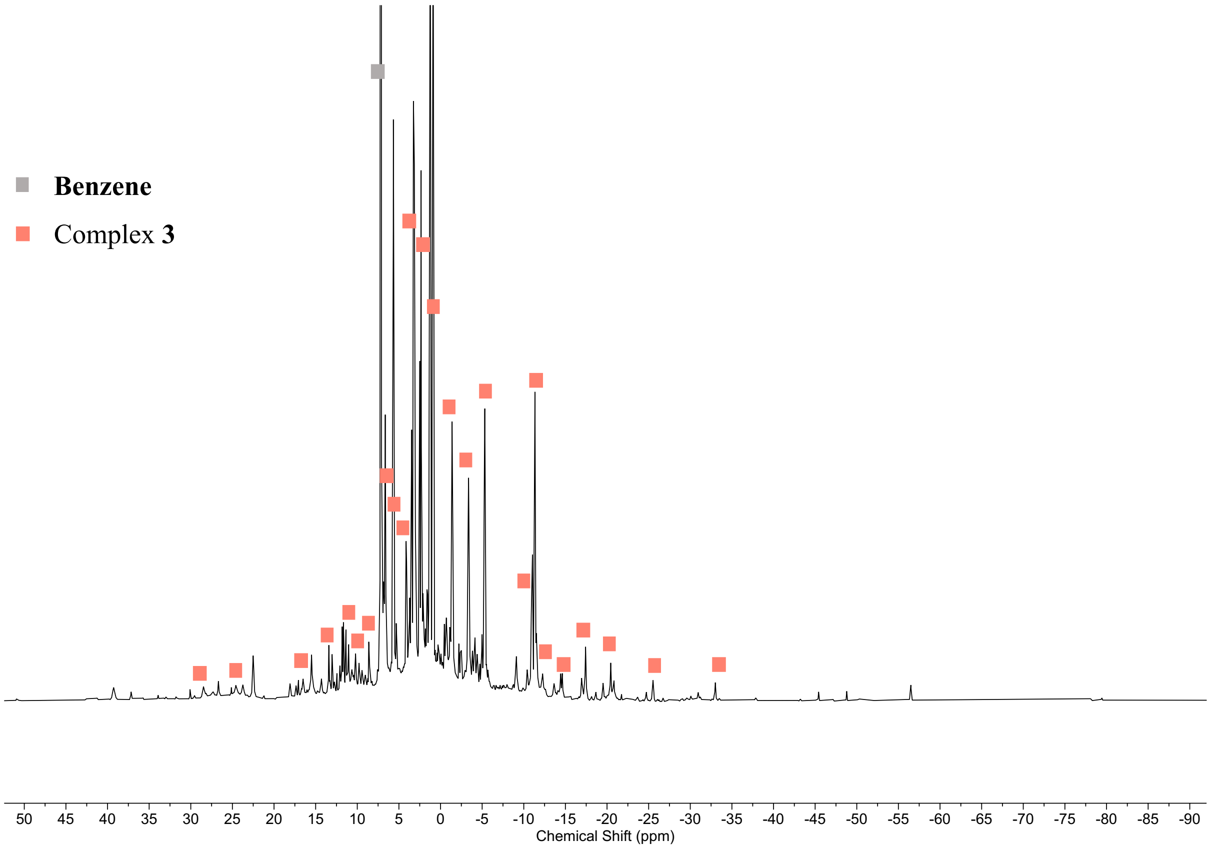

Figure S23. ^{1}H NMR spectrum (400 MHz, d_6-benzene, 298 K) of the crude reaction mixture obtained by reacting **A** and 2.2 equiv. KC_8 in hexane at r.t. for three days.

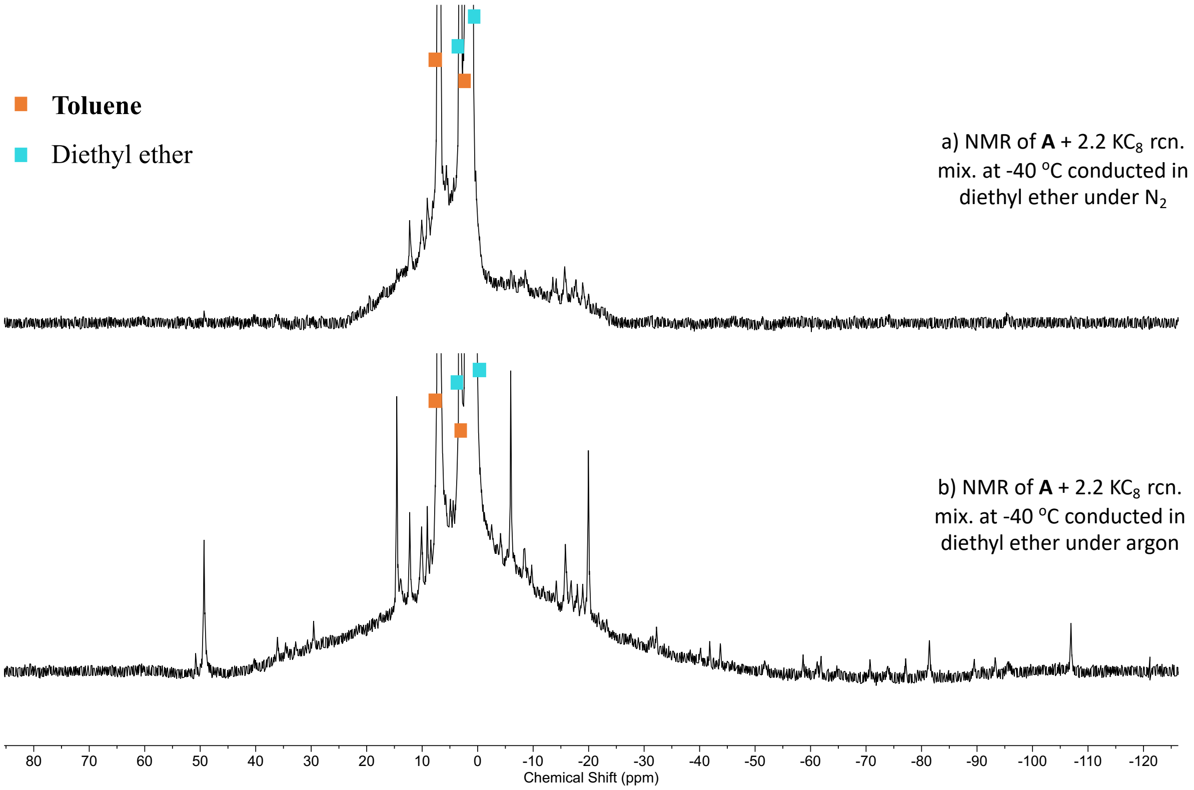

Figure S24. ^{1}H NMR spectrum (400 MHz, d_8-toluene, 233 K) of a) the crude reaction mixture resulting from **A** + 2.2 equiv. of KC_8 for three days in diethyl ether at -40 °C under dinitrogen and b) the crude reaction mixture resulting from **A** + 2.2 equiv. of KC_8 for three days in diethyl ether at -40 °C under argon.

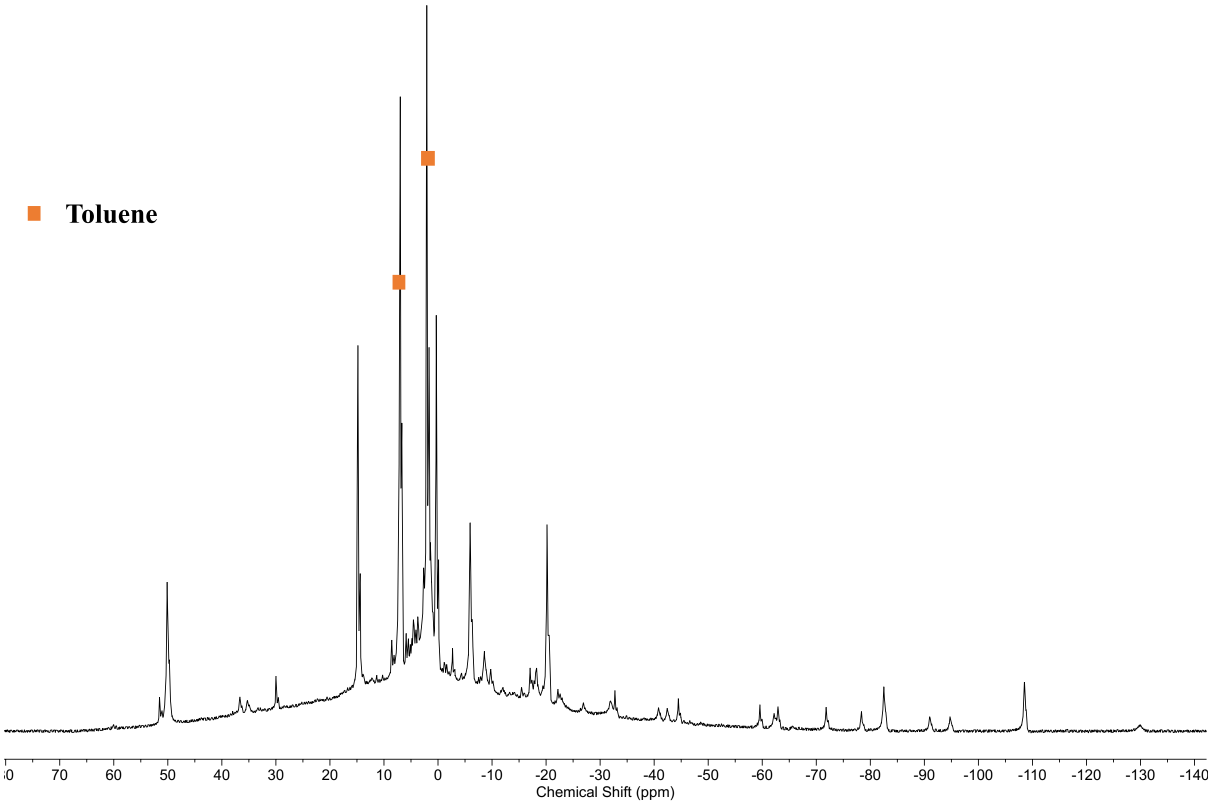

Figure S25. ^{1}H NMR spectrum (400 MHz, d_8-toluene, 233 K) of the crude reaction mixture obtained by reacting complex **A** with 2.2 equiv. of KC_8 in THF at -40 °C overnight (14 h).

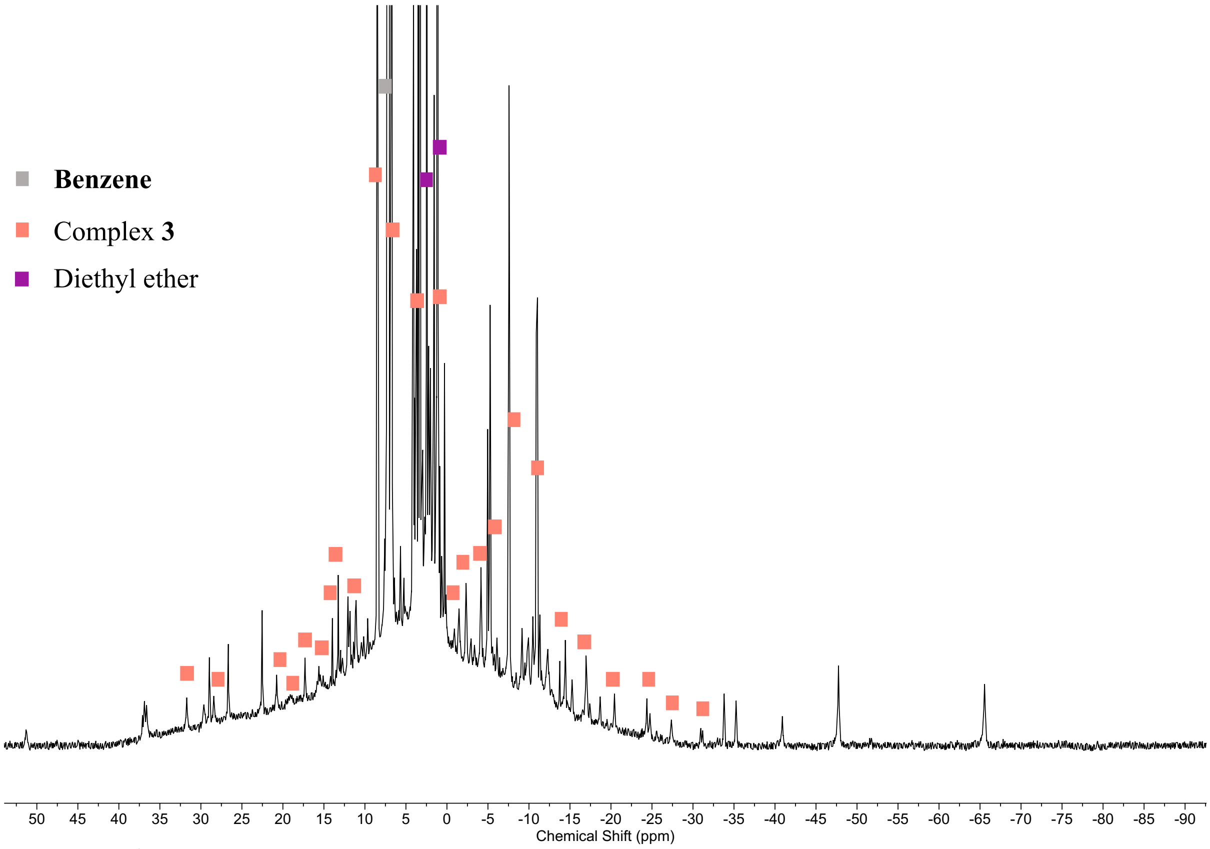

Figure S26. ^{1}H NMR spectrum (400 MHz, d_6-benzene, 298 K) of the crude reaction mixture obtained when the **A** + 2.2 equiv. of KC_8 reaction carried out under dinitrogen in diethyl ether for three days was suspended in d_6-benzene.

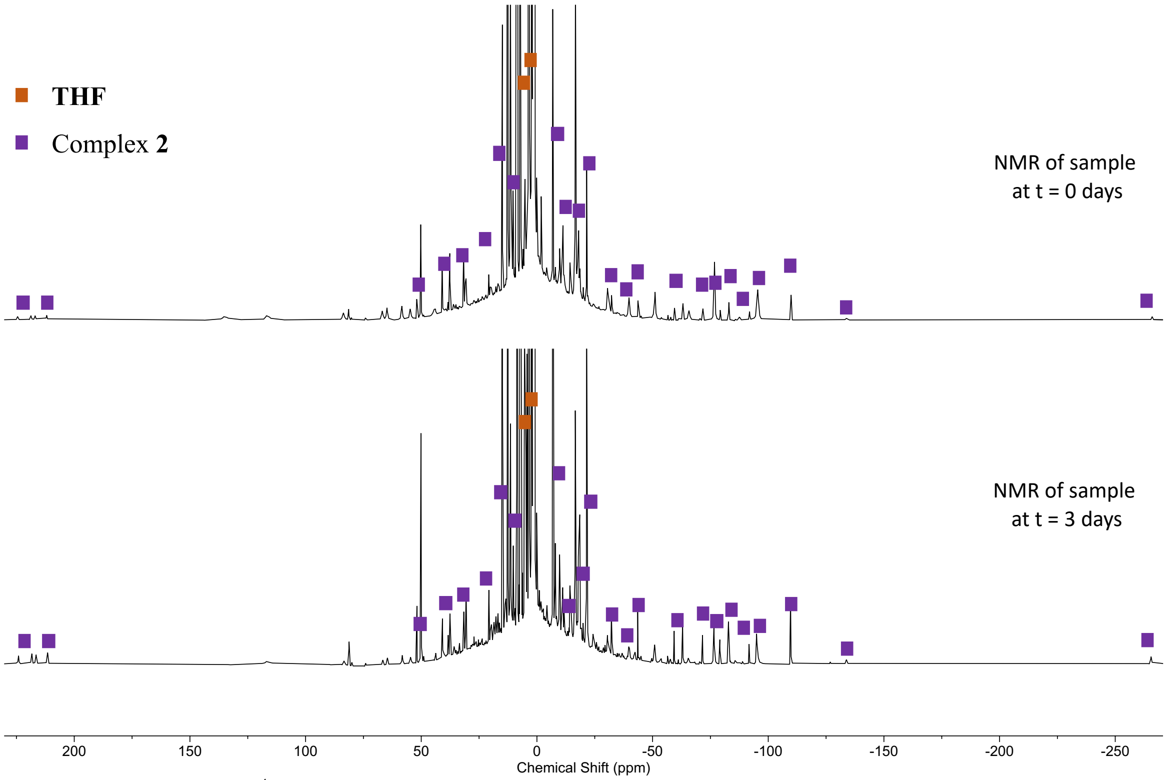

Figure S27. Evolution of ^{1}H NMR spectrum (400 MHz, d_8-THF, 233 K) of the crude reaction mixture obtained when the **A** + 2.2 equiv. of KC_8 reaction carried out under dinitrogen in diethyl ether for three days was suspended in cold (-40 °C) d_8-THF (sample was stored at -40 °C).

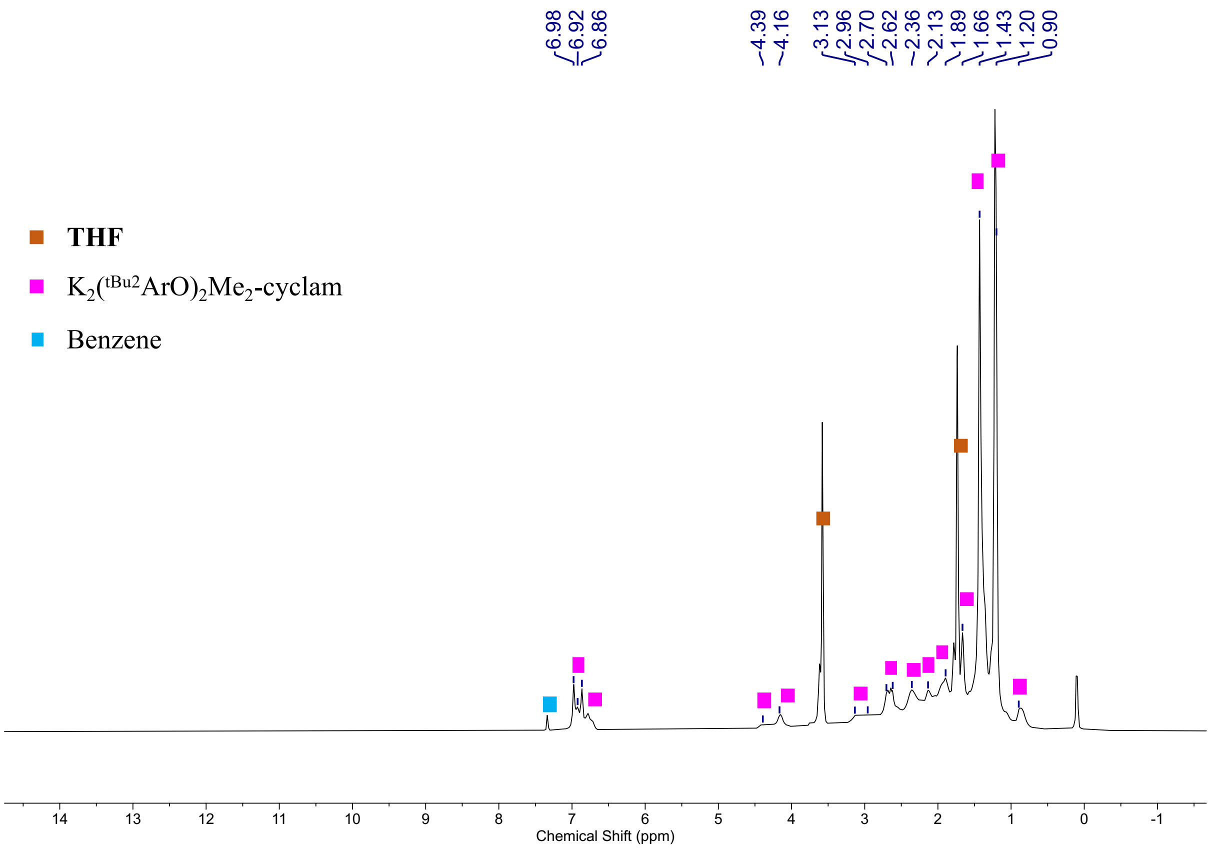

Figure S28. ^{1}H NMR spectrum (400 MHz, d_8-THF, 233 K) of $K_2(^{tBu2}ArO)_2Me_2$-cyclam.

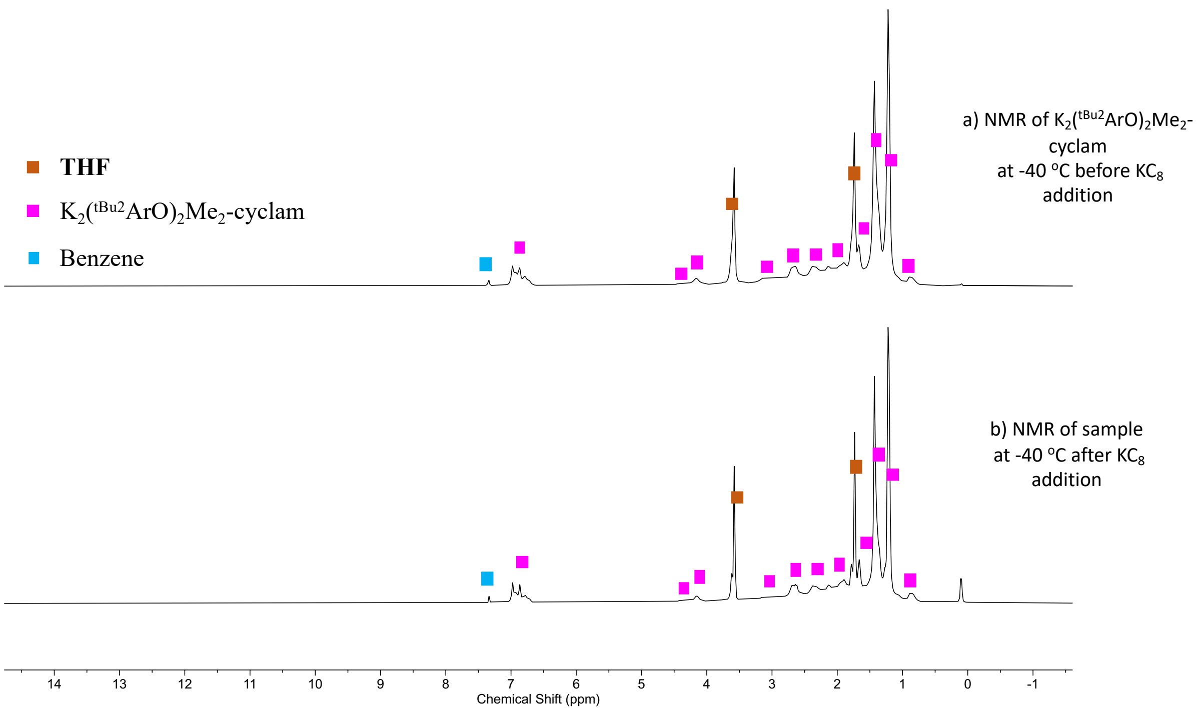

Figure S29. ^{1}H NMR spectrum (400 MHz, d_8-THF, 233 K) of a) $K_2(^{tBu2}ArO)_2Me_2$-cyclam before addition of KC_8 and b) the reaction mixture (after filtration to remove KC_8) obtained by reacting $K_2(^{tBu2}ArO)_2Me_2$-cyclam with 2.2 equiv. of KC_8 in d_8-THF at -40 °C for one day.

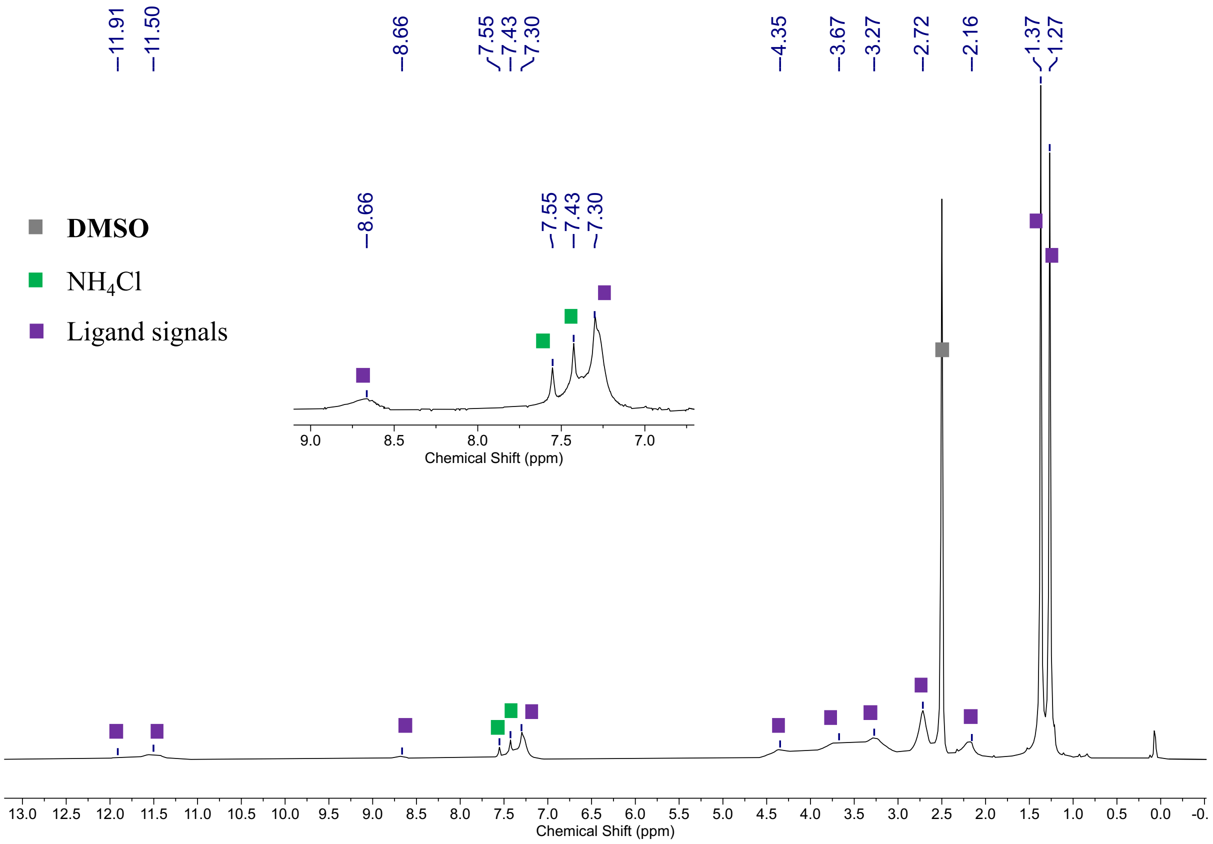

Figure S30. ^{1}H NMR spectrum (400 MHz, *d*$_6$-DMSO, 298 K) of the resulting reaction mixture obtained upon acid quenching (HCl in diethyl ether) of the reaction of **A** with KC_8 (2.2 equiv.) in diethyl ether at -40 °C for three days at under dinitrogen.

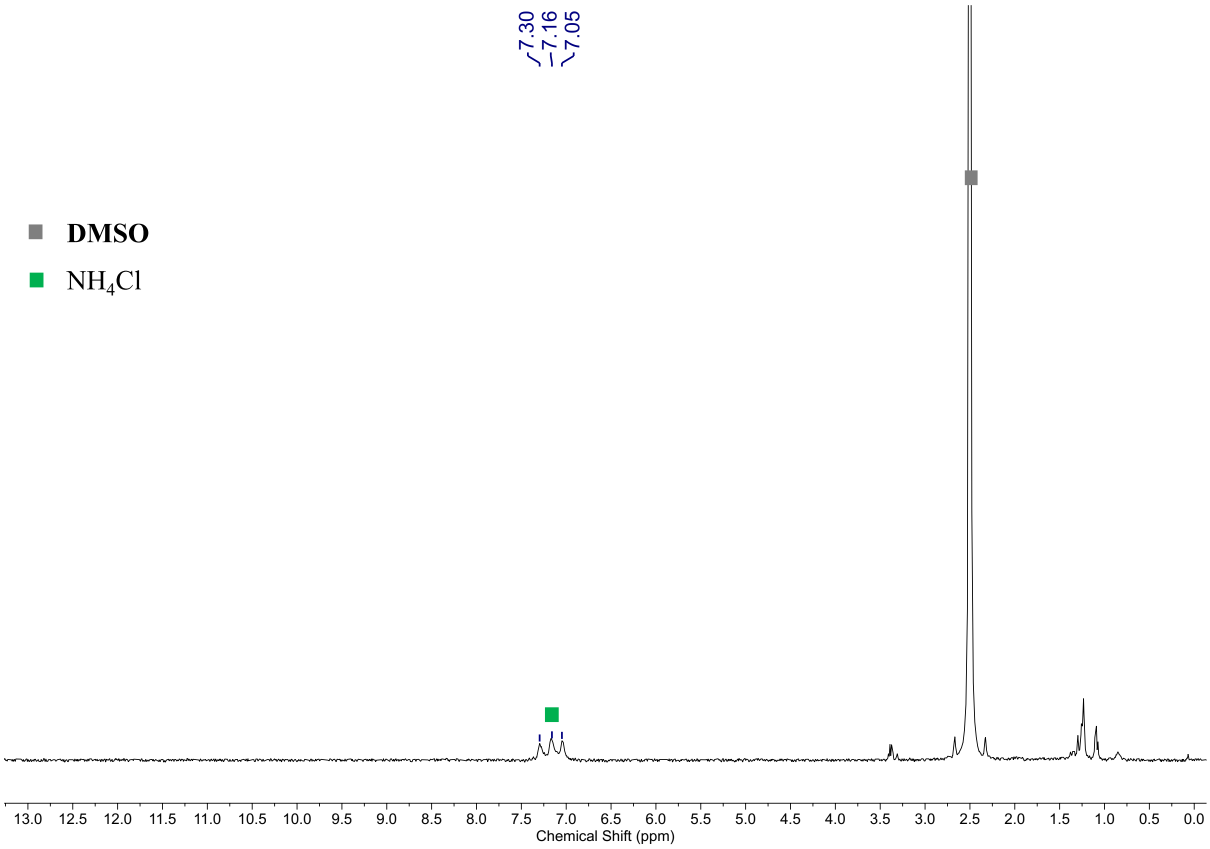

Figure S31. ^{1}H NMR spectrum (400 MHz, d_6-DMSO, 298 K) of the white residue obtained by the volatiles-transfer experiment conducted on the reaction of **A** with KC_8 (2.2 equiv.) in diethyl ether at -40 °C for three days at under dinitrogen.

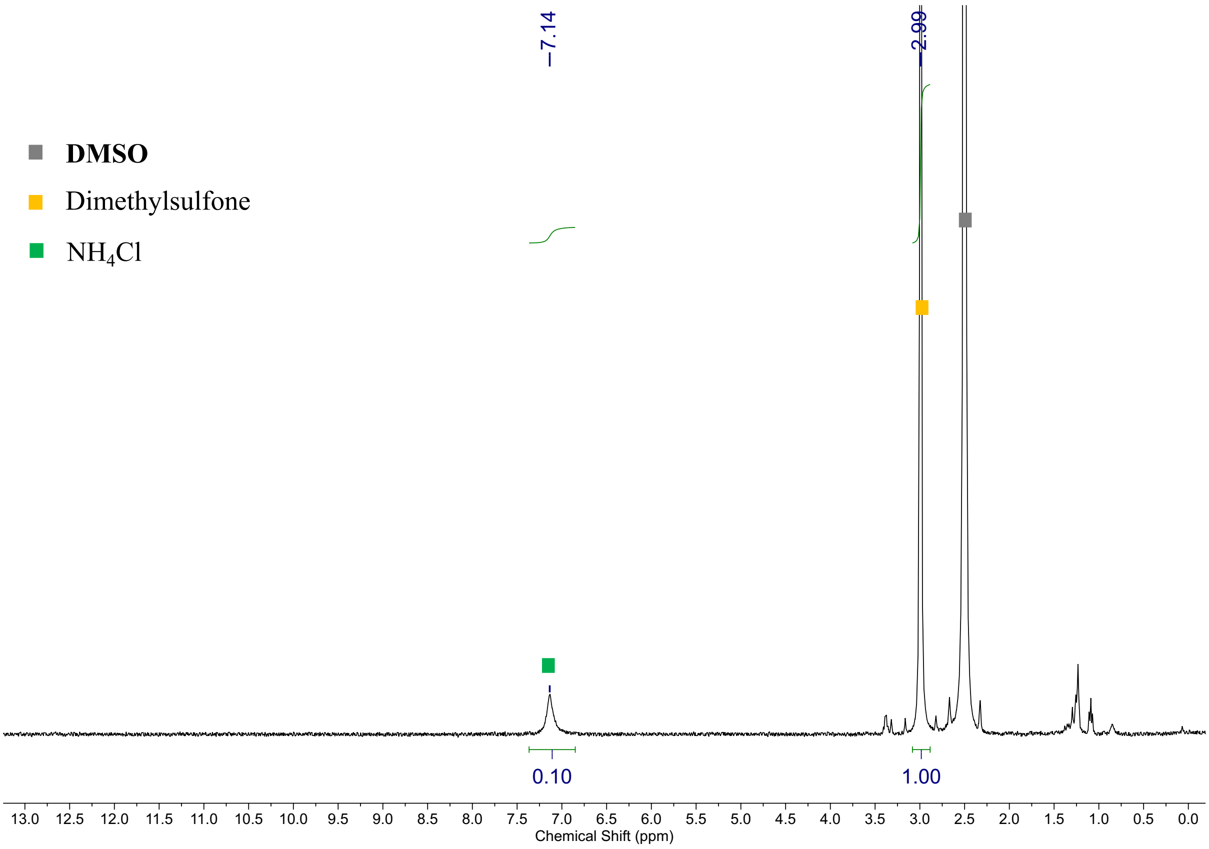

Figure S32. Quantitative ^{1}H NMR spectrum (400 MHz, d_6-DMSO, 298 K) of the white residue obtained by the volatiles-transfer experiment conducted on the reaction of **A** with KC_8 (2.2 equiv.) in diethyl ether at -40 °C for three days at under dinitrogen (dimethylsulfone used as the internal standard).

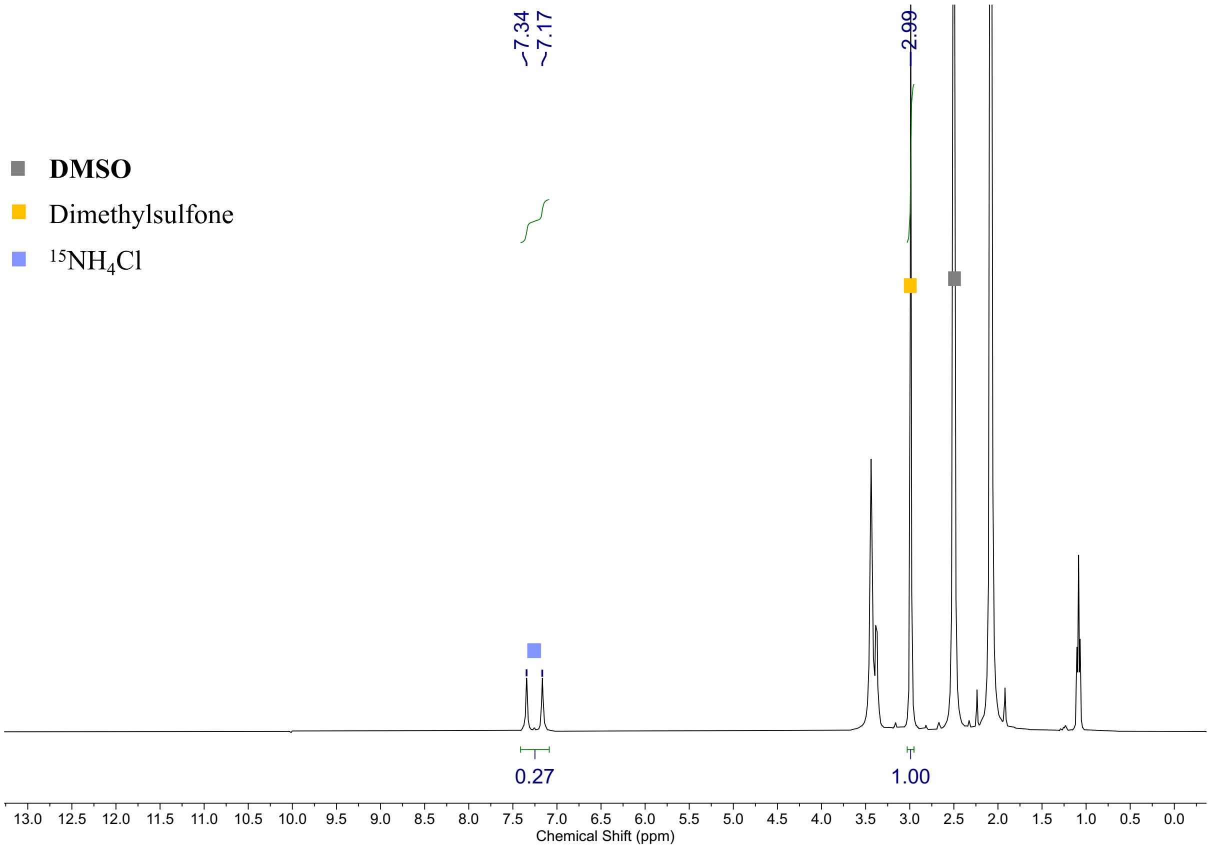

Figure S33. Quantitative ^{1}H NMR spectrum (400 MHz, d_6-DMSO, 298 K) of the white residue obtained by the volatiles-transfer experiment conducted on the reaction of **A** with KC_8 (2.2 equiv.) in diethyl ether at -40 °C for three days at under $^{15}N_2$ (dimethylsulfone used as the internal standard).

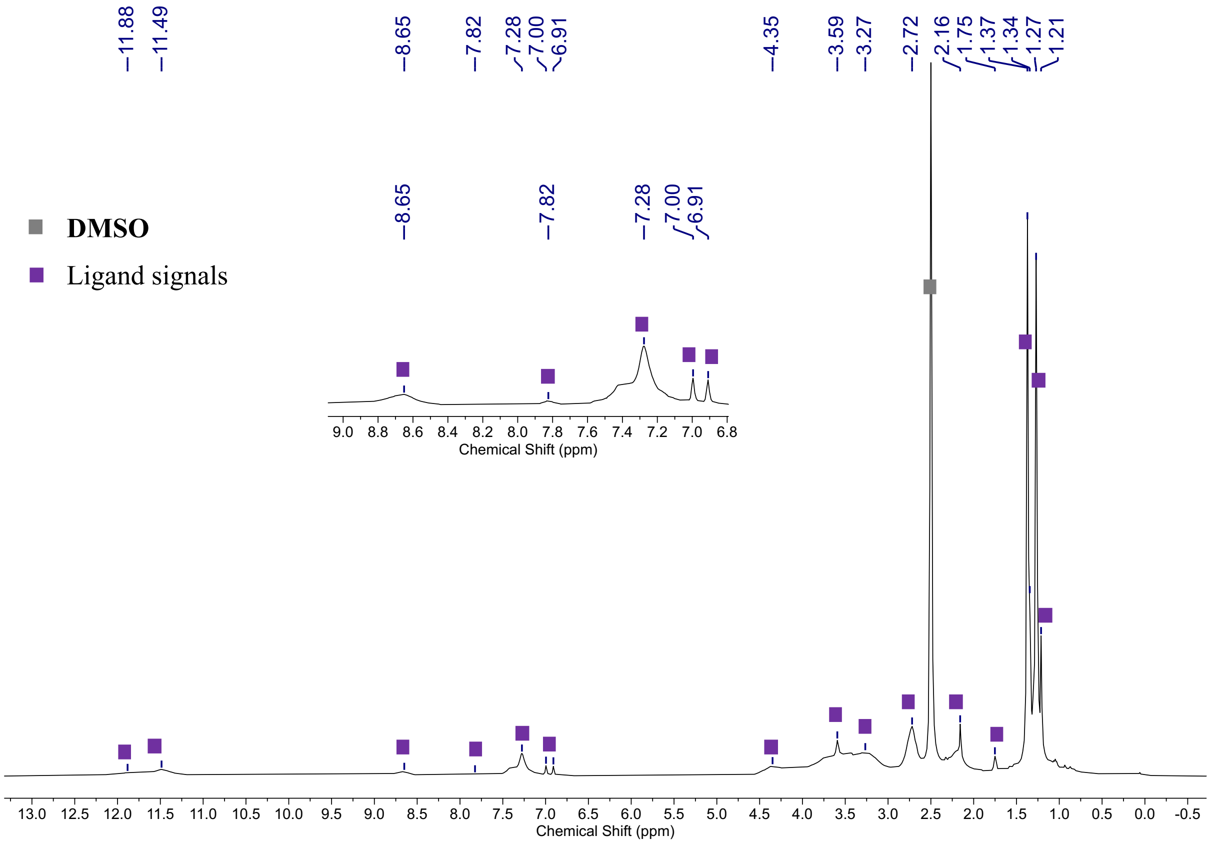

Figure S34. ^{1}H NMR spectrum (400 MHz, d_6-DMSO, 298 K) of the resulting reaction mixture obtained upon acid quenching (HCl in diethyl ether) of the reaction of **A** with KC_8 (2.2 equiv.) in diethyl ether at -40 °C for three days at under argon.

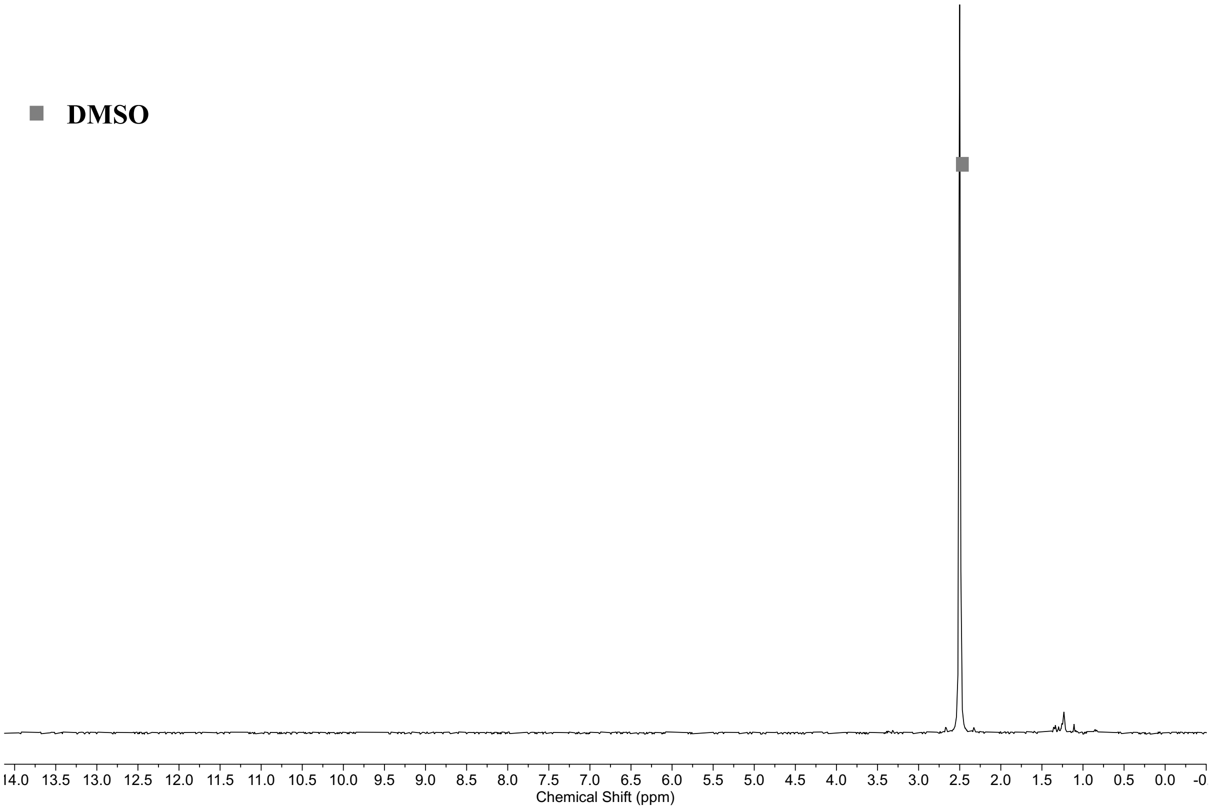

Figure S35. ^{1}H NMR spectrum (400 MHz, d_6-DMSO, 298 K) of the resulting reaction mixture obtained upon acid quenching (HCl in diethyl ether) of the reaction of **A** with KC_8 (2.2 equiv.) in hexane at r.t. for three days at under dinitrogen.

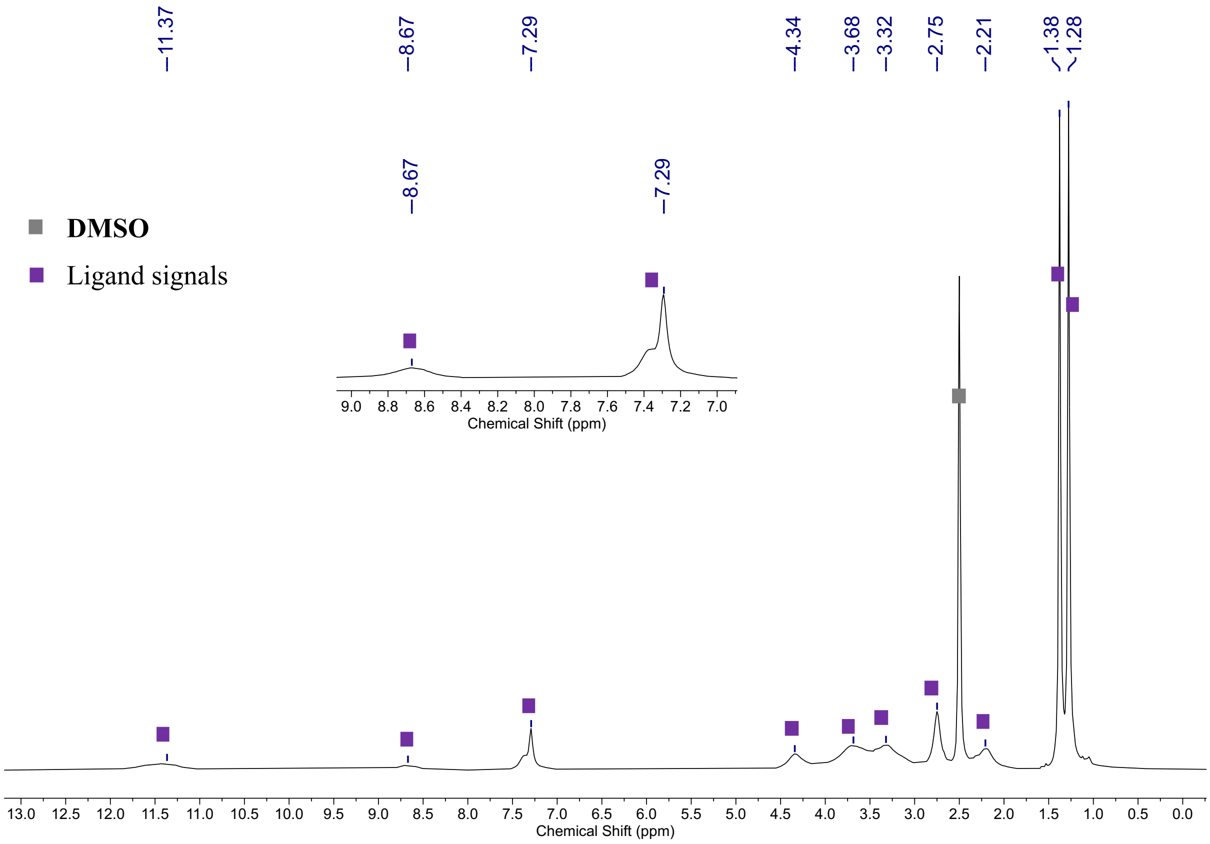

Figure S36. ^{1}H NMR spectrum (400 MHz, d_6-DMSO, 298 K) of the resulting reaction mixture obtained upon acid quenching (HCl in diethyl ether) of $H_2(^{tBu2}ArO)_2Me_2$-cyclam.

C. X-Ray Crystal Structure Determination Details

Suitable crystals were selected and mounted on various Rigaku diffractometers (XtaLAB Synergy R, DW system, HyPix-Arc 150 detector or SuperNova, Dual, Cu at home/near, AtlasS type detectors). The crystals were kept at a steady T = 140.00(10) K during data collection. Data were measured using ω scans with Cu Kα radiation. The diffraction patterns were indexed and the total number of runs and images were based on the strategy calculation from the program CrysAlisPro 1.171.44.91a.[6] The unit cells were refined using CrysAlisPro 1.171.44.91a.[6] Data reduction, scaling and absorption corrections were performed using CrysAlisPro 1.171.44.91a.[6]

The structures were solved with the ShelXT solution program using dual methods and by using Olex2 1.5 as the graphical interface.[7,8] The models were refined with ShelXL 2019/3 using full matrix least squares minimisation on F2.[9] All non-hydrogen atoms were refined anisotropically. Some hydrogen atom positions were calculated geometrically and refined using the riding model, but most hydrogen atoms were refined freely.

The structures displayed problems dealing with disorder (disordered ligands or solvent) or twinning. In particular, the crystal structure of compound **1** was treated for twinning (3 BASF parameters (0.212(2), 0.142(2), 0.124(2)) were refined during the last cycles of least-squares). The refinement of all structures needed several restraints to adjust the atomic parameters of the disordered moieties. The solvent was squeezed in the last stages of the refinement (using Olex2) in the case of compound **2** and **3**.

Table S1. Crystal data and structural refinement parameters for complexes [U(κ^6-{(^{tBu2}ArO)$_2$Me$_2$-cyclam})(OSi(O^{t}Bu)$_3$)].(hexane)$_{0.5}$ (**1.(hexane)$_{0.5}$**), [U(κ^7-{(^{tBu2}ArO)(^{tBu2}ArO-κ^2-N,C)Me$_2$-cyclam})].(THF)$_2$ (**2.(THF)$_2$**) and [{U(κ^5-{(^{tBu2}ArO)$_2$Me$_2$-cyclam})}$_2$(μ-η^6:η^6-benzene)] (**3.(toluene)$_{3.6}$**).

Compound	**1.(hexane)$_{0.5}$**	**2.(THF)$_2$**	**3.(toluene)$_{3.6}$**
Formula	$C_{57}H_{104}N_4O_6SiU$	$C_{50}H_{86}N_4O_4U$	$C_{115.2}H_{174.8}N_8O_4U_2$
Crystal Size (mm)	0.23×0.11×0.09	0.37×0.08×0.07	0.20×0.06×0.04
Crystal System	triclinic	monoclinic	monoclinic
Space Group	*P*-1	*P*2$_1$/*n*	*P2$_1$/c*
Volume (Å^3)	3015.04(16)	4980.6(3)	5387.5(4)
a (Å)	11.2069(3)	16.1897(4)	9.8734(7)
b (Å)	14.7053(5)	15.6587(6)	20.7159(8)
c (Å)	18.7776(6)	19.7929(8)	26.4929(3)
α (°)	84.700(3)	90	90
β (°)	80.165(2)	96.971(3)	96.162(3)
γ (°)	82.473(2)	90	90
Z	2	4	2
Formula Weight	1207.56	1045.25	2211.88
Density (g cm^{-3})	1.330	1.394	1.364
μ (mm^{-1})	8.138	9.514	8.801
F (000)	1256	2152	2276
Temperature (K)	199.99(10)	140.00(10)	140.00(10)
Total Reflections	13062	27066	57981
Unique Reflections	13062	9714	9157
R_{int}	.	0.0975	0.0734
R Indices [I >2σ(I)]	R_1 = 0.0434<br>wR_2 = 0.0977	R_1 = 0.0633<br>wR_2 = 0.1386	R_1 = 0.0412<br>wR_2 = 0.1039
Largest Diff. Peak and Hole (e A^{-3})	3.762 and -1.696	1.946 and -2.135	1.456 and -1.936
GooF	0.975	1.002	1.050
CCDC	2411216	2411217	2411215

D. EPR Spectroscopy Data

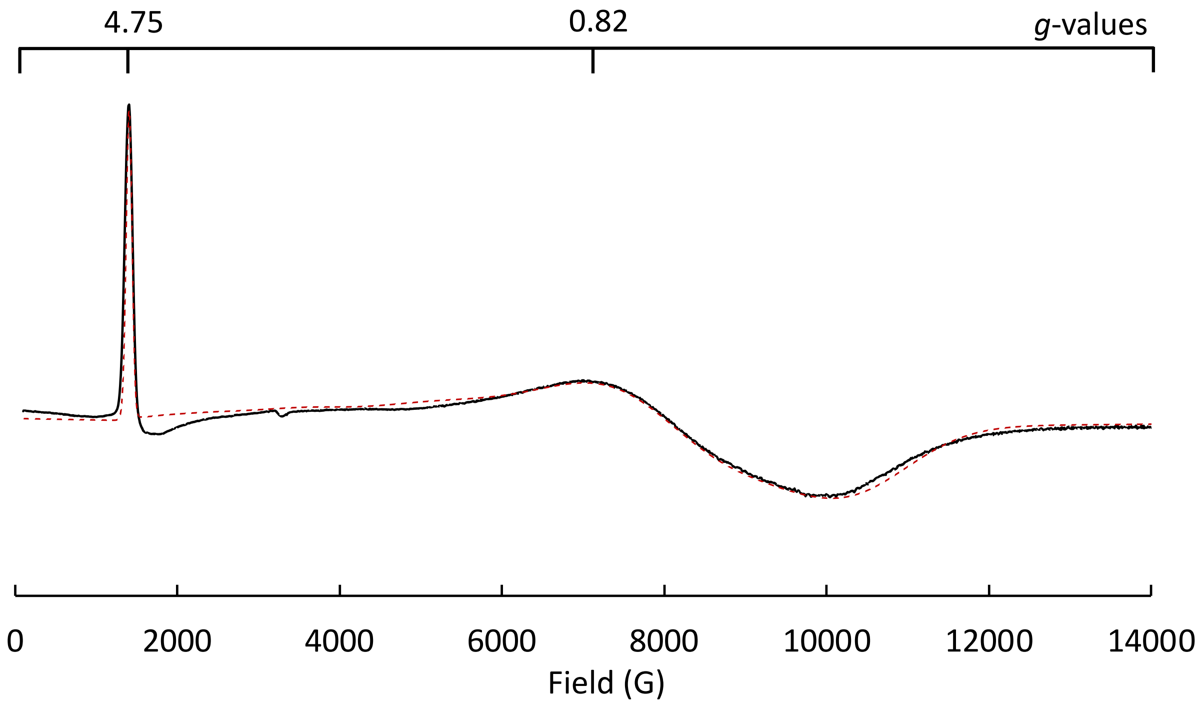

Figure S37. Solid state X-band (9.40 GHz) EPR spectrum of **A** (8.0 mg) at 6 K (*black line*, experiment; *red dashed line*, simulation). *g*-values: g_1 = 4.75; g_2 = 0.82; g_3 = 0.65.

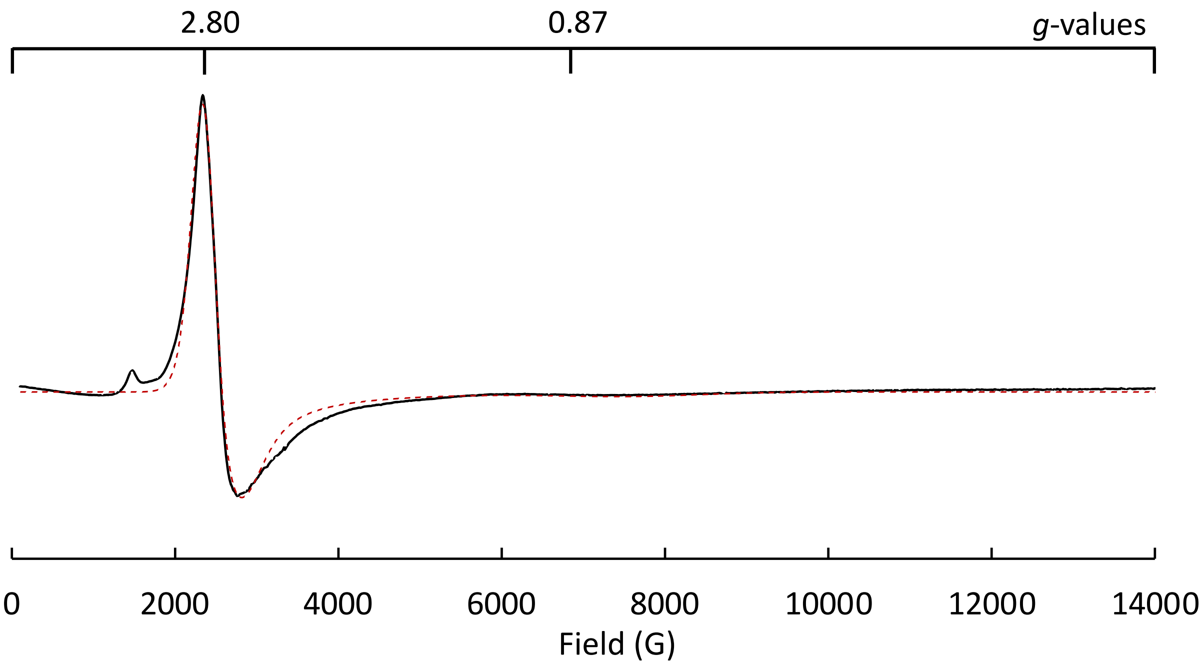

Figure S38. Solid state X-band (9.40 GHz) EPR spectrum of **3** (8.0 mg) at 6 K (*black line*, experiment; *red dashed line*, simulation). *g*-values: g_1 = 2.80; g_2 = 2.60; g_3 = 0.87.

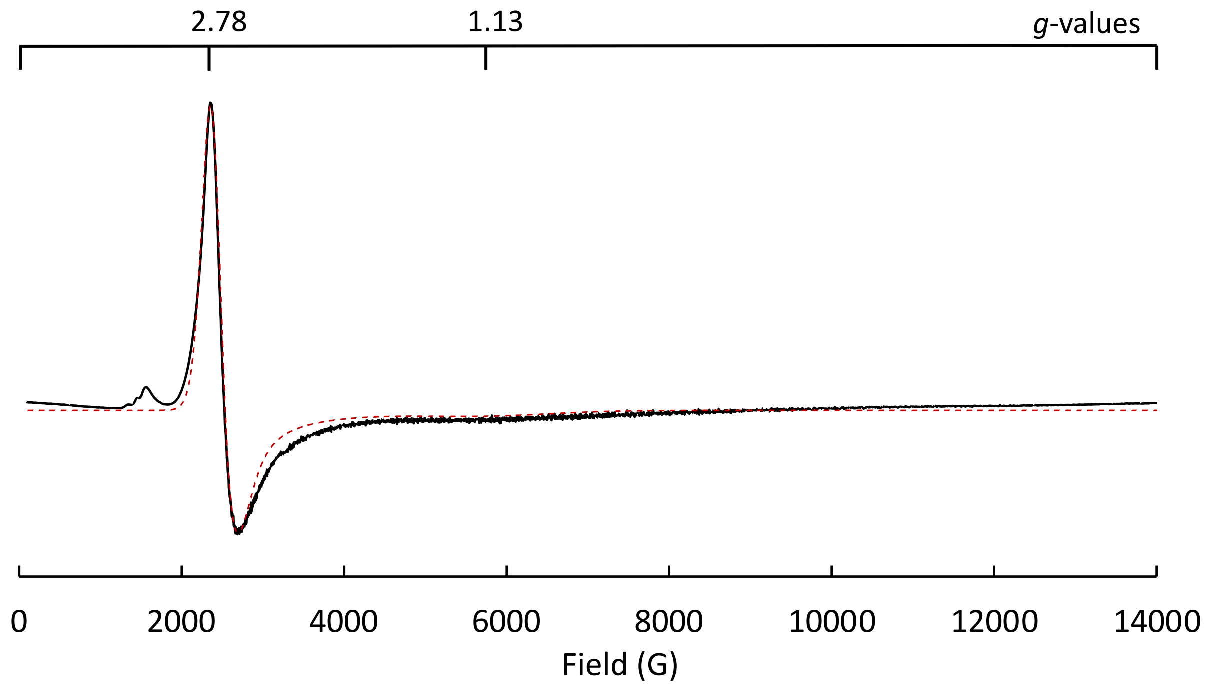

Figure S39. Frozen solution state X-band (9.40 GHz) EPR spectrum of **3** (10 mM in toluene) at 6 K (*black line*, experiment; *red dashed line*, simulation). *g*-values: g_1 = 2.78; g_2 = 2.65; g_3 = 1.13.

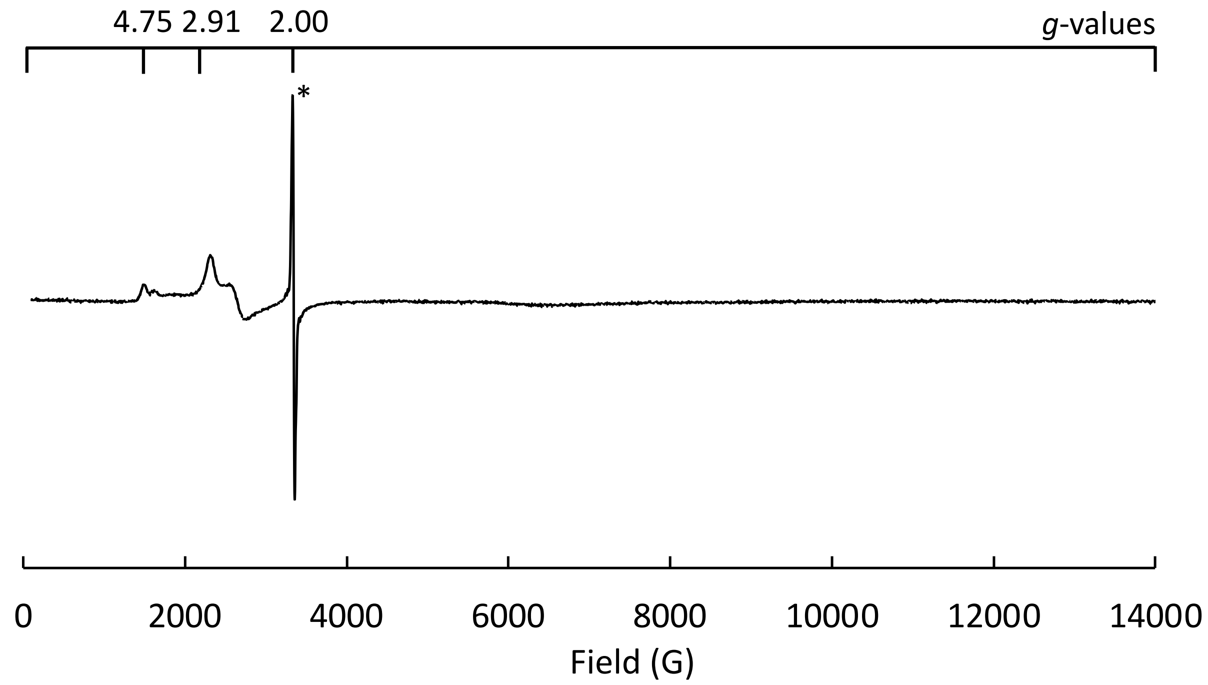

Figure S40. Solid state X-band (9.40 GHz) EPR spectrum of the precipitate obtained from **A** + 2.2 KC_8 reaction in hexane (which contains the intermediate species **B**) at 6 K (* corresponds to a signal arising from the unpaired electron from the excess KC_8 present).

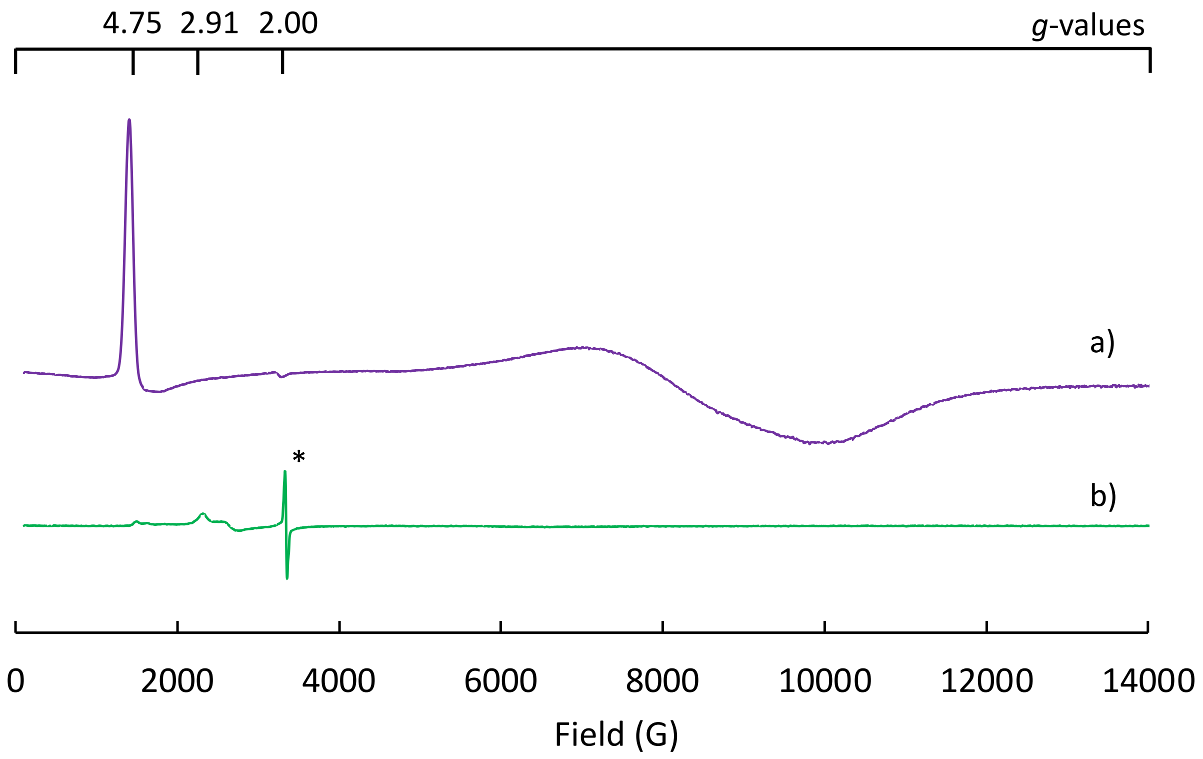

Figure S41. Solid state X-band (9.40 GHz) EPR spectrum of a) isolated **A** (8.0 mg) at 6 K and b) the precipitate obtained from **A** + 2.2 KC_8 reaction in hexane (which contains the intermediate species **B**) at 6 K (* corresponds to a signal arising from the unpaired electron from the excess KC_8 present).

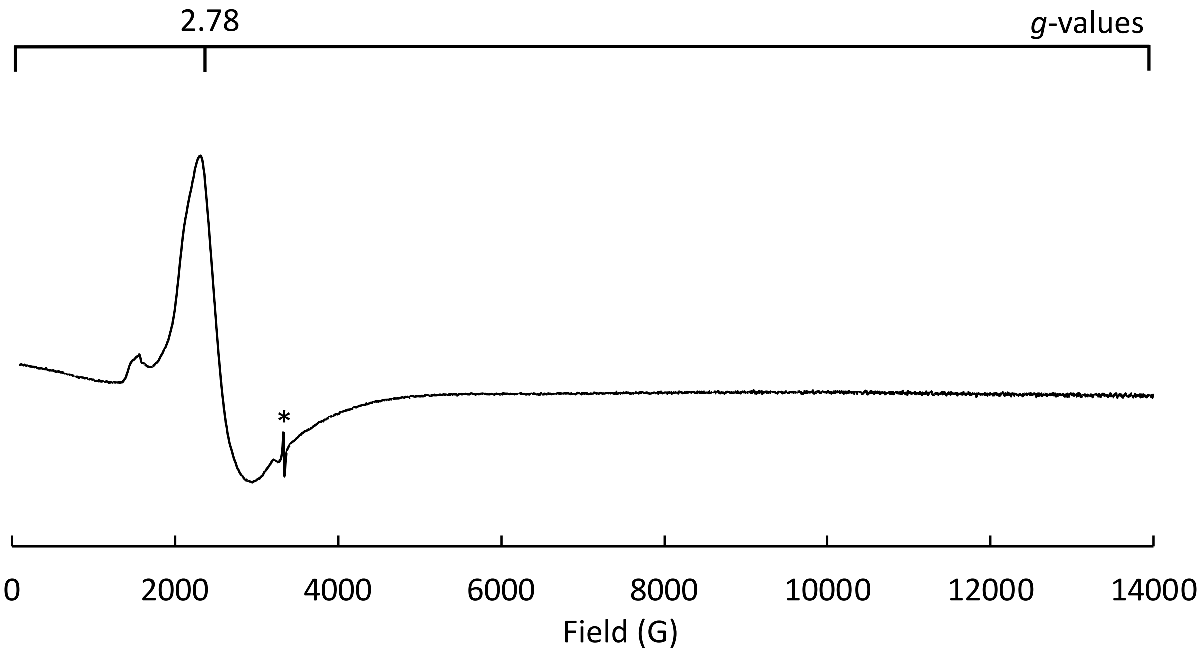

Figure S42. Frozen solution state (benzene) X-band (9.40 GHz) EPR spectrum of the resulting red brown solution obtained when the precipitate from the **A** + 2.2 KC_8 reaction in hexane is suspended in benzene and filtered (to remove graphite and excess KC_8)(* represents an organic radical impurity present in the sample).

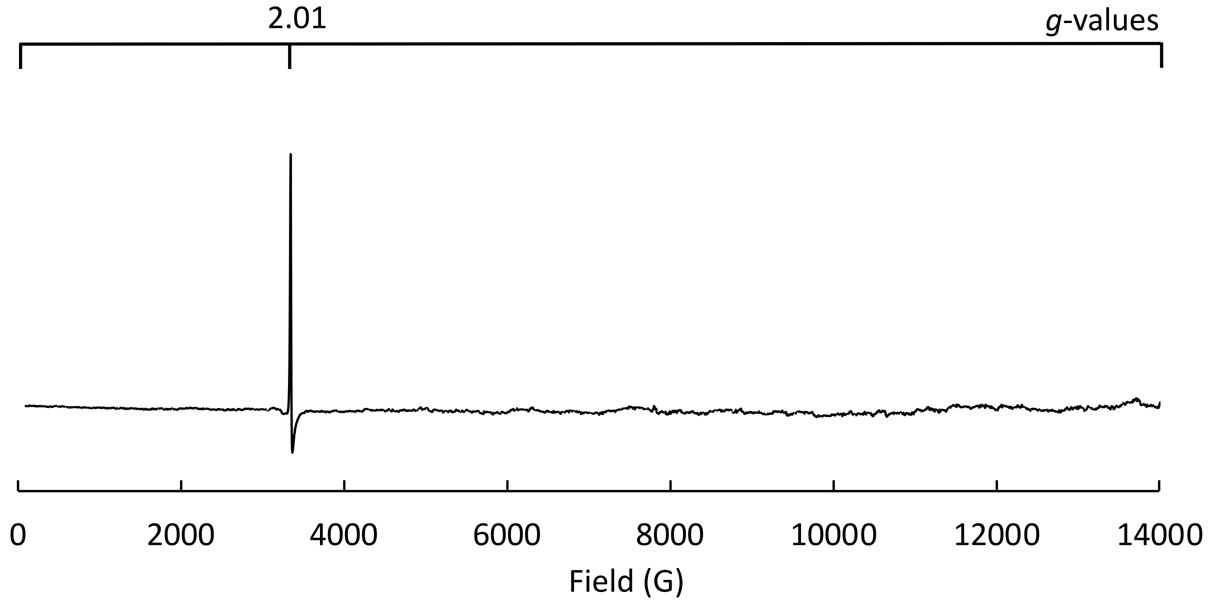

Figure S43. Solid state X-band (9.40 GHz) EPR spectrum of KC_8 (13.0 mg) at 6 K.

E. SQUID Magnetometry Data

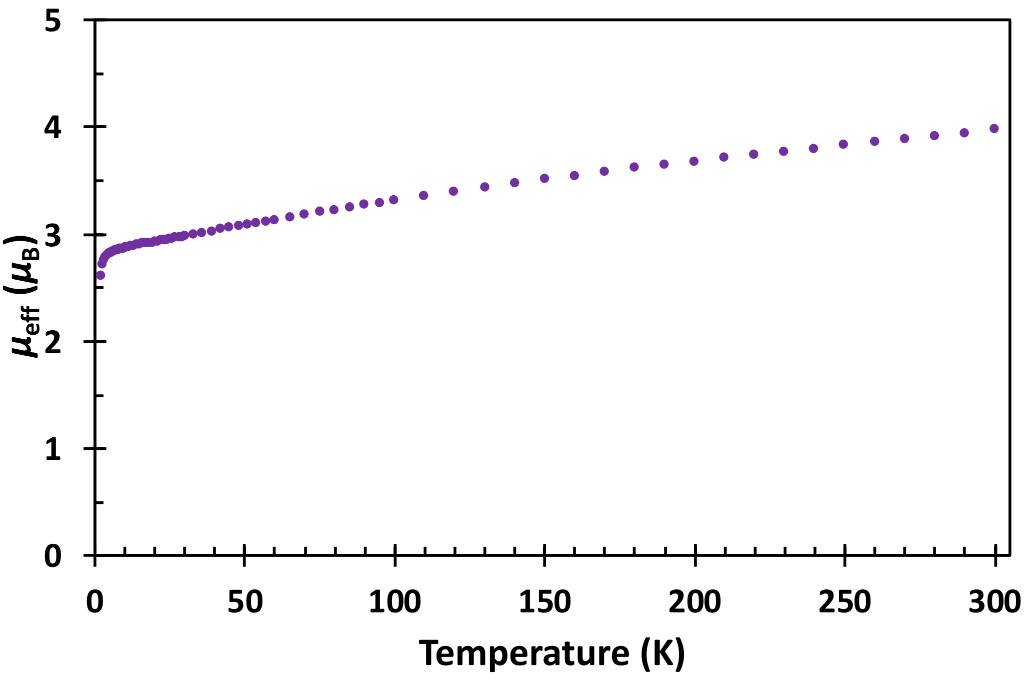

Figure S44. Temperature dependent SQUID magnetisation data for **A** plotted as a function of μ_{eff} vs. temperature, measured at 1 T.

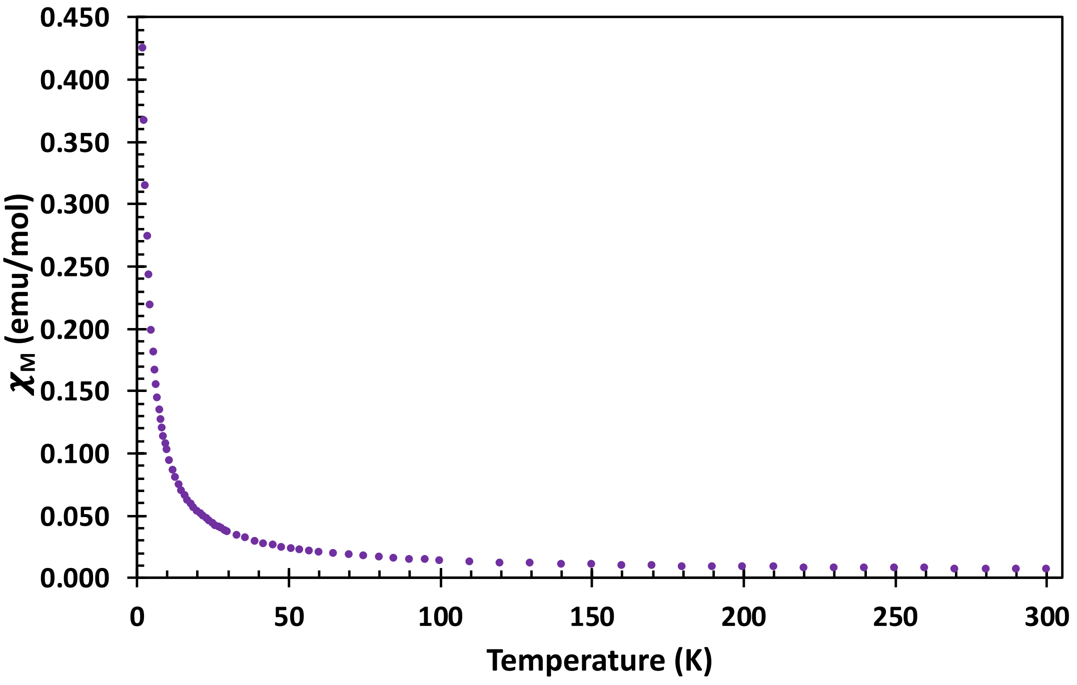

Figure S45. Temperature dependent SQUID magnetisation data for **A** plotted as a function of χ_M (molar magnetic susceptibility) vs. temperature, measured at 1 T.

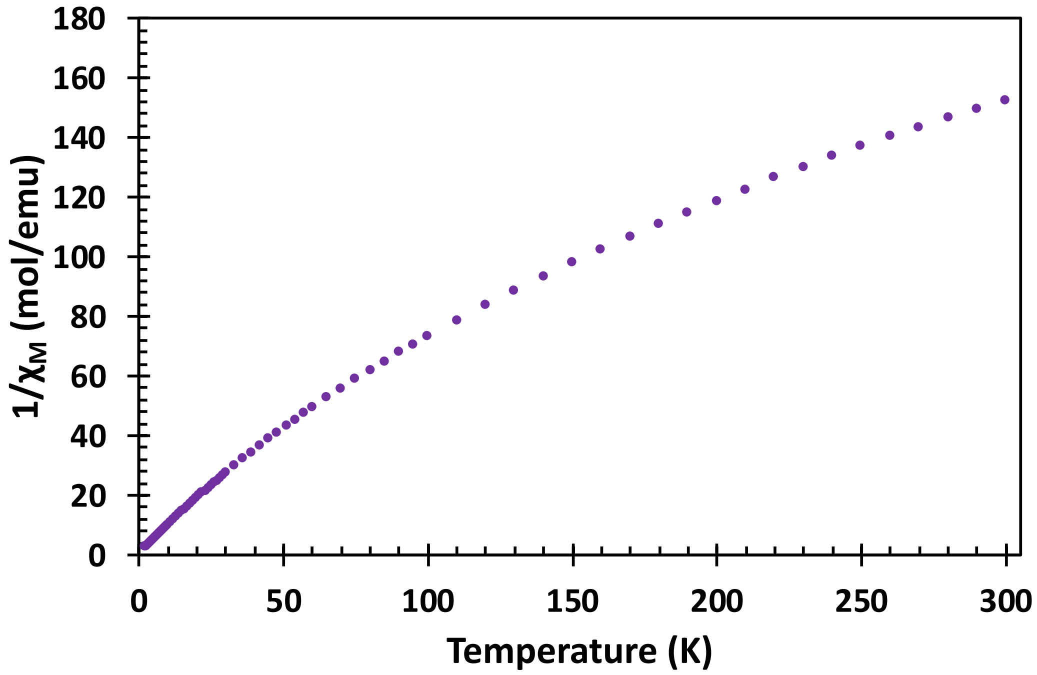

Figure S46. Temperature dependent SQUID magnetisation data for **A** plotted as a function of $1/\chi_M$ (where χ_M is the molar magnetic susceptibility) vs. temperature, measured at 1 T.

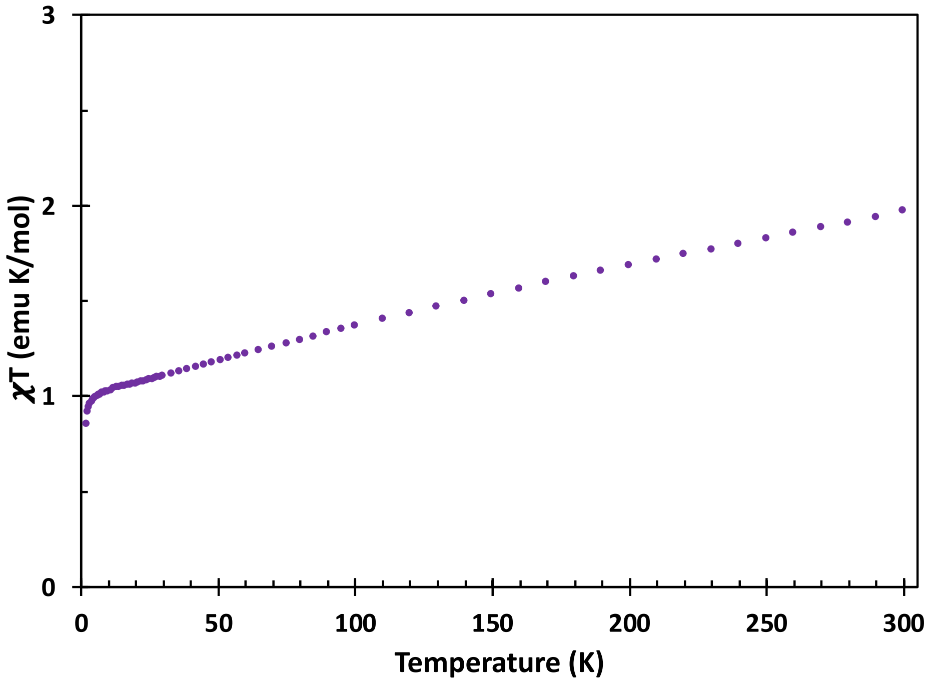

Figure S47. Temperature dependent SQUID magnetisation data for **A** plotted as a function of χT vs. temperature, measured at 1 T.

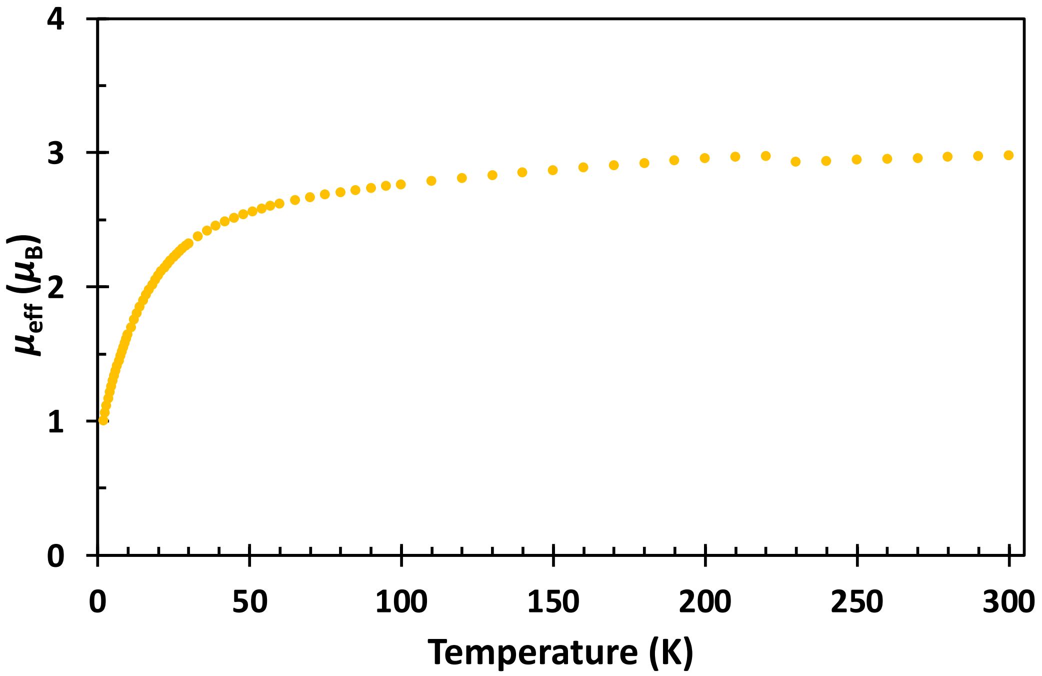

Figure S48. Temperature dependent SQUID magnetisation data for **2** plotted as a function of μ_{eff} vs. temperature, measured at 1 T.

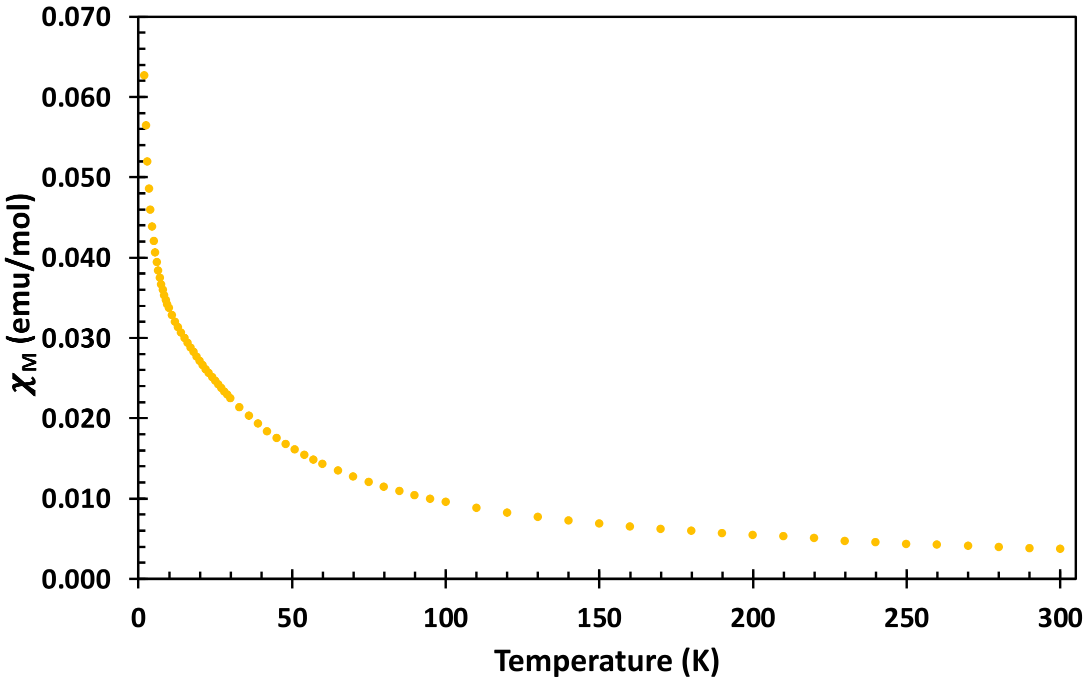

Figure S49. Temperature dependent SQUID magnetisation data for **2** plotted as a function of χ_M (molar magnetic susceptibility) vs. temperature, measured at 1 T.

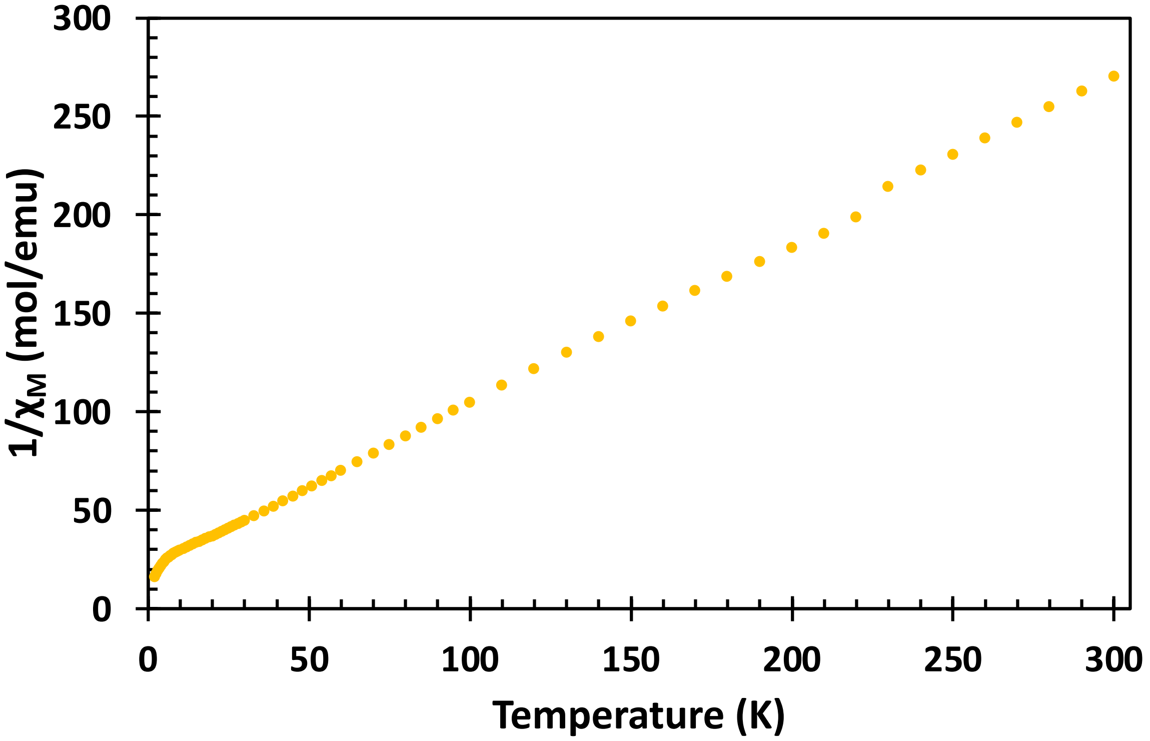

Figure S50. Temperature dependent SQUID magnetisation data for **2** plotted as a function of $1/\chi_M$ (where χ_M is the molar magnetic susceptibility) vs. temperature, measured at 1 T.

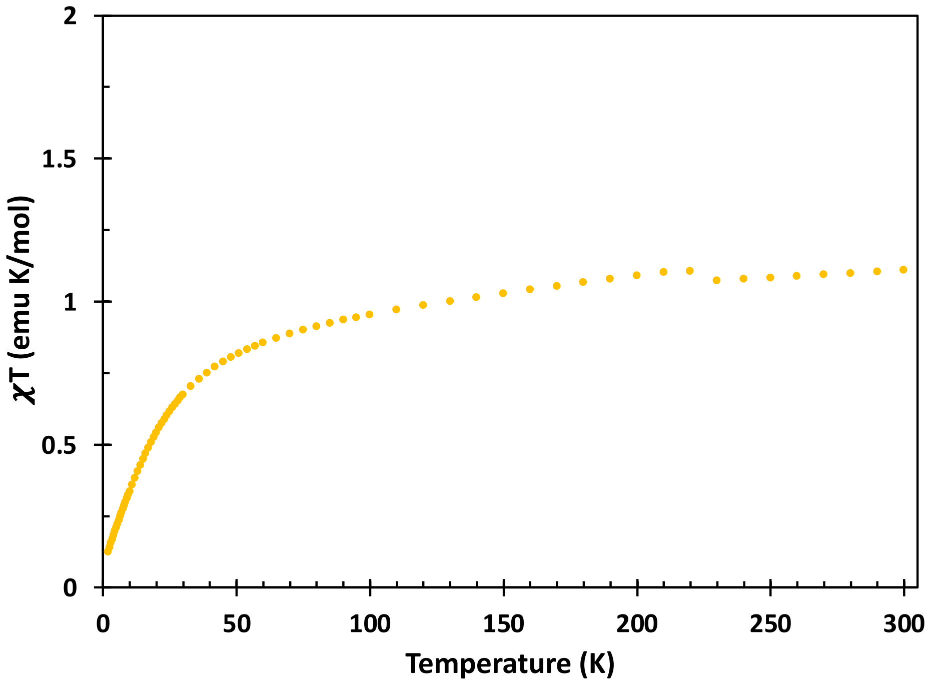

Figure S51. Temperature dependent SQUID magnetisation data (per complex) for **3** plotted as a function of χT vs. temperature, measured at 1 T.

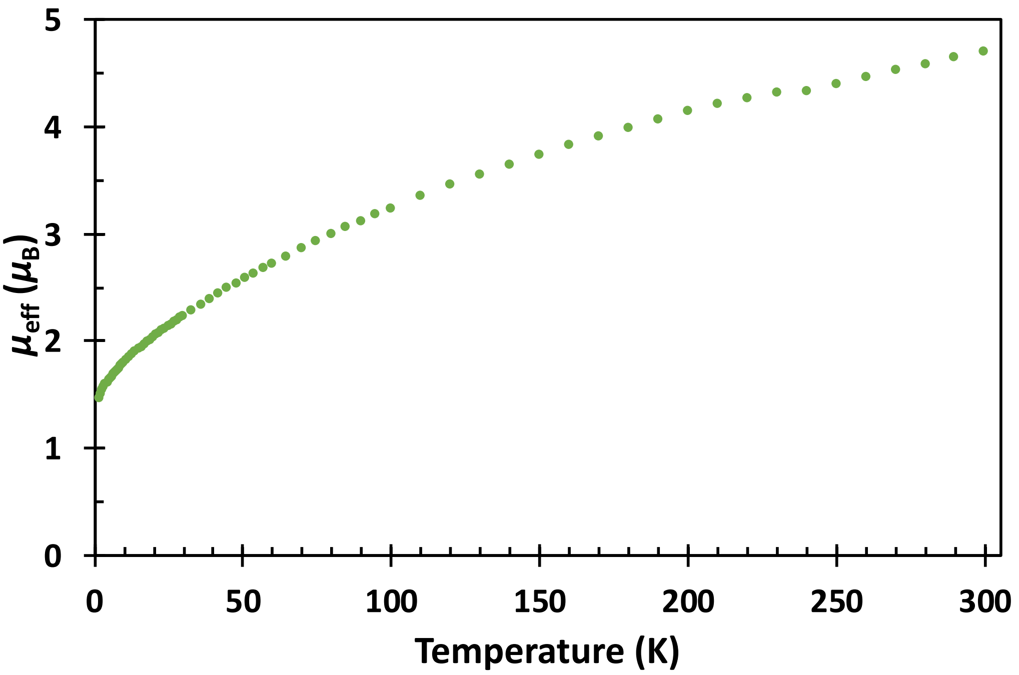

Figure S52. Temperature dependent SQUID magnetisation data (per complex) for **3** plotted as a function of μ_{eff} vs. temperature, measured at 1 T.

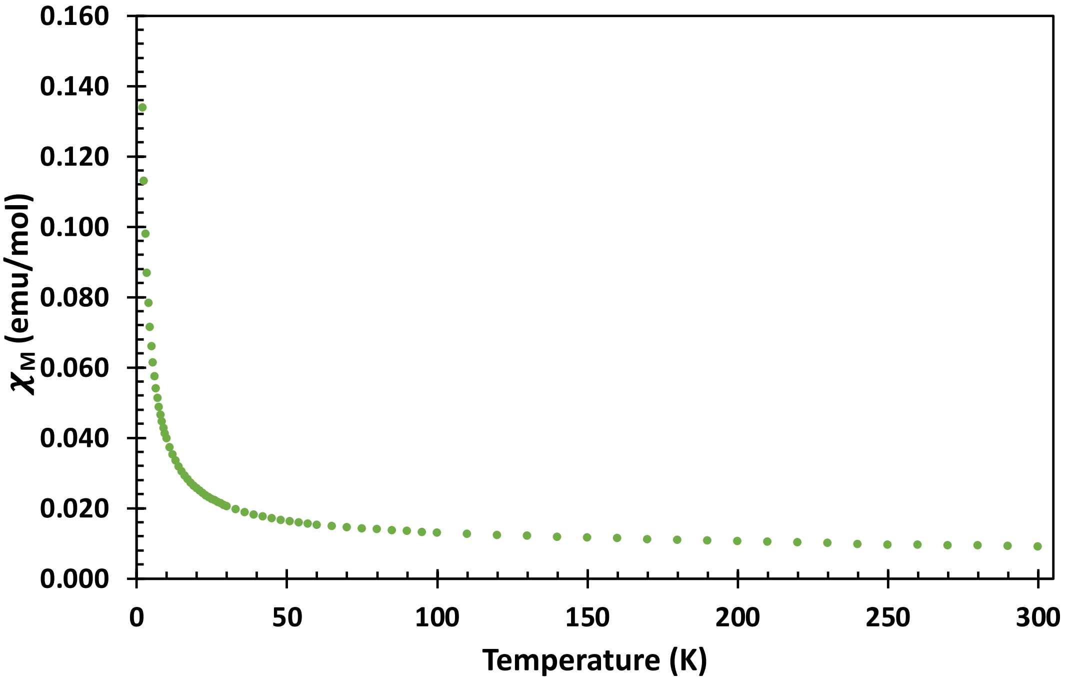

Figure S53. Temperature dependent SQUID magnetisation data (per complex) for **3** plotted as a function of χ_M (molar magnetic susceptibility) vs. temperature, measured at 1 T.

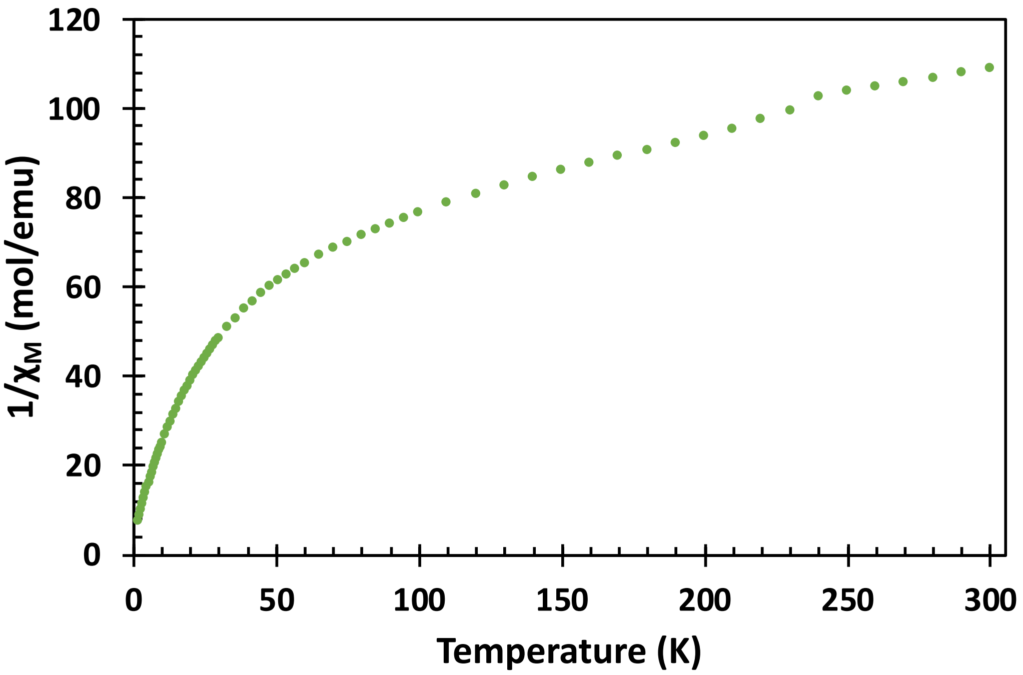

Figure S54. Temperature dependent SQUID magnetisation data (per complex) for **3** plotted as a function of $1/\chi_M$ (where χ_M is the molar magnetic susceptibility) vs. temperature, measured at 1 T.

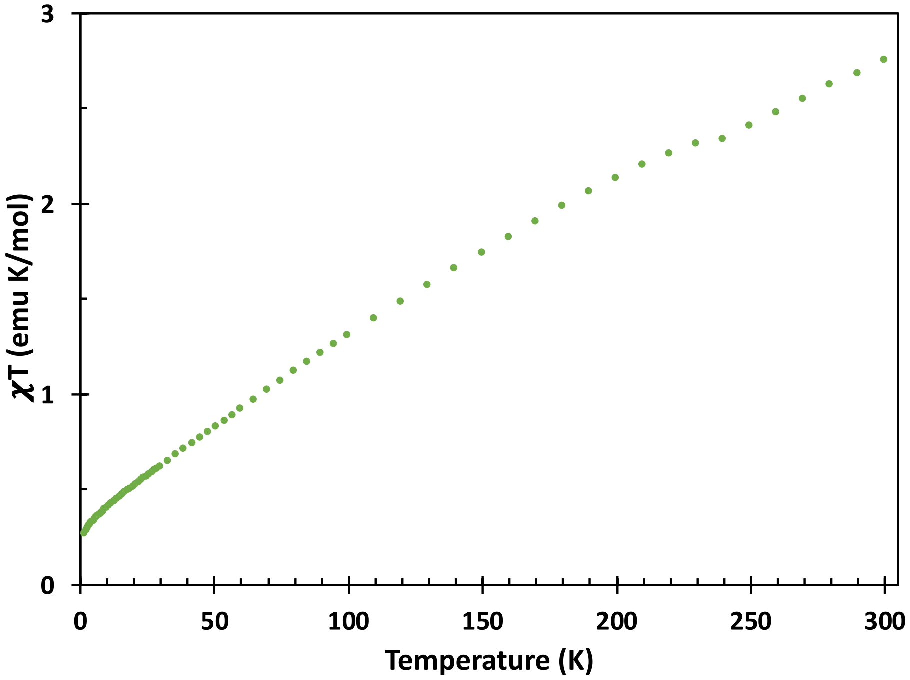

Figure S55. Temperature dependent SQUID magnetisation data (per complex) for **3** plotted as a function of χT vs. temperature, measured at 1 T.

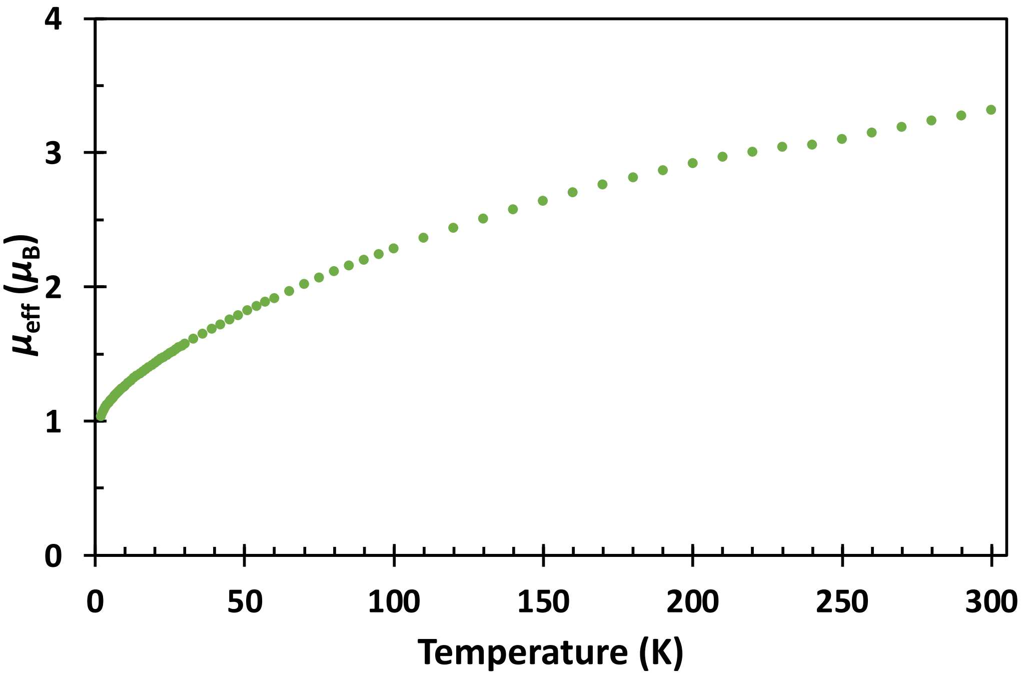

Figure S56. Temperature dependent SQUID magnetisation data (per U ion) for **3** plotted as a function of μ_{eff} vs. temperature, measured at 1 T.

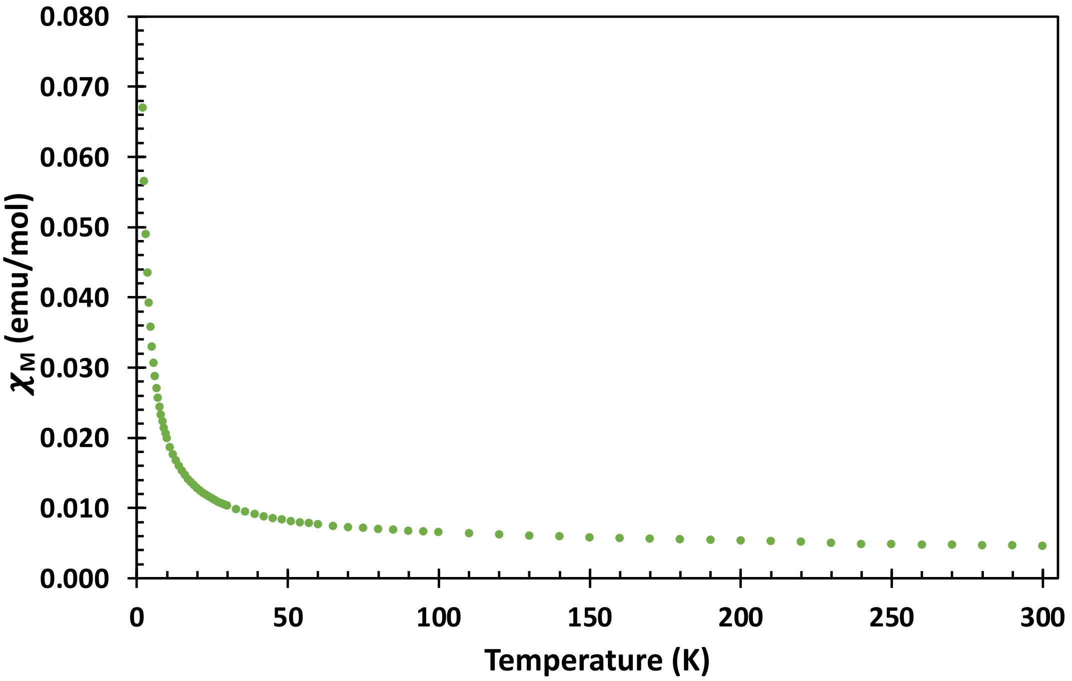

Figure S57. Temperature dependent SQUID magnetisation data (per U ion) for **3** plotted as a function of χ_M (molar magnetic susceptibility) vs. temperature, measured at 1 T.

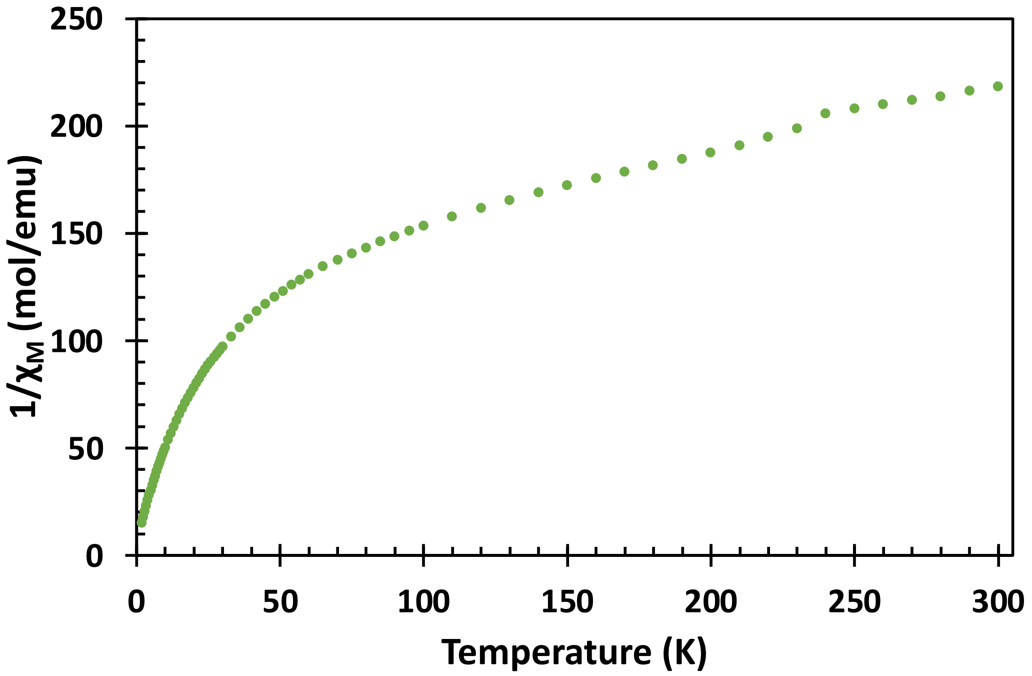

Figure S58. Temperature dependent SQUID magnetisation data (per U ion) for **3** plotted as a function of $1/\chi_M$ (where χ_M is the molar magnetic susceptibility) vs. temperature, measured at 1 T.

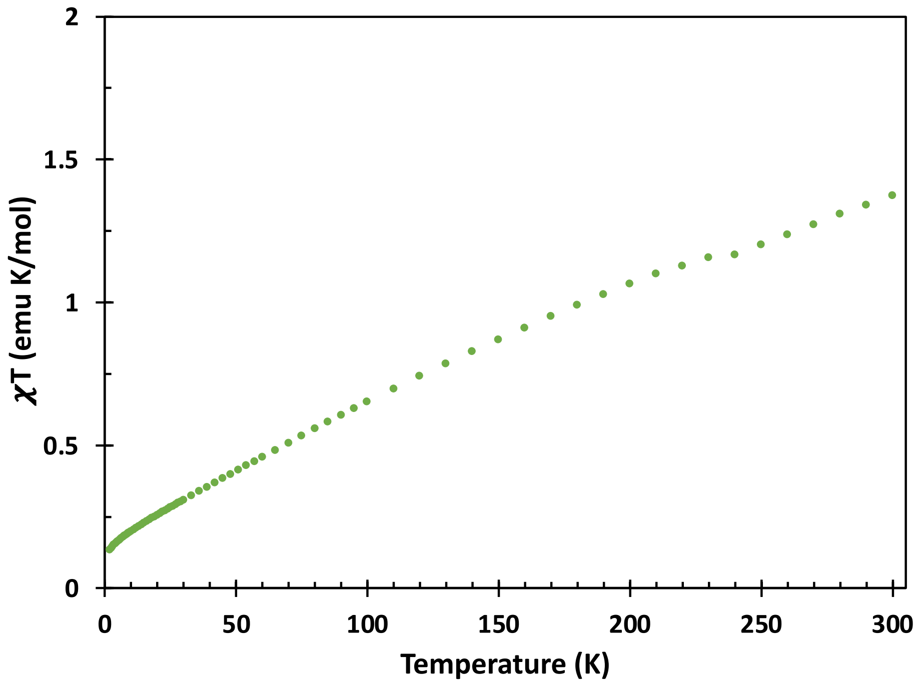

Figure S59. Temperature dependent SQUID magnetisation data (per U ion) for **3** plotted as a function of χT vs. temperature, measured at 1 T.

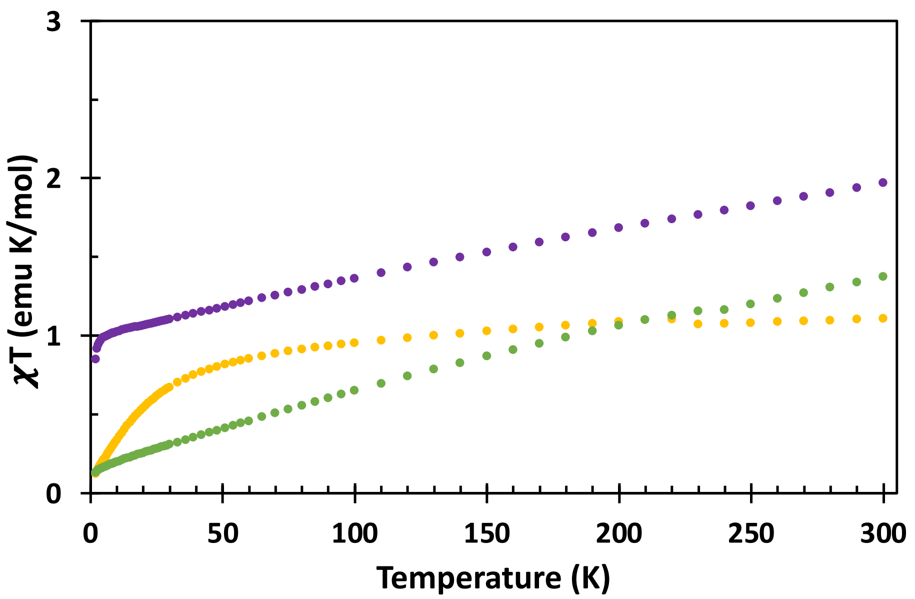

Figure S60. Temperature dependent SQUID magnetisation data (per U ion) for **A**, **2** and **3** plotted as a function of χT vs. temperature, measured at 1 T.

F. Cyclic Voltammetry Data

Table S2. Electrochemical redox potentials (in V vs Fc/Fc$^+$) for complex **A** including the E_{red}, E_{ox}, and $E_{1/2}$ values for events **I** and **II** in 0.1 M [NBu_4][BPh_4] electrolyte in THF.

	E_{red}	E_{ox}	$E_{1/2}$
Wave I	-1.77	-1.61	-1.69
Wave II	-0.39	-0.28	-0.34

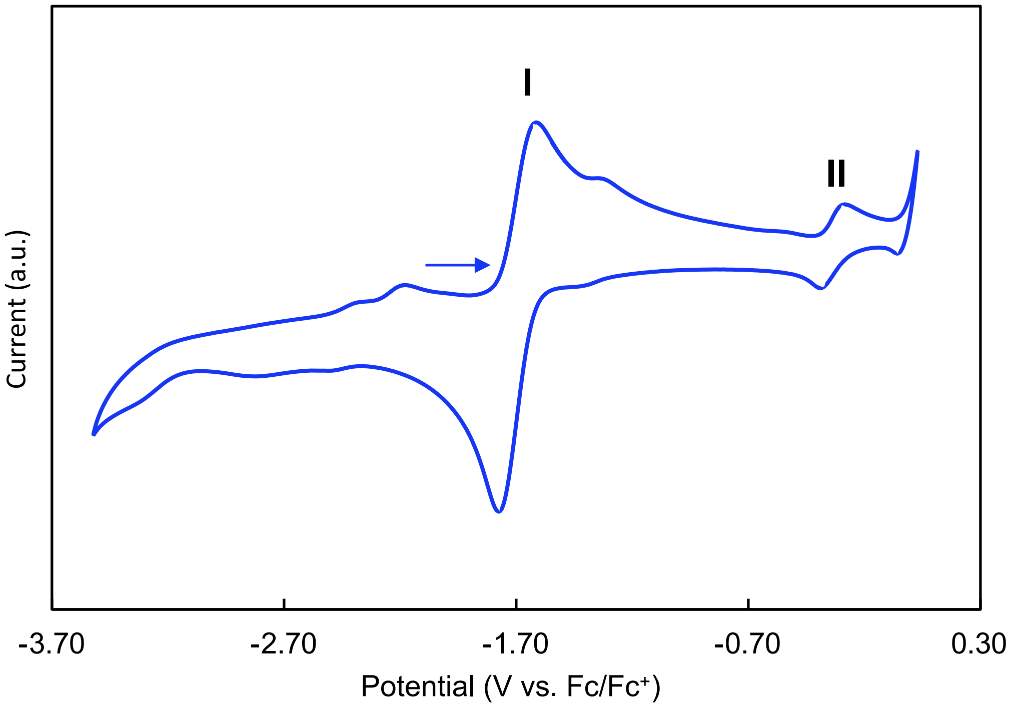

Figure S61. Cyclic voltammogram for complex **A** in THF (3 mM) with 0.1 M [NBu_4][BPh_4] as the supporting electrolyte, where arrows indicate the scan direction (Pt disk working electrode, 100 mV/s scan rate, referenced to the Fc/Fc$^+$ couple)(anodic scan).

G. UV/Vis/NIR Spectra

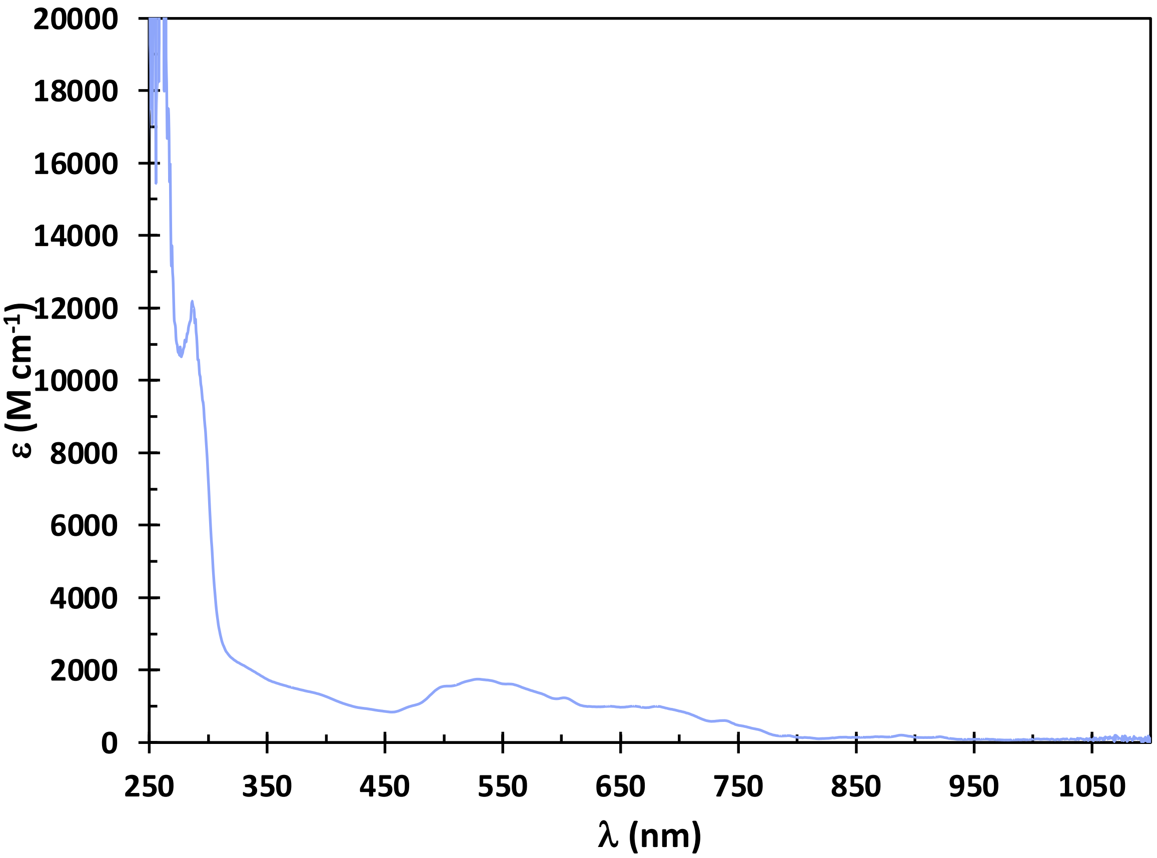

Figure S62. UV/Vis/NIR spectrum of **A** in THF (0.2 mM).

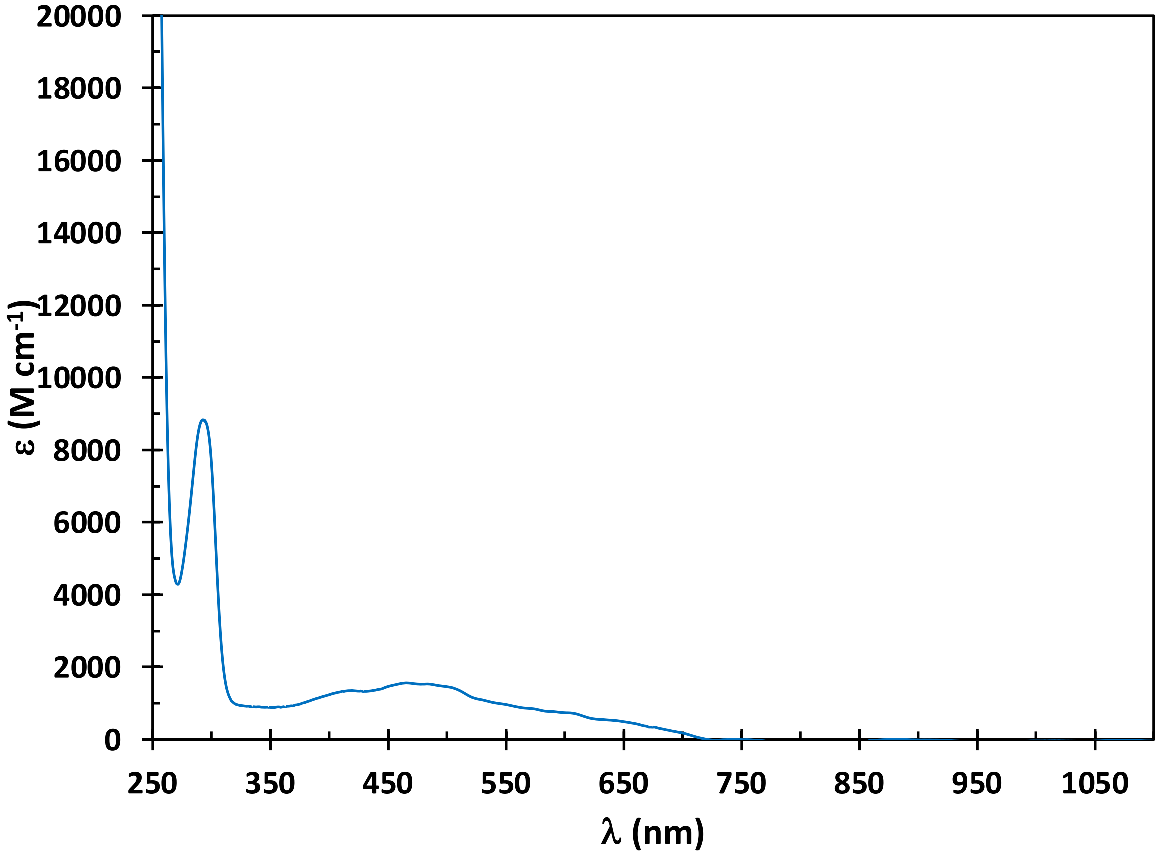

Figure S63. UV/Vis/NIR spectrum of **1** in THF (0.4 mM).

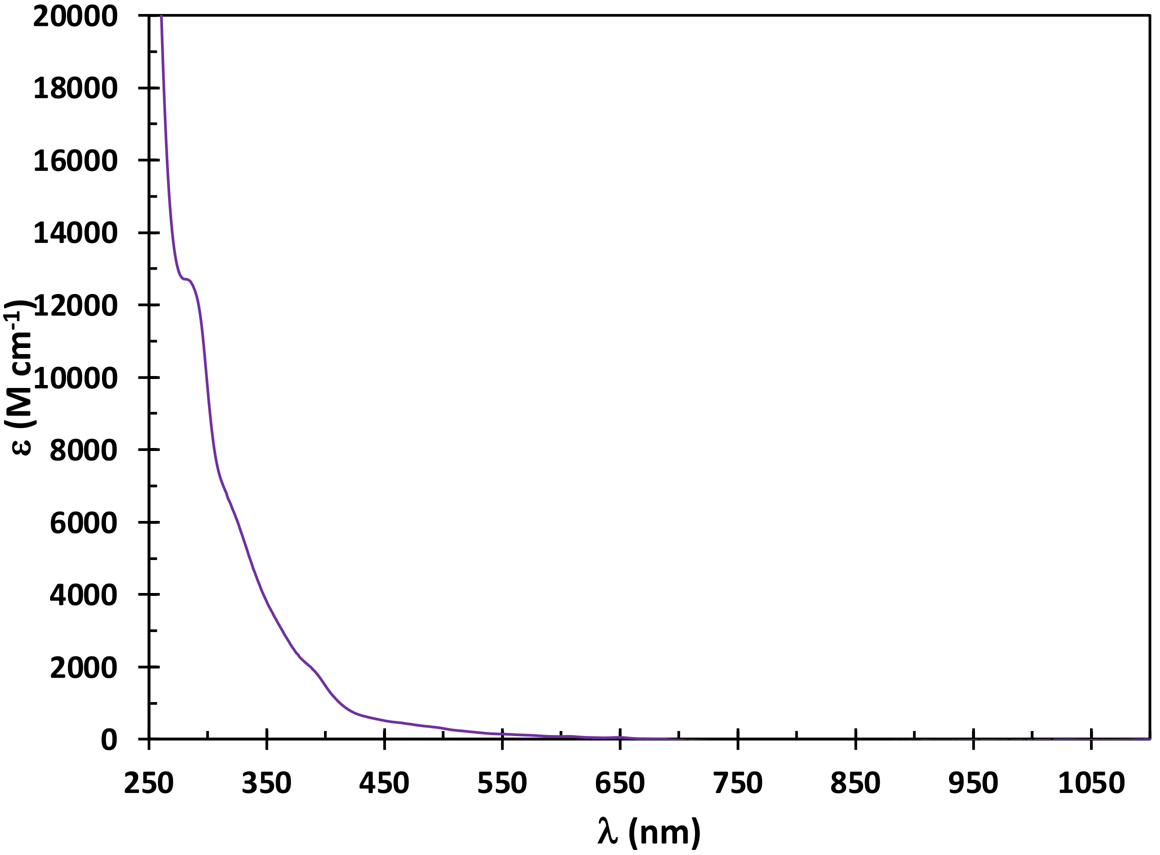

Figure S64. UV/Vis/NIR spectrum of **2** in THF (0.4 mM).

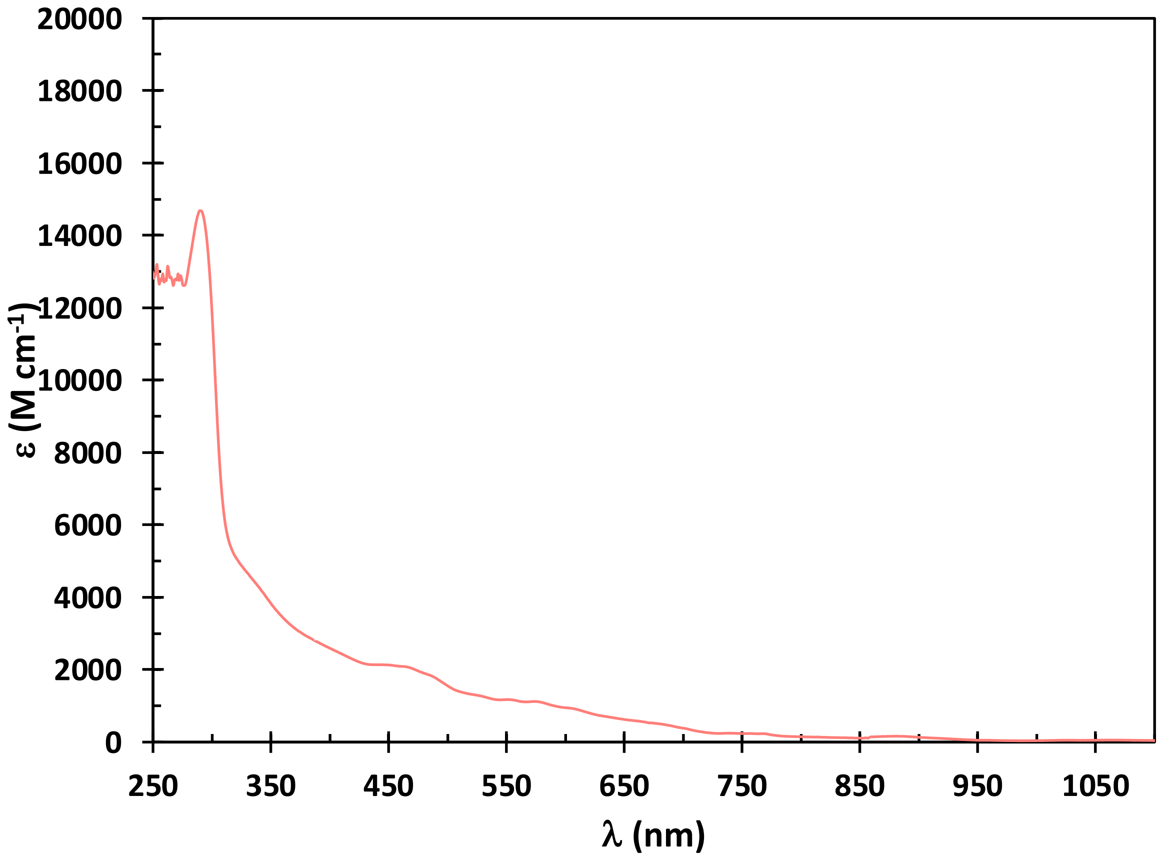

Figure S65. UV/Vis/NIR spectrum of **3** in THF (0.7 mM).

H. Computational Details

All the DFT calculations were performed using Gaussian09 suite of programs.[10] Hybrid DFT functional (B3PW91) along with SDD basis sets for U atoms and Pople (6-31G**) basis set were used for the rest of the atoms are used for geometry optimization and frequency calculations to obtain thermal corrections.[11–15]

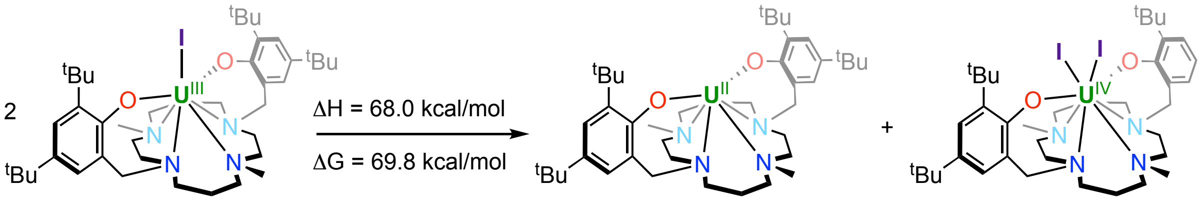

(**A**) (**B**) (**C**)

s = 3/2 s = 2 s = 1

Scheme S1. Thermodynamic parameters calculated for the disproportionation mechanism starting from complex **A**.

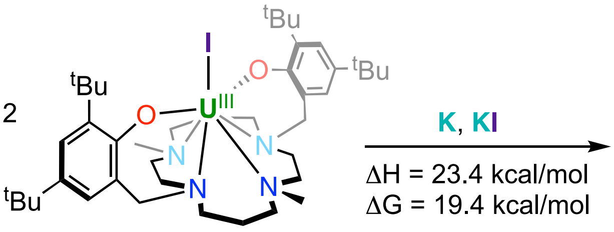

(**A**) (**B**)

s = 3/2 s = 2

Scheme S2. Thermodynamic parameters calculated for the reduction mechanism starting from complex **A**.

Table S3. Energetics of two spin states computed for $L^{cyclam}UC_6H_6UL^{cyclam}$ complex (**3**)

	ΔH (ΔG), kcal/mol
s=2	0.0
s=3	4.8 (2.5)

Table S4. Comparison of selected bond parameters (Å) between DFT optimized and X-ray structures

Atom labels	DFT		X-ray
	s=2	s=3	
U1-O2	2.20	2.21	2.21
U1-O3	2.21	2.22	2.22
U1-N5	2.93	2.91	2.91
U1-N6	2.77	2.81	2.74
U1-C13	2.67	2.77	2.60
U1-C36	2.52	2.72	2.57
U1-C43	2.56	2.81	2.60
U1-C126	2.55	2.79	2.62
U1-C128	2.72	2.92	2.66
U1-C130	2.64	2.74	2.62
C13-C36	1.45	1.47	1.44
C13-C43	1.45	1.39	1.42
C36-C130	1.45	1.45	1.43
C43-C128	1.46	1.45	1.43
C126-C128	1.46	1.47	1.44
C126-C130	1.45	1.39	1.42
U132-C13	2.56	2.73	2.62

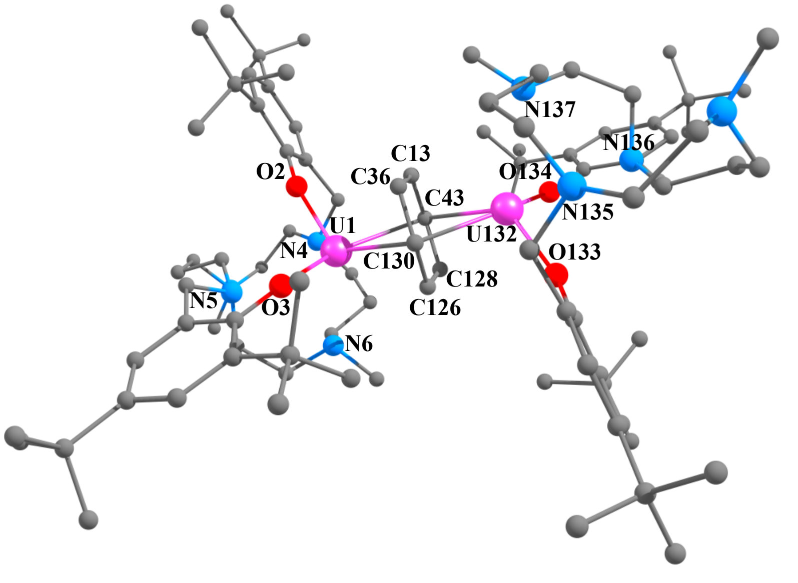

U132-C36	2.70	2.78	2.66	
U132-C43	2.67	2.71	2.62	
U132-C126	2.67	2.70	2.60	
U132-C128	2.50	2.62	2.57	
U132-C130	2.56	2.73	2.60	
U132-O133	2.22	2.22	2.21	
U132-O134	2.21	2.22	2.22	
U132-N136	2.95	2.95	2.91	
U132-N137	2.79	2.85	2.74	

Table S5. Computed natural charges for LUC_6H_6UL complex **3** (s=2)

Atom labels	Natural charges	
	s=2	s=3
U1	1.39949	1.42115
O2	-0.75391	-0.79448
O3	-0.75235	-0.79319
N5	-0.52265	-0.53882
N6	-0.52895	-0.54482
C13	-0.46035	-0.36383
C36	-0.48488	-0.55808
C43	-0.46653	-0.34051
C126	-0.46255	-0.36325
C128	-0.49215	-0.55767
C130	-0.45315	-0.34339
O133	-0.76312	-0.78140
O134	-0.75775	-0.77846
N136	-0.52347	-0.53349
N137	-0.52713	-0.53573

Table S6. Computed Wiberg bond index between selected atoms in LUC_6H_6UL complex **3** (s=2, s=3)

Atom labels	Wiberg bond index	Atom labels	Wiberg bond index	Atom labels	Wiberg bond index
U1	0.0000	U1	0.0000	U1	0.0000
O2	0.6985	O3	0.7005	N5	0.1709
	0.6144		0.6122		0.1702
Atom labels	Wiberg bond index	Atom labels	Wiberg bond index	Atom labels	Wiberg bond index
U1	0.0000	U1	0.0000	U1	0.0000
N6	0.1928	C13	0.2763	C36	0.3955
	0.1806		0.1761		0.2656
Atom labels	Wiberg bond index	Atom labels	Wiberg bond index	Atom labels	Wiberg bond index
U1	0.0000	U1	0.0000	U1	0.0000
C43	0.3957	C126	0.3987	C128	0.2430
	0.1748		0.1890		0.2000
Atom labels	Wiberg bond index	Atom labels	Wiberg bond index	Atom labels	Wiberg bond index
U1	0.0000	U132	0.0000	U132	0.0000
C130	0.2772	C13	0.3771	C36	0.2556
	0.1702		0.2515		0.2838
Atom labels	Wiberg bond index	Atom labels	Wiberg bond index	Atom labels	Wiberg bond index
U132	0.0000	U132	0.0000	U132	0.0000
C43	0.2579	C126	0.2634	C128	0.4263
	0.2141		0.2443		0.3560
Atom labels	Wiberg bond index	Atom labels	Wiberg bond index	Atom labels	Wiberg bond index
U132	0.0000	U132	0.0000	U132	0.0000

C130	0.3788	O133	0.6667	O134	0.6813
	0.2264		0.6291		0.6288
Atom labels	Wiberg bond index	Atom labels	Wiberg bond index		
U132	0.0000	U132	0.0000		
N136	0.1645	N137	0.1892		
	0.1685		0.1826		

Bonding orbitals (Alpha Molecular orbitals, AMO) from NBO analysis for ground state spin of LUC_6H_6UL complex **3** (s=2)
(0.99074) BD (1) U 1- O 2
(7.07%) 0.2658* U 1 s(16.26%)p 0.02(0.29%)d 3.19(51.85%) f 1.94(31.54%)g 0.00(0.05%)
(92.93%) 0.9640* O 2 s(63.78%)p 0.57(36.22%)d 0.00(0.00%)
(0.97313) BD (2) U 1- O 2
(7.21%) 0.2686* U 1 s(0.66%)p 0.71(0.47%)d71.60(47.35%) f77.85(51.48%)g 0.04(0.03%)
(92.79%) 0.9632* O 2 s(0.25%)p99.99(99.74%)d 0.06(0.01%)
(0.95673) BD (1) U 1- N 6
(5.64%) 0.2375* U 1 s(18.65%)p 0.05(0.86%)d 3.07(57.27%) f 1.25(23.22%)g 0.00(0.01%)
(94.36%) 0.9714* N 6 s(18.62%)p 4.37(81.36%)d 0.00(0.02%)
(0.94132) BD (1) C 13- C 36
(49.80%) 0.7057* C 13 s(33.58%)p 1.98(66.36%)d 0.00(0.07%)
(50.20%) 0.7085* C 36 s(33.23%)p 2.01(66.71%)d 0.00(0.07%)
(0.94176) BD (1) C 13- C 43
(50.14%) 0.7081* C 13 s(34.18%)p 1.92(65.75%)d 0.00(0.07%)
(49.86%) 0.7061* C 43 s(33.43%)p 1.99(66.50%)d 0.00(0.07%)
(0.94211) BD (1) C 36- C130
(50.18%) 0.7084* C 36 s(33.12%)p 2.02(66.81%)d 0.00(0.07%)
(49.82%) 0.7059* C130 s(33.75%)p 1.96(66.18%)d 0.00(0.07%)
(0.94187) BD (1) C 43- C128
(49.89%) 0.7063* C 43 s(33.52%)p 1.98(66.41%)d 0.00(0.07%)
(50.11%) 0.7079* C128 s(32.99%)p 2.03(66.94%)d 0.00(0.07%)
(0.94180) BD (1) C126- C128
(49.78%) 0.7056* C126 s(33.44%)p 1.99(66.49%)d 0.00(0.07%)
(50.22%) 0.7086* C128 s(33.31%)p 2.00(66.62%)d 0.00(0.07%)
(0.93895) BD (1) C126- C130
(50.00%) 0.7071* C126 s(33.77%)p 1.96(66.16%)d 0.00(0.07%)
(50.00%) 0.7071* C130 s(33.63%)p 1.97(66.30%)d 0.00(0.07%)
(0.99157) BD (1) U132- O133
(7.14%) 0.2672* U132 s(9.44%)p 0.03(0.25%)d 5.99(56.54%)f 3.57(33.72%)g 0.00(0.04%)
(92.86%) 0.9636* O133 s(63.73%)p 0.57(36.26%)d 0.00(0.00%)
(0.96789) BD (2) U132- O133
(6.09%) 0.2467* U132 s(0.16%)p 5.29(0.84%)d99.99(61.87%)f99.99(37.08%)g 0.27(0.04%)
(93.91%) 0.9691* O133 s(0.00%)p 1.00(99.98%)d 0.00(0.02%)
(0.99143) BD (1) U132- O134
(7.24%) 0.2691* U132 s(8.55%)p 0.02(0.18%)d 6.65(56.87%)f 4.02(34.35%)g 0.01(0.05%)
(92.76%) 0.9631* O134 s(63.97%)p 0.56(36.02%)d 0.00(0.01%)
(0.97543) BD (2) U132- O134
(7.06%) 0.2657* U132 s(0.42%)p 1.15(0.49%)d74.86(31.81%)f99.99(67.25%)g 0.06(0.03%)
(92.94%) 0.9641* O134 s(0.29%)p99.99(99.69%)d 0.08(0.02%)
(0.95597) BD (1) U132- N137
(5.57%) 0.2359* U132 s(13.31%)p 0.08(1.03%)d 4.61(61.40%)f 1.82(24.25%)g 0.00(0.01%)
(94.43%) 0.9718* N137 s(18.30%)p 4.46(81.68%)d 0.00(0.02%)

Bonding orbitals (Alpha Molecular orbitals, AMO) from NBO analysis for ground state spin of LUC6H6UL complex **3** (s=3)
(0.98768) BD (1) U 1- O 2
(6.03%) 0.2455* U 1 s(11.72%)p 0.03(0.38%)d 6.35(74.46%)f 1.14(13.37%)g 0.01(0.06%)
(93.97%) 0.9694* O 2 s(61.55%)p 0.62(38.44%)d 0.00(0.00%)
(0.97177) BD (2) U 1- O 2
(6.24%) 0.2497* U 1 s(0.32%)p 2.33(0.74%)d99.99(46.82%)f99.99(52.09%)g 0.09(0.03%)
(93.76%) 0.9683* O 2 s(0.09%)p99.99(99.89%)d 0.20(0.02%)

(0.99026) BD (1) U 1- O 3
(6.06%) 0.2461* U 1 s(13.98%)p 0.03(0.35%)d 5.20(72.63%)f 0.93(12.98%)g 0.00(0.06%)
(93.94%) 0.9692* O 3 s(64.08%)p 0.56(35.91%)d 0.00(0.01%)
(0.96979) BD (2) U 1- O 3
(5.79%) 0.2407* U 1 s(0.80%)p 0.55(0.44%)d47.65(38.33%)f75.09(60.40%)g 0.03(0.03%)
(94.21%) 0.9706* O 3 s(0.12%)p99.99(99.85%)d 0.24(0.03%)
(0.95751) BD (1) U 1- N 6
(5.14%) 0.2267* U 1 s(13.38%)p 0.05(0.66%)d 4.65(62.17%)f 1.78(23.78%)g 0.00(0.01%)
(94.86%) 0.9740* N 6 s(18.89%)p 4.29(81.09%)d 0.00(0.02%)
(0.97799) BD (1) U 1- U132
(69.37%) 0.8329* U 1 s(0.03%)p 0.56(0.01%)d99.99(5.52%)f99.99(94.43%)g 0.07(0.00%)
(30.63%) 0.5534* U132 s(0.00%)p 1.00(0.01%)d99.99(5.31%)f99.99(94.68%)g 0.06(0.00%)
(0.95994) BD (1) C 13- C 36
(50.45%) 0.7103* C 13 s(33.45%)p 1.99(66.49%)d 0.00(0.06%)
(49.55%) 0.7039* C 36 s(33.31%)p 2.00(66.63%)d 0.00(0.06%)
(0.96145) BD (1) C 13- C 43
(49.68%) 0.7049* C 13 s(35.82%)p 1.79(64.11%)d 0.00(0.07%)
(50.32%) 0.7094* C 43 s(35.75%)p 1.80(64.18%)d 0.00(0.06%)
(0.96093) BD (1) C 36- C130
(49.24%) 0.7017* C 36 s(33.71%)p 1.96(66.23%)d 0.00(0.06%)
(50.76%) 0.7125* C130 s(33.80%)p 1.96(66.14%)d 0.00(0.06%)
(0.96234) BD (1) C 43- C128
(50.97%) 0.7139* C 43 s(33.71%)p 1.96(66.23%)d 0.00(0.06%)
(49.03%) 0.7002* C128 s(32.52%)p 2.07(67.41%)d 0.00(0.07%)
(0.96087) BD (1) C126- C128
(50.65%) 0.7117* C126 s(33.38%)p 1.99(66.56%)d 0.00(0.06%)
(49.35%) 0.7025* C128 s(32.30%)p 2.09(67.64%)d 0.00(0.06%)
(0.96028) BD (1) C126- C130
(49.76%) 0.7054* C126 s(35.66%)p 1.80(64.28%)d 0.00(0.07%)
(50.24%) 0.7088* C130 s(35.65%)p 1.80(64.29%)d 0.00(0.06%)
(0.98815) BD (1) U132- O133
(6.39%) 0.2527* U132 s(12.21%)p 0.02(0.30%)d 5.84(71.31%)f 1.32(16.12%)g 0.00(0.05%)
(93.61%) 0.9675* O133 s(62.62%)p 0.60(37.38%)d 0.00(0.00%)
(0.97124) BD (2) U132- O133
(6.39%) 0.2528* U132 s(0.43%)p 1.53(0.66%)d99.99(49.69%)f99.99(49.19%)g 0.06(0.03%)
(93.61%) 0.9675* O133 s(0.10%)p99.99(99.88%)d 0.18(0.02%)
(0.99028) BD (1) U132- O134
(6.31%) 0.2512* U132 s(15.07%)p 0.02(0.30%)d 4.79(72.17%)f 0.82(12.40%)g 0.00(0.06%)
(93.69%) 0.9679* O134 s(64.11%)p 0.56(35.88%)d 0.00(0.01%)
(0.97057) BD (2) U132- O134
(5.89%) 0.2426* U132 s(0.76%)p 0.58(0.44%)d48.29(36.83%)f81.22(61.94%)g 0.03(0.03%)
(94.11%) 0.9701* O134 s(0.19%)p99.99(99.78%)d 0.15(0.03%)
(0.95674) BD (1) U132- N137
(5.28%) 0.2299* U132 s(14.28%)p 0.05(0.69%)d 4.19(59.84%)f 1.76(25.19%)g 0.00(0.01%)
(94.72%) 0.9732* N137 s(18.63%)p 4.37(81.35%)d 0.00(0.02%)

Table S7. NBO Second order perturbation analysis (AMO) for LUC_6H_6UL complex **3** (s=2)

Donor NBO	Acceptor NBO	E(2) kcal/mol
(0.92040) LP (1) O 3<br>s(62.91%)p 0.59(37.09%)d 0.00(0.00%)	(0.09933) LV (5) U 1<br>s(22.39%)p 0.01(0.27%)d 2.37(53.01%)f 1.08(24.28%)g 0.00(0.05%)	58.90
(0.90714) LP (2) O 3<br>s(0.19%)p99.99(99.78%)d 0.12(0.02%)	(0.11566) LV (3) U 1<br>s(0.03%)p11.03(0.31%)d99.99(35.42%)f99.99(64.22%)g 0.87(0.02%)	17.16
(0.91372) LP (1) N 5<br>s(16.47%)p 5.07(83.50%)d 0.00(0.03%)	(0.08581) LV (6) U 1<br>s(25.50%)p 0.02(0.62%)d 1.96(49.96%)f 0.94(23.91%)g 0.00(0.01%)	15.94
(0.61258) LP (1) C 13<br>s(0.38%)p99.99(99.55%)d 0.18(0.07%)	(0.28128) LV (1) U 1	6.14

	s(0.25%)p 0.91(0.22%)d99.99(31.43%)f99.99(68.09%)g 0.02(0.01%)	
(0.61258) LP (1) C 13<br>s(0.38%)p99.99(99.55%)d 0.18(0.07%)	(0.10748) LV (4) U 1<br>s(0.69%)p 1.19(0.82%)d99.99(76.80%)f31.47(21.68%)g 0.01(0.01%)	6.81
(0.62644) LP (1) C 36<br>s(1.83%)p53.76(98.11%)d 0.03(0.06%)	(0.23068) LV (2) U 1<br>s(0.36%)p 0.43(0.15%)d76.37(27.39%)f99.99(72.08%)g 0.04(0.01%)	20.05
(0.61494) LP (1) C 43<br>s(1.03%)p96.09(98.91%)d 0.06(0.06%)	(0.28128) LV (1) U 1<br>s(0.25%)p 0.91(0.22%)d99.99(31.43%)f99.99(68.09%)g 0.02(0.01%)	8.61
(0.61494) LP (1) C 43<br>s(1.03%)p96.09(98.91%)d 0.06(0.06%)	(0.23068) LV (2) U 1<br>s(0.36%)p 0.43(0.15%)d76.37(27.39%)f99.99(72.08%)g 0.04(0.01%)	11.44
(0.61534) LP (1) C126<br>s(0.81%)p99.99(99.12%)d 0.08(0.07%)	(0.28128) LV (1) U 1<br>s(0.25%)p 0.91(0.22%)d99.99(31.43%)f99.99(68.09%)g 0.02(0.01%)	27.21
(0.60890) LP (1) C130<br>s(0.37%)p99.99(99.56%)d 0.19(0.07%)	(0.23068) LV (2) U 1<br>s(0.36%)p 0.43(0.15%)d76.37(27.39%)f99.99(72.08%)g 0.04(0.01%)	4.23
(0.94132) BD (1) C 13- C 36<br>(49.80%) 0.7057* C 13 s(33.58%)p 1.98(66.36%)d 0.00(0.07%)<br>(50.20%) 0.7085* C 36 s(33.23%)p 2.01(66.71%)d 0.00(0.07%)	(0.10748) LV (4) U 1<br>s(0.69%)p 1.19(0.82%)d99.99(76.80%)f31.47(21.68%)g 0.01(0.01%)	7.57
(0.94176) BD (1) C 13- C 43<br>(50.14%) 0.7081* C 13 s(34.18%)p 1.92(65.75%)d 0.00(0.07%)<br>(49.86%) 0.7061* C 43 s(33.43%)p 1.99(66.50%)d 0.00(0.07%)	(0.08079) LV (7) U 1<br>s(0.89%)p 0.49(0.43%)d24.46(21.76%)f86.41(76.89%)g 0.03(0.02%)	8.33
(0.94211) BD (1) C 36- C130<br>(50.18%) 0.7084* C 36 s(33.12%)p 2.02(66.81%)d 0.00(0.07%)<br>(49.82%) 0.7059* C130 s(33.75%)p 1.96(66.18%)d 0.00(0.07%)	(0.06202) LV (8) U 1<br>s(3.23%)p 0.28(0.91%)d11.94(38.59%)f17.70(57.21%)g 0.02(0.06%)	9.19
(0.94187) BD (1) C 43- C128<br>(49.89%) 0.7063* C 43 s(33.52%)p 1.98(66.41%)d 0.00(0.07%)<br>(50.11%) 0.7079* C128 s(32.99%)p 2.03(66.94%)d 0.00(0.07%)	(0.08581) LV (6) U 1<br>s(25.50%)p 0.02(0.62%)d 1.96(49.96%)f 0.94(23.91%)g 0.00(0.01%)	5.36
(0.94180) BD (1) C126- C128<br>(49.78%) 0.7056* C126 s(33.44%)p 1.99(66.49%)d 0.00(0.07%)<br>(50.22%) 0.7086* C128 s(33.31%)p 2.00(66.62%)d 0.00(0.07%)	(0.10748) LV (4) U 1<br>s(0.69%)p 1.19(0.82%)d99.99(76.80%)f31.47(21.68%)g 0.01(0.01%)	7.98
(0.93895) BD (1) C126- C130<br>(50.00%) 0.7071* C126 s(33.77%)p 1.96(66.16%)d 0.00(0.07%)<br>(50.00%) 0.7071* C130 s(33.63%)p 1.97(66.30%)d 0.00(0.07%)	(0.06202) LV (8) U 1<br>s(3.23%)p 0.28(0.91%)d11.94(38.59%)f17.70(57.21%)g 0.02(0.06%)	8.33
(0.61258) LP (1) C 13<br>s(0.38%)p99.99(99.55%)d 0.18(0.07%)	(0.27612) LV (1) U132<br>s(0.21%)p 0.28(0.06%)d99.99(29.42%)f99.99(70.31%)g 0.03(0.01%)	18.25
(0.61258) LP (1) C 13<br>s(0.38%)p99.99(99.55%)d 0.18(0.07%)	(0.21766) LV (2) U132<br>s(2.14%)p 0.23(0.49%)d14.04(30.11%)f31.35(67.24%)g 0.01(0.02%)	5.38
(0.61258) LP (1) C 13<br>s(0.38%)p99.99(99.55%)d 0.18(0.07%)	(0.10407) LV (3) U132<br>s(0.70%)p 1.22(0.86%)d99.99(81.93%)f23.47(16.50%)g 0.01(0.01%)	5.55
(0.62644) LP (1) C 36<br>s(1.83%)p53.76(98.11%)d 0.03(0.06%)	(0.27612) LV (1) U132	5.27

	s(0.21%)p 0.28(0.06%)d99.99(29.42%)f99.99(70.31%)g 0.03(0.01%)	
(0.61494) LP (1) C 43<br>s(1.03%)p96.09(98.91%)d 0.06(0.06%)	(0.21766) LV (2) U132<br>s(2.14%)p 0.23(0.49%)d14.04(30.11%)f31.35(67.24%)g 0.01(0.02%)	7.13
(0.61534) LP (1) C126<br>s(0.81%)p99.99(99.12%)d 0.08(0.07%)	(0.10407) LV (3) U132<br>s(0.70%)p 1.22(0.86%)d99.99(81.93%)f23.47(16.50%)g 0.01(0.01%)	7.19
(0.63105) LP (1) C128<br>s(2.08%)p47.02(97.86%)d 0.03(0.06%)	(0.27612) LV (1) U132<br>s(0.21%)p 0.28(0.06%)d99.99(29.42%)f99.99(70.31%)g 0.03(0.01%)	24.01
(0.60890) LP (1) C130<br>s(0.37%)p99.99(99.56%)d 0.19(0.07%)	(0.21766) LV (2) U132<br>s(2.14%)p 0.23(0.49%)d14.04(30.11%)f31.35(67.24%)g 0.01(0.02%)	14.10
(0.94132) BD (1) C 13- C 36<br>(49.80%) 0.7057* C 13 s(33.58%)p 1.98(66.36%)d 0.00(0.07%)<br>(50.20%) 0.7085* C 36 s(33.23%)p 2.01(66.71%)d 0.00(0.07%)	(0.10407) LV (3) U132<br>s(0.70%)p 1.22(0.86%)d99.99(81.93%)f23.47(16.50%)g 0.01(0.01%)	6.10
(0.94176) BD (1) C 13- C 43<br>(50.14%) 0.7081* C 13 s(34.18%)p 1.92(65.75%)d 0.00(0.07%)<br>(49.86%) 0.7061* C 43 s(33.43%)p 1.99(66.50%)d 0.00(0.07%)	(0.10407) LV (3) U132<br>s(0.70%)p 1.22(0.86%)d99.99(81.93%)f23.47(16.50%)g 0.01(0.01%)	5.97
(0.94211) BD (1) C 36- C130<br>(50.18%) 0.7084* C 36 s(33.12%)p 2.02(66.81%)d 0.00(0.07%)<br>(49.82%) 0.7059* C130 s(33.75%)p 1.96(66.18%)d 0.00(0.07%)	(0.09344) LV (4) U132<br>s(25.03%)p 0.03(0.71%)d 1.75(43.91%)f 1.21(30.35%)g 0.00(0.01%)	6.95
(0.94180) BD (1) C126- C128<br>(49.78%) 0.7056* C126 s(33.44%)p 1.99(66.49%)d 0.00(0.07%)<br>(50.22%) 0.7086* C128 s(33.31%)p 2.00(66.62%)d 0.00(0.07%)	(0.10407) LV (3) U132<br>s(0.70%)p 1.22(0.86%)d99.99(81.93%)f23.47(16.50%)g 0.01(0.01%)	7.31
(0.94180) BD (1) C126- C128<br>(49.78%) 0.7056* C126 s(33.44%)p 1.99(66.49%)d 0.00(0.07%)<br>(50.22%) 0.7086* C128 s(33.31%)p 2.00(66.62%)d 0.00(0.07%)	(0.09344) LV (4) U132<br>s(25.03%)p 0.03(0.71%)d 1.75(43.91%)f 1.21(30.35%)g 0.00(0.01%)	9.39
(0.93895) BD (1) C126- C130<br>(50.00%) 0.7071* C126 s(33.77%)p 1.96(66.16%)d 0.00(0.07%)<br>(50.00%) 0.7071* C130 s(33.63%)p 1.97(66.30%)d 0.00(0.07%)	(0.10407) LV (3) U132<br>s(0.70%)p 1.22(0.86%)d99.99(81.93%)f23.47(16.50%)g 0.01(0.01%)	7.19
(0.93895) BD (1) C126- C130<br>(50.00%) 0.7071* C126 s(33.77%)p 1.96(66.16%)d 0.00(0.07%)<br>(50.00%) 0.7071* C130 s(33.63%)p 1.97(66.30%)d 0.00(0.07%)	(0.09344) LV (4) U132<br>s(25.03%)p 0.03(0.71%)d 1.75(43.91%)f 1.21(30.35%)g 0.00(0.01%)	9.81
(0.91437) LP (1) N136<br>s(15.25%)p 5.56(84.72%)d 0.00(0.03%)	(0.05768) LV (5) U132<br>s(18.80%)p 0.01(0.21%)d 0.73(13.74%)f 3.58(67.24%)g 0.00(0.02%)	7.63

Table S8. NBO Second order perturbation analysis (AMO) for LUC_6H_6UL complex **3** (s=3)

Donor NBO	Acceptor NBO	E(2) kcal/mol
(0.91402) LP (1) N 5<br>s(19.41%)p 4.15(80.56%)d 0.00(0.02%)	(0.07547) LV (3) U 1<br>s(40.75%)p 0.02(0.72%)d 1.04(42.18%)f 0.40(16.34%)g 0.00(0.01%)	7.57
(0.91402) LP (1) N 5	(0.05585) LV (4) U 1	8.57

s(19.41%)p 4.15(80.56%)d 0.00(0.02%)	s(7.96%)p 0.02(0.14%)d 0.96(7.66%)f10.58(<br>84.22%)g 0.00(0.02%)	
(0.91423) LP (1) N136<br>s(18.10%)p 4.52(81.88%)d 0.00(0.03%)	(0.08336) LV (3) U132<br>s(35.55%)p 0.02(0.64%)d 1.24(44.13%)f 0.55(<br>19.67%)g 0.00(0.01%)	7.00
(0.91423) LP (1) N136<br>s(18.10%)p 4.52(81.88%)d 0.00(0.03%)	(0.05564) LV (4) U132<br>s(11.35%)p 0.00(0.05%)d 0.95(10.78%)f 6.86(<br>77.80%)g 0.00(0.02%)	8.42
(0.56669) LP (1) C 13<br>s(0.16%)p99.99(99.78%)d 0.38(0.06%)	(0.09356) LV (2) U 1<br>s(3.51%)p 0.17(0.58%)d22.91(80.43%)f 4.41(<br>15.47%)g 0.00(0.00%)	5.53
(0.56669) LP (1) C 13<br>s(0.16%)p99.99(99.78%)d 0.38(0.06%)	(0.09685) LV (2) U132<br>s(1.38%)p 0.53(0.73%)d51.42(70.88%)f19.59(<br>27.00%)g 0.00(0.01%)	3.04
(0.65887) LP (1) C 36<br>s(0.02%)p99.99(99.94%)d 2.37(0.04%)	(0.14994) LV (1) U 1<br>s(0.25%)p 0.33(0.08%)d99.22(25.19%)f99.99(<br>74.47%)g 0.03(0.01%)	11.54
(0.65887) LP (1) C 36<br>s(0.02%)p99.99(99.94%)d 2.37(0.04%)	(0.23573) LV (1) U132<br>s(0.07%)p 0.75(0.05%)d99.99(32.89%)f99.99(<br>66.99%)g 0.09(0.01%)	6.47
(0.60887) LP (1) C 43<br>s(0.29%)p99.99(99.66%)d 0.18(0.05%)	(0.04438) LV (5) U 1<br>s(0.41%)p 2.71(1.12%)d80.54(33.31%)f99.99(<br>65.09%)g 0.14(0.06%)	3.54
(0.60887) LP (1) C 43<br>s(0.29%)p99.99(99.66%)d 0.18(0.05%)	(0.04763) LV (5) U132<br>s(1.36%)p 0.92(1.25%)d25.45(34.66%)f46.01(<br>62.67%)g 0.05(0.06%)	7.29
(0.56512) LP (1) C126<br>s(0.42%)p99.99(99.52%)d 0.15(0.06%)	(0.23573) LV (1) U132<br>s(0.07%)p 0.75(0.05%)d99.99(32.89%)f99.99(<br>66.99%)g 0.09(0.01%)	3.07
(0.56512) LP (1) C126<br>s(0.42%)p99.99(99.52%)d 0.15(0.06%)	(0.09685) LV (2) U132<br>s(1.38%)p 0.53(0.73%)d51.42(70.88%)f19.59(<br>27.00%)g 0.00(0.01%)	8.38
(0.65829) LP (1) C128<br>s(2.76%)p35.25(97.20%)d 0.01(0.04%)	(0.14994) LV (1) U 1<br>s(0.25%)p 0.33(0.08%)d99.22(25.19%)f99.99(<br>74.47%)g 0.03(0.01%)	3.04
(0.65829) LP (1) C128<br>s(2.76%)p35.25(97.20%)d 0.01(0.04%)	(0.23573) LV (1) U132<br>s(0.07%)p 0.75(0.05%)d99.99(32.89%)f99.99(<br>66.99%)g 0.09(0.01%)	19.47
(0.65829) LP (1) C128<br>s(2.76%)p35.25(97.20%)d 0.01(0.04%)	(0.08336) LV (3) U132<br>s(35.55%)p 0.02(0.64%)d 1.24(44.13%)f 0.55(<br>19.67%)g 0.00(0.01%)	3.59
(0.61149) LP (1) C130<br>s(0.13%)p99.99(99.82%)d 0.42(0.05%)	(0.04438) LV (5) U 1<br>s(0.41%)p 2.71(1.12%)d80.54(33.31%)f99.99(<br>65.09%)g 0.14(0.06%)	4.97
(0.61149) LP (1) C130<br>s(0.13%)p99.99(99.82%)d 0.42(0.05%)	(0.23573) LV (1) U132<br>s(0.07%)p 0.75(0.05%)d99.99(32.89%)f99.99(<br>66.99%)g 0.09(0.01%)	4.04
(0.61149) LP (1) C130<br>s(0.13%)p99.99(99.82%)d 0.42(0.05%)	(0.04763) LV (5) U132<br>s(1.36%)p 0.92(1.25%)d25.45(34.66%)f46.01(<br>62.67%)g 0.05(0.06%)	4.77
0.95994) BD (1) C 13- C 36<br>(50.45%) 0.7103* C 13 s(33.45%)p 1.99(<br>66.49%)d 0.00(0.06%)<br>(49.55%) 0.7039* C 36 s(33.31%)p 2.00(<br>66.63%)d 0.00(0.06%)	(0.07547) LV (3) U 1<br>s(40.75%)p 0.02(0.72%)d 1.04(42.18%)f 0.40(<br>16.34%)g 0.00(0.01%)	9.98
0.95994) BD (1) C 13- C 36<br>(50.45%) 0.7103* C 13 s(33.45%)p 1.99(<br>66.49%)d 0.00(0.06%)<br>(49.55%) 0.7039* C 36 s(33.31%)p 2.00(<br>66.63%)d 0.00(0.06%)	(0.09685) LV (2) U132<br>s(1.38%)p 0.53(0.73%)d51.42(70.88%)f19.59(<br>27.00%)g 0.00(0.01%)	5.24
(0.96145) BD (1) C 13- C 43	(0.07547) LV (3) U 1	6.78

(49.68%) 0.7049* C 13 s(35.82%)p 1.79(64.11%)d 0.00(0.07%)<br>(50.32%) 0.7094* C 43 s(35.75%)p 1.80(64.18%)d 0.00(0.06%)	s(40.75%)p 0.02(0.72%)d 1.04(42.18%)f 0.40(16.34%)g 0.00(0.01%)	
(0.96145) BD (1) C 13- C 43<br>(49.68%) 0.7049* C 13 s(35.82%)p 1.79(64.11%)d 0.00(0.07%)<br>(50.32%) 0.7094* C 43 s(35.75%)p 1.80(64.18%)d 0.00(0.06%)	(0.04438) LV (5) U 1<br>s(0.41%)p 2.71(1.12%)d80.54(33.31%)f99.99(65.09%)g 0.14(0.06%)	3.63
(0.96145) BD (1) C 13- C 43<br>(49.68%) 0.7049* C 13 s(35.82%)p 1.79(64.11%)d 0.00(0.07%)<br>(50.32%) 0.7094* C 43 s(35.75%)p 1.80(64.18%)d 0.00(0.06%)	(0.08336) LV (3) U132<br>s(35.55%)p 0.02(0.64%)d 1.24(44.13%)f 0.55(19.67%)g 0.00(0.01%)	3.20
(0.96145) BD (1) C 13- C 43<br>(49.68%) 0.7049* C 13 s(35.82%)p 1.79(64.11%)d 0.00(0.07%)<br>(50.32%) 0.7094* C 43 s(35.75%)p 1.80(64.18%)d 0.00(0.06%)	(0.04763) LV (5) U132<br>s(1.36%)p 0.92(1.25%)d25.45(34.66%)f46.01(62.67%)g 0.05(0.06%)	4.90
(0.96093) BD (1) C 36- C130<br>(49.24%) 0.7017* C 36 s(33.71%)p 1.96(66.23%)d 0.00(0.06%)<br>(50.76%) 0.7125* C130 s(33.80%)p 1.96(66.14%)d 0.00(0.06%)	(0.07547) LV (3) U 1<br>s(40.75%)p 0.02(0.72%)d 1.04(42.18%)f 0.40(16.34%)g 0.00(0.01%)	7.89
(0.96093) BD (1) C 36- C130<br>(49.24%) 0.7017* C 36 s(33.71%)p 1.96(66.23%)d 0.00(0.06%)<br>(50.76%) 0.7125* C130 s(33.80%)p 1.96(66.14%)d 0.00(0.06%)	(0.04438) LV (5) U 1<br>s(0.41%)p 2.71(1.12%)d80.54(33.31%)f99.99(65.09%)g 0.14(0.06%)	3.65
(0.96093) BD (1) C 36- C130<br>(49.24%) 0.7017* C 36 s(33.71%)p 1.96(66.23%)d 0.00(0.06%)<br>(50.76%) 0.7125* C130 s(33.80%)p 1.96(66.14%)d 0.00(0.06%)	(0.08336) LV (3) U132<br>s(35.55%)p 0.02(0.64%)d 1.24(44.13%)f 0.55(19.67%)g 0.00(0.01%)	5.33
(0.96093) BD (1) C 36- C130<br>(49.24%) 0.7017* C 36 s(33.71%)p 1.96(66.23%)d 0.00(0.06%)<br>(50.76%) 0.7125* C130 s(33.80%)p 1.96(66.14%)d 0.00(0.06%)	(0.04763) LV (5) U132<br>s(1.36%)p 0.92(1.25%)d25.45(34.66%)f46.01(62.67%)g 0.05(0.06%)	4.12
(0.96234) BD (1) C 43- C128<br>(50.97%) 0.7139* C 43 s(33.71%)p 1.96(66.23%)d 0.00(0.06%)<br>(49.03%) 0.7002* C128 s(32.52%)p 2.07(67.41%)d 0.00(0.07%)	(0.07547) LV (3) U 1<br>s(40.75%)p 0.02(0.72%)d 1.04(42.18%)f 0.40(16.34%)g 0.00(0.01%)	3.38
(0.96234) BD (1) C 43- C128<br>(50.97%) 0.7139* C 43 s(33.71%)p 1.96(66.23%)d 0.00(0.06%)<br>(49.03%) 0.7002* C128 s(32.52%)p 2.07(67.41%)d 0.00(0.07%)	(0.04438) LV (5) U 1<br>s(0.41%)p 2.71(1.12%)d80.54(33.31%)f99.99(65.09%)g 0.14(0.06%)	3.35
(0.96234) BD (1) C 43- C128<br>(50.97%) 0.7139* C 43 s(33.71%)p 1.96(66.23%)d 0.00(0.06%)<br>(49.03%) 0.7002* C128 s(32.52%)p 2.07(67.41%)d 0.00(0.07%)	(0.08336) LV (3) U132<br>s(35.55%)p 0.02(0.64%)d 1.24(44.13%)f 0.55(19.67%)g 0.00(0.01%)	7.30
(0.96234) BD (1) C 43- C128<br>(50.97%) 0.7139* C 43 s(33.71%)p 1.96(66.23%)d 0.00(0.06%)<br>(49.03%) 0.7002* C128 s(32.52%)p 2.07(67.41%)d 0.00(0.07%)	(0.04763) LV (5) U132<br>s(1.36%)p 0.92(1.25%)d25.45(34.66%)f46.01(62.67%)g 0.05(0.06%)	5.05
(0.96087) BD (1) C126- C128	(0.09356) LV (2) U 1	4.83

(50.65%) 0.7117* C126 s(33.38%)p 1.99(66.56%)d 0.00(0.06%)<br>(49.35%) 0.7025* C128 s(32.30%)p 2.09(67.64%)d 0.00(0.06%)	s(3.51%)p 0.17(0.58%)d22.91(80.43%)f 4.41(15.47%)g 0.00(0.00%)	
(0.96087) BD (1) C126- C128<br>(50.65%) 0.7117* C126 s(33.38%)p 1.99(66.56%)d 0.00(0.06%)<br>(49.35%) 0.7025* C128 s(32.30%)p 2.09(67.64%)d 0.00(0.06%)	(0.09685) LV (2) U132<br>s(1.38%)p 0.53(0.73%)d51.42(70.88%)f19.59(27.00%)g 0.00(0.01%)	4.16
(0.96087) BD (1) C126- C128<br>(50.65%) 0.7117* C126 s(33.38%)p 1.99(66.56%)d 0.00(0.06%)<br>(49.35%) 0.7025* C128 s(32.30%)p 2.09(67.64%)d 0.00(0.06%)	(0.08336) LV (3) U132<br>s(35.55%)p 0.02(0.64%)d 1.24(44.13%)f 0.55(19.67%)g 0.00(0.01%)	9.90
(0.96028) BD (1) C126- C130<br>(49.76%) 0.7054* C126 s(35.66%)p 1.80(64.28%)d 0.00(0.07%)<br>(50.24%) 0.7088* C130 s(35.65%)p 1.80(64.29%)d 0.00(0.06%)	(0.09356) LV (2) U 1<br>s(3.51%)p 0.17(0.58%)d22.91(80.43%)f 4.41(15.47%)g 0.00(0.00%)	3.86
(0.96028) BD (1) C126- C130<br>(49.76%) 0.7054* C126 s(35.66%)p 1.80(64.28%)d 0.00(0.07%)<br>(50.24%) 0.7088* C130 s(35.65%)p 1.80(64.29%)d 0.00(0.06%)	(0.07547) LV (3) U 1<br>s(40.75%)p 0.02(0.72%)d 1.04(42.18%)f 0.40(16.34%)g 0.00(0.01%)	3.51
(0.96028) BD (1) C126- C130<br>(49.76%) 0.7054* C126 s(35.66%)p 1.80(64.28%)d 0.00(0.07%)<br>(50.24%) 0.7088* C130 s(35.65%)p 1.80(64.29%)d 0.00(0.06%)	(0.04438) LV (5) U 1<br>s(0.41%)p 2.71(1.12%)d80.54(33.31%)f99.99(65.09%)g 0.14(0.06%)	4.33
(0.96028) BD (1) C126- C130<br>(49.76%) 0.7054* C126 s(35.66%)p 1.80(64.28%)d 0.00(0.07%)<br>(50.24%) 0.7088* C130 s(35.65%)p 1.80(64.29%)d 0.00(0.06%)	(0.08336) LV (3) U132<br>s(35.55%)p 0.02(0.64%)d 1.24(44.13%)f 0.55(19.67%)g 0.00(0.01%)	8.25
(0.96028) BD (1) C126- C130<br>(49.76%) 0.7054* C126 s(35.66%)p 1.80(64.28%)d 0.00(0.07%)<br>(50.24%) 0.7088* C130 s(35.65%)p 1.80(64.29%)d 0.00(0.06%)	(0.04763) LV (5) U132<br>s(1.36%)p 0.92(1.25%)d25.45(34.66%)f46.01(62.67%)g 0.05(0.06%)	3.35

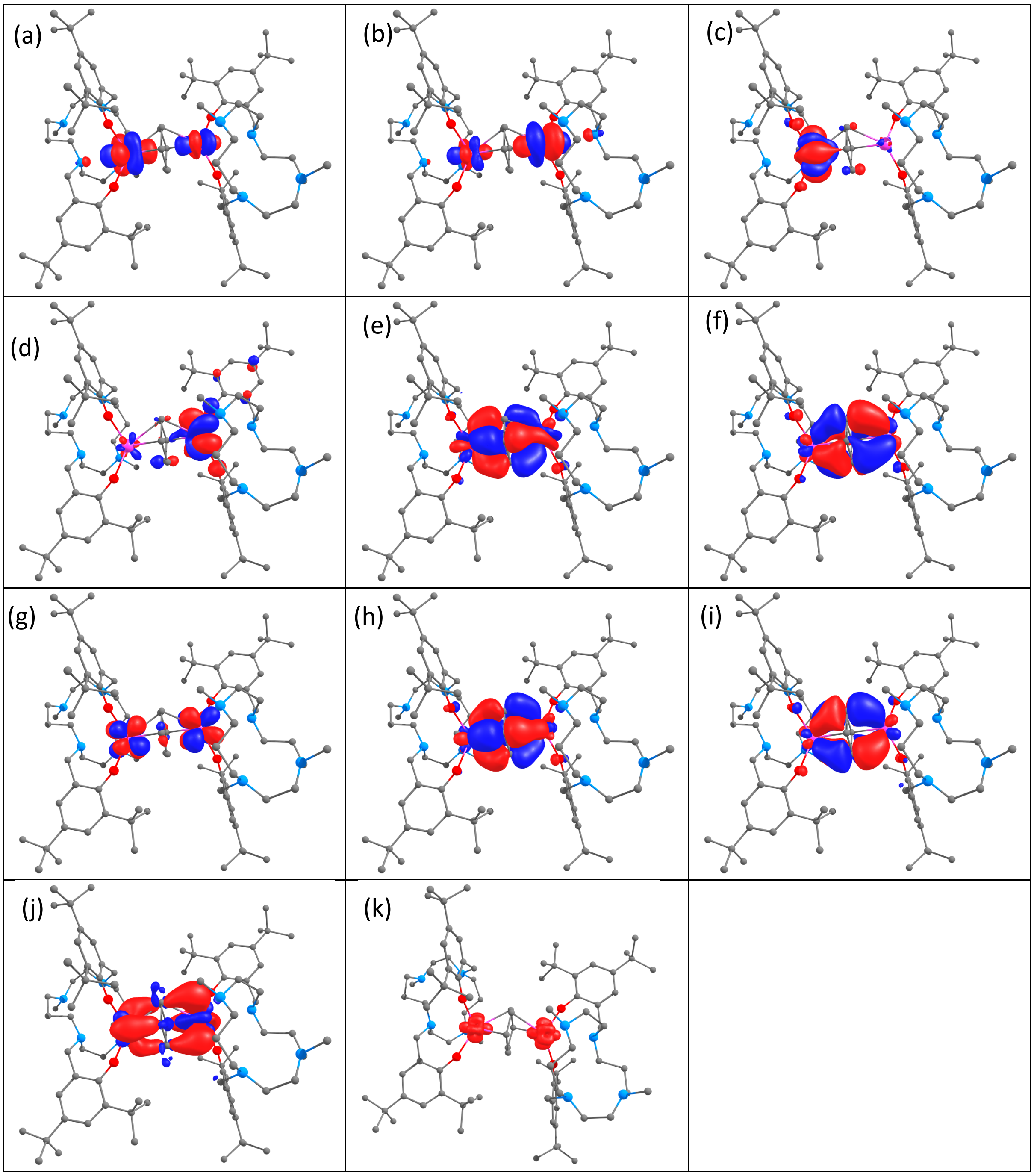

Figure S66. Computed MOs for LUC_6H_6UL complex **3** (s=2). (a)AMO-HOMO-5 (b)AMO-HOMO-4 (c)AMO-HOMO-3 (d)AMO-HOMO-2 (e)AMO-HOMO-1 (f)AMO-HOMO (g)AMO-LUMO (h)BMO-HOMO-1 (i)BMO-HOMO (j)BMO-LUMO (k) spin density plot

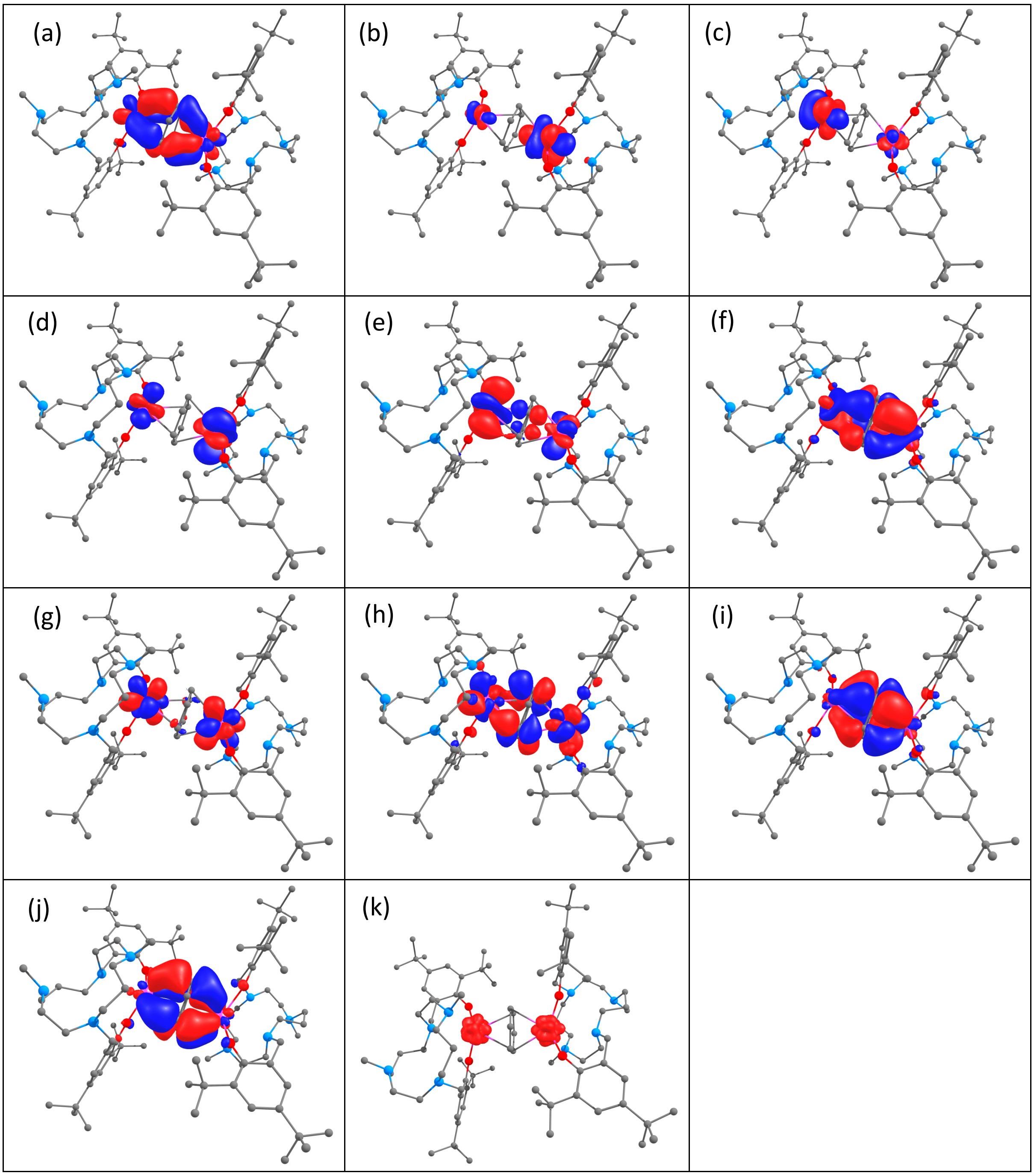

Figure S67. Computed MOs for LUC_6H_6UL complex **3** (s=3). a)AMO-HOMO-6 (b)AMO-HOMO-5 (c)AMO-HOMO-4 (d)AMO-HOMO-3 (e)AMO-HOMO-2 (f)AMO-HOMO-1 (g)AMO- HOMO (h) AMO- LUMO (i)BMO-HOMO (j)BMO-LUMO (k) spin density plot

Table S9. Energetics of two spin states computed for $^{Ad}LUC_6H_5CH_3UL^{Ad}$ complex

	ΔH (ΔG), kcal/mol
s=2	0.0
s=3	15.3(16.9)

Table S10. Comparison of selected bond parameters (Å) between DFT optimized and X-ray structures for ${}^{Ad}LUC_6H_5CH_3UL^{Ad}$ complex

Atom labels	DFT		X-ray
	s=2	s=3	
U1-N3	2.33	2.35	2.33
U1-N4	2.33	2.35	2.34
U1-C7	2.58	2.79	2.57
U1-C8	2.51	2.74	2.54
U1-C9	2.66	2.78	2.66
U1-C10	2.49	2.72	2.54
U1-C11	2.61	2.82	2.59
U1-C12	2.66	2.95	2.66
U2-N5	2.32	2.33	2.34
U2-N6	2.32	2.33	2.33
U2-C7	2.61	2.69	2.60
U2-C8	2.63	2.65	2.62
U2-C9	2.46	2.56	2.50
U2-C10	2.66	2.65	2.64
U2-C11	2.59	2.65	2.61
U2-C12	2.60	2.61	2.59

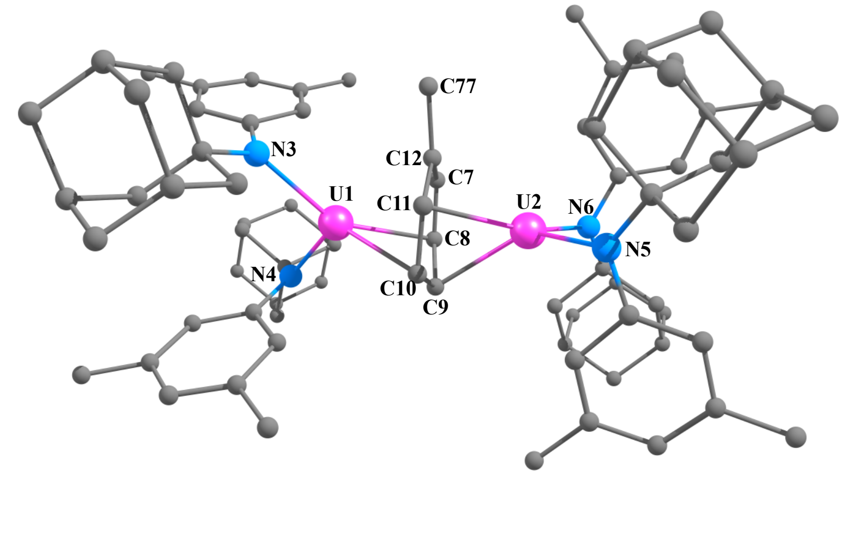

Table S11. Computed natural charges for selected atoms in ${}^{Ad}LUC_6H_5CH_3UL^{Ad}$ complex (s=2)

Atom labels	Natural charges
U1	1.24560
U2	1.25222
N3	-0.72521
N4	-0.71393
N5	-0.72082
N6	-0.72498
C7	-0.45072
C8	-0.43667
C9	-0.48909
C10	-0.47808
C11	-0.41635
C12	-0.22285
C77	-0.69044

Table S12. Computed Wiberg bond index between selected atoms in ${}^{Ad}LUC_6H_5CH_3UL^{Ad}$ complex (s=2)

Atom labels	Wiberg bond index	Atom labels	Wiberg bond index	Atom labels	Wiberg bond index
U1	0.0000	U1	0.0000	U1	0.0000
N3	0.6414	N4	0.6476	C7	0.3455
Atom labels	Wiberg bond index	Atom labels	Wiberg bond index	Atom labels	Wiberg bond index
U1	0.0000	U1	0.0000	U1	0.0000
C8	0.4194	C9	0.2542	C10	0.4521
Atom labels	Wiberg bond index	Atom labels	Wiberg bond index	Atom labels	Wiberg bond index
U1	0.0000	U1	0.0000	U1	0.0000
C11	0.3253	C12	0.2689	C77	0.0122
Atom labels	Wiberg bond index	Atom labels	Wiberg bond index	Atom labels	Wiberg bond index
U2	0.0000	U2	0.0000	U2	0.0000
N5	0.6510	N6	0.6590	C7	0.3111
Atom labels	Wiberg bond index	Atom labels	Wiberg bond index	Atom labels	Wiberg bond index
U2	0.0000	U2	0.0000	U2	0.0000

C8	0.2991	C9	0.4941	C10	0.2718
Atom labels	Wiberg bond index	Atom labels	Wiberg bond index	Atom labels	Wiberg bond index
U2	0.0000	U2	0.0000	U2	0.0000
C11	0.3411	C12	0.3375	C77	0.0110

Bonding orbitals (Alpha Molecular orbitals, AMO) from NBO analysis for ground state spin of $^{Ad}LUC_6H_5CH_3UL^{Ad}$ complex
(0.88011) BD (1) U 1- N 3
(11.98%) 0.3461* U 1 s(0.67%)p 0.51(0.34%)d72.56(48.25%) f76.28(50.73%)g 0.02(0.01%)
(88.02%) 0.9382* N 3 s(2.76%)p35.23(97.22%)d 0.01(0.02%)
(0.87678) BD (1) U 1- N 4
(11.95%) 0.3456* U 1 s(0.81%)p 0.44(0.35%)d61.23(49.37%) f61.35(49.46%)g 0.02(0.01%)
(88.05%) 0.9384* N 4 s(3.29%)p29.36(96.68%)d 0.01(0.02%)
(0.87836) BD (1) U 2- N 5
(11.55%) 0.3399* U 2 s(0.20%)p 1.87(0.37%)d99.99(52.03%) f99.99(47.38%)g 0.08(0.02%)
(88.45%) 0.9405* N 5 s(2.89%)p33.57(97.09%)d 0.01(0.02%)
(0.88262) BD (1) U 2- N 6
(11.82%) 0.3438* U 2 s(0.33%)p 1.19(0.39%)d99.99(54.59%) f99.99(44.68%)g 0.04(0.01%)
(88.18%) 0.9390* N 6 s(2.93%)p33.07(97.05%)d 0.01(0.02%)
(0.82005) BD (1) U 2- C 9
(25.80%) 0.5079* U 2 s(5.03%)p 0.09(0.43%)d 8.25(41.50%) f10.55(53.03%)g 0.00(0.01%)
(74.20%) 0.8614* C 9 s(6.33%)p14.78(93.61%)d 0.01(0.05%)
(0.93300) BD (1) C 7- C 8
(50.65%) 0.7117* C 7 s(34.09%)p 1.93(65.84%)d 0.00(0.06%)
(49.35%) 0.7025* C 8 s(33.26%)p 2.00(66.67%)d 0.00(0.07%)
(0.84869) BD (2) C 7- C 8
(50.62%) 0.7115* C 7 s(0.01%)p 1.00(99.89%)d 0.00(0.10%)
(49.38%) 0.7027* C 8 s(0.49%)p99.99(99.40%)d 0.22(0.11%)
(0.93521) BD (1) C 7- C 12
(49.42%) 0.7030* C 7 s(34.13%)p 1.93(65.80%)d 0.00(0.07%)
(50.58%) 0.7112* C 12 s(33.16%)p 2.01(66.79%)d 0.00(0.05%)
(0.93010) BD (1) C 8- C 9
(49.94%) 0.7067* C 8 s(33.44%)p 1.99(66.49%)d 0.00(0.07%)
(50.06%) 0.7075* C 9 s(31.26%)p 2.20(68.67%)d 0.00(0.07%)
(0.92842) BD (1) C 9- C 10
(49.86%) 0.7061* C 9 s(30.78%)p 2.25(69.15%)d 0.00(0.07%)
(50.14%) 0.7081* C 10 s(32.85%)p 2.04(67.08%)d 0.00(0.06%)
(0.93316) BD (1) C 10- C 11
(49.92%) 0.7065* C 10 s(33.15%)p 2.01(66.78%)d 0.00(0.07%)
(50.08%) 0.7077* C 11 s(33.92%)p 1.95(66.02%)d 0.00(0.06%)
(0.93584) BD (1) C 11- C 12
(49.14%) 0.7010* C 11 s(34.30%)p 1.91(65.63%)d 0.00(0.07%)
(50.86%) 0.7132* C 12 s(33.14%)p 2.02(66.81%)d 0.00(0.05%)
(0.84424) BD (2) C 11- C 12
(49.52%) 0.7037* C 11 s(0.02%)p99.99(99.88%)d 5.58(0.10%)
(50.48%) 0.7105* C 12 s(0.15%)p99.99(99.76%)d 0.64(0.09%)
(0.98670) BD (1) C 12- C 77
(52.61%) 0.7253* C 12 s(33.41%)p 1.99(66.55%)d 0.00(0.04%)
(47.39%) 0.6884* C 77 s(29.06%)p 2.44(70.88%)d 0.00(0.06%)

Table S13. NBO Second order perturbation analysis (AMO) for $^{Ad}LUC_6H_5CH_3UL^{Ad}$ complex (s=2)

Donor NBO	Acceptor NBO	E(2) kcal/mol
(0.87145) LP (1) N 3<br>s(23.38%)p 3.28(76.60%)d 0.00(0.02%)	(0.15873) LV (3) U 1<br>s(3.56%)p 0.07(0.24%)d23.57(83.86%)f 3.47(12.33%)g 0.00(0.01%)	34.32
(0.87145) LP (1) N 3<br>s(23.38%)p 3.28(76.60%)d 0.00(0.02%)	(0.11744) LV (5) U 1<br>s(0.16%)p 0.57(0.09%)d99.99(79.64%)f99.99(20.10%)g 0.04(0.01%)	12.36
(0.87145) LP (1) N 3	(0.06751) LV (6) U 1	9.46

s(23.38%)p 3.28(76.60%)d 0.00(0.02%)	s(66.20%)p 0.00(0.32%)d 0.36(23.59%)f 0.15(9.88%)g 0.00(0.01%)	
(0.86925) LP (1) N 4<br>s(23.40%)p 3.27(76.58%)d 0.00(0.02%)	(0.11744) LV (5) U 1<br>s(0.16%)p 0.57(0.09%)d99.99(79.64%)f99.99(20.10%)g 0.04(0.01%)	11.26
(0.86925) LP (1) N 4<br>s(23.40%)p 3.27(76.58%)d 0.00(0.02%)	(0.06751) LV (6) U 1<br>s(66.20%)p 0.00(0.32%)d 0.36(23.59%)f 0.15(9.88%)g 0.00(0.01%)	6.73
(0.63550) LP (1) C 10<br>s(1.84%)p53.39(98.10%)d 0.03(0.06%)	(0.32605) LV (1) U 1<br>s(0.48%)p 0.12(0.06%)d73.01(35.09%)f99.99(64.36%)g 0.02(0.01%)	19.00
(0.63550) LP (1) C 10<br>s(1.84%)p53.39(98.10%)d 0.03(0.06%)	(0.19233) LV (2) U 1<br>s(7.54%)p 0.00(0.03%)d 5.03(37.91%)f 7.23(54.52%)g 0.00(0.01%)	10.21
(0.63550) LP (1) C 10<br>s(1.84%)p53.39(98.10%)d 0.03(0.06%)	(0.13594) LV (4) U 1<br>s(0.01%)p 1.00(1.02%)d65.42(66.66%)f31.71(32.31%)g 0.00(0.00%)	12.11
(0.93300) BD (1) C 7- C 8<br>(50.65%) 0.7117* C 7 s(34.09%)p 1.93(65.84%)d 0.00(0.06%)<br>(49.35%) 0.7025* C 8 s(33.26%)p 2.00(66.67%)d 0.00(0.07%)	(0.13594) LV (4) U 1<br>s(0.01%)p 1.00(1.02%)d65.42(66.66%)f31.71(32.31%)g 0.00(0.00%)	19.10
(0.84869) BD (2) C 7- C 8<br>(50.62%) 0.7115* C 7 s(0.01%)p 1.00(99.89%)d 0.00(0.10%)<br>(49.38%) 0.7027* C 8 s(0.49%)p99.99(99.40%)d 0.22(0.11%)	(0.13594) LV (4) U 1<br>s(0.01%)p 1.00(1.02%)d65.42(66.66%)f31.71(32.31%)g 0.00(0.00%)	11.26
(0.84869) BD (2) C 7- C 8<br>(50.62%) 0.7115* C 7 s(0.01%)p 1.00(99.89%)d 0.00(0.10%)<br>(49.38%) 0.7027* C 8 s(0.49%)p99.99(99.40%)d 0.22(0.11%)	(0.19233) LV (2) U 1<br>s(7.54%)p 0.00(0.03%)d 5.03(37.91%)f 7.23(54.52%)g 0.00(0.01%)	7.59
(0.93521) BD (1) C 7- C 12<br>(49.42%) 0.7030* C 7 s(34.13%)p 1.93(65.80%)d 0.00(0.07%)<br>(50.58%) 0.7112* C 12 s(33.16%)p 2.01(66.79%)d 0.00(0.05%)	(0.13594) LV (4) U 1<br>s(0.01%)p 1.00(1.02%)d65.42(66.66%)f31.71(32.31%)g 0.00(0.00%)	6.33
(0.93521) BD (1) C 7- C 12<br>(49.42%) 0.7030* C 7 s(34.13%)p 1.93(65.80%)d 0.00(0.07%)<br>(50.58%) 0.7112* C 12 s(33.16%)p 2.01(66.79%)d 0.00(0.05%)	(0.11744) LV (5) U 1<br>s(0.16%)p 0.57(0.09%)d99.99(79.64%)f99.99(20.10%)g 0.04(0.01%)	9.36
(0.93010) BD (1) C 8- C 9<br>(49.94%) 0.7067* C 8 s(33.44%)p 1.99(66.49%)d 0.00(0.07%)<br>(50.06%) 0.7075* C 9 s(31.26%)p 2.20(68.67%)d 0.00(0.07%)	(0.11744) LV (5) U 1<br>s(0.16%)p 0.57(0.09%)d99.99(79.64%)f99.99(20.10%)g 0.04(0.01%)	13.56
(0.92842) BD (1) C 9- C 10<br>(49.86%) 0.7061* C 9 s(30.78%)p 2.25(69.15%)d 0.00(0.07%)<br>(50.14%) 0.7081* C 10 s(32.85%)p 2.04(67.08%)d 0.00(0.06%)	(0.13594) LV (4) U 1<br>s(0.01%)p 1.00(1.02%)d65.42(66.66%)f31.71(32.31%)g 0.00(0.00%)	6.84
(0.92842) BD (1) C 9- C 10<br>(49.86%) 0.7061* C 9 s(30.78%)p 2.25(69.15%)d 0.00(0.07%)<br>(50.14%) 0.7081* C 10 s(32.85%)p 2.04(67.08%)d 0.00(0.06%)	(0.11744) LV (5) U 1<br>s(0.16%)p 0.57(0.09%)d99.99(79.64%)f99.99(20.10%)g 0.04(0.01%)	12.08
(0.93316) BD (1) C 10- C 11<br>(49.92%) 0.7065* C 10 s(33.15%)p 2.01(66.78%)d 0.00(0.07%)	(0.13594) LV (4) U 1<br>s(0.01%)p 1.00(1.02%)d65.42(66.66%)f31.71(32.31%)g 0.00(0.00%)	20.27

(50.08%) 0.7077* C 11 s(33.92%)p 1.95(66.02%)d 0.00(0.06%)		
(0.93584) BD (1) C 11- C 12<br>(49.14%) 0.7010* C 11 s(34.30%)p 1.91(65.63%)d 0.00(0.07%)<br>(50.86%) 0.7132* C 12 s(33.14%)p 2.02(66.81%)d 0.00(0.05%)	(0.11744) LV (5) U 1<br>s(0.16%)p 0.57(0.09%)d99.99(79.64%)f99.99(20.10%)g 0.04(0.01%)	10.77
(0.84424) BD (2) C 11- C 12<br>(49.52%) 0.7037* C 11 s(0.02%)p99.99(99.88%)d 5.58(0.10%)<br>(50.48%) 0.7105* C 12 s(0.15%)p99.99(99.76%)d 0.64(0.09%)	(0.11744) LV (5) U 1<br>s(0.16%)p 0.57(0.09%)d99.99(79.64%)f99.99(20.10%)g 0.04(0.01%)	5.81
(0.87007) LP (1) N 5<br>s(23.13%)p 3.32(76.85%)d 0.00(0.02%)	(0.20993) LV (1) U 2<br>s(0.25%)p 0.76(0.19%)d99.99(33.90%)f99.99(65.66%)g 0.01(0.00%)	15.02
(0.87007) LP (1) N 5<br>s(23.13%)p 3.32(76.85%)d 0.00(0.02%)	(0.14943) LV (3) U 2<br>s(2.00%)p 0.26(0.51%)d42.16(84.47%)f 6.49(13.00%)g 0.01(0.01%)	19.38
(0.87007) LP (1) N 5<br>s(23.13%)p 3.32(76.85%)d 0.00(0.02%)	(0.11688) LV (4) U 2<br>s(0.04%)p 1.41(0.06%)d99.99(79.43%)f99.99(20.46%)g 0.16(0.01%)	14.76
(0.87007) LP (1) N 5<br>s(23.13%)p 3.32(76.85%)d 0.00(0.02%)	(0.06796) LV (5) U 2<br>s(74.83%)p 0.01(0.38%)d 0.21(15.88%)f 0.12(8.89%)g 0.00(0.01%)	8.01
(0.86957) LP (1) N 6<br>s(23.00%)p 3.35(76.99%)d 0.00(0.01%)	(0.20993) LV (1) U 2<br>s(0.25%)p 0.76(0.19%)d99.99(33.90%)f99.99(65.66%)g 0.01(0.00%)	8.96
(0.86957) LP (1) N 6<br>s(23.00%)p 3.35(76.99%)d 0.00(0.01%)	(0.16594) LV (2) U 2<br>s(2.20%)p 0.24(0.52%)d24.94(54.98%)f19.18(42.29%)g 0.00(0.01%)	21.63
(0.86957) LP (1) N 6<br>s(23.00%)p 3.35(76.99%)d 0.00(0.01%)	(0.14943) LV (3) U 2<br>s(2.00%)p 0.26(0.51%)d42.16(84.47%)f 6.49(13.00%)g 0.01(0.01%)	15.77
(0.86957) LP (1) N 6<br>s(23.00%)p 3.35(76.99%)d 0.00(0.01%)	(0.11688) LV (4) U 2<br>s(0.04%)p 1.41(0.06%)d99.99(79.43%)f99.99(20.46%)g 0.16(0.01%)	10.97
(0.86957) LP (1) N 6<br>s(23.00%)p 3.35(76.99%)d 0.00(0.01%)	(0.06796) LV (5) U 2<br>s(74.83%)p 0.01(0.38%)d 0.21(15.88%)f 0.12(8.89%)g 0.00(0.01%)	9.58
(0.63550) LP (1) C 10<br>s(1.84%)p53.39(98.10%)d 0.03(0.06%)	(0.13259) BD*(1) U 2- C 9<br>(74.20%) 0.8614* U 2 s(5.03%)p 0.09(0.43%)d 8.25(41.50%)f10.55(53.03%)g 0.00(0.01%)<br>(25.80%) -0.5079* C 9 s(6.33%)p14.78(93.61%)d 0.01(0.05%)	13.96
(0.93300) BD (1) C 7- C 8<br>(50.65%) 0.7117* C 7 s(34.09%)p 1.93(65.84%)d 0.00(0.06%)<br>(49.35%) 0.7025* C 8 s(33.26%)p 2.00(66.67%)d 0.00(0.07%)	(0.11688) LV (4) U 2<br>s(0.04%)p 1.41(0.06%)d99.99(79.43%)f99.99(20.46%)g 0.16(0.01%)	14.20
(0.84869) BD (2) C 7- C 8<br>(50.62%) 0.7115* C 7 s(0.01%)p 1.00(99.89%)d 0.00(0.10%)<br>(49.38%) 0.7027* C 8 s(0.49%)p99.99(99.40%)d 0.22(0.11%)	(0.13259) BD*(1) U 2- C 9<br>(74.20%) 0.8614* U 2 s(5.03%)p 0.09(0.43%)d 8.25(41.50%)f10.55(53.03%)g 0.00(0.01%)<br>(25.80%) -0.5079* C 9 s(6.33%)p14.78(93.61%)d 0.01(0.05%)	10.02
(0.93521) BD (1) C 7- C 12<br>(49.42%) 0.7030* C 7 s(34.13%)p 1.93(65.80%)d 0.00(0.07%)<br>(50.58%) 0.7112* C 12 s(33.16%)p 2.01(66.79%)d 0.00(0.05%)	(0.06796) LV (5) U 2<br>s(74.83%)p 0.01(0.38%)d 0.21(15.88%)f 0.12(8.89%)g 0.00(0.01%)	5.38

(0.93010) BD (1) C 8- C 9<br>(49.94%) 0.7067* C 8 s(33.44%)p 1.99(66.49%)d 0.00(0.07%)<br>(50.06%) 0.7075* C 9 s(31.26%)p 2.20(68.67%)d 0.00(0.07%)	(0.14943) LV (3) U 2<br>s(2.00%)p 0.26(0.51%)d42.16(84.47%)f 6.49(13.00%)g 0.01(0.01%)	8.23
(0.92842) BD (1) C 9- C 10<br>(49.86%) 0.7061* C 9 s(30.78%)p 2.25(69.15%)d 0.00(0.07%)<br>(50.14%) 0.7081* C 10 s(32.85%)p 2.04(67.08%)d 0.00(0.06%)	(0.14943) LV (3) U 2<br>s(2.00%)p 0.26(0.51%)d42.16(84.47%)f 6.49(13.00%)g 0.01(0.01%)	7.14
(0.93316) BD (1) C 10- C 11<br>(49.92%) 0.7065* C 10 s(33.15%)p 2.01(66.78%)d 0.00(0.07%)<br>(50.08%) 0.7077* C 11 s(33.92%)p 1.95(66.02%)d 0.00(0.06%)	(0.11688) LV (4) U 2<br>s(0.04%)p 1.41(0.06%)d99.99(79.43%)f99.99(20.46%)g 0.16(0.01%)	13.95
(0.93584) BD (1) C 11- C 12<br>(49.14%) 0.7010* C 11 s(34.30%)p 1.91(65.63%)d 0.00(0.07%)<br>(50.86%) 0.7132* C 12 s(33.14%)p 2.02(66.81%)d 0.00(0.05%)	(0.16594) LV (2) U 2<br>s(2.20%)p 0.24(0.52%)d24.94(54.98%)f19.18(42.29%)g 0.00(0.01%)	13.63
(0.84424) BD (2) C 11- C 12<br>(49.52%) 0.7037* C 11 s(0.02%)p99.99(99.88%)d 5.58(0.10%)<br>(50.48%) 0.7105* C 12 s(0.15%)p99.99(99.76%)d 0.64(0.09%)	(0.16594) LV (2) U 2<br>s(2.20%)p 0.24(0.52%)d24.94(54.98%)f19.18(42.29%)g 0.00(0.01%)	13.35

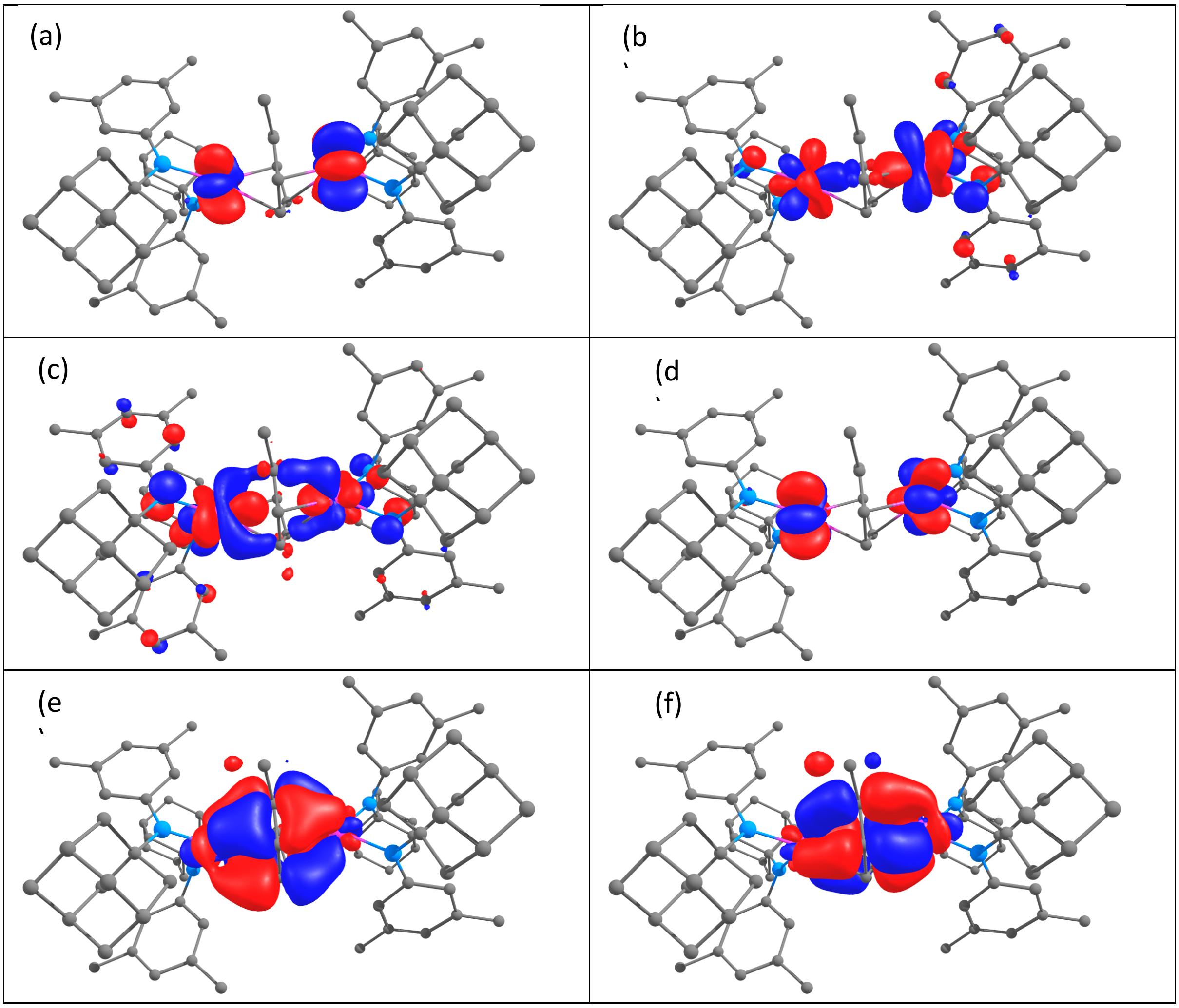

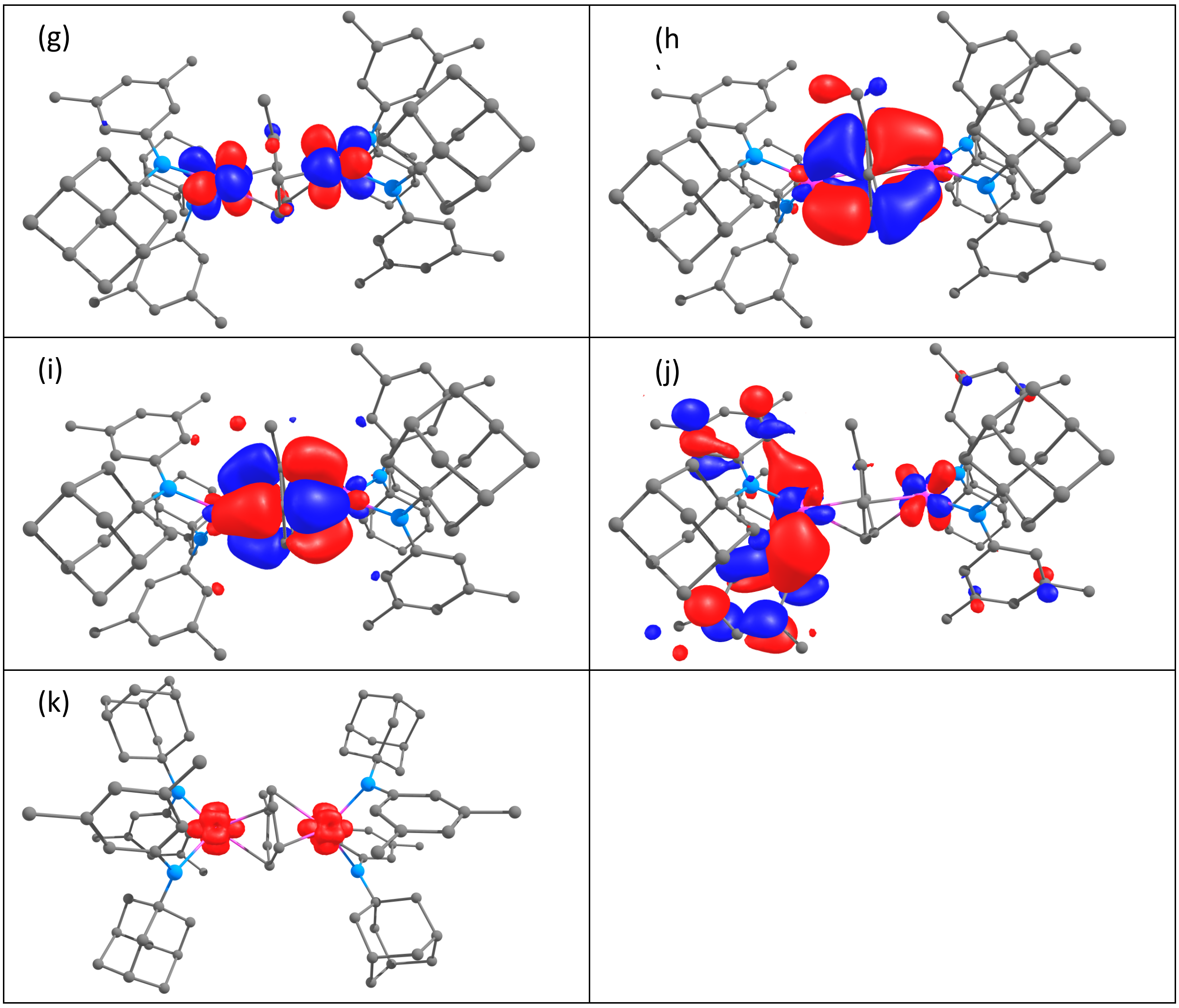

Figure S68. Computed MOs for $^{Ad}LUC_6H_5CH_3UL^{Ad}$ complex (s=2). (a)AMO-HOMO-5 (b)AMO-HOMO-4 (c)AMO-HOMO-3 (d)AMO-HOMO-2 (e)AMO-HOMO-1 (f)AMO-HOMO (g)AMO-LUMO (h)BMO-HOMO-1 (i)BMO-HOMO (j)BMO-LUMO (k)spin density plot

Optimized coordinates

A

U	-0.023122000	-0.746079000	0.106174000
I	0.509148000	-0.164877000	3.161881000
O	1.633109000	0.690753000	-0.265595000
O	-1.771759000	0.626849000	0.109847000
N	2.249981000	-2.227842000	-0.542058000
N	-0.106177000	-3.562867000	1.106542000
N	-2.345984000	-2.071912000	-1.045483000
N	-0.176337000	-0.701013000	-2.738668000
C	2.100309000	-3.630446000	-0.112797000
H	3.100306000	-4.085378000	0.006426000
H	1.617287000	-4.183866000	-0.923464000
C	1.341740000	-3.835784000	1.191794000
H	1.752999000	-3.191334000	1.974566000
H	1.511007000	-4.877177000	1.522274000
C	-0.740151000	-4.633606000	0.315598000
H	-0.634265000	-5.583509000	0.871246000
H	-0.185737000	-4.762055000	-0.618934000
C	-2.205830000	-4.430492000	-0.044287000
H	-2.597401000	-5.417538000	-0.320385000

H	-2.796896000	-4.121820000	0.822537000
C	-2.461834000	-3.528624000	-1.245293000
H	-1.767282000	-3.826556000	-2.038508000
H	-3.476943000	-3.744323000	-1.626694000
C	-2.540968000	-1.431686000	-2.357106000
H	-2.816516000	-0.389402000	-2.191459000
H	-3.386705000	-1.889400000	-2.899736000
C	-1.299423000	-1.517034000	-3.232661000
H	-0.961425000	-2.553954000	-3.315696000
H	-1.567099000	-1.200807000	-4.256752000
C	1.061064000	-1.071245000	-3.459139000
H	1.770385000	-0.249299000	-3.322763000
H	0.836802000	-1.114236000	-4.539595000
C	1.742101000	-2.375211000	-3.026744000
H	2.265819000	-2.770864000	-3.905370000
H	0.999637000	-3.140470000	-2.765685000
C	2.786197000	-2.218958000	-1.917861000
H	3.547127000	-3.012265000	-2.014776000
H	3.314325000	-1.272277000	-2.059181000
C	-0.657059000	-3.616386000	2.472788000
H	-1.722289000	-3.377852000	2.467153000
H	-0.161734000	-2.873617000	3.099517000
H	-0.526968000	-4.621162000	2.909997000
C	-0.453702000	0.714288000	-3.038036000
H	0.377319000	1.323983000	-2.687040000
H	-1.354828000	1.049426000	-2.524293000
H	-0.579209000	0.873182000	-4.122214000
C	3.198909000	-1.554600000	0.401805000
H	2.664503000	-1.396245000	1.349390000
H	4.027461000	-2.249304000	0.616764000
C	3.790872000	-0.242633000	-0.028401000
C	5.178420000	-0.133410000	-0.090992000
H	5.771337000	-1.032050000	0.071019000
C	5.810654000	1.086611000	-0.328735000
C	4.976214000	2.199591000	-0.456272000
H	5.440333000	3.166203000	-0.599233000
C	3.576370000	2.156084000	-0.398474000
C	2.958313000	0.884222000	-0.238994000
C	7.340853000	1.165458000	-0.402760000
C	7.849195000	0.247471000	-1.531933000
H	7.446638000	0.561287000	-2.501014000
H	7.554893000	-0.794442000	-1.370256000
H	8.943638000	0.278350000	-1.591722000
C	7.946695000	0.704514000	0.937652000
H	9.041859000	0.747709000	0.901639000
H	7.662311000	-0.324582000	1.178916000
H	7.606334000	1.343576000	1.758825000
C	7.839752000	2.588990000	-0.686622000
H	8.933596000	2.592796000	-0.744869000
H	7.550461000	3.287415000	0.105705000
H	7.456775000	2.973413000	-1.638165000
C	2.771341000	3.465141000	-0.446520000
C	3.667484000	4.710402000	-0.544648000
H	4.259158000	4.729067000	-1.467021000
H	4.350641000	4.795717000	0.307066000
H	3.033544000	5.603534000	-0.545305000
C	1.941932000	3.596628000	0.845813000
H	1.343813000	4.515252000	0.819618000
H	2.598328000	3.645562000	1.720840000
H	1.267665000	2.749745000	0.979204000
C	1.841015000	3.475453000	-1.669485000
H	2.411180000	3.353018000	-2.597895000

H	1.293104000	4.422797000	-1.729893000
H	1.114143000	2.669185000	-1.589912000
C	-3.382776000	-1.659801000	-0.044612000
H	-2.935761000	-1.805489000	0.949403000
H	-4.233456000	-2.355786000	-0.110909000
C	-3.938283000	-0.265689000	-0.129780000
C	-5.313633000	-0.098134000	-0.278179000
H	-5.928323000	-0.986004000	-0.417043000
C	-5.908350000	1.163279000	-0.233902000
C	-5.054206000	2.247723000	-0.009952000
H	-5.491664000	3.235144000	0.054690000
C	-3.665899000	2.144089000	0.144162000
C	-3.086576000	0.850045000	0.047115000
C	-7.426290000	1.309595000	-0.405033000
H	-7.650176000	3.226289000	0.633860000
C	-2.818193000	3.390220000	0.440679000
C	-3.665228000	4.668433000	0.544044000
H	-4.406687000	4.609530000	1.348294000
H	-4.187115000	4.900830000	-0.391384000
H	-3.006426000	5.513833000	0.769179000
C	-2.091203000	3.207487000	1.787906000
H	-2.814635000	3.093260000	2.602466000
H	-1.474095000	4.086901000	2.005987000
H	-1.444093000	2.329226000	1.784718000
C	-1.793084000	3.607405000	-0.685754000
H	-1.190251000	4.499705000	-0.483136000
H	-2.296860000	3.752454000	-1.648905000
H	-1.119692000	2.754260000	-0.764678000
C	-8.148396000	0.528279000	0.710564000
H	-7.870753000	0.912405000	1.697560000
H	-7.898329000	-0.537129000	0.686312000
H	-9.235999000	0.617660000	0.602792000
C	-7.847343000	0.742790000	-1.775329000
H	-8.931501000	0.832812000	-1.913580000
H	-7.587022000	-0.315939000	-1.873442000
H	-7.354471000	1.284461000	-2.589691000
C	-7.884179000	2.772717000	-0.334943000
H	-7.422059000	3.382276000	-1.118829000
H	-8.969779000	2.827200000	-0.470689000

B

U	-0.019861000	-0.761476000	-0.510710000
O	-1.682431000	0.716492000	-0.303122000
O	1.741660000	0.619019000	-0.617032000
N	-2.326405000	-2.240590000	0.073623000
N	0.037145000	-3.500375000	-1.597981000
N	2.240610000	-2.101275000	0.644290000
N	0.040894000	-0.824497000	2.430974000
C	-2.163698000	-3.629786000	-0.389717000
H	-3.158887000	-4.095073000	-0.514680000
H	-1.664401000	-4.196601000	0.402606000
C	-1.405462000	-3.787025000	-1.700464000
H	-1.818993000	-3.114554000	-2.458071000
H	-1.562857000	-4.816454000	-2.072143000
C	0.696698000	-4.634087000	-0.928775000
H	0.661926000	-5.511918000	-1.599677000
H	0.111583000	-4.904844000	-0.043323000
C	2.134949000	-4.409641000	-0.481673000
H	2.538941000	-5.401669000	-0.243205000
H	2.755840000	-4.039130000	-1.302360000
C	2.315326000	-3.568685000	0.779999000
H	1.564451000	-3.892742000	1.509172000

H	3.299011000	-3.820723000	1.217798000
C	2.416197000	-1.511090000	1.982160000
H	2.675602000	-0.458835000	1.852315000
H	3.271106000	-1.974014000	2.506965000
C	1.182006000	-1.644317000	2.867206000
H	0.856349000	-2.687975000	2.910587000
H	1.467684000	-1.372986000	3.900345000
C	-1.187642000	-1.261577000	3.121676000
H	-1.901450000	-0.433259000	3.069773000
H	-0.964314000	-1.411446000	4.193405000
C	-1.869033000	-2.516809000	2.562996000
H	-2.419774000	-2.976665000	3.392578000
H	-1.124555000	-3.265496000	2.263139000
C	-2.883011000	-2.264719000	1.439556000
H	-3.679524000	-3.027719000	1.490721000
H	-3.373457000	-1.302885000	1.611307000
C	0.572766000	-3.367402000	-2.963968000
H	1.649515000	-3.187079000	-2.937219000
H	0.111437000	-2.496834000	-3.443314000
H	0.382978000	-4.282598000	-3.553987000
C	0.289358000	0.582098000	2.775408000
H	-0.554432000	1.190159000	2.448535000
H	1.180828000	0.955014000	2.267864000
H	0.422377000	0.711213000	3.863645000
C	-3.251836000	-1.539864000	-0.872186000
H	-2.675819000	-1.355547000	-1.797745000
H	-4.076224000	-2.225570000	-1.131279000
C	-3.842235000	-0.237514000	-0.421235000
C	-5.226337000	-0.131512000	-0.292256000
H	-5.823129000	-1.032471000	-0.425586000
C	-5.849949000	1.085530000	-0.017317000
C	-5.014142000	2.202465000	0.076578000
H	-5.472561000	3.167136000	0.250577000
C	-3.619987000	2.161220000	-0.051059000
C	-3.005443000	0.894054000	-0.253668000
C	-7.374781000	1.158714000	0.136605000
C	-7.823592000	0.222569000	1.276079000
H	-7.369031000	0.520259000	2.227059000
H	-7.539996000	-0.816739000	1.082080000
H	-8.913257000	0.253090000	1.395303000
C	-8.048319000	0.717112000	-1.177691000
H	-9.140557000	0.756185000	-1.085788000
H	-7.773557000	-0.307005000	-1.449225000
H	-7.751801000	1.370580000	-2.004633000
C	-7.861622000	2.576030000	0.468489000
H	-8.950710000	2.574599000	0.586151000
H	-7.617486000	3.287729000	-0.327193000
H	-7.427812000	2.946723000	1.403452000
C	-2.801712000	3.460312000	-0.010068000
C	-3.680360000	4.713152000	0.131085000
H	-4.241611000	4.725526000	1.072359000
H	-4.391097000	4.814674000	-0.696215000
H	-3.038620000	5.600816000	0.122416000
C	-2.006302000	3.600219000	-1.323307000
H	-1.380498000	4.500090000	-1.294619000
H	-2.689031000	3.691211000	-2.175262000
H	-1.363280000	2.735082000	-1.493914000
C	-1.842494000	3.435441000	1.191717000
H	-2.399758000	3.324688000	2.129545000
H	-1.267836000	4.367051000	1.247656000
H	-1.141185000	2.607156000	1.096265000
C	3.316271000	-1.675635000	-0.309927000

H	2.898790000	-1.793058000	-1.321570000
H	4.158650000	-2.379994000	-0.225374000
C	3.873007000	-0.287815000	-0.178337000
C	5.232739000	-0.128578000	0.082331000
H	5.828340000	-1.021264000	0.267042000
C	5.837124000	1.128667000	0.092305000
C	5.008545000	2.219986000	-0.189608000
H	5.454718000	3.205882000	-0.206062000
C	3.637357000	2.124270000	-0.457225000
C	3.042824000	0.831906000	-0.427531000
C	7.338521000	1.262829000	0.379597000
H	7.651744000	3.189888000	-0.617043000
C	2.815819000	3.380527000	-0.782861000
C	3.668839000	4.658424000	-0.796774000
H	4.463928000	4.615759000	-1.549251000
H	4.125531000	4.865581000	0.177700000
H	3.028494000	5.511615000	-1.045157000
C	2.177235000	3.237477000	-2.178543000
H	2.951670000	3.145837000	-2.948183000
H	1.573566000	4.122937000	-2.410506000
H	1.533838000	2.358415000	-2.233135000
C	1.719721000	3.567818000	0.280247000
H	1.120516000	4.457825000	0.056998000
H	2.162516000	3.700458000	1.274610000
H	1.053218000	2.705330000	0.303920000
C	8.138076000	0.488965000	-0.687403000
H	7.943531000	0.891283000	-1.687003000
H	7.873700000	-0.573196000	-0.699925000
H	9.214889000	0.562424000	-0.493016000
C	7.652725000	0.678463000	1.770987000
H	8.724336000	0.756513000	1.990641000
H	7.376633000	-0.378691000	1.838287000
H	7.105221000	1.216266000	2.552338000
C	7.809537000	2.723322000	0.361073000
H	7.293393000	3.327040000	1.115207000
H	8.882238000	2.768747000	0.578492000

C

U	-0.027382000	-0.657384000	0.033817000
I	-1.589870000	-0.573010000	-2.668592000
O	-1.656603000	0.682147000	0.390774000
O	1.597375000	0.669474000	0.161805000
N	-2.369648000	-1.929670000	1.360975000
N	-0.316260000	-3.677722000	-0.271644000
N	2.293754000	-1.796492000	1.612409000
N	0.146852000	-0.104913000	3.015365000
C	-2.208797000	-3.393448000	1.362674000
H	-3.179067000	-3.867860000	1.599045000
H	-1.529663000	-3.661076000	2.179124000
C	-1.720191000	-3.984172000	0.054447000
H	-2.339536000	-3.637567000	-0.775740000
H	-1.859938000	-5.078851000	0.100693000
C	0.537126000	-4.562208000	0.554157000
H	0.456621000	-5.583160000	0.143867000
H	0.116706000	-4.612077000	1.564691000
C	2.006565000	-4.169304000	0.666708000
H	2.575761000	-5.093560000	0.820758000
H	2.372171000	-3.745927000	-0.274807000
C	2.318142000	-3.256438000	1.852756000
H	1.607053000	-3.478483000	2.657157000
H	3.315526000	-3.512930000	2.252125000
C	2.409566000	-1.136948000	2.926225000

H	2.845426000	-0.148733000	2.771088000
H	3.126530000	-1.686830000	3.560354000
C	1.096336000	-1.024040000	3.660136000
H	0.631510000	-2.010396000	3.734527000
H	1.295207000	-0.691132000	4.695619000
C	-1.103911000	-0.108170000	3.802934000
H	-1.738505000	0.690861000	3.407105000
H	-0.850956000	0.171824000	4.841633000
C	-1.910720000	-1.404902000	3.823546000
H	-2.501722000	-1.393531000	4.747543000
H	-1.272670000	-2.290697000	3.910832000
C	-2.905001000	-1.536337000	2.683795000
H	-3.693597000	-2.256660000	2.964636000
H	-3.405867000	-0.573289000	2.561240000
C	-0.120755000	-4.074452000	-1.684552000
H	0.933087000	-4.017492000	-1.949900000
H	-0.658435000	-3.388456000	-2.341098000
H	-0.483874000	-5.104627000	-1.837365000
C	0.677451000	1.267738000	3.105330000
H	-0.067546000	1.967027000	2.728599000
H	1.576210000	1.389410000	2.505083000
H	0.901596000	1.531876000	4.152848000
C	-3.409778000	-1.581236000	0.357798000
H	-3.020360000	-1.790923000	-0.642141000
H	-4.282540000	-2.238559000	0.514414000
C	-3.886775000	-0.157897000	0.372508000
C	-5.264930000	0.043851000	0.366996000
H	-5.906446000	-0.832667000	0.427071000
C	-5.828627000	1.314233000	0.272804000
C	-4.931733000	2.376392000	0.174777000
H	-5.335015000	3.374833000	0.084067000
C	-3.535940000	2.243085000	0.182944000
C	-2.994101000	0.936664000	0.311356000
C	-7.351443000	1.492726000	0.273789000
C	-7.933966000	0.916956000	1.579892000
H	-7.530534000	1.440933000	2.453021000
H	-7.704456000	-0.147341000	1.692868000
H	-9.024773000	1.024573000	1.593054000
C	-7.957228000	0.741113000	-0.927956000
H	-9.047379000	0.857068000	-0.941975000
H	-7.737227000	-0.330285000	-0.890005000
H	-7.560180000	1.128331000	-1.871757000
C	-7.766071000	2.967093000	0.175854000
H	-8.858467000	3.044501000	0.186484000
H	-7.412658000	3.428927000	-0.751985000
H	-7.385526000	3.554552000	1.018405000
C	-2.673071000	3.503191000	0.035519000
C	-3.501564000	4.784201000	-0.160533000
H	-4.147437000	5.003052000	0.696936000
H	-4.120686000	4.743718000	-1.062742000
H	-2.815519000	5.630315000	-0.273860000
C	-1.774761000	3.357397000	-1.204514000
H	-1.152512000	4.249438000	-1.331675000
H	-2.388449000	3.241273000	-2.103419000
H	-1.124537000	2.484989000	-1.142307000
C	-1.851256000	3.695072000	1.319865000
H	-2.515250000	3.825368000	2.182325000
H	-1.209540000	4.579317000	1.248295000
H	-1.220283000	2.825541000	1.498347000
C	3.520119000	-1.460695000	0.850778000
H	3.445097000	-1.932911000	-0.133272000
H	4.378407000	-1.914911000	1.375645000

C	3.845338000	-0.002433000	0.642029000
C	5.192668000	0.330279000	0.785459000
H	5.867522000	-0.444132000	1.143496000
C	5.695232000	1.589097000	0.465119000
C	4.777035000	2.510513000	-0.035186000
H	5.139047000	3.488080000	-0.320657000
C	3.409389000	2.251911000	-0.195920000
C	2.925863000	0.975671000	0.193901000
C	7.186389000	1.897669000	0.646133000
H	7.315573000	3.568507000	-0.767378000
C	2.510220000	3.342477000	-0.796644000
C	3.296393000	4.597445000	-1.214080000
H	4.061663000	4.375197000	-1.965014000
H	3.774788000	5.096779000	-0.364081000
H	2.599675000	5.314081000	-1.661248000
C	1.820217000	2.810470000	-2.067303000
H	2.566934000	2.559932000	-2.827260000
H	1.154298000	3.574086000	-2.484147000
H	1.234931000	1.909136000	-1.884228000
C	1.484887000	3.788459000	0.256653000
H	0.859893000	4.595745000	-0.138500000
H	1.992503000	4.164665000	1.152809000
H	0.832775000	2.966971000	0.548401000
C	8.014646000	0.964709000	-0.259453000
H	7.758550000	1.115327000	-1.313099000
H	7.839989000	-0.089550000	-0.023564000
H	9.086032000	1.162181000	-0.135733000
C	7.584409000	1.665250000	2.117093000
H	8.650883000	1.873204000	2.262370000
H	7.403060000	0.632187000	2.429642000
H	7.016086000	2.321310000	2.785097000
C	7.530871000	3.348533000	0.283410000
H	6.982120000	4.065185000	0.903997000
H	8.600139000	3.523389000	0.442835000
I	2.084745000	-1.424505000	-2.341751000

Int2

U	0.006181000	0.490293000	0.628734000
O	1.704977000	-0.860797000	0.004722000
O	-1.683300000	-0.894994000	-0.011019000
N	2.316728000	1.280427000	2.118503000
N	0.014848000	3.361756000	1.520308000
N	-2.258565000	0.838772000	2.385501000
N	-0.044386000	-1.224938000	3.026295000
C	2.169264000	2.654141000	2.626378000
H	3.169716000	3.077892000	2.835702000
H	1.657908000	2.608216000	3.592899000
C	1.452830000	3.624098000	1.698782000
H	1.916113000	3.603802000	0.709158000
H	1.607232000	4.645832000	2.093824000
C	-0.692134000	3.761689000	2.749286000
H	-0.609915000	4.859413000	2.857050000
H	-0.172150000	3.331299000	3.611196000
C	-2.160139000	3.361114000	2.840372000
H	-2.624349000	4.034038000	3.572016000
H	-2.686819000	3.551617000	1.900538000
C	-2.419130000	1.946074000	3.345572000
H	-1.751722000	1.766187000	4.195743000
H	-3.448180000	1.905870000	3.748478000
C	-2.418967000	-0.419409000	3.134161000
H	-2.696975000	-1.205406000	2.431705000
H	-3.251882000	-0.345166000	3.855249000

C	-1.159808000	-0.810920000	3.892612000
H	-0.819785000	0.024139000	4.512701000
H	-1.413565000	-1.629557000	4.591383000
C	1.178257000	-1.355806000	3.844526000
H	1.883004000	-1.973140000	3.278554000
H	0.930875000	-1.927302000	4.757774000
C	1.889936000	-0.058920000	4.235651000
H	2.459049000	-0.272793000	5.148881000
H	1.172363000	0.720081000	4.521926000
C	2.889635000	0.455914000	3.199023000
H	3.685262000	1.028274000	3.708697000
H	3.384312000	-0.399689000	2.732180000
C	-0.461856000	4.208911000	0.415323000
H	-1.513550000	4.014453000	0.202026000
H	0.103369000	3.975917000	-0.486362000
H	-0.341868000	5.278895000	0.658730000
C	-0.337743000	-2.553384000	2.466508000
H	0.495805000	-2.869458000	1.839489000
H	-1.226674000	-2.516816000	1.838728000
H	-0.485374000	-3.300501000	3.265602000
C	3.242184000	1.312915000	0.941568000
H	2.677716000	1.732072000	0.101734000
H	4.065140000	2.014403000	1.161844000
C	3.849010000	0.010713000	0.501469000
C	5.235479000	-0.123412000	0.543686000
H	5.814800000	0.687957000	0.982064000
C	5.885407000	-1.244725000	0.028834000
C	5.069225000	-2.213403000	-0.561016000
H	5.546430000	-3.080753000	-0.997388000
C	3.672770000	-2.136523000	-0.636276000
C	3.029762000	-1.008478000	-0.048726000
C	7.413668000	-1.357817000	0.101274000
C	7.865485000	-1.298774000	1.573808000
H	7.434563000	-2.126533000	2.147161000
H	7.556482000	-0.364725000	2.053686000
H	8.957740000	-1.365575000	1.647126000
C	8.055554000	-0.189093000	-0.672393000
H	9.149763000	-0.246813000	-0.623359000
H	7.754620000	0.780423000	-0.262994000
H	7.758914000	-0.210633000	-1.726147000
C	7.932413000	-2.670464000	-0.502123000
H	9.023964000	-2.710155000	-0.417810000
H	7.680254000	-2.758226000	-1.564108000
H	7.528370000	-3.545284000	0.018634000
C	2.884843000	-3.236550000	-1.364848000
C	3.792435000	-4.318061000	-1.972157000
H	4.366053000	-4.857628000	-1.209997000
H	4.493979000	-3.906157000	-2.705601000
H	3.169185000	-5.053839000	-2.492017000
C	2.078579000	-2.611855000	-2.519425000
H	1.495822000	-3.383967000	-3.036018000
H	2.748428000	-2.146148000	-3.249950000
H	1.392183000	-1.847898000	-2.153138000
C	1.934387000	-3.935339000	-0.379785000
H	2.492234000	-4.374155000	0.455755000
H	1.381935000	-4.739545000	-0.879069000
H	1.214602000	-3.218246000	0.012578000
C	-3.286634000	1.007883000	1.305865000
H	-2.834657000	1.644664000	0.530978000
H	-4.136874000	1.574002000	1.715841000
C	-3.846386000	-0.229262000	0.659290000
C	-5.222219000	-0.445315000	0.709427000

H	-5.827899000	0.243491000	1.296341000
C	-5.828808000	-1.497300000	0.022391000
C	-4.984362000	-2.303771000	-0.747112000
H	-5.430928000	-3.107944000	-1.317107000
C	-3.596402000	-2.140124000	-0.836955000
C	-2.995851000	-1.097429000	-0.073192000
C	-7.344746000	-1.714768000	0.116460000
H	-7.599039000	-2.780375000	-1.782368000
C	-2.767137000	-3.049091000	-1.757301000
C	-3.634338000	-4.045196000	-2.543848000
H	-4.367402000	-3.541455000	-3.183434000
H	-4.168333000	-4.741222000	-1.887182000
H	-2.987654000	-4.642907000	-3.195081000
C	-2.011002000	-2.186637000	-2.787342000
H	-2.715957000	-1.633871000	-3.417959000
H	-1.400550000	-2.822741000	-3.439041000
H	-1.355131000	-1.467779000	-2.294044000
C	-1.767298000	-3.867802000	-0.923274000
H	-1.175648000	-4.523185000	-1.572609000
H	-2.292660000	-4.498603000	-0.196605000
H	-1.083183000	-3.209539000	-0.389165000
C	-8.082448000	-0.460610000	-0.392608000
H	-7.824627000	-0.254267000	-1.436660000
H	-7.827250000	0.427375000	0.194360000
H	-9.168575000	-0.598883000	-0.330450000
C	-7.735971000	-1.972172000	1.585126000
H	-8.818833000	-2.118796000	1.677716000
H	-7.457795000	-1.133044000	2.230713000
H	-7.236885000	-2.867955000	1.969736000
C	-7.814948000	-2.914591000	-0.717132000
H	-7.344836000	-3.847336000	-0.387717000
H	-8.898564000	-3.035825000	-0.613008000
N	0.673437000	1.864715000	-1.408030000
N	-0.653654000	1.675979000	-1.427190000
C	-3.431084000	3.423935000	-4.061245000
C	-2.139385000	3.947115000	-3.981377000
C	-3.762172000	2.313575000	-3.280914000
C	-1.184732000	3.374499000	-3.148468000
C	-2.822909000	1.735894000	-2.436659000
C	-1.511789000	2.251325000	-2.356476000
C	3.126853000	1.954886000	-4.836001000
C	3.488552000	2.634154000	-3.672921000
C	1.920750000	1.251329000	-4.865132000
C	2.658070000	2.613283000	-2.557350000
C	1.081483000	1.227651000	-3.758272000
C	1.438441000	1.912905000	-2.579394000
H	-4.167480000	3.874664000	-4.720340000
H	-1.868266000	4.815182000	-4.577438000
H	-4.762186000	1.890335000	-3.330542000
H	-0.187126000	3.796618000	-3.103072000
H	-3.080108000	0.867182000	-1.839411000
H	3.776335000	1.969513000	-5.706391000
H	4.423579000	3.187122000	-3.633064000
H	1.631360000	0.707970000	-5.761109000
H	2.937937000	3.156970000	-1.659109000
H	0.153705000	0.666412000	-3.786492000

TS1

U	-0.005977000	-0.660393000	-0.437830000
O	1.682967000	0.815433000	-0.302708000
O	-1.629262000	0.889150000	-0.287038000
N	2.306034000	-1.629295000	-1.894914000

N	0.173445000	-3.650441000	-0.838241000
N	-2.270159000	-1.397140000	-2.108724000
N	-0.159697000	0.563634000	-3.310391000
C	2.189893000	-3.062133000	-2.209774000
H	3.190053000	-3.469830000	-2.449482000
H	1.592926000	-3.171885000	-3.120276000
C	1.598475000	-3.911000000	-1.099346000
H	2.145595000	-3.740054000	-0.169465000
H	1.751829000	-4.975163000	-1.356109000
C	-0.621665000	-4.326203000	-1.877322000
H	-0.526001000	-5.418493000	-1.734015000
H	-0.179238000	-4.107968000	-2.855469000
C	-2.098917000	-3.953744000	-1.931286000
H	-2.619170000	-4.789225000	-2.415548000
H	-2.530254000	-3.886426000	-0.928181000
C	-2.420067000	-2.713386000	-2.758459000
H	-1.786072000	-2.730341000	-3.652362000
H	-3.461071000	-2.796845000	-3.121083000
C	-2.478547000	-0.377111000	-3.153458000
H	-2.788431000	0.549793000	-2.671931000
H	-3.312897000	-0.669362000	-3.814656000
C	-1.245601000	-0.136823000	-4.009634000
H	-0.856349000	-1.088896000	-4.384273000
H	-1.551548000	0.441561000	-4.902363000
C	1.039472000	0.570487000	-4.167060000
H	1.705344000	1.354347000	-3.791792000
H	0.747607000	0.876291000	-5.189385000
C	1.831941000	-0.733690000	-4.237791000
H	2.413446000	-0.700276000	-5.167149000
H	1.162006000	-1.594088000	-4.351343000
C	2.826371000	-0.943024000	-3.094176000
H	3.699539000	-1.507048000	-3.468988000
H	3.208607000	0.029326000	-2.774573000
C	-0.155435000	-4.251784000	0.462566000
H	-1.196550000	-4.067224000	0.726315000
H	0.458194000	-3.785646000	1.231791000
H	0.025784000	-5.341026000	0.449201000
C	-0.549113000	1.964139000	-3.096267000
H	0.269545000	2.495338000	-2.610737000
H	-1.414537000	2.029935000	-2.439794000
H	-0.776105000	2.466591000	-4.053345000
C	3.289730000	-1.485603000	-0.777838000
H	2.789248000	-1.819829000	0.137316000
H	4.134048000	-2.171062000	-0.966214000
C	3.854984000	-0.114323000	-0.534847000
C	5.241440000	0.022531000	-0.527838000
H	5.840736000	-0.852825000	-0.772925000
C	5.866705000	1.225910000	-0.204391000
C	5.022046000	2.290738000	0.115313000
H	5.475596000	3.236003000	0.381789000
C	3.623048000	2.220127000	0.117734000
C	3.008583000	0.982317000	-0.231954000
C	7.397097000	1.329559000	-0.197996000
C	7.944666000	0.990158000	-1.598411000
H	7.560460000	1.690423000	-2.347784000
H	7.661660000	-0.020197000	-1.910504000
H	9.039846000	1.046696000	-1.609357000
C	7.976158000	0.334114000	0.826868000
H	9.071655000	0.384287000	0.837941000
H	7.692318000	-0.697146000	0.594260000
H	7.613446000	0.558520000	1.835286000
C	7.886366000	2.735605000	0.174905000

H	8.981422000	2.761974000	0.161573000
H	7.561874000	3.026048000	1.179697000
H	7.528798000	3.491812000	-0.532222000
C	2.803017000	3.462987000	0.494575000
C	3.678300000	4.663314000	0.889873000
H	4.322608000	4.999118000	0.069709000
H	4.308825000	4.447130000	1.759221000
H	3.027713000	5.502935000	1.157722000
C	1.894848000	3.144172000	1.695526000
H	1.304504000	4.026412000	1.969674000
H	2.493128000	2.851390000	2.564703000
H	1.210885000	2.328515000	1.462998000
C	1.956023000	3.890726000	-0.715561000
H	2.598152000	4.136933000	-1.569283000
H	1.352896000	4.773961000	-0.476948000
H	1.285075000	3.080896000	-1.001042000
C	-3.303820000	-1.300430000	-1.025994000
H	-2.875074000	-1.769962000	-0.131594000
H	-4.175066000	-1.906976000	-1.319336000
C	-3.819739000	0.066741000	-0.661204000
C	-5.197868000	0.271410000	-0.697666000
H	-5.823424000	-0.532583000	-1.081460000
C	-5.784758000	1.454301000	-0.248461000
C	-4.915636000	2.415118000	0.273994000
H	-5.342814000	3.330447000	0.661380000
C	-3.523089000	2.274176000	0.333914000
C	-2.945797000	1.083410000	-0.195134000
C	-7.304801000	1.646130000	-0.329903000
H	-7.494753000	3.119177000	1.282649000
C	-2.669057000	3.368670000	0.993102000
C	-3.514031000	4.524075000	1.552846000
H	-4.228115000	4.186221000	2.311759000
H	-4.067191000	5.053776000	0.769105000
H	-2.849163000	5.251860000	2.030468000
C	-1.890358000	2.758378000	2.174478000
H	-2.580153000	2.381789000	2.937598000
H	-1.250297000	3.515878000	2.641105000
H	-1.260130000	1.931124000	1.843613000
C	-1.690835000	3.969808000	-0.029725000
H	-1.094529000	4.762477000	0.436651000
H	-2.234812000	4.409719000	-0.873719000
H	-1.010924000	3.208245000	-0.408692000
C	-8.012969000	0.542369000	0.480283000
H	-7.717724000	0.581632000	1.533936000
H	-7.770846000	-0.456759000	0.104509000
H	-9.101610000	0.662586000	0.425986000
C	-7.752322000	1.558896000	-1.802520000
H	-8.839030000	1.681256000	-1.885180000
H	-7.490790000	0.592959000	-2.246072000
H	-7.274714000	2.341144000	-2.401995000
C	-7.752976000	3.005441000	0.224461000
H	-7.305387000	3.838355000	-0.328246000
H	-8.841045000	3.099036000	0.138217000
N	1.040198000	-1.323333000	1.594686000
N	-0.831998000	-1.387623000	1.417548000
C	-3.138642000	-2.490519000	4.749053000
C	-1.925003000	-3.137405000	4.507938000
C	-3.540015000	-1.460418000	3.895756000
C	-1.127561000	-2.777828000	3.429096000
C	-2.745287000	-1.078917000	2.822472000
C	-1.519462000	-1.738298000	2.548837000
C	2.625849000	-0.848825000	5.428464000

C	3.138985000	-1.814986000	4.555313000
C	1.560923000	-0.042397000	5.009886000
C	2.608544000	-1.971478000	3.285555000
C	0.999834000	-0.198539000	3.753783000
C	1.515536000	-1.167943000	2.843994000
H	-3.759326000	-2.781884000	5.591658000
H	-1.595354000	-3.935092000	5.169366000
H	-4.479609000	-0.942311000	4.071670000
H	-0.181117000	-3.280875000	3.269404000
H	-3.055223000	-0.273044000	2.165070000
H	3.053632000	-0.722281000	6.418606000
H	3.969988000	-2.442248000	4.868650000
H	1.161914000	0.715706000	5.679122000
H	3.014882000	-2.714141000	2.604356000
H	0.173335000	0.424227000	3.430045000

Int3

U	-0.032686000	-0.659732000	-0.359238000
O	1.536693000	0.849972000	-0.037904000
O	-1.439555000	0.923100000	0.260841000
N	2.299315000	-0.883815000	-2.221223000
N	0.114112000	-2.981771000	-2.158906000
N	-1.927492000	0.041806000	-2.504981000
N	0.318771000	2.338884000	-3.374121000
C	2.261863000	-2.079378000	-3.069066000
H	3.283857000	-2.404477000	-3.336906000
H	1.771126000	-1.829123000	-4.011012000
C	1.543463000	-3.244743000	-2.412531000
H	2.010229000	-3.481885000	-1.453511000
H	1.658318000	-4.139817000	-3.050493000
C	-0.608283000	-3.018725000	-3.448653000
H	-0.717608000	-4.074839000	-3.750927000
H	0.014259000	-2.550644000	-4.216424000
C	-1.974448000	-2.344449000	-3.481461000
H	-2.505916000	-2.770839000	-4.340909000
H	-2.566836000	-2.626494000	-2.607775000
C	-1.950424000	-0.825244000	-3.702347000
H	-1.078423000	-0.595666000	-4.319610000
H	-2.836507000	-0.547788000	-4.303659000
C	-2.011309000	1.467339000	-2.929347000
H	-1.981415000	2.070625000	-2.019469000
H	-3.007520000	1.620754000	-3.382347000
C	-0.986870000	1.996217000	-3.928620000
H	-0.850706000	1.284721000	-4.747872000
H	-1.443973000	2.885344000	-4.402612000
C	1.392188000	2.063666000	-4.324975000
H	2.256961000	2.686606000	-4.068488000
H	1.111518000	2.338611000	-5.358443000
C	1.785813000	0.587798000	-4.241314000
H	2.304232000	0.276042000	-5.156037000
H	0.861356000	0.003225000	-4.188079000
C	2.642647000	0.324971000	-2.999637000
H	3.706876000	0.287026000	-3.290762000
H	2.527084000	1.164298000	-2.312578000
C	-0.397442000	-4.077978000	-1.318231000
H	-1.468492000	-3.964272000	-1.155531000
H	0.086549000	-4.049419000	-0.342338000
H	-0.207985000	-5.054841000	-1.795874000
C	0.335531000	3.704159000	-2.874622000
H	1.294145000	3.913560000	-2.394783000
H	-0.441274000	3.834815000	-2.116157000
H	0.165302000	4.456106000	-3.669026000

C	3.353627000	-1.099787000	-1.200022000
H	3.015527000	-1.888973000	-0.525353000
H	4.254892000	-1.475279000	-1.716198000
C	3.776505000	0.099269000	-0.396049000
C	5.149863000	0.259499000	-0.219631000
H	5.808905000	-0.460182000	-0.701995000
C	5.691689000	1.290349000	0.543500000
C	4.775992000	2.174671000	1.112157000
H	5.159349000	2.992842000	1.705811000
C	3.386807000	2.076974000	0.965501000
C	2.859274000	1.000674000	0.194472000
C	7.209729000	1.413983000	0.720814000
C	7.880150000	1.572776000	-0.657875000
H	7.522838000	2.476129000	-1.163526000
H	7.670302000	0.719839000	-1.310740000
H	8.968677000	1.649791000	-0.550377000
C	7.748779000	0.141814000	1.404615000
H	8.836106000	0.204177000	1.533021000
H	7.533140000	-0.755683000	0.816585000
H	7.295002000	0.007727000	2.391959000
C	7.601035000	2.622171000	1.582850000
H	8.691003000	2.667520000	1.681639000
H	7.180898000	2.555548000	2.591977000
H	7.270864000	3.566173000	1.136045000
C	2.493495000	3.101232000	1.677327000
C	3.291864000	4.197850000	2.402407000
H	3.924918000	4.772896000	1.717605000
H	3.921749000	3.796798000	3.203653000
H	2.587073000	4.898014000	2.863751000
C	1.672595000	2.359792000	2.745598000
H	1.064925000	3.061624000	3.325008000
H	2.342790000	1.839647000	3.438686000
H	1.008665000	1.624926000	2.290947000
C	1.589616000	3.810404000	0.654261000
H	2.205618000	4.364430000	-0.064045000
H	0.932775000	4.530158000	1.154828000
H	0.975720000	3.095097000	0.106385000
C	-3.195063000	-0.238002000	-1.780736000
H	-3.118674000	-1.247790000	-1.377436000
H	-4.010289000	-0.238335000	-2.526433000
C	-3.626041000	0.673447000	-0.661426000
C	-4.993166000	0.951073000	-0.619292000
H	-5.603338000	0.615725000	-1.455962000
C	-5.592432000	1.609988000	0.451450000
C	-4.742713000	1.978310000	1.494055000
H	-5.174505000	2.474514000	2.352064000
C	-3.360458000	1.755334000	1.503138000
C	-2.769518000	1.104295000	0.381738000
C	-7.099994000	1.891423000	0.449804000
H	-7.360859000	2.020965000	2.621512000
C	-2.537402000	2.247853000	2.700855000
C	-3.405127000	2.821740000	3.834064000
H	-4.114395000	2.085058000	4.226976000
H	-3.963487000	3.712641000	3.526506000
H	-2.751395000	3.120132000	4.660671000
C	-1.733032000	1.085697000	3.312674000
H	-2.410070000	0.316540000	3.699359000
H	-1.116928000	1.445533000	4.144696000
H	-1.079694000	0.615089000	2.578753000
C	-1.621070000	3.380799000	2.210962000
H	-1.025593000	3.782794000	3.037973000
H	-2.223470000	4.200538000	1.802665000

H	-0.945237000	3.029901000	1.431808000
C	-7.875149000	0.562800000	0.350390000
H	-7.647229000	-0.085435000	1.202925000
H	-7.625423000	0.012768000	-0.562468000
H	-8.956047000	0.747204000	0.341892000
C	-7.454860000	2.777598000	-0.760648000
H	-8.531968000	2.981470000	-0.786494000
H	-7.184122000	2.298312000	-1.706865000
H	-6.927624000	3.736074000	-0.710250000
C	-7.558852000	2.615066000	1.723090000
H	-7.066813000	3.586556000	1.839782000
H	-8.638350000	2.795275000	1.677670000
N	1.090278000	-1.691811000	0.847249000
N	-1.483139000	-1.763294000	0.284543000
C	-3.821417000	-4.247353000	2.748217000
C	-2.432612000	-4.384034000	2.720069000
C	-4.423130000	-3.263152000	1.964354000
C	-1.655495000	-3.562138000	1.912066000
C	-3.656618000	-2.431828000	1.153198000
C	-2.252911000	-2.571774000	1.102338000
C	3.187660000	-3.556181000	3.973221000
C	3.133675000	-4.147482000	2.711771000
C	2.531631000	-2.340548000	4.183242000
C	2.438209000	-3.536270000	1.673270000
C	1.835382000	-1.720766000	3.154296000
C	1.773722000	-2.303592000	1.867535000
H	-4.425061000	-4.892290000	3.380671000
H	-1.947156000	-5.135707000	3.337753000
H	-5.502331000	-3.132766000	1.989135000
H	-0.575007000	-3.656907000	1.907223000
H	-4.132390000	-1.645866000	0.576043000
H	3.730360000	-4.035625000	4.782711000
H	3.634934000	-5.095842000	2.533510000
H	2.562595000	-1.870416000	5.163045000
H	2.392449000	-4.012259000	0.697462000
H	1.325503000	-0.777598000	3.320620000

TS2

U	-0.126739000	-0.752263000	-0.286923000
O	-1.517190000	0.951312000	-0.146864000
O	1.703484000	0.561653000	-0.515387000
N	-1.369444000	-1.527674000	2.078321000
N	1.972582000	-1.902846000	1.176630000
N	1.290207000	-2.769648000	-1.733492000
N	-2.037022000	-2.346926000	-0.699697000
C	-0.817671000	-2.672362000	2.823122000
H	-0.916817000	-3.564547000	2.194869000
H	-1.449969000	-2.844052000	3.713290000
C	0.637324000	-2.557572000	3.272197000
H	0.769682000	-3.289561000	4.078729000
H	0.839287000	-1.587211000	3.736028000
C	1.691588000	-2.902686000	2.225223000
H	2.638241000	-3.129577000	2.749101000
H	1.382287000	-3.832711000	1.735601000
C	2.883588000	-2.527725000	0.197845000
H	3.672754000	-3.101630000	0.713908000
H	3.397408000	-1.730364000	-0.341637000
C	2.175259000	-3.457069000	-0.777180000
H	1.574155000	-4.193518000	-0.234534000
H	2.943650000	-4.033590000	-1.324035000
C	0.469546000	-3.777997000	-2.441660000
H	1.120271000	-4.621253000	-2.734924000

H	0.127300000	-3.314699000	-3.373862000
C	-0.764573000	-4.306840000	-1.707205000
H	-0.532164000	-4.595312000	-0.674125000
H	-1.047323000	-5.239138000	-2.216044000
C	-1.972807000	-3.355629000	-1.749182000
H	-1.934074000	-2.831544000	-2.717678000
H	-2.902876000	-3.951974000	-1.758679000
C	-2.796013000	-2.830988000	0.448101000
H	-2.441669000	-3.831460000	0.741450000
H	-3.862644000	-2.959486000	0.186663000
C	-2.738996000	-1.865041000	1.623198000
H	-3.224781000	-0.934841000	1.323038000
H	-3.320053000	-2.279323000	2.465625000
C	-1.466824000	-0.354432000	2.961549000
H	-0.477048000	-0.026257000	3.286907000
H	-2.070164000	-0.578659000	3.857101000
H	-1.930296000	0.467787000	2.416569000
C	2.588408000	-0.690318000	1.818414000
H	1.753310000	-0.077156000	2.190337000
H	3.163914000	-1.012977000	2.699984000
C	3.487567000	0.162590000	0.965746000
C	4.820423000	0.345456000	1.346378000
H	5.181555000	-0.191445000	2.219558000
C	5.667903000	1.194618000	0.640888000
C	5.109487000	1.873638000	-0.453865000
H	5.749545000	2.558468000	-0.999517000
C	3.788542000	1.735432000	-0.881754000
C	2.957294000	0.824023000	-0.165772000
C	7.138359000	1.420483000	1.015689000
C	7.561956000	0.579992000	2.228209000
H	6.982795000	0.834917000	3.122401000
H	8.617306000	0.763659000	2.456503000
H	7.446980000	-0.493018000	2.038957000
C	7.363303000	2.905518000	1.362420000
H	7.117263000	3.560175000	0.520899000
H	8.411793000	3.085495000	1.628587000
H	6.739637000	3.206360000	2.210720000
C	8.041536000	1.033206000	-0.171807000
H	7.913132000	-0.023077000	-0.431419000
H	9.096866000	1.197190000	0.076708000
H	7.814902000	1.624571000	-1.064209000
C	3.239692000	2.571778000	-2.049333000
C	2.068715000	3.437125000	-1.542968000
H	1.273936000	2.817254000	-1.125688000
H	1.650913000	4.029249000	-2.365721000
H	2.408619000	4.131105000	-0.766428000
C	2.751538000	1.659675000	-3.190667000
H	3.571792000	1.041140000	-3.573696000
H	2.372439000	2.266094000	-4.021795000
H	1.946879000	1.005588000	-2.851627000
C	4.296284000	3.517910000	-2.640312000
H	4.672671000	4.231756000	-1.899598000
H	3.843575000	4.097334000	-3.451961000
H	5.149838000	2.974958000	-3.061892000
C	2.099066000	-2.057918000	-2.736718000
H	1.435300000	-1.516100000	-3.417210000
H	2.717675000	-2.756938000	-3.324825000
H	2.749252000	-1.323978000	-2.260730000
C	-2.852006000	1.136212000	-0.207922000
C	-3.485561000	2.286147000	0.301041000
C	-4.894690000	2.294537000	0.351743000
H	-5.383510000	3.166209000	0.763793000

C	-5.689843000	1.226671000	-0.094975000
C	-5.043948000	0.136582000	-0.667238000
H	-5.622859000	-0.691683000	-1.072632000
C	-3.634274000	0.081005000	-0.792720000
C	-3.004555000	-0.969799000	-1.542106000
H	-2.163402000	-0.658052000	-2.196553000
H	-3.705662000	-1.617192000	-2.069621000
C	-2.679001000	3.524291000	0.737307000
C	-3.582993000	4.685894000	1.180033000
H	-4.259202000	5.006831000	0.380800000
H	-2.958854000	5.545146000	1.449581000
H	-4.186494000	4.429415000	2.058018000
C	-1.739105000	3.203892000	1.914460000
H	-2.310254000	2.865839000	2.787448000
H	-1.180993000	4.101003000	2.209941000
H	-1.020431000	2.430752000	1.637410000
C	-1.843286000	4.017914000	-0.460618000
H	-1.164873000	3.237319000	-0.809189000
H	-1.247835000	4.894966000	-0.177780000
H	-2.495953000	4.304358000	-1.292347000
C	-7.223672000	1.240263000	0.004672000
C	-7.757558000	2.511363000	0.680184000
H	-7.372617000	2.621422000	1.699651000
H	-8.850861000	2.468305000	0.739955000
H	-7.492898000	3.412452000	0.117028000
C	-7.696548000	0.029619000	0.833174000
H	-7.381772000	-0.916301000	0.381392000
H	-8.790709000	0.015216000	0.911566000
H	-7.283542000	0.068354000	1.847066000
C	-7.837394000	1.157921000	-1.406786000
H	-7.521613000	2.010221000	-2.017451000
H	-8.933200000	1.161958000	-1.354214000
H	-7.530512000	0.245670000	-1.927768000
2			
U	-0.099014000	-1.404070000	-0.209315000
O	-1.851377000	-0.831918000	0.982902000
O	0.878910000	0.545176000	0.071883000
N	0.137717000	-3.080561000	1.966045000
N	2.698030000	-1.743165000	0.073551000
N	1.186531000	-1.682015000	-2.589207000
N	-0.621572000	-3.584281000	-0.668123000
C	1.209456000	-4.090211000	1.948729000
H	1.047127000	-4.723342000	1.072837000
H	1.102704000	-4.732536000	2.841235000
C	2.641163000	-3.566526000	1.890939000
H	3.284022000	-4.418425000	2.146168000
H	2.834254000	-2.826516000	2.673647000
C	3.134305000	-3.080541000	0.526898000
H	4.239746000	-3.091286000	0.544640000
H	2.819484000	-3.811227000	-0.225039000
C	3.295878000	-1.477919000	-1.249317000
H	4.352913000	-1.796240000	-1.268109000
H	3.302134000	-0.396836000	-1.401621000
C	2.564964000	-2.170526000	-2.388970000
H	2.524092000	-3.245166000	-2.202818000
H	3.149292000	-2.033178000	-3.315490000
C	0.453079000	-2.576725000	-3.519192000
H	1.018594000	-2.623941000	-4.467020000
H	-0.500434000	-2.085773000	-3.739987000
C	0.175975000	-3.995221000	-3.032460000
H	1.095731000	-4.486548000	-2.692617000

H	-0.145791000	-4.557133000	-3.919383000
C	-0.907483000	-4.169382000	-1.969818000
H	-1.865816000	-3.795119000	-2.379781000
H	-1.046161000	-5.259057000	-1.859764000
C	-1.260640000	-4.387321000	0.361315000
H	-0.829294000	-5.407510000	0.382129000
H	-2.337384000	-4.537743000	0.160987000
C	-1.163264000	-3.750206000	1.746600000
H	-1.930916000	-2.980354000	1.851019000
H	-1.338852000	-4.514368000	2.524338000
C	0.098557000	-2.378076000	3.253506000
H	1.032986000	-1.839078000	3.430111000
H	-0.058719000	-3.078172000	4.090714000
H	-0.716827000	-1.652969000	3.240682000
C	3.144091000	-0.726223000	1.073576000
H	2.477687000	-0.824008000	1.939686000
H	4.157805000	-0.987036000	1.419764000
C	3.155425000	0.711408000	0.631658000
C	4.335940000	1.451236000	0.740936000
H	5.232677000	0.937505000	1.076265000
C	4.370040000	2.810337000	0.442998000
C	3.160860000	3.398367000	0.041687000
H	3.165710000	4.459815000	-0.179256000
C	1.949218000	2.715287000	-0.086085000
C	1.959938000	1.321379000	0.199248000
C	5.640844000	3.663073000	0.551085000
C	6.852676000	2.839731000	1.008432000
H	6.694704000	2.399880000	1.999139000
H	7.736071000	3.483907000	1.071352000
H	7.083318000	2.031190000	0.306102000
C	5.418451000	4.794753000	1.574030000
H	4.589507000	5.447267000	1.284064000
H	6.317154000	5.416867000	1.660483000
H	5.189540000	4.386490000	2.563978000
C	5.970686000	4.278251000	-0.823156000
H	6.152365000	3.496474000	-1.568411000
H	6.869421000	4.903030000	-0.758875000
H	5.154759000	4.906873000	-1.192569000
C	0.659685000	3.457119000	-0.475483000
C	-0.372576000	3.313629000	0.659771000
H	-0.605893000	2.266972000	0.856747000
H	-1.306918000	3.816289000	0.387315000
H	0.004733000	3.768223000	1.582772000
C	0.075946000	2.884795000	-1.780765000
H	0.800835000	2.965290000	-2.599765000
H	-0.819973000	3.446670000	-2.066123000
H	-0.214583000	1.840901000	-1.660522000
C	0.898342000	4.958848000	-0.699601000
H	1.277327000	5.457052000	0.199496000
H	-0.052948000	5.434517000	-0.959425000
H	1.595892000	5.151564000	-1.522824000
C	1.221595000	-0.343038000	-3.205486000
H	0.200792000	0.023284000	-3.317991000
H	1.707966000	-0.380416000	-4.194018000
H	1.760474000	0.364207000	-2.574505000
C	-2.739755000	0.092891000	0.518200000
C	-3.553509000	0.879576000	1.359029000
C	-4.367411000	1.846068000	0.743330000
H	-4.989066000	2.463036000	1.378634000
C	-4.409035000	2.054228000	-0.637098000
C	-3.612480000	1.223908000	-1.436248000
H	-3.634695000	1.323456000	-2.520109000

C	-2.797382000	0.233943000	-0.890992000
C	-1.952543000	-0.704730000	-1.693270000
H	-1.902775000	-0.380191000	-2.741585000
H	-2.441228000	-1.698432000	-1.698915000
C	-3.551018000	0.708561000	2.886882000
C	-4.569465000	1.621267000	3.587540000
H	-4.351855000	2.682338000	3.425767000
H	-4.534168000	1.440460000	4.667939000
H	-5.593685000	1.425235000	3.252545000
C	-3.919014000	-0.743847000	3.245994000
H	-4.929081000	-0.982132000	2.895282000
H	-3.894085000	-0.889998000	4.333210000
H	-3.226389000	-1.447225000	2.781674000
C	-2.159100000	1.057651000	3.448647000
H	-1.380020000	0.472460000	2.956537000
H	-2.117628000	0.866563000	4.528848000
H	-1.934766000	2.116878000	3.285596000
C	-5.310547000	3.111198000	-1.291639000
C	-6.081816000	3.947013000	-0.260804000
H	-6.749569000	3.327490000	0.347141000
H	-6.699628000	4.692028000	-0.774628000
H	-5.406139000	4.483773000	0.413802000
C	-6.338357000	2.413298000	-2.204983000
H	-5.848310000	1.832931000	-2.992896000
H	-6.992717000	3.149855000	-2.687719000
H	-6.965735000	1.725166000	-1.628620000
C	-4.456094000	4.073872000	-2.138811000
H	-3.726187000	4.598347000	-1.512976000
H	-5.089040000	4.825407000	-2.626705000
H	-3.904246000	3.543641000	-2.920867000

LUC6H6UL complex (s=2)

U	3.830446000	8.827230000	11.685739000
O	4.010036000	6.827908000	12.589393000
O	5.529797000	9.249462000	10.344299000
N	0.692672000	5.800207000	11.642751000
N	4.048522000	6.889877000	9.364226000
N	1.949805000	9.035983000	9.521817000
C	6.202337000	9.183289000	9.184024000
N	1.017903000	4.266403000	9.045191000
C	0.083523000	8.126224000	10.965093000
H	-0.777047000	8.803802000	10.917935000
H	0.742201000	8.529888000	11.743496000
C	6.965799000	10.267704000	8.661921000
C	3.581591000	9.395361000	14.279090000
H	3.715413000	8.581028000	14.981233000
C	7.518024000	8.913005000	6.663895000
C	7.129830000	11.589670000	9.424565000
C	2.755252000	8.768077000	8.310459000
H	2.154590000	8.981647000	7.406946000
H	3.596326000	9.468824000	8.310319000
C	7.584273000	10.088747000	7.422052000
H	8.160312000	10.915472000	7.021556000
C	1.239183000	6.055752000	12.991360000
H	0.438185000	5.901465000	13.742057000
H	1.506948000	7.114306000	13.038881000
C	-0.403532000	6.731693000	11.372203000
H	-1.081177000	6.811970000	12.243589000
H	-1.007297000	6.328074000	10.552399000
C	6.149779000	7.979746000	8.451720000
C	5.487798000	6.769745000	9.043323000
H	5.990151000	6.503243000	9.978622000

H	5.643162000	5.927128000	8.349881000
C	1.409892000	10.395713000	9.392131000
H	0.823348000	10.658540000	10.271658000
H	0.764783000	10.478659000	8.500997000
H	2.227842000	11.113238000	9.299202000
C	4.749633000	10.032814000	13.694696000
H	5.740169000	9.827629000	14.083290000
C	6.296225000	5.415756000	13.865298000
C	7.749451000	11.302963000	10.806739000
H	7.153383000	10.581776000	11.367634000
H	7.829348000	12.228935000	11.389639000
H	8.759045000	10.892992000	10.690524000
C	2.298571000	9.534401000	13.612570000
H	1.432810000	9.053464000	14.047247000
C	3.773620000	5.698827000	13.270415000
C	4.501102000	3.744824000	14.511989000
H	5.308268000	3.164476000	14.937638000
C	2.185934000	4.062727000	14.138823000
H	1.145683000	3.774117000	14.279403000
C	6.791081000	7.862551000	7.216345000
H	6.717765000	6.910117000	6.698356000
C	2.435259000	5.242954000	13.436056000
C	0.299374000	4.406516000	11.465452000
H	-0.587359000	4.144494000	12.079422000
H	1.116129000	3.791877000	11.851976000
C	5.755710000	12.272157000	9.558279000
H	5.372935000	12.547004000	8.568566000
H	5.830861000	13.190105000	10.153371000
H	5.032570000	11.608818000	10.034704000
C	8.062922000	12.580638000	8.710922000
H	9.072940000	12.176655000	8.581008000
H	8.146797000	13.489823000	9.316111000
H	7.680519000	12.878709000	7.728561000
C	3.199368000	3.266654000	14.668885000
C	3.570755000	5.583074000	9.869805000
H	2.578559000	5.737050000	10.302615000
H	4.224120000	5.307011000	10.699319000
C	2.273686000	3.538268000	9.241534000
H	2.280566000	2.593901000	8.668871000
H	2.362904000	3.255961000	10.295616000
C	3.481250000	4.408130000	8.876095000
H	4.389125000	3.793126000	8.891736000
H	3.371010000	4.758451000	7.842601000
C	4.829737000	4.939459000	13.858899000
C	6.392918000	6.727781000	14.667115000
H	5.754679000	7.497167000	14.230468000
H	7.426403000	7.096079000	14.673853000
H	6.082100000	6.567415000	15.705466000
C	7.247669000	4.407050000	14.531123000
H	7.000402000	4.231376000	15.583370000
H	8.266813000	4.807238000	14.499166000
H	7.258574000	3.442115000	14.012418000
C	8.239320000	8.823373000	5.313085000
C	0.516616000	4.100663000	7.692800000
H	1.292284000	4.367401000	6.967966000
H	-0.337790000	4.764626000	7.522692000
H	0.194206000	3.064958000	7.469873000
C	-0.016892000	3.976841000	10.028816000
H	-0.268559000	2.897241000	10.069379000
H	-0.931959000	4.473767000	9.684356000
C	2.861495000	1.963611000	15.404531000
C	0.837363000	8.069498000	9.635993000

H	1.244412000	7.061763000	9.551119000
H	0.140570000	8.207822000	8.789079000
C	3.268991000	7.341246000	8.195538000
H	2.426488000	6.661668000	8.042288000
H	3.873548000	7.292373000	7.280102000
C	6.818995000	5.646359000	12.438657000
H	6.753086000	4.727816000	11.844808000
H	7.869666000	5.958631000	12.462716000
H	6.243601000	6.424995000	11.940072000
C	4.115065000	1.232327000	15.904229000
H	4.786600000	0.965123000	15.081107000
H	3.825221000	0.303876000	16.407959000
H	4.679420000	1.834514000	16.624269000
C	1.970412000	2.274276000	16.623315000
H	2.485814000	2.940469000	17.322983000
H	1.711555000	1.352374000	17.157527000
H	1.035380000	2.760919000	16.328669000
C	9.750348000	9.048628000	5.517746000
H	9.959028000	10.030270000	5.954066000
H	10.281638000	8.989934000	4.560230000
H	10.169867000	8.290801000	6.187625000
C	2.105361000	1.016281000	14.452063000
H	1.173876000	1.463659000	14.091038000
H	1.848334000	0.080244000	14.962173000
H	2.718707000	0.770083000	13.578662000
C	8.049072000	7.454304000	4.645709000
H	8.451699000	6.642698000	5.261269000
H	8.576748000	7.433499000	3.686160000
H	6.993034000	7.241826000	4.446483000
C	7.689225000	9.901696000	4.358912000
H	6.616593000	9.760978000	4.188098000
H	8.198058000	9.853979000	3.388685000
H	7.832571000	10.909775000	4.759927000
C	3.270901000	11.275897000	12.100663000
H	3.142206000	12.088790000	11.396407000
C	2.099517000	10.663368000	12.713325000
H	1.109455000	10.860091000	12.319358000
C	4.554090000	11.130184000	12.763207000
H	5.415867000	11.631029000	12.344497000
U	3.033318000	11.865775000	14.692304000
O	3.232082000	13.906340000	13.845664000
O	1.171860000	11.652022000	15.870107000
N	6.504864000	14.584468000	15.114898000
N	2.811212000	13.805840000	17.036890000
N	4.718860000	11.477342000	17.007585000
C	0.404723000	11.785996000	16.961124000
N	6.083662000	16.174873000	17.648053000
C	6.838059000	12.221648000	15.850589000
H	7.632424000	11.482017000	16.006219000
H	6.258025000	11.867155000	14.990941000
C	-0.504293000	10.780588000	17.402459000
C	-1.126664000	12.196289000	19.336550000
C	-0.687384000	9.457344000	16.645439000
C	3.802610000	11.773991000	18.130061000
H	4.266421000	11.464629000	19.084922000
H	2.906084000	11.159171000	17.994874000
C	-1.228656000	11.027805000	18.571090000
H	-1.918220000	10.262568000	18.909109000
C	6.088528000	14.326621000	13.722418000
H	6.982793000	14.343878000	13.067492000
H	5.697780000	13.307232000	13.685398000
C	7.479481000	13.574087000	15.525339000

H	8.262491000	13.438737000	14.754001000
H	7.991939000	13.932350000	16.424311000
C	0.511230000	12.976881000	17.709051000
C	1.371792000	14.101754000	17.212195000
H	1.005642000	14.434575000	16.235597000
H	1.247614000	14.952530000	17.901658000
C	5.120068000	10.070025000	17.134279000
H	5.756138000	9.777362000	16.298860000
H	5.672600000	9.902609000	18.074524000
H	4.235240000	9.430391000	17.131858000
C	1.257367000	15.564690000	12.332843000
C	-1.088425000	9.743084000	15.185001000
H	-0.357366000	10.383182000	14.689991000
H	-1.178409000	8.804348000	14.624293000
H	-2.061328000	10.246301000	15.151388000
C	3.664860000	14.959588000	13.142976000
C	3.324175000	16.899477000	11.723493000
H	2.644769000	17.542208000	11.180683000
C	5.529601000	16.334918000	12.368763000
H	6.606794000	16.490322000	12.337575000
C	-0.239066000	13.162622000	18.872323000
H	-0.117635000	14.101361000	19.405960000
C	5.059677000	15.244884000	13.103004000
C	6.996001000	15.944708000	15.301943000
H	7.954895000	16.118428000	14.770222000
H	6.272691000	16.613912000	14.828888000
C	0.626373000	8.655001000	16.717929000
H	0.854865000	8.398699000	17.758670000
H	0.542779000	7.717457000	16.154502000
H	1.464003000	9.225161000	16.312152000
C	-1.791136000	8.572791000	17.248036000
H	-2.766011000	9.072376000	17.246056000
H	-1.886032000	7.661990000	16.646698000
H	-1.563593000	8.261868000	18.273441000
C	4.685899000	17.201147000	11.676210000
C	3.501635000	15.042822000	16.610365000
H	4.512379000	14.770692000	16.294633000
H	2.993758000	15.396076000	15.711389000
C	4.901254000	16.980941000	17.338130000
H	4.912858000	17.935794000	17.893473000
H	4.918085000	17.245836000	16.275752000
C	3.610942000	16.202484000	17.617700000
H	2.754052000	16.883528000	17.547130000
H	3.627644000	15.842045000	18.653784000
C	2.778079000	15.809614000	12.413373000
C	0.976583000	14.202265000	11.673224000
H	1.422566000	13.387099000	12.244519000
H	-0.104353000	14.027370000	11.607631000
H	1.387124000	14.175175000	10.657658000
C	0.524269000	16.626757000	11.495399000
H	0.860310000	16.641274000	10.453039000
H	-0.546386000	16.395242000	11.490117000
H	0.638311000	17.634187000	11.909912000
C	-1.970277000	12.363274000	20.606765000
C	6.476198000	16.325727000	19.037339000
H	5.622562000	16.127539000	19.693179000
H	7.261648000	15.605042000	19.289372000
H	6.852357000	17.339209000	19.278065000
C	7.215385000	16.381050000	16.754840000
H	7.539654000	17.441414000	16.722249000
H	8.063298000	15.833276000	17.183871000
C	5.259523000	18.393356000	10.899292000

C	5.921320000	12.333946000	17.069529000
H	5.603938000	13.374555000	17.132800000
H	6.481873000	12.110859000	17.996022000
C	3.407409000	13.237288000	18.260200000
H	4.282907000	13.826257000	18.547978000
H	2.705681000	13.301949000	19.101607000
C	0.634052000	15.593959000	13.737035000
H	0.788675000	16.567905000	14.214580000
H	-0.445413000	15.409041000	13.684583000
H	1.083452000	14.826079000	14.364871000
C	4.168461000	19.213305000	10.197777000
H	3.447808000	19.627809000	10.910877000
H	4.623921000	20.053659000	9.662813000
H	3.618079000	18.614567000	9.464232000
C	6.242002000	17.886583000	9.825235000
H	5.735291000	17.225909000	9.114083000
H	6.669446000	18.727317000	9.265874000
H	7.071625000	17.325435000	10.266432000
C	-3.467359000	12.285375000	20.247951000
H	-3.725713000	11.325387000	19.790455000
H	-4.085622000	12.403467000	21.145950000
H	-3.739815000	13.075260000	19.540190000
C	6.009114000	19.326739000	11.870675000
H	6.828043000	18.807548000	12.378560000
H	6.438454000	20.180541000	11.332893000
H	5.332474000	19.714859000	12.639525000
C	-1.716450000	13.710720000	21.296614000
H	-1.972075000	14.554553000	20.646762000
H	-2.335422000	13.788033000	22.196925000
H	-0.670768000	13.819817000	21.604281000
C	-1.628246000	11.241523000	21.607002000
H	-0.570330000	11.278413000	21.887874000
H	-2.226919000	11.342892000	22.520143000
H	-1.826511000	10.250396000	21.187581000

LUC6H6UL complex (s=3)

U	3.861002000	8.715347000	11.528919000
O	3.983261000	6.772934000	12.582800000
O	5.623631000	9.237015000	10.287976000
N	0.702605000	5.824102000	11.533857000
N	4.140881000	6.862605000	9.297438000
N	2.045237000	8.999206000	9.396680000
C	6.289895000	9.171490000	9.128756000
N	1.106650000	4.274115000	8.946068000
C	0.130952000	8.145293000	10.807344000
H	-0.721577000	8.830545000	10.734085000
H	0.775973000	8.560376000	11.592337000
C	7.037166000	10.261948000	8.595434000
C	3.529290000	9.366333000	14.205849000
H	3.622260000	8.561109000	14.925580000
C	7.586272000	8.906033000	6.597287000
C	7.187378000	11.587591000	9.355272000
C	2.863100000	8.722345000	8.197424000
H	2.276178000	8.930860000	7.283834000
H	3.707500000	9.419315000	8.205270000
C	7.646381000	10.085731000	7.350870000
H	8.208913000	10.917569000	6.941419000
C	1.194922000	6.077733000	12.904481000
H	0.357762000	5.949821000	13.619406000
H	1.485258000	7.130970000	12.953967000
C	-0.377936000	6.761469000	11.223267000
H	-1.076300000	6.860151000	12.075807000

H	-0.965258000	6.353347000	10.393889000
C	6.244072000	7.963517000	8.400201000
C	5.590293000	6.753204000	9.002097000
H	6.079849000	6.509676000	9.951473000
H	5.764542000	5.900242000	8.326746000
C	1.552256000	10.378850000	9.291242000
H	0.957279000	10.636733000	10.167211000
H	0.930922000	10.509104000	8.389264000
H	2.396167000	11.070956000	9.234598000
C	4.732497000	10.055068000	13.730648000
H	5.714319000	9.796376000	14.101924000
C	6.201841000	5.319796000	13.918602000
C	7.816309000	11.313419000	10.735935000
H	7.233703000	10.582293000	11.298489000
H	7.883160000	12.241673000	11.317093000
H	8.831900000	10.919196000	10.618376000
C	2.280332000	9.650849000	13.655613000
H	1.406227000	9.137371000	14.037174000
C	3.703956000	5.663635000	13.273401000
C	4.345728000	3.713308000	14.567685000
H	5.126199000	3.120940000	15.025717000
C	2.050199000	4.080036000	14.118310000
H	0.999542000	3.816438000	14.228219000
C	6.877041000	7.848627000	7.160243000
H	6.810672000	6.893733000	6.645615000
C	2.351183000	5.241977000	13.405648000
C	0.308873000	4.431522000	11.343907000
H	-0.600173000	4.174857000	11.926433000
H	1.108292000	3.814045000	11.760926000
C	5.805301000	12.252045000	9.501072000
H	5.401175000	12.508016000	8.514852000
H	5.876900000	13.179046000	10.082755000
H	5.100982000	11.583479000	9.999314000
C	8.101601000	12.589532000	8.633131000
H	9.115183000	12.197309000	8.495515000
H	8.179655000	13.501047000	9.235634000
H	7.707686000	12.880476000	7.653159000
C	3.027708000	3.270504000	14.694280000
C	3.655736000	5.560365000	9.813705000
H	2.656260000	5.723397000	10.227709000
H	4.295094000	5.295644000	10.657594000
C	2.344954000	3.527574000	9.181309000
H	2.349868000	2.576096000	8.620590000
H	2.404002000	3.258083000	10.240842000
C	3.575592000	4.373096000	8.834745000
H	4.473175000	3.745233000	8.884814000
H	3.499692000	4.709451000	7.793654000
C	4.722740000	4.886527000	13.902399000
C	6.329040000	6.668427000	14.652419000
H	5.738551000	7.438528000	14.153801000
H	7.376542000	6.994151000	14.671190000
H	5.983009000	6.577160000	15.688082000
C	7.107634000	4.317093000	14.652947000
H	6.827306000	4.195863000	15.704746000
H	8.138312000	4.687312000	14.630743000
H	7.104872000	3.329513000	14.178619000
C	8.293841000	8.820936000	5.238836000
C	0.642589000	4.108327000	7.579891000
H	1.442933000	4.359235000	6.876588000
H	-0.196546000	4.783792000	7.381460000
H	0.311509000	3.076291000	7.353545000
C	0.038094000	4.000119000	9.898450000

H	-0.225331000	2.923335000	9.934636000
H	-0.860376000	4.503990000	9.521746000
C	2.635156000	1.989372000	15.441305000
C	0.906452000	8.063502000	9.490469000
H	1.290471000	7.045248000	9.413914000
H	0.228045000	8.219755000	8.631954000
C	3.375545000	7.293435000	8.109485000
H	2.534052000	6.611675000	7.961395000
H	3.991597000	7.226823000	7.203360000
C	6.752388000	5.459599000	12.489812000
H	6.676021000	4.511081000	11.946519000
H	7.808743000	5.752422000	12.513045000
H	6.199241000	6.221177000	11.941023000
C	3.855112000	1.234411000	15.987217000
H	4.541338000	0.936151000	15.187204000
H	3.527640000	0.322644000	16.498326000
H	4.415589000	1.834487000	16.711978000
C	1.719128000	2.342899000	16.629485000
H	2.232066000	3.006943000	17.332964000
H	1.421358000	1.436802000	17.170485000
H	0.805777000	2.848664000	16.300853000
C	9.804513000	9.062912000	5.425808000
H	10.007309000	10.047862000	5.857476000
H	10.326102000	9.007529000	4.462736000
H	10.239439000	8.311459000	6.093032000
C	1.880908000	1.045684000	14.483669000
H	0.969829000	1.509356000	14.092394000
H	1.588577000	0.123745000	15.000433000
H	2.510106000	0.771858000	13.629977000
C	8.110827000	7.448465000	4.576382000
H	8.529016000	6.642405000	5.188841000
H	8.627785000	7.431254000	3.610928000
H	7.054899000	7.224392000	4.389426000
C	7.721443000	9.890741000	4.288109000
H	6.648391000	9.738233000	4.130296000
H	8.219218000	9.845930000	3.311988000
H	7.858739000	10.901408000	4.684740000
C	3.312315000	11.375953000	12.178959000
H	3.224673000	12.167946000	11.444051000
C	2.101995000	10.733071000	12.700313000
H	1.126360000	10.951541000	12.289287000
C	4.565047000	11.081767000	12.715957000
H	5.441081000	11.580899000	12.320063000
U	3.044859000	11.983488000	14.801101000
O	3.161260000	13.979072000	13.840735000
O	1.197347000	11.595653000	15.975044000
N	6.431465000	14.627601000	15.120505000
N	2.770315000	13.843306000	17.080263000
N	4.688423000	11.545759000	17.086676000
C	0.445645000	11.724223000	17.072833000
N	5.995998000	16.237090000	17.654963000
C	6.790282000	12.276013000	15.887247000
H	7.592433000	11.542960000	16.033784000
H	6.197307000	11.902314000	15.043338000
C	-0.411569000	10.693206000	17.557655000
C	-1.043931000	12.122029000	19.478406000
C	-0.567206000	9.357757000	16.816561000
C	3.792844000	11.852759000	18.220391000
H	4.279264000	11.574293000	19.173861000
H	2.902894000	11.224483000	18.115078000
C	-1.115266000	10.933415000	18.739975000
H	-1.762769000	10.147011000	19.111925000

C	6.027342000	14.364588000	13.723934000
H	6.928509000	14.375168000	13.078681000
H	5.636565000	13.343830000	13.688457000
C	7.416935000	13.628746000	15.536040000
H	8.193738000	13.489351000	14.759429000
H	7.935777000	14.002405000	16.425022000
C	0.512602000	12.938324000	17.789803000
C	1.316062000	14.083020000	17.243477000
H	0.935838000	14.356158000	16.252549000
H	1.155163000	14.955823000	17.896423000
C	5.072227000	10.132210000	17.190291000
H	5.701600000	9.847762000	16.346670000
H	5.625865000	9.939813000	18.125271000
H	4.178981000	9.502993000	17.181467000
C	1.212119000	15.637177000	12.322012000
C	-1.053583000	9.624700000	15.378208000
H	-0.386694000	10.312433000	14.855901000
H	-1.114260000	8.685264000	14.814160000
H	-2.055095000	10.069066000	15.393553000
C	3.611258000	15.017583000	13.131158000
C	3.292730000	16.946503000	11.692465000
H	2.620842000	17.591417000	11.142673000
C	5.492107000	16.363119000	12.345369000
H	6.570879000	16.507643000	12.314798000
C	-0.216032000	13.117987000	18.967725000
H	-0.125253000	14.073902000	19.476628000
C	5.009197000	15.285156000	13.089444000
C	6.906839000	15.994703000	15.306184000
H	7.863092000	16.177926000	14.773198000
H	6.175773000	16.654515000	14.831975000
C	0.783890000	8.616765000	16.816526000
H	1.078726000	8.367659000	17.842386000
H	0.713125000	7.677621000	16.253750000
H	1.569533000	9.229797000	16.370877000
C	-1.595314000	8.425213000	17.476337000
H	-2.593335000	8.874958000	17.516521000
H	-1.672362000	7.504000000	16.888292000
H	-1.303888000	8.138766000	18.492730000
C	4.658499000	17.231696000	11.643436000
C	3.423610000	15.095432000	16.636918000
H	4.437393000	14.840750000	16.314953000
H	2.898248000	15.428277000	15.740291000
C	4.812949000	17.043486000	17.346527000
H	4.827158000	18.000051000	17.898529000
H	4.826399000	17.304346000	16.283056000
C	3.523091000	16.265757000	17.632488000
H	2.664333000	16.943025000	17.550906000
H	3.537088000	15.916958000	18.672346000
C	2.734717000	15.870268000	12.393308000
C	0.916293000	14.261637000	11.694960000
H	1.338200000	13.454402000	12.295478000
H	-0.166821000	14.104206000	11.620820000
H	1.336560000	14.202331000	10.684517000
C	0.489022000	16.687024000	11.460997000
H	0.828456000	16.677205000	10.419572000
H	-0.583455000	16.464111000	11.457588000
H	0.609793000	17.702213000	11.854358000
C	-1.854449000	12.276901000	20.771449000
C	6.394266000	16.394045000	19.042279000
H	5.542839000	16.200427000	19.702312000
H	7.179560000	15.673383000	19.294764000
H	6.772876000	17.408055000	19.276330000

C	7.125218000	16.438769000	16.756984000
H	7.447191000	17.499501000	16.717160000
H	7.975088000	15.895899000	17.188292000
C	5.246760000	18.409617000	10.855563000
C	5.899064000	12.391629000	17.126609000
H	5.590116000	13.435114000	17.199754000
H	6.476498000	12.161529000	18.040856000
C	3.382421000	13.314799000	18.315435000
H	4.254809000	13.920999000	18.574573000
H	2.688423000	13.398858000	19.161373000
C	0.593254000	15.706745000	13.727841000
H	0.756908000	16.691830000	14.178803000
H	-0.487860000	15.529606000	13.681962000
H	1.037285000	14.951411000	14.374892000
C	4.165604000	19.236567000	10.146835000
H	3.449944000	19.665852000	10.856227000
H	4.630910000	20.066837000	9.604626000
H	3.607930000	18.638165000	9.418536000
C	6.222765000	17.881433000	9.785947000
H	5.707632000	17.221618000	9.080047000
H	6.661302000	18.711929000	9.219902000
H	7.044975000	17.313101000	10.231945000
C	-3.357041000	12.119086000	20.465924000
H	-3.582450000	11.139831000	20.032522000
H	-3.949248000	12.221402000	21.383251000
H	-3.692762000	12.882449000	19.756263000
C	6.007876000	19.342866000	11.818116000
H	6.820655000	18.818664000	12.330726000
H	6.447294000	20.186562000	11.272541000
H	5.336062000	19.745975000	12.583465000
C	-1.644701000	13.650073000	21.424557000
H	-1.959846000	14.465815000	20.764984000
H	-2.239011000	13.718702000	22.342057000
H	-0.596583000	13.815604000	21.696869000
C	-1.426380000	11.195631000	21.783321000
H	-0.363069000	11.289838000	22.028272000
H	-2.000065000	11.288633000	22.713303000
H	-1.588779000	10.186790000	21.391463000

^{Ad}LUC6H5CH3UL^{Ad} (s=2)

U	3.558291000	19.304035000	3.412244000
U	4.511154000	15.653990000	5.437310000
N	2.414551000	19.617768000	1.407492000
N	3.973200000	21.474708000	4.144299000
N	5.744781000	13.841241000	4.674623000
N	4.018394000	15.035476000	7.614699000
C	2.673507000	17.452385000	4.982882000
C	3.762852000	18.163683000	5.637466000
C	5.129482000	18.011912000	5.130008000
C	5.284467000	17.545278000	3.741598000
C	4.199299000	16.787277000	3.129137000
C	2.882216000	16.748599000	3.727209000
C	1.211636000	20.179325000	1.802829000
C	0.549799000	21.296353000	1.224374000
C	-0.612335000	21.823382000	1.771866000
C	-1.147610000	21.270315000	2.950225000
C	-0.547175000	20.169590000	3.549081000
C	0.606102000	19.623882000	2.959716000
C	4.948468000	21.850670000	3.238125000
C	5.962636000	20.891165000	2.978293000
C	6.937590000	21.070470000	1.978698000
C	6.939379000	22.253407000	1.252340000
C	5.987022000	23.258218000	1.515346000

C	5.005498000	23.049359000	2.473310000
C	6.925049000	13.962505000	5.386286000
C	7.624380000	12.940359000	6.084058000
C	8.748940000	13.218956000	6.848710000
C	9.202907000	14.546240000	6.975396000
C	8.565405000	15.579276000	6.300172000
C	7.452917000	15.275319000	5.495372000
C	3.118345000	13.992166000	7.467629000
C	2.114550000	14.154702000	6.477009000
C	1.212911000	13.128450000	6.142666000
C	1.282159000	11.924213000	6.833058000
C	2.230199000	11.736490000	7.855166000
C	3.134436000	12.746983000	8.151682000
C	2.924956000	20.501919000	-2.821975000
C	2.901642000	19.651739000	0.020122000
C	1.819503000	19.195772000	-0.982929000
C	3.476166000	21.018873000	-0.427056000
C	4.058833000	18.631416000	-0.058565000
C	2.392427000	19.121096000	-2.407778000
C	3.538383000	18.099258000	-2.448067000
C	4.039423000	20.933456000	-1.856226000
C	5.182613000	19.910943000	-1.899390000
C	4.643506000	18.537772000	-1.475756000
C	3.358608000	24.416958000	6.543640000
C	3.219723000	22.443268000	4.955754000
C	2.549376000	21.645338000	6.095784000
C	4.152455000	23.483243000	5.615417000
C	2.091323000	23.174010000	4.188794000
C	2.702027000	23.594465000	7.661700000
C	1.752004000	22.561814000	7.037540000
C	0.656912000	23.289088000	6.244467000
C	1.309933000	24.111222000	5.125036000
C	2.269984000	25.142520000	5.737214000
C	5.979818000	10.867918000	2.250444000
C	5.340547000	12.616391000	3.972092000
C	4.173766000	13.023282000	3.046875000
C	6.473072000	12.066383000	3.077928000
C	3.669222000	11.840000000	2.209104000
C	4.822692000	11.311193000	1.342742000
C	4.814318000	11.483392000	4.887832000
C	3.176485000	10.724984000	3.141162000
C	4.331566000	10.285470000	4.051228000
C	5.490850000	9.756718000	3.192536000
C	3.684790000	15.409754000	10.059182000
C	4.688303000	15.347307000	8.886921000
C	5.296435000	16.757484000	8.725405000
C	5.853403000	14.395696000	9.256610000
C	6.538789000	14.847953000	10.558138000
C	7.130278000	16.250140000	10.363481000
C	4.977994000	17.264870000	11.154252000
C	6.000175000	17.225616000	10.008002000
C	4.384137000	15.862169000	11.351781000
C	5.509661000	14.876404000	11.699372000
C	1.728186000	16.129923000	2.969591000
H	1.986849000	15.134642000	2.591788000
H	0.852656000	16.018219000	3.616161000
H	1.427690000	16.732584000	2.103511000
H	4.367082000	16.292865000	2.179579000
H	6.274197000	17.489627000	3.300731000
H	5.926731000	18.587278000	5.590464000
H	3.592377000	18.619642000	6.604503000
H	1.694535000	17.432572000	5.451338000

C	2.257614000	10.436737000	8.617292000
H	1.318630000	10.279601000	9.160915000
H	2.382535000	9.583190000	7.941595000
H	3.073723000	10.412601000	9.344315000
C	0.149649000	13.352809000	5.099499000
H	0.578146000	13.403036000	4.092339000
H	-0.587934000	12.545744000	5.106627000
H	-0.382998000	14.294852000	5.270128000
H	0.572517000	11.131611000	6.605690000
H	3.868293000	12.569495000	8.927198000
C	9.486163000	12.111606000	7.556795000
H	9.522505000	12.287733000	8.637834000
H	9.012882000	11.140325000	7.390165000
H	10.523565000	12.044411000	7.208843000
C	9.083487000	16.991993000	6.368656000
H	8.363727000	17.658441000	6.858279000
H	10.022031000	17.046795000	6.926798000
H	9.265223000	17.396422000	5.366514000
H	7.280468000	11.915209000	6.025031000
H	10.082931000	14.757093000	7.579218000
H	7.090930000	16.033718000	4.799160000
H	1.931843000	15.159518000	6.086394000
C	7.994923000	20.020370000	1.759554000
H	8.532525000	19.800170000	2.688918000
H	7.556696000	19.078162000	1.410572000
H	8.729529000	20.342746000	1.016811000
C	6.033772000	24.547715000	0.737231000
H	6.994276000	25.057212000	0.876517000
H	5.921396000	24.365323000	-0.337605000
H	5.241180000	25.234441000	1.046098000
H	7.703402000	22.422589000	0.496691000
H	6.094807000	20.076647000	3.692379000
H	4.267657000	23.825195000	2.634047000
H	0.951694000	18.652991000	3.315668000
H	0.953628000	21.760340000	0.333494000
H	-2.057598000	21.687967000	3.375644000
C	-1.303618000	22.991266000	1.116561000
H	-0.748730000	23.351190000	0.246066000
H	-2.310674000	22.715333000	0.782290000
H	-1.416649000	23.828314000	1.814599000
C	-1.149478000	19.522049000	4.768976000
H	-2.087977000	20.005048000	5.053931000
H	-1.360030000	18.461330000	4.590746000
H	-0.471344000	19.575848000	5.628621000
H	1.295467000	21.953653000	7.829716000
H	5.456589000	17.799870000	-1.482514000
H	6.807187000	10.491865000	1.634283000
H	7.341846000	14.140287000	10.802841000
H	-0.045722000	22.565350000	5.812520000
H	0.079429000	23.943424000	6.911634000
H	0.532580000	24.627790000	4.546756000
H	4.669367000	24.072935000	4.850306000
H	3.471790000	23.089025000	8.260257000
H	3.328559000	21.108858000	6.650631000
H	4.044195000	25.153898000	6.982544000
H	2.729949000	25.747746000	4.944102000
H	4.928508000	22.949792000	6.180253000
H	2.148021000	24.253533000	8.343872000
H	1.718054000	25.834576000	6.387450000
H	1.878821000	20.886664000	5.662592000
H	2.498380000	23.755087000	3.355567000
H	1.418234000	22.429530000	3.748406000

H	1.597619000	18.809980000	-3.098490000
H	4.418877000	21.921079000	-2.149967000
H	0.964653000	19.880683000	-0.960005000
H	4.260841000	21.323283000	0.275808000
H	4.854692000	18.927422000	0.645156000
H	3.941924000	18.020172000	-3.466582000
H	2.110163000	21.238654000	-2.811975000
H	5.606863000	19.854947000	-2.911111000
H	1.442974000	18.213020000	-0.669279000
H	3.308660000	20.464971000	-3.850478000
H	2.708299000	21.797638000	-0.391155000
H	3.684584000	17.653194000	0.265193000
H	5.990256000	20.226742000	-1.226505000
H	3.165598000	17.103460000	-2.173270000
H	2.847261000	12.177049000	1.563541000
H	3.984197000	9.494458000	4.728777000
H	6.821855000	12.871189000	2.417467000
H	5.162953000	8.885784000	2.608978000
H	5.164271000	12.091983000	0.649919000
H	2.336797000	11.080877000	3.751856000
H	4.511487000	13.838801000	2.396354000
H	5.593469000	11.146759000	5.578462000
H	7.330154000	11.769587000	3.693464000
H	6.314624000	9.418188000	3.835351000
H	4.476735000	10.468378000	0.729296000
H	3.348560000	13.415438000	3.664897000
H	3.995821000	11.871800000	5.505799000
H	2.810101000	9.873641000	2.551623000
H	3.649804000	15.885175000	12.167781000
H	6.412646000	18.230306000	9.846219000
H	4.494399000	17.455425000	8.457324000
H	6.016964000	16.749366000	7.891175000
H	5.462005000	17.605836000	12.079450000
H	4.179925000	17.984081000	10.926121000
H	5.994977000	15.173954000	12.638797000
H	5.094802000	13.871855000	11.858845000
H	7.880716000	16.229555000	9.562889000
H	7.640457000	16.580967000	11.278292000
H	5.497682000	13.368813000	9.380129000
H	6.576736000	14.379972000	8.432943000
H	3.217463000	14.429870000	10.210866000
H	2.878722000	16.106798000	9.794417000

^{Ad}LUC6H5CH3UL^{Ad} (s=3)

U	3.414082000	19.488075000	3.400539000
U	4.441555000	15.532412000	5.520779000
N	2.264638000	19.650522000	1.352079000
N	4.032588000	21.630705000	4.138244000
N	5.761572000	13.801723000	4.690601000
N	3.923096000	14.954644000	7.719637000
C	2.577455000	17.418757000	5.082623000
C	3.671461000	18.057426000	5.723230000
C	4.990193000	18.010418000	5.157690000
C	5.147998000	17.427775000	3.802561000
C	4.051345000	16.751265000	3.197822000
C	2.778448000	16.641838000	3.836503000
C	1.117605000	20.269587000	1.794687000
C	0.459729000	21.401232000	1.232448000
C	-0.632462000	21.994467000	1.848626000
C	-1.108784000	21.496835000	3.078389000
C	-0.520709000	20.382726000	3.661650000
C	0.570256000	19.774053000	3.013699000

C	4.996038000	21.898190000	3.190828000
C	5.902689000	20.835564000	2.910418000
C	6.854365000	20.902841000	1.874929000
C	6.935253000	22.061142000	1.114551000
C	6.090091000	23.156447000	1.386108000
C	5.136717000	23.068153000	2.389738000
C	6.905389000	13.972078000	5.446787000
C	7.650860000	12.974249000	6.133747000
C	8.730459000	13.299663000	6.942304000
C	9.092308000	14.649164000	7.127117000
C	8.404323000	15.661796000	6.472237000
C	7.333991000	15.314900000	5.627252000
C	3.128615000	13.846565000	7.499307000
C	2.155445000	13.963005000	6.468207000
C	1.346160000	12.879699000	6.073039000
C	1.488705000	11.662854000	6.726440000
C	2.421236000	11.511106000	7.770948000
C	3.230723000	12.576659000	8.133442000
C	2.727774000	20.510265000	-2.885447000
C	2.716468000	19.664134000	-0.042304000
C	1.600138000	19.251990000	-1.026763000
C	3.335881000	21.007456000	-0.500349000
C	3.828596000	18.596011000	-0.138572000
C	2.146710000	19.152086000	-2.460851000
C	3.251601000	18.086376000	-2.518301000
C	3.872833000	20.899216000	-1.937612000
C	4.974593000	19.832281000	-1.995016000
C	4.388449000	18.480314000	-1.563650000
C	3.581378000	24.691513000	6.420627000
C	3.343260000	22.667232000	4.911688000
C	2.641703000	21.942728000	6.082398000
C	4.326125000	23.687014000	5.526414000
C	2.246214000	23.418281000	4.120232000
C	2.895499000	23.940815000	7.571777000
C	1.894727000	22.929088000	6.993825000
C	0.829239000	23.673617000	6.176084000
C	1.511163000	24.423064000	5.023260000
C	2.520227000	25.432766000	5.591752000
C	6.275327000	10.888989000	2.233175000
C	5.458678000	12.576533000	3.941258000
C	4.326722000	12.941937000	2.955264000
C	6.662550000	12.098545000	3.099923000
C	3.927240000	11.747116000	2.077173000
C	5.148619000	11.286132000	1.267935000
C	4.942316000	11.404336000	4.811038000
C	3.439688000	10.596577000	2.968531000
C	4.563945000	10.198237000	3.934928000
C	5.792835000	9.740900000	3.134067000
C	3.587555000	15.082606000	10.197893000
C	4.563663000	15.243988000	9.011295000
C	4.982035000	16.730318000	8.968514000
C	5.844127000	14.416689000	9.279469000
C	6.500994000	14.838074000	10.604650000
C	6.906623000	16.316323000	10.525686000
C	4.669642000	16.978600000	11.439789000
C	5.655002000	17.170106000	10.277391000
C	4.259017000	15.500785000	11.516927000
C	5.506870000	14.636480000	11.758018000
C	1.594531000	16.060318000	3.100452000
H	1.894985000	15.192713000	2.503793000
H	0.821637000	15.726087000	3.799266000
H	1.124232000	16.774505000	2.410879000

H	4.197677000	16.270924000	2.234980000
H	6.122121000	17.408450000	3.327759000
H	5.799900000	18.571234000	5.609953000
H	3.516904000	18.556319000	6.674550000
H	1.609033000	17.391558000	5.572307000
C	2.533734000	10.186703000	8.480390000
H	1.590765000	9.923893000	8.973913000
H	2.762058000	9.378732000	7.776191000
H	3.318229000	10.201744000	9.241585000
C	0.295950000	13.054670000	5.008011000
H	0.744441000	13.191599000	4.017990000
H	-0.363440000	12.184085000	4.956929000
H	-0.326879000	13.934484000	5.204605000
H	0.850296000	10.826207000	6.451250000
H	3.956962000	12.428927000	8.922885000
C	9.520897000	12.219028000	7.633913000
H	9.547532000	12.379139000	8.717601000
H	9.097445000	11.228038000	7.450027000
H	10.560771000	12.209626000	7.286518000
C	8.824505000	17.101828000	6.604633000
H	8.043705000	17.702821000	7.085126000
H	9.735825000	17.198892000	7.200731000
H	9.016945000	17.550455000	5.623400000
H	7.378326000	11.931294000	6.029140000
H	9.939457000	14.895228000	7.763640000
H	6.948516000	16.070912000	4.938603000
H	1.892403000	14.967900000	6.122168000
C	7.802114000	19.754942000	1.644403000
H	8.311104000	19.468203000	2.571712000
H	7.273986000	18.866272000	1.278659000
H	8.568237000	20.012685000	0.908356000
C	6.226262000	24.416360000	0.570780000
H	7.227011000	24.850142000	0.681366000
H	6.081830000	24.214286000	-0.496586000
H	5.496910000	25.173055000	0.871760000
H	7.680353000	22.139704000	0.325897000
H	6.003006000	20.040826000	3.653827000
H	4.485572000	23.916984000	2.558665000
H	0.867814000	18.777723000	3.348661000
H	0.813877000	21.820091000	0.298683000
H	-1.966458000	21.967604000	3.553663000
C	-1.313816000	23.180327000	1.215828000
H	-0.839054000	23.462319000	0.272353000
H	-2.369012000	22.964869000	1.011233000
H	-1.288646000	24.051935000	1.879759000
C	-1.076517000	19.783364000	4.927371000
H	-1.948240000	20.339959000	5.281516000
H	-1.385831000	18.743725000	4.768601000
H	-0.332438000	19.778229000	5.732211000
H	1.415363000	22.375034000	7.811788000
H	5.171955000	17.711195000	-1.584706000
H	7.152946000	10.562895000	1.659498000
H	7.393291000	14.221059000	10.773671000
H	0.092394000	22.965541000	5.775845000
H	0.284705000	24.377875000	6.819791000
H	0.753823000	24.952713000	4.430424000
H	4.866074000	24.221034000	4.736705000
H	3.646013000	23.423955000	8.184767000
H	3.397455000	21.386367000	6.652445000
H	4.300798000	25.413604000	6.828891000
H	3.000824000	25.987004000	4.773996000
H	5.080338000	23.140287000	6.107987000

H	2.375929000	24.648735000	8.231691000
H	2.003338000	26.173018000	6.217266000
H	1.930694000	21.206214000	5.674062000
H	2.678345000	23.948126000	3.265446000
H	1.541592000	22.685532000	3.707627000
H	1.329808000	18.872081000	-3.139043000
H	4.286542000	21.870390000	-2.239884000
H	0.775168000	19.972455000	-0.991774000
H	4.143107000	21.282182000	0.190009000
H	4.640977000	18.857723000	0.559168000
H	3.635231000	17.992055000	-3.543231000
H	1.943202000	21.278930000	-2.864620000
H	5.382917000	19.759499000	-3.012229000
H	1.188225000	18.286321000	-0.704634000
H	3.094077000	20.456950000	-3.919631000
H	2.596747000	21.813247000	-0.451143000
H	3.414481000	17.633854000	0.189124000
H	5.802538000	20.116580000	-1.332795000
H	2.844618000	17.105712000	-2.237395000
H	3.124700000	12.053208000	1.392943000
H	4.219693000	9.380204000	4.581414000
H	7.003096000	12.930150000	2.468825000
H	5.541564000	8.863418000	2.523028000
H	5.487588000	12.091411000	0.602352000
H	2.552616000	10.904755000	3.536911000
H	4.664829000	13.779026000	2.331596000
H	5.700676000	11.097150000	5.538069000
H	7.502579000	11.834918000	3.752337000
H	6.596621000	9.435067000	3.817284000
H	4.877920000	10.435432000	0.628012000
H	3.451430000	13.291859000	3.526252000
H	4.075001000	11.744105000	5.389101000
H	3.145538000	9.737649000	2.350299000
H	3.547684000	15.359079000	12.341299000
H	5.936083000	18.228859000	10.201204000
H	4.087690000	17.336348000	8.776533000
H	5.668245000	16.889704000	8.121894000
H	5.133865000	17.294342000	12.383909000
H	3.782607000	17.609160000	11.292273000
H	5.974472000	14.908653000	12.713927000
H	5.223856000	13.577591000	11.831883000
H	7.632992000	16.459483000	9.715381000
H	7.396613000	16.628451000	11.457902000
H	5.616724000	13.346509000	9.310784000
H	6.545653000	14.562144000	8.449668000
H	3.239935000	14.046632000	10.272349000
H	2.699193000	15.699571000	10.007762000

I. References

1 G. A. Bain and J. F. Berry, *J. Chem. Educ.*, 2008, **85**, 532.
2 D. E. Bergbreiter and J. M. Killough, *J. Am. Chem. Soc.*, 1978, **100**, 2126–2134.
3 L. Maria, I. C. Santos, V. R. Sousa and J. Marçalo, *Inorg. Chem.*, 2015, **54**, 9115–9126.
4 L. Maria, I. C. Santos, L. G. Alves, J. Marçalo and A. M. Martins, *J. Organomet. Chem.*, 2013, **728**, 57–67.
5 J. Andrez, J. Pécaut, P.-A. Bayle and M. Mazzanti, *Angew. Chem.*, 2014, **126**, 10616–10620.
6 CrysAlisPro (Rigaku, V1.171.44.91a, 2025).
7 G. M. Sheldrick, *Acta Cryst A*, 2015, **71**, 3–8.
8 O. V. Dolomanov, L. J. Bourhis, R. J. Gildea, J. a. K. Howard and H. Puschmann, *J Appl Cryst*, 2009, **42**, 339–341.
9 G. M. Sheldrick, *Acta Cryst C*, 2015, **71**, 3–8.
10 Gaussian 09, Revision D.01: M. J. Frisch, G. W. Trucks, H. B. Schlegel, G. E. Scuseria, M. A. Robb, J. R. Cheesman, G. Scalmani, V. Barone, B. Mennucci, G. A. Petersson, H. Nakatsuji, M. Caricato, X. Li, H. P. Hratchian, A. F. Izmaylov, J. Bloino, G. Zheng, J. L. Sonnenberg, M. Hada, M. Ehara, K. Toyota, R. Fukuda, J. Hasegawa, M. Ishida, T. Nakajima, Y. Honda, O. Kitao, H. Nakai, T. Vreven, J. A., Jr. Montgomery, J. E. Peralta, F. Ogliaro, M. Bearpark, J. J. Heyd, E. Brothers, K. N. Kudin, V. N. Staroverov, R. Kobayashi, J. Normand, K. Raghavachari, J. C. Burant, S. S. Iyengar, J. Tomasi, M. Cossi, N. Rega, M. J. Millam, M. Klene, J. E. Knox, J. B. Cross, V. Bakken, C. Adamo, J. Jaramillo, R. Gomperts, R. E. Stratmann, O. Yazyev, A. J. Austin, R. Cammi, C. Pomelli, J. W. Ochterski, R. L. Martin, K. Morokuma, V. G. Zakrzewski, G. A. Voth, P. Salvador, J. J. Dannenberg, S. Dapprich, A. D. Daniels, O. Farkas, J. B. Foresman, J. V. Ortiz, J. Cioslowski and D. J. Fox, Gaussian Inc., 2009, Wallingford CT.
11 A. D. Becke, *J. Chem. Phys.*, 1993, **98**, 5648–5652.
12 K. Burke, J. P. Perdew and Y. Wang, in *Electronic Density Functional Theory: Recent Progress and New Directions*, eds. J. F. Dobson, G. Vignale and M. P. Das, Springer US, Boston, MA, 1998, pp. 81–111.
13 A. Moritz, X. Cao and M. Dolg, *Theor Chem Account*, 2007, **118**, 845–854.
14 P. C. Hariharan and J. A. Pople, *Theoret. Chim. Acta*, 1973, **28**, 213–222.
15 W. J. Hehre, R. Ditchfield and J. A. Pople, *J. Chem. Phys.*, 1972, **56**, 2257–2261.
